# Transcription Activator FgDDT Interacts With FgISW1 to Regulate Fungal Development and Pathogenicity in the Global Pathogen *Fusarium graminearum*

**DOI:** 10.1111/mpp.70076

**Published:** 2025-03-28

**Authors:** Xiaozhen Zhao, Yuxin Qiu, Aning Jiang, Yan Huang, Peixue Ma, Bingqin Yuan, Li Chen, Chengqi Zhang

**Affiliations:** ^1^ School of Plant Protection Anhui Agricultural University Hefei China; ^2^ Key Laboratory of Agri‐Products Quality and Biosafety (Anhui Agricultural University) Ministry of Education Hefei China; ^3^ Department of Plant Pathology, College of Plant Protection Nanjing Agricultural University, Key Laboratory of Monitoring and Management of Crop Diseases and Pest Insects, Ministry of Education Nanjing China

**Keywords:** DDT‐domain protein, imitation switch (ISWI) complex, MAPK signalling pathway, metabolic pathway, pathogenicity

## Abstract

Several DNA‐binding homeobox and different transcription factor (DDT)‐domain proteins form stable remodelling complexes with imitation switch (ISWI) chromatin remodelling factors. ISWI complexes have been reported to be involved in various biological processes in many eukaryotic species. However, in phytopathogenic fungi, the regulatory mechanisms underlying the functions of DDT‐domain proteins in ISWI complexes remain unclear. Here, chromatin immunoprecipitation‐sequencing (ChIP‐seq) assays were used to demonstrate that FgDDT from *Fusarium graminearum* was enriched within the promoter regions of genes associated with metabolic and MAPK signalling pathways, thereby activating their expression. Moreover, two additional ISWI genes, *FgISW1* and *FgISW2*, were identified and characterised, with subsequent analyses indicating that the ISWI components FgISW1 and FgDDT are essential for fungal development and pathogenicity rather than FgISW2. Further experiments revealed that FgDDT binds to FgISW1 to form an ISWI complex that activates the expression of functional genes in *F. graminearum*, consequently contributing to its pathogenicity and development. FgDDT was also observed as highly conserved in *Fusarium* species but exhibits low similarity to homologues in *Homo sapiens* and *Arabidopsis thaliana* , suggesting that functional studies of FgDDT are crucial to uncover its unique role within *Fusarium*. These findings provide a basis for further understanding the molecular mechanisms by which ISWI complexes function in fungi and contribute to their pathogenicity.

## Graphical abstract

FgDDT, serving as an Imitation Switch component, interacts with FgISW1 to activate the expression of functional genes, thereby affecting fungal development and pathogenicity in *Fusarium graminearum*.

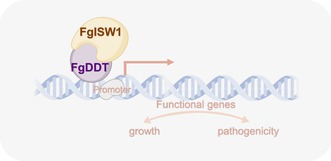

## 1 Introduction

*Fusarium graminearum* is a major globally distributed fungal pathogen that causes Fusarium head blight (FHB) in wheat ( *Triticum aestivum* ) and other grain crops (Palacios et al. 2021), leading to substantial losses in yields and mycotoxin contamination (Chen et al. 2019; Starkey et al. 2007) that threaten human and animal health. The scarcity of resistance genes, combined with complex genetic patterns, pathogen variability and environmental factors, contributes to the dearth of FHB‐resistant wheat varieties, posing a major challenge in managing this disease (Khan et al. 2020). Consequently, in‐depth studies of the molecular mechanisms underpinning *F. graminearum* infection will help to identify solutions to FHB. Transcriptional regulation is a mechanism for controlling cellular development and pathogenicity in plant pathogens (Hofstad et al. 2016; Luo et al. 2016; Tran et al. 2014). While many transcription factors (TFs) involved in the regulation of infection‐related morphogenesis in *F. graminearum* have been identified and characterised (Chen et al. 2019; Gu et al. 2022; Liu et al. 2019b; Kazan and Gardiner 2018; Son et al. 2011), the extent of understanding varies widely among these TFs. Specifically, TFs such as FgPacC, FgSR and TRI6 have seen significant progress in elucidating their regulatory mechanisms, including histone acetylation repression, phosphorylation pathways and identified DNA motif targets (Gu et al. 2022; Liu et al. 2019b; Kazan and Gardiner 2018). However, there are still many TFs whose precise regulatory mechanisms and specific targets have yet to be fully characterised and elucidated.

Imitation switch (ISWI) proteins comprise a family of ATP‐dependent chromatin remodellers that are responsible for the regular spacing of nucleosomes on chromatin and that affect higher‐order chromatin structure and gene expression (Bartholomew 2014; Tan et al. 2020; Tao et al. 2023). ISWI proteins usually associate with one to three accessory subunits, forming multiple ISWI complexes that are functional in eukaryotes (Yadon and Tsukiyama 2011). Recent research has shown that the chromatin remodeller ISWI regulates the sleep of adult *Drosophila* during development (Gong et al. 2020). Further, increasing numbers of preclinical and clinical studies have highlighted that ISWI complexes are involved in tumourigenesis, tumour development, tumour immunity and drug responses. ISWI subunits exert multiple functions that affect tumour cell phenotypes by regulating the transcription of oncogenic genes (Li et al. 2021).

ISWI complexes frequently contain proteins with structurally conserved domains referred to as DDT (DNA‐binding homeobox and different transcription factor) domains (Dong et al. 2013). The ISWI subfamily remodeller was initially discovered in *Drosophila*. Two DDT‐domain proteins from *Drosophila*, ACF1 and NURF301 (Tsukiyama et al. 1995; Xiao et al. 2001), play a crucial role in three ISWI complexes: ACF, CHRAC and NURF (Ito et al. 1997, 1999; Varga‐Weisz et al. 1997; Eberharter et al. 2001; Tsukiyama and Wu 1995). Various other DDT‐domain proteins were subsequently identified as components of ISWI complexes in yeast, humans and *Arabidopsis* (Clapier and Cairns 2009; Narlikar et al. 2013; Li et al. 2017). The ISWI complex of yeast contains a DDT‐domain, Itc1 and ISW2 proteins (Gelbart et al. 2001; Sugiyama and Nikawa 2001). The DDT‐domain proteins of humans, TIP5 and WSTF, are also structurally essential for ISWI complexes (Bozhenok et al. 2002; Strohner et al. 2001). In addition, the *Arabidopsis* DDT‐domain proteins RLT1 and RLT2 serve as ISWI subunits and play crucial roles in meristem fate transition, floral organ formation, flowering time and fertility (Li et al. 2017). These observations indicate the formation of relatively stable complexes between DDT‐domain proteins and ISWI across eukaryotes; however, whether these mechanisms are conserved in non‐model species and whether the complex is also important during fungal infection of plants is poorly understood. In addition, the transcriptional regulatory mechanisms mediated by DDT‐domain proteins in fungal species require further elucidation.

In this study, novel regulatory targets of the transcription factor FgDDT were identified in *F. graminearum* through chromatin immunoprecipitation‐sequencing (ChIP‐seq). ChIP‐seq analysis demonstrated the enrichment of FgDDT in the promoter regions of genes associated with metabolic and mitogen‐activated protein kinase (MAPK) signalling pathways, leading to the activation of their expression. Additionally, two other ISWI genes, FgISW1 and FgISW2, were identified and characterised, with FgISW1 specifically implicated in pathogenicity. Moreover, FgDDT was found to physically interact with FgISW1 proteins and regulate key genes essential for hyphal growth and pathogenicity of *F. graminearum*. These findings demonstrate that the ISWI complex, in addition to core metabolic and developmental roles, is also a prerequisite for pathogenicity development in *F. graminearum*, thereby expanding our understanding in a damaging plant pathogen.

## 2 Results

### 2.1 Identification of FgDDT in *F. graminearum*

**FIGURE 1:**
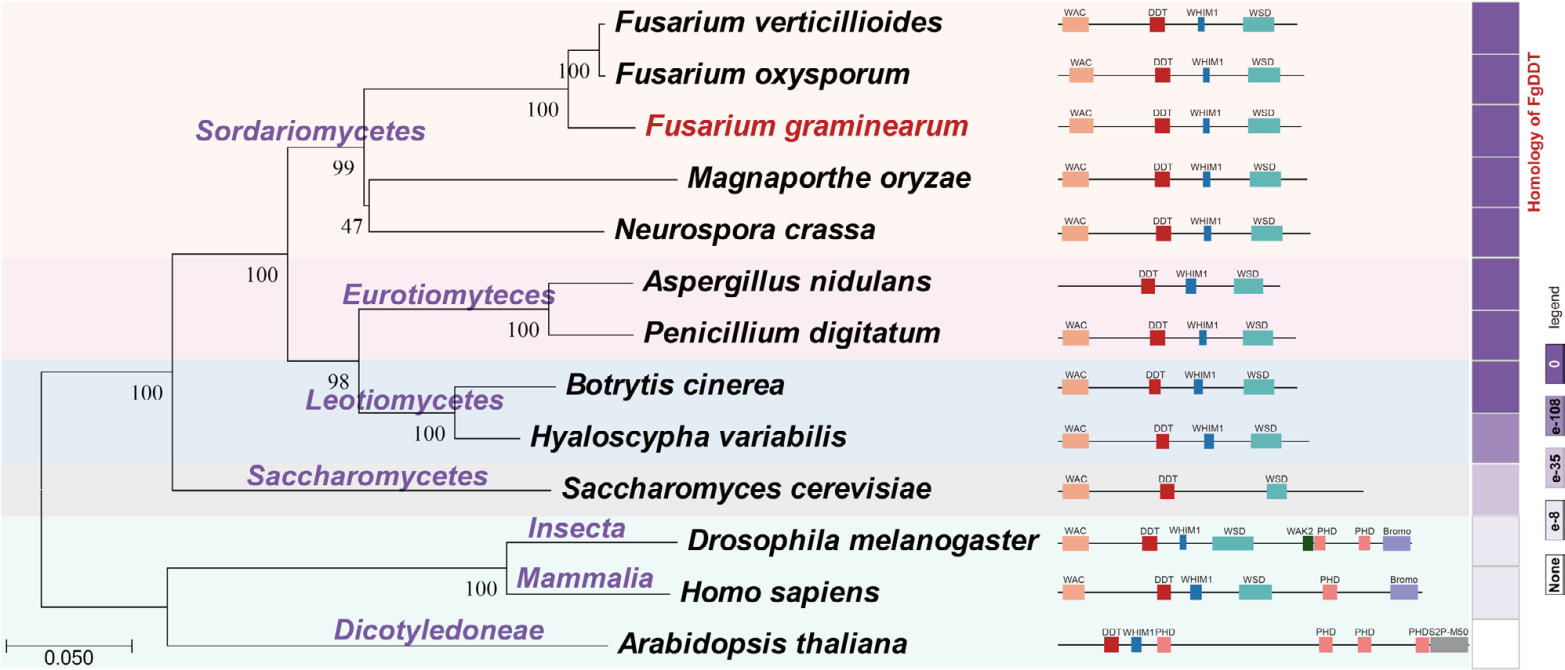
DDT is highly conserved in the *Fusarium* genus. The phylogenetic tree was created using RPB2 proteins sequences from 13 eukaryotes (left). Bootstrap values (out of 1000 replicates) are indicated on branches. Conserved domains in DNA‐binding homeobox and different transcription factor (DDT) protein orthologues were predicted using the Smart program (middle). The purple colours indicate the level of homology with the DDT protein (right).

To investigate the function of DDT in *F. graminearum*, a BLASTp search of the *F. graminearum* genome database (http://fungi.ensembl.org/Fusarium_graminearum/Info/Index) was conducted using genes from *Saccharomyces cerevisiae* ITC1. Only one DDT‐domain protein, FgDDT (Fg06065), was identified in *F. graminearum*. Sequence alignment revealed that the DDT is highly conserved in *Fusarium* but exhibits low similarity to those in *Homo sapiens* and *Arabidopsis thaliana* (Figure 1), suggesting potential differences in biological functions. The WAC, WHIM1 and WSD domains of the DDT protein are present in most eukaryotes, whereas the PHD domain is only found in *H. sapiens* , *Drosophila melanogaster* and *A. thaliana*, further suggesting functional diversity.

### 2.2 FgDDT Regulates Fungal Development and Pathogenicity of *F. graminearum*

**FIGURE 2:**
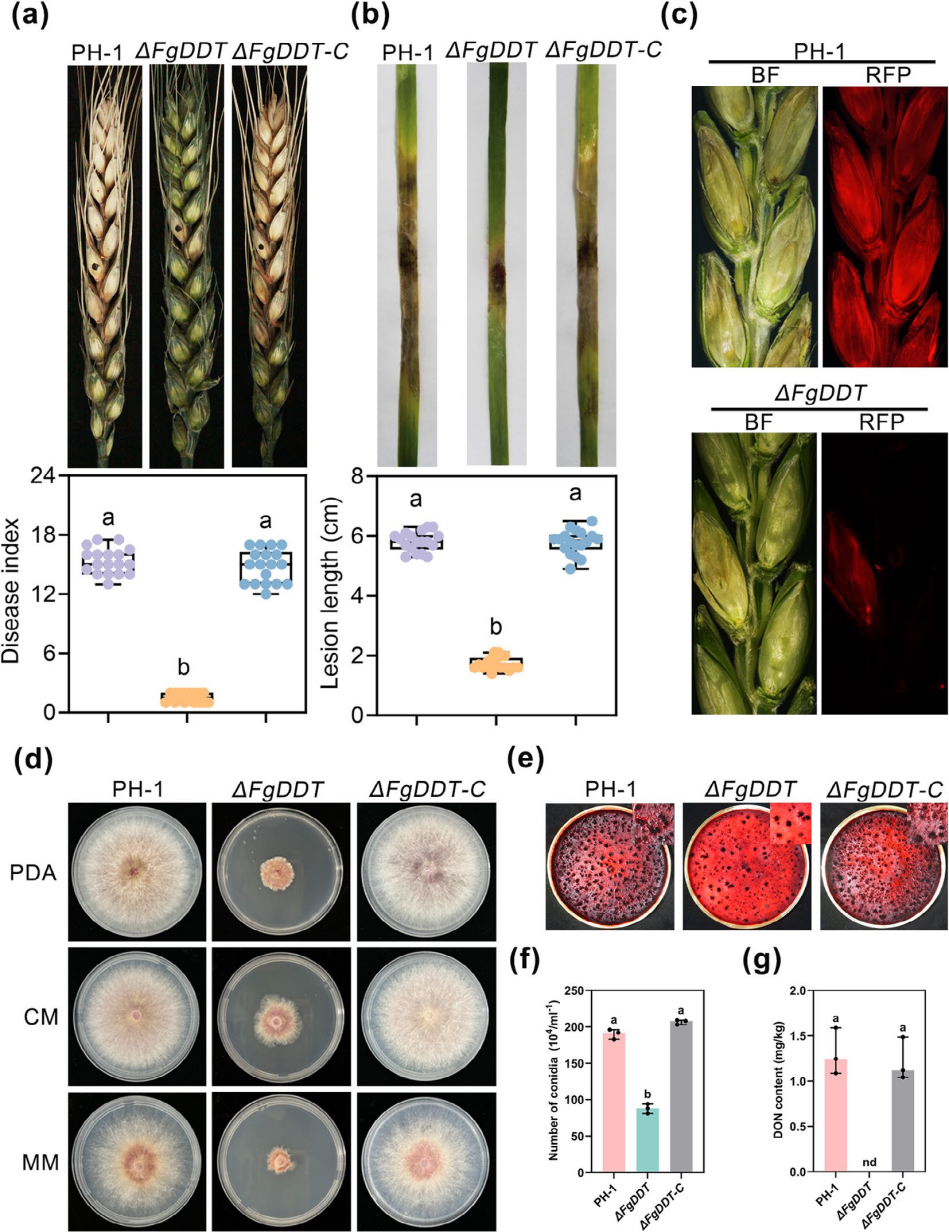
FgDDT is required for pathogenicity and development in *Fusarium graminearum*. (a) Pathogenicity assays of the PH‐1 (wild‐type, WT), Δ*FgDDT* and complementary strain Δ*FgDDT‐*C on flowering wheat heads. Representative pictures were taken at 14 days post‐inoculation (dpi), and the disease index was measured. (b) Pathogenicity of PH‐1 and the other indicated strains on wheat seedling leaves. Representative photographs were taken at 5 dpi, and the leaf lesion lengths were measured. The data represent mean ± SD (*n* = 20). Median, interquartile ranges, minimum and maximum values are shown for the box plots. Different letters indicate statistically significant differences (*p* < 0.05, one‐way ANOVA). (c) Cross sections of inoculated and adjacent wheat spikelets. Spikelets were inoculated with the indicated strains containing FgACTIN‐RFP. Representative photographs were taken at 7 dpi. BF, bright field; RFP, red fluorescent protein. (d) PH‐1 and its variants after cultivation on potato dextrose agar (PDA), complete medium (CM), minimal medium (MM) for 3 days at 25°C. (e) Evaluation of perithecium formation in mating cultures of PH‐1 and the indicated strains. (f) Conidiation of PH‐1 and the indicated strains was measured by counting the number of conidia produced in carboxymethyl cellulose (CMC) cultures after 4 days. Data are presented as mean ± SD from three repeated experiments. Different letters indicate a significant difference (*p* < 0.05, one‐way ANOVA). (g) Deoxynivalenol (DON) production in the mutants and WT growing in trichothecene biosynthesis‐inducing (TBI) medium. Line bar in each column denotes SD of three repeated experiments. nd, no detection.

To further investigate the functions of FgDDT, *FgDDT* was deleted using homologous recombination, and the knockout mutant, Δ*FgDDT*, was confirmed by PCR and Southern blot assays (Figure S1). The pathogenicity of the wild‐type (WT) PH‐1, the mutant Δ*FgDDT* and the complementary strain Δ*FgDDT‐C* was subsequently evaluated on flowering wheat heads. The disease index of Δ*FgDDT* on wheat spikelets was significantly decreased compared to that of the WT PH‐1 and complementary strain Δ*FgDDT‐*C (Figure 2a). To further analyse pathogenicity defects, a red fluorescent protein (RFP) gene driven by the promoter of the *F. graminearum ACTIN* gene was introduced into Δ*FgDDT* and the WT PH‐1. The resulting strains were used to inoculate flowering wheat heads. Epifluorescence stereoscope analysis revealed diminished RFP signals in Δ*FgDDT* that were confined to the inoculated spikelets at 7 days post‐inoculation (dpi), whereas PH‐1 exhibited RFP signals in both the inoculated and nearby spikelets (Figure 2c). Concurrently, the lesion areas on wheat seedling leaves caused by Δ*FgDDT* were significantly reduced (Figure 2b). Taken together, these results show that FgDDT contributes to *F. graminearum* pathogenicity in host plants.

The requirement of FgDDT for *F. graminearum* growth was subsequently investigated, revealing hyphal growth defects in the Δ*FgDDT* mutant when cultured on potato dextrose agar (PDA), minimal medium (MM), or complete medium (CM). The radial growth of the Δ*FgDDT* mutant was significantly reduced on PDA, CM and MM plates compared to that of the WT strain PH‐1 and the complemented strain Δ*FgDDT‐*C (Figure 2d). Ascospores and conidiophores play crucial roles in FHB epidemics (Trail 2009; Cavinder et al. 2012). Therefore, we investigated the sexual reproduction and conidial production of PH‐1, Δ*FgDDT* and the complemented strain Δ*FgDDT‐*C. The results revealed significant decreases in sexual reproduction for the Δ*FgDDT* mutant compared to the wild‐type strain PH‐1 and the complemented strain Δ*FgDDT‐*C (Figure 2e). Additionally, the number of conidia produced by the Δ*FgDDT* mutant was significantly reduced (Figure 2f). Collectively, these findings indicate that FgDDT is involved in vegetative growth, asexual reproduction and sexual development in *F. graminearum*.

Deoxynivalenol (DON) toxin plays a critical role in the spread of the pathogen to the neighbouring spikelets (Seong et al. 2009). Therefore, we assayed the levels of DON toxin after culturing wild‐type PH‐1, mutant Δ*FgDDT* and the complemented strain Δ*FgDDT‐*C in trichothecene biosynthesis‐inducing (TBI) medium. Our results indicate that the FgDDT mutant produced undetectable amounts of DON, whereas both the wild‐type and complemented strains produced similar levels of DON (Figure 2g). This observation underscores the critical role of FgDDT in facilitating the production of toxins by *F. graminearum*.

### 2.3 FgDDT Is Involved in the Regulation of Genes Associated With Metabolic and MAPK Signalling Pathways

**FIGURE 3:**
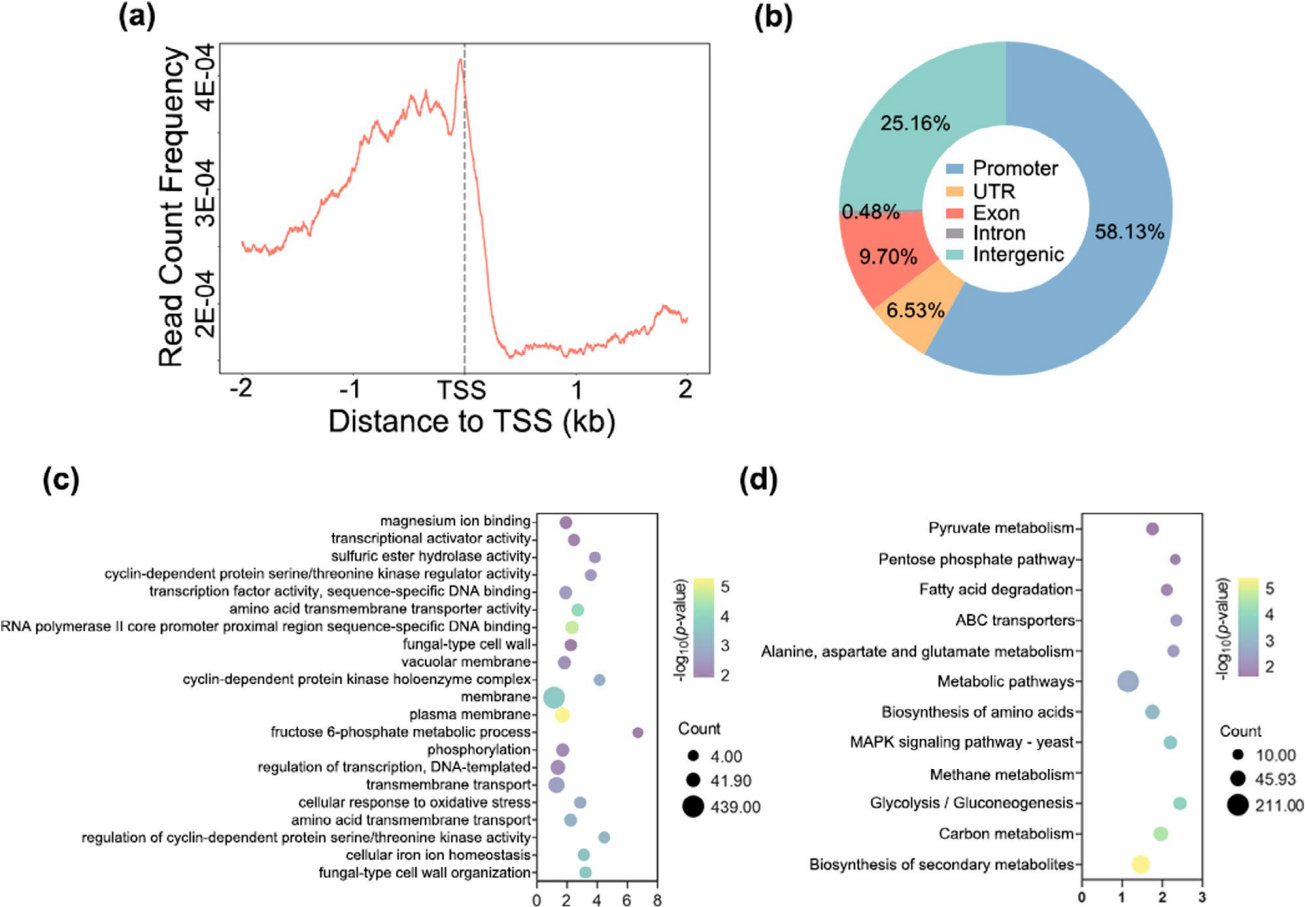
Genome‐wide identification of target genes bound by FgDDT. (a) FgDDT‐binding peaks highly enriched near the gene promoter. (b) Distributions of the overlapping FgDDT‐binding peaks in promoter, untranslated region (UTR), intron, exon and intergenic regions from two biological replicates. (c) Twenty‐one most‐enriched Gene Ontology (GO) categories within the larger biological processes category in FgDDT‐targeted genes. (d) Twelve most‐enriched KEGG pathways among FgDDT‐targeted genes.

To further evaluate the function of FgDDT in regulating pathogenicity and growth in *F. graminearum*, genome‐wide ChIP‐seq analysis was conducted for FgDDT to profile the chromatin landscape. In two biological replicates, we identified 4393 peaks as FgDDT‐binding sites. Notable enrichment of FgDDT‐binding sites was observed in gene promoters (Figure 3a), with 58.13%, 6.53%, 9.7%, 0.48% and 25.16% localised in gene promoters (1 kb upstream of open reading frames [ORFs]), untranslated regions (UTRs), intergenic regions, exons and introns, respectively (Figure 3b). Genome‐wide annotation revealed that these peaks correspond to 2429 genes (Table S1). Gene Ontology (GO) and Kyoto Encyclopedia of Genes and Genomes (KEGG) enrichment analyses of the FgDDT‐bound genes revealed their involvement in various functional categories, including cellular components, protein serine/threonine kinase activity and cellular responses to oxidative stress (Figure 3c). KEGG enrichment analysis further revealed significant enrichment of metabolic pathways, biosynthesis of secondary metabolites, carbon metabolism, biosynthesis of amino acids and MAPK signalling pathway among the FgDDT‐bound genes (Figure 3d).

**FIGURE 4:**
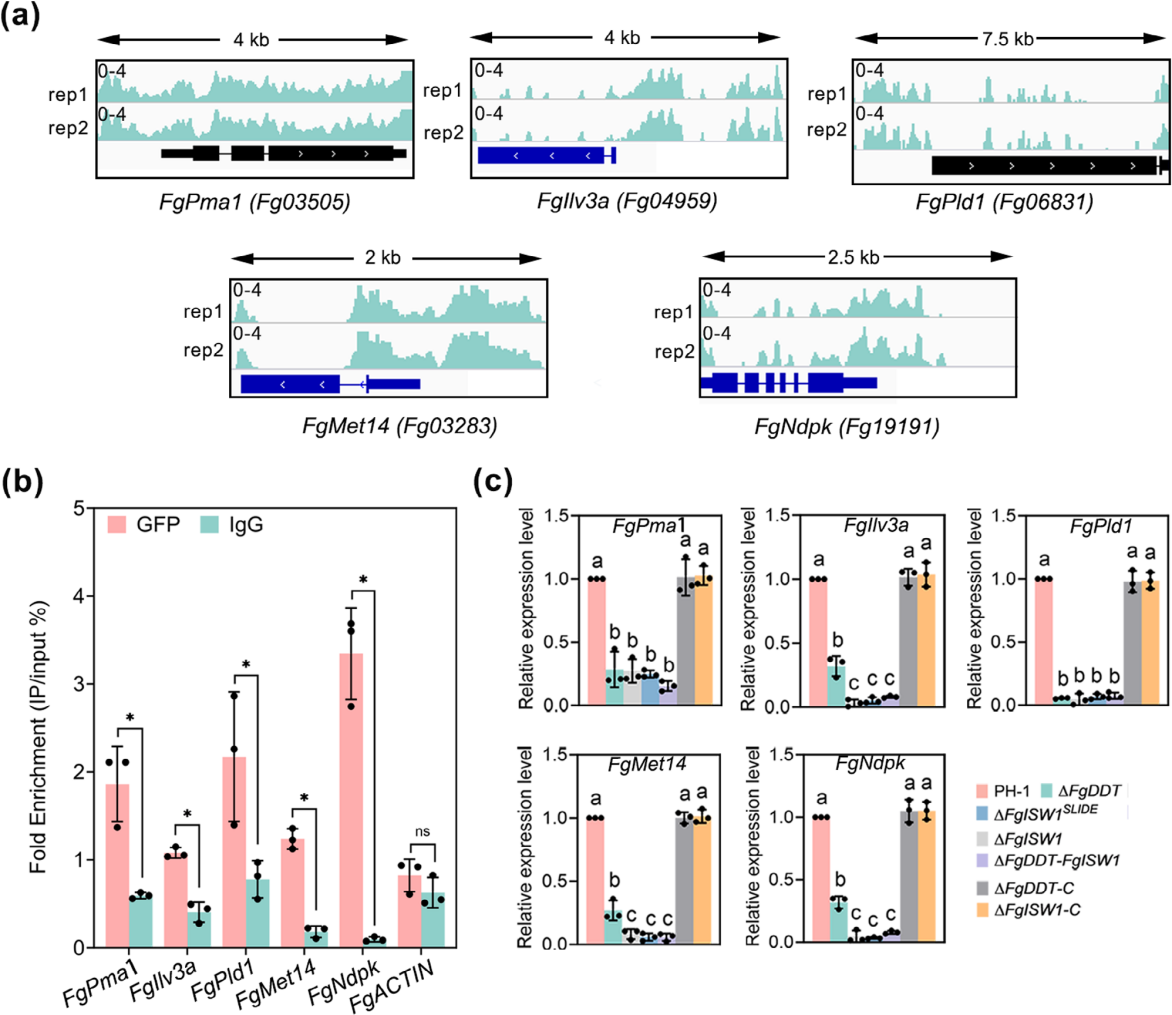
FgDDT is involved in the regulation of metabolic pathway genes. (a) Representative examples of genes enriched in the FgDDT‐GFP signal based on observation via the Integrative Genomics Viewer platform. ChIP signals were calculated as log_2_(IP_RPKM_/Input_RPKM_). Blue or black boxes with white arrows indicate the open reading frames of genes. (b) Chromatin immunoprecipitation (ChIP)‐quantitative PCR measurements of FgDDT‐GFP deposition for the indicated genes. Relative enrichment levels are shown as percentages of input. IgG represents the negative control. Significance was measured using an unpaired *t* test (ns: not significant, *p < 0.05). (c) Relative expression level of the indicated genes in the wild‐type PH‐1, Δ*FgDDT*, Δ*FgISW1*, Δ*FgISW1* ^*SLIDE*^, Δ*FgDDT‐FgISW1* and complementary strains (Δ*FgDDT‐*C and Δ*FgISW1‐*C). Data are presented as mean ± SD from three repeated experiments in (b) and (c). Different letters indicate a significant difference (*p* < 0.05, one‐way ANOVA).

The metabolic and MAPK signalling pathways, which are known to play crucial roles in regulating fungal development, growth and pathogenicity (Gu et al. 2015; Jiang et al. 2018; Ene et al. 2014), were also found to be targeted by FgDDT. Among these FgDDT‐targeted genes, we selected those with known pathogenic roles and enrichment in metabolic pathway categories for further target regulation analysis: *FgPma1*, which is involved in plasma membrane homeostasis and pathogenicity (Wu et al. 2022); *FgIlv3a*, a critical component of amino acid metabolism and virulence (Liu et al. 2019a); *FgPld1*, implicated in phospholipid metabolism and fungal infection (Ding et al. 2017); *FgMet14*, essential for methionine metabolism and linked to pathogenicity (Zhao et al. 2022); and *FgNdpk*, a nucleoside diphosphate kinase associated with fungal virulence (Mao et al. 2024). Notably, these five genes exhibited relatively high enrichments compared to the input control in the genome browser (Figure 4a). ChIP‐qPCR confirmed that FgDDT‐GFP was effectively enriched at the promoter regions of these genes (Figure 4b). The reverse transcription‐quantitative PCR (RT‐qPCR) assays subsequently revealed significantly lower transcriptional levels in the Δ*FgDDT* mutant compared to those of the wild type (WT) (Figure 4c).

**FIGURE 5:**
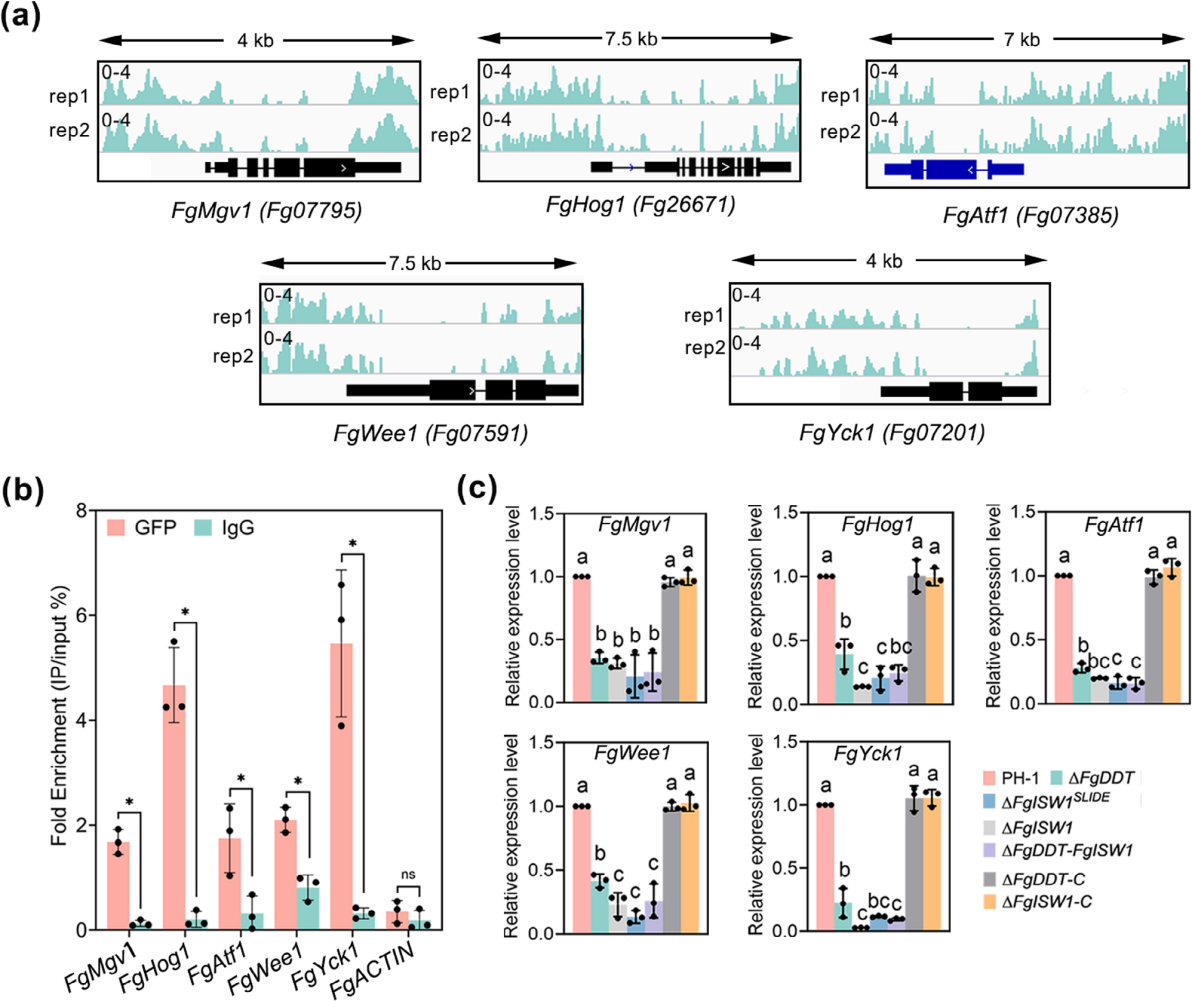
FgDDT is involved in the regulation of MAPK signalling pathway genes. (a) Representative examples of genes enriched in the FgDDT‐GFP signal visualised with the Integrative Genomics Viewer platform. ChIP signals were calculated as log_2_(IP_RPKM_/Input_RPKM_). Blue or black boxes with white arrows represent the open reading frames of genes. (b) Chromatin immunoprecipitatin (ChIP)‐quantitative PCR measurements of FgDDT‐GFP deposition of indicated genes. Relative enrichment levels are indicated as percentages of input. IgG represents the negative control. Significance was measured using an unpaired *t* test (ns: not significant, *p < 0.05). (c) Relative expression level of the indicated genes in the wild‐type PH‐1, Δ*FgDDT*, Δ*FgISW1*, Δ*FgISW1* ^*SLIDE*^, Δ*FgDDT‐FgISW1* and complementary strains (Δ*FgDDT‐*C and Δ*FgISW1‐*C). Data are presented as mean ± SD from three triplicate experiments in (b) and (c). Different letters indicate a significant difference (*p* < 0.05, one‐way ANOVA).

We then focused our investigation on whether FgDDT regulates a set of MAPK genes known to be critically involved in pathogenicity: *FgMgv1*, which plays a pivotal role in maintaining cell wall integrity and thus pathogenesis (Hou et al. 2002); *FgHog1*, a central mediator of the osmotic stress response that is also linked to virulence (Zheng et al. 2012); *FgAtf1*, integral to the oxidative stress signalling pathways that contribute to disease development (Jiang et al. 2015); *FgWee1*, a crucial regulator of cell cycle progression that impacts fungal virulence (Wang et al. 2011); and Fg*Yck1*, recently identified as a key player in nutrient sensing and signalling processes related to pathogenicity (Zhu et al. 2022). Notably, we observed relatively high enrichments of these selected MAPK genes compared with the input control in the genome browser (Figure 5a). Moreover, ChIP‐qPCR and RT‐qPCR assays confirmed the downregulation of selected genes, further indicating a role of FgDDT in regulating the MAPK signalling pathway in *F. graminearum* (Figure 5b,c). We also tested the ability of recombinant FgDDT to bind to the targeted genes *FgHog1* and *FgNdpk* promoters using an electrophoretic mobility shift assay (EMSA; Figure S2), the results demonstrating that FgDDT can directly bind to the *FgHog1* and *FgNdpk* promoters. Taken together, these results indicated that the deletion of FgDDT affects the expression of genes associated with metabolic and MAPK signalling pathways, providing a plausible explanation for the perturbed phenotypes observed in the gene‐deletion mutant.

### 2.4 Transcription Factor FgDDT Interacts With ISWI to Regulate the Development and Pathogenicity of *F. graminearum*

**FIGURE 6:**
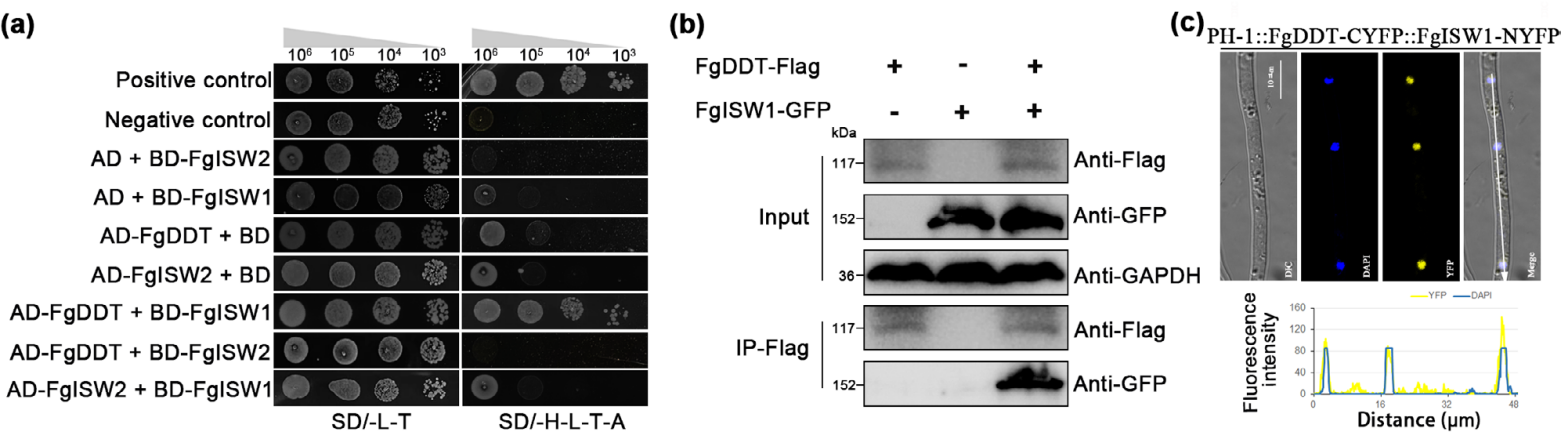
FgDDT interacts with the FgISW1 in *Fusarium graminearum*. (a) Determination of the interactions among FgDDT, FgISW1 and FgISW2 in yeast two‐hyrbid assays. SD/−L−T indicates medium lacking leucine and tryptophan. SD/−H−L−T−A indicates medium lacking histidine, leucine, tryptophan adenine. A yeast strain containing pGBKT7‐53 and pGADT7 was used as the positive control, while a strain containing pGBKT7‐Lam and pGADT7 was used as the negative control. (b) FgDDT interaction with FgISW1 in co‐immunoprecipitation (Co‐IP) assays. Protein extracts were immunoprecipitated with anti‐FLAG magnetic beads and detected using anti‐GFP and anti‐FLAG antibodies. GAPDH was used as the loading control. (c) Bimolecular fluorescence complementation (BiFC) assays for the interaction of FgDDT with FgISW1. The constructs were stained with 4,6‐diamidino‐2‐phenylindole (DAPI) and examined by epifluorescence microscopy (YFP, yellow fluorescent protein). Bar, 10 μm (upper panel). White arrows highlight areas analysed by line‐scan graph analysis. *y* axis: The intensity of YFP and DAPI signals quantified by ImageJ; *x* axis: The distance (μm) (lower panel).

ISWI proteins are conserved in eukaryotes and usually form complexes with DDT‐domain proteins (Dong et al. 2013). Here, two ISWI proteins were identified in *F. graminearum*: FgISW1 and FgISW2 by the BLASTp search of the *F. graminearum* genome database using genes from *S. cerevisiae* ISW1 and ISW2. Sequence alignment revealed that FgISW1 shares 45% similarity with FgISW2 (Figure S3). To further elucidate the mechanism by which FgDDT regulates fungal pathogenicity with chaperone proteins in *F. graminearum*, yeast two‐hybrid assays were conducted to explore whether FgDDT interacts with FgISW1 and FgISW2. FgDDT only interacted with FgISW1, but not with the other subunit of the ISWI complex, FgISW2, and no direct interaction was observed between FgISW1 and FgISW2 (Figure 6a). Co‐immunoprecipitation (Co‐IP) assays further confirmed the presence of interactions between FgDDT and FgISW1 (Figure 6b). In addition, the bimolecular fluorescence complementation (BiFC) assays showed that FgDDT could interact with FgISW1 in the nucleus (Figure 6c). These results indicate that the transcription factor FgDDT is associated with the ISWI complex via direct interaction with the FgIWS1 subunit in *F. graminearum*.

**FIGURE 7:**
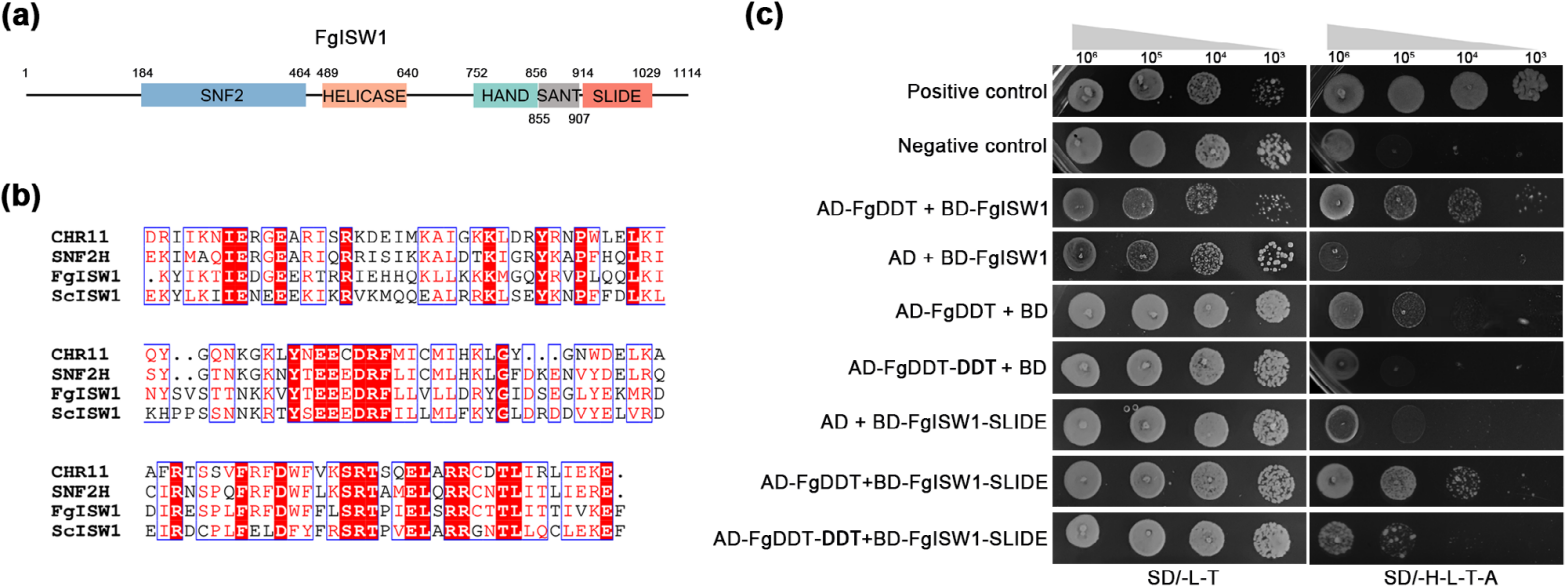
FgISW1 interacts with FgDDT via the SLIDE domain. (a) Domain architecture of *Fusarium graminearum* ISW1. (b) Multiple sequence alignment of the SLIDE domain of ISW1 protein sequences. ScISW1, *Saccharomyces cerevisiae* ISW1 protein; CHR11, *Arabidopsis thaliana* ISW1 protein; SNF2H, human ISW1 protein. (c) Determination of interaction between the SLIDE domain of FgISW1 and FgDDT protein in yeast two‐hybrid assays. SD/−L−T indicates medium lacking leucine and tryptophan; SD/−H−L−T−A indicates medium lacking histidine, leucine, tryptophan and adenine. Yeast strains containing pGBKT7‐53 and pGADT7 were used as the positive control, while those containing pGBKT7‐Lam and pGADT7 were used as negative controls.

Sequence analysis of FgISW1 using the Pfam database revealed the presence of typical domains, including SNF, HELICASE, HHAND, SANT and SLIDE (Figure 7a). The SLIDE domain of ISW1 in *Arabidopsis thaliana* and humans is known to interact with DDT‐domain proteins (Dong et al. 2013), and in *S. cerevisiae* , the SLIDE domain is required for histone interactions (Pinskaya et al. 2009). Sequence alignment of SLIDE from *A. thaliana* , humans, *S. cerevisiae* and *F. graminearum* revealed a high degree of amino acid sequence similarity between the SLIDE domain of FgISW1 and those of *S. cerevisiae* , *A. thaliana* and humans (47%, 43% and 50%, respectively) (Figure 7b). Subsequent evaluation of the interactions between the SLIDE domain of FgISW1 and the FgDDT protein using yeast two‐hybrid (Y2H) assays yielded results consistent with those observed in *A. thaliana* , indicating that the SLIDE domain plays a crucial role in interacting with DDT‐domain proteins. In contrast, there was no interaction between the DDT and SLIDE domains (Figure 7c), suggesting the DDT domain facilitates interactions between FgISW1 and FgDDT, but it may not be sufficient.

### 2.5 ISWI Component FgISW1, Rather Than FgISW2, Is Involved in the Pathogenicity of *F. graminearum*

**FIGURE 8:**
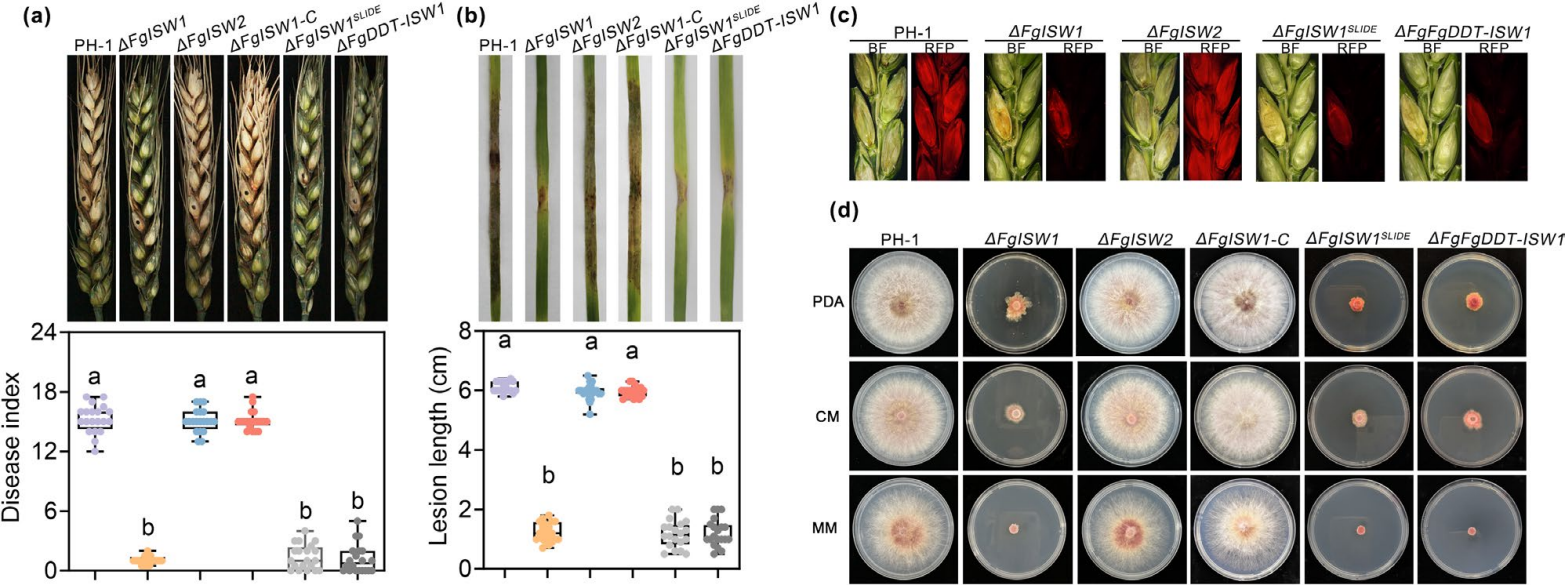
FgDDT associates with FgISW1 to regulate transcription of metabolic and MAPK signalling pathways genes. (a) Pathogenicity assays of the PH‐1 (wild‐type), Δ*FgISW1*, Δ*FgISW2*, Δ*FgISW1* ^*SLIDE*^, Δ*FgDDT‐FgISW1* and complementary strain (Δ*FgISW1*‐C) on flowering wheat heads. Representative pictures were taken at 14 days after inoculation and the disease index was measured. (b) Wheat seedling leaves were inoculated with mycelial plugs and photographs were taken at 5 days after inoculation. The leaf lesion lengths were measured and the data represent the mean ± SD (*n* = 20). Median, interquartile ranges, minimum and maximum values are shown for the box plots. Different letters indicate statistically significant differences (*p* < 0.05, one‐way ANOVA). (c) Cross sections of inoculated and adjacent wheat spikelets. Spikelets were inoculated with the indicated strains containing FgACTIN‐RFP. Representative photographs were taken at 7 days after inoculation. BF, bright field; RFP, red fluorescent protein. (d) PH‐1 and its variants after cultivation on potato dextrose agar (PDA), complete medium (CM), minimal medium (MM) for 3 days at 25°C.

To explore the possible contribution of ISWI complexes in the pathogenicity of *F. graminearum*, independent deletion mutants of FgISW1 and FgISW2 were generated. Pathogenicity assays revealed that the Δ*FgISW1* mutant caused less severe symptoms on flowering wheat heads and seedling leaves compared with the WT PH‐1 strain. However, lesions caused by the Δ*FgISW2* mutant on flowering wheat heads and seedling leaves were similar to those caused by PH‐1 (Figure 8a–c). In addition, the growth rate and colony morphology of the FgISW2‐deletion mutant (Δ*FgISW2*) were similar to those of PH‐1, whereas the Δ*FgISW1* mutant exhibited hyphal growth defects (Figure 8d). Overall, these results highlight the crucial involvement of the ISWI component FgISW1 in the pathogenicity of *F. graminearum*, whereas FgISW2 exhibits a negligible contribution. Analogous to FgDDT, FgISW1 regulates both hyphal growth and pathogenicity in *F. graminearum*. When viewed collectively, these findings align seamlessly with the interactions between the aforementioned ISWI complexes. Taken together, these results suggest that the subunits of the ISWI complex exhibit different functions in *F. graminearum*.

To explore the possible contribution of the SLIDE domain to the pathogenicity and growth function of FgISW1, we generated a deletion mutant lacking the SLIDE domain of FgISW1. Pathogenicity and growth assays showed that the SLIDE domain of FgISW1 is involved in the vegetative growth and pathogenicity of *F. graminearum* (Figure 8a–d). Notably, the phenotypic defects in pathogenicity and growth caused by the deletion of the SLIDE domain were comparable to those observed in the complete deletion of FgISW1, aligning with the interactions between the SLIDE domain and DDT‐domain proteins. These findings further underscore the importance of the SLIDE domain within FgISW1.

Next, we also generated the double‐deletion mutants of FgDDT and FgISW1 (Δ*FgDDT‐FgISW1*). Remarkably, the Δ*FgDDT‐FgISW1* mutant exhibited no additional attenuation in pathogenicity or growth compared to the Δ*FgISW1* single mutant (Figure 8a–d). These findings indicate that FgDDT and FgISW1 have no additive effects on the pathogenicity of *F. graminearum*.

### 2.6 FgDDT Associates With FgISW1 to Regulate Transcription of Genes Involved in Metabolic and MAPK Signalling Pathways

Given that FgISW1 interacts with FgDDT and is essential for the pathogenicity of *F. graminearum*, the regulation of metabolic and MAPK signalling pathways gene transcription by FgISW1 was evaluated. The transcription levels of five metabolic pathway genes, *FgPma1*, *FgIlv3a*, *FgPld1*, *FgMet14* and *FgNdpk*, were significantly reduced in the Δ*FgISW1* mutant (Figure 4c). Concomitantly, the expression levels of five MAPK genes, *FgMgv1, FgHog1, FgAtf1, FgWee1* and *FgYck1*, were also lower in Δ*FgISW1* compared to the WT (Figure 5c). These results suggest that FgISW1 coordinates the activation of functional gene expression. This observation is consistent with the regulation by FgDDT. Further analysis revealed that the majority of the 10 functional genes exhibited a more pronounced decrease in expression levels in Δ*FgISW1* compared to Δ*FgDDT* (Figures 4c and 5c), suggesting the important role of FgISW1 within the ISWI complex in modulating the expression profiles of the identified functional genes.

Subsequently, we examined the expression levels of 10 target functional genes in the Δ*FgISW1* ^*SLIDE*^ mutant and Δ*FgDDT‐FgISW1* double mutant. Our results revealed that, compared to the WT strain, the expression levels of these target genes were significantly decreased in both the Δ*FgISW1* ^*SLIDE*^ mutant and the Δ*FgDDT‐FgISW1* double mutant (Figures 4c and 5c). Notably, in the Δ*FgDDT‐FgISW1* double mutant, the extent of downregulation for these target genes was comparable to that observed in the Δ*FgISW1* single mutant. This observation aligns with our previous results, where no discernible difference was found in the pathogenicity between the double mutant and the Δ*FgISW1* single mutant. Collectively, these results suggest that FgDDT synergistically regulates the expression of metabolic and MAPK signalling pathways genes with FgISW1, thereby contributing to the pathogenicity of *F. graminearum*.

To gain deeper insights into the intricate interplay between FgDDT and FgISW1, we conducted a comprehensive analysis of their transcriptional levels and protein abundances in the mutant backgrounds. Our findings revealed a reciprocal influence between FgDDT and FgISW1 at both the transcriptional and protein expression levels (Figure S4a,b). Fluorescence localisation assays revealed that, independent of the deletion of either gene, the subcellular localisation of the remaining protein remained unchanged, yet the nuclear localisation intensity was significantly attenuated upon knockout of the partner gene (Figure S4c). These observations underscore a mutualistic relationship between FgDDT and FgISW1, where they intricately interact and cooperate to regulate the expression of functional genes.

## 3 Discussion

Previous studies have shown that ISWI complexes are involved in several developmental processes in organisms (Li et al. 2012; Smaczniak et al. 2012). For instance, *Arabidopsis* encodes two ISWI members, CHR11 and CHR17, that are required for the even spacing of nucleosomes in gene bodies (Li et al. 2014). The loss of function of CHR11/17 results in pleiotropic developmental defects and early flowering (Li et al. 2012; Smaczniak et al. 2012). Furthermore, the ISWI subunits of humans exhibit multiple functions that affect tumour cell phenotypes by modulating the transcription of oncogenic genes (Li et al. 2021). In fungi, while maintaining a core role in chromatin remodelling, ISWI subunits manifest distinct functions tailored to species‐specific needs. ISW1 and ISW2 of yeast play important roles in coordinating the repression, activation and elongation phases of transcription (Yadon and Tsukiyama 2011; Mellor and Morillon 2004). In contrast, the TrISW1 of *Trichoderma reesei* is an important chromatin remodeller with a secondary role in coordinating the cellulolytic response and biosynthesis of major secondary metabolites (Cao et al. 2022). This functional expansion highlights the adaptability of ISWI subunits to specific ecological niches. *Neurospora* ISW1 complexes are required for normal patterns of H3K27 and H3K36 methylation in the *Neurospora* genome, and their absence makes strains hypersensitive to genotoxic stress (Kamei et al. 2021). ISW1 of *Cryptococcus neoformans* plays a critical role in modulating gene expression responsible for multidrug resistance (Meng et al. 2024). *Candida albicans* ISW2 regulates chlamydospore suspensor cell formation and virulence in vivo in a mouse model of disseminated candidiasis (Navarathna et al. 2016). These findings underscore the unique roles of ISWI complexes in different fungal pathogens, particularly in adapting to host environments and virulence mechanisms. In this study, three ISWI protein components in *F. graminearum* were identified: FgDDT, FgISW1 and FgISW2. FgDDT and FgISW1 were determined to play critical roles in fungal development and pathogenicity, whereas FgISW2 did not exhibit a direct involvement in these processes, suggesting that it may have distinct or more specialised functions within the ISWI complex. The subunits of the ISWI complex were determined to exhibit these differential functions, highlighting the complexity and specificity of chromatin remodelling in this pathogenic fungus. These insights not only deepen our understanding of ISWI biology but also provide a foundation for future investigations into the specific functional roles of ISWI complexes in diverse eukaryotic lineages.

ISWI proteins typically form complexes with different DDT‐domain proteins, resulting in diverse functions (Yadon and Tsukiyama 2011; Doerks et al. 2001; Kingston and Narlikar 1999). Indeed, protein interactions between ISWI remodellers and DDT‐domain proteins have been identified across various eukaryotic species. For example, in *A. thaliana* , as a result of complex formation between ISWI proteins CHR11 and CHR17 and the DDT‐containing RLT1 and RLT2 genes, multiple developmental processes are affected, including meristem fate transition and organ formation (Li et al. 2017, 2012). In *S. cerevisiae* , two ISWI catalytic subunit variants (ISW1 and ISW2) form four different complexes via association with different subunits. In conjunction with DDT‐domain protein Itc1, ISW2 regulates nucleosome spacing and subsequently affects nucleosome remodelling (Hada et al. 2019). The DDT‐domain proteins of humans, TIP5 and WSTF, perform specialised functions in cancers as components of the ISWI complexes (Bozhenok et al. 2002; Strohner et al. 2001; Pietrzak et al. 2020). Moreover, at least 12 DDT‐domain proteins have been identified in *Arabidopsis* that contribute to diverse biological functions (Li et al. 2017). Here, only one DDT‐domain protein, FgDDT, was identified in *F. graminearum*, and it was observed to exclusively interact with FgISW1, the catalytic subunit of the ISWI complex, but not with the other subunit FgISW2. This observation aligns with previous studies in other species, highlighting the conserved nature of interactions between ISWI remodellers and DDT‐domain proteins. However, our findings underscore the specificity of these interactions, as not all ISWI subunits in *F. graminearum* directly engage with DDT proteins. This underscores the complexity and diversity of protein–protein interactions within the ISWI chromatin remodelling machinery. Future studies should investigate the biological function of ISW2 and determine how different types of ISWI complexes cooperatively function to regulate chromatin states and gene expression during diverse biological processes. Moreover, the SLIDE domain of FgISW1 is responsible for interaction with DDT‐domain proteins, whereas specific DDT‐ISWI functions may rely on distinct components of DDT‐domain proteins. The identity of the functional domain of the ISWI complex is a key outstanding question, in addition to how they participate in gene transcription.

TFs are a class of sequence‐specific DNA‐binding proteins that play important roles in regulating gene expression (Son et al. 2011). TFs regulate the rate of expression for genes involved in various biological processes under different conditions and play important roles in fungal pathogenesis. In particular, organisms rely on a suite of operating TFs to orchestrate the expression of genes involved in phytopathogenicity (John et al. 2021). Several fungal TFs implicated in the regulation of virulence gene expression have been identified in plant pathogens. For instance, the bZIP transcription factor VdAtf1 regulates virulence by mediating nitrogen metabolism in *Verticillium dahliae* (Tang et al. 2020). In addition, the unfolded protein response (UPR) pathway transcription activator MoHac1 of *Magnaporthe oryzae* is alternatively spliced to regulate the expression of several putative target genes involved in endoplasmic reticulum (ER) stress responses (Tang et al. 2015). Likewise, the transcription factor FgSR of *F. graminearum* controls virulence primarily by modulating DON biosynthesis and responses to phytoalexin (Liu et al. 2019b). The transcription factor FgPacC is able to enhance adaptation of *F. graminearum* to cope with host‐derived high iron environments during infection by directly binding and inhibiting the HAT activity of FgGcn5 (Gu et al. 2022). In addition, the transcription factor FgStuA of *F. graminearum* regulates virulence and mycotoxin biosynthesis by recruiting the SAGA complex (Xu et al. 2023). Although previous studies have identified the association of certain TFs with pathogenicity, the mechanism related to the transcription factor FgDDT has not been reported in pathogenic fungi. In the present study, FgDDT was shown to be enriched in the promoter region of metabolic and MAPK signalling pathway genes, leading to regulation of the expression of these genes. FgDDT also interacted with the ISWI subunit FgISW1 to synergistically regulate these genes, leading to development and pathogenicity defects in *F. graminearum*. FgDDT and FgISW1 have a mutual influence on each other's expression levels. Additional studies are needed to further elucidate the complicated regulatory network of the ISWI complex.

MAPK signalling pathways are ubiquitous in eukaryotes and play important roles in cellular growth, differentiation and stress responses (Babazadeh et al. 2014; Saito and Posas 2012). The pathways are versatile signalling tools and comprise a three‐tiered protein kinase cascade: a MAP kinase kinase kinase (MAPKKK), a MAP kinase kinase (MAPKK) and a MAPK that are activated by sequential phosphorylation upon pathway stimulation (Cuevas et al. 2007). This multilayered mechanistic structure provides considerable flexibility of system responses, allowing for signal amplification, multiple points of regulation, noise tolerance and the generation of switch‐like responses. Three MAPK signalling pathways have been characterised in *F. graminearum* that play crucial roles in environmental stress adaptation and virulence regulation (Gu et al. 2015; Zheng et al. 2012). Here, FgDDT‐FgISW1 was demonstrated to specifically regulate MAPK genes, enhance signal amplification, improve adaptation to environmental stresses and facilitate flexible regulation. Thus, further investigations are needed to elucidate the mechanisms of interaction between the ISWI complex and the MAPK pathway, which would then allow a comprehensive understanding of the diverse functions that the ISWI complex achieves in various fungal systems.

Taken together, this study reveals a novel regulatory mechanism in *F. graminearum* involving the transcription factor FgDDT that forms a complex with FgISW1 to activate the expression of specific genes. While TFs like FgDDT are highly conserved in *Fusarium*, they exhibit low similarity with counterparts in *Homo sapiens* and *A. thaliana*, underscoring the potential for unique functional roles. Notably, the ISWI components FgISW1 and FgDDT, rather than FgISW2, are indispensable for fungal development and pathogenicity. Understanding the regulatory mechanisms of TFs in plant‐pathogenic fungi is fundamental for identifying novel pathogenicity factors and identifying regulatory networks associated with pathogen evolution. Moreover, TFs themselves can be targeted in disease control strategies. Our findings demonstrate that FgDDT, through its interaction with FgISW1, plays a pivotal role in the ISWI complex of this damaging pathogenic fungus. By targeting this complex, we can potentially affect multiple downstream pathogenicity factors simultaneously, offering a strategy for disrupting the pathogen's pathogenicity network.

## 4 Experimental Procedures

### 4.1 Fungal Strains and Culture Conditions

The *F. graminearum* WT strain PH‐1 (NRRL 31084) served as the parental strain for transformation. For mycelial growth tests, PH‐1 and transformants were cultured on potato dextrose agar (PDA) (200 g potato, 20 g glucose, 10 g agar, 1 L water), complete medium (CM) (10 g glucose, 2 g peptone, 1 g yeast extract, 1 g casamino acids, nitrate salts, trace elements, 0.01% of vitamins, 10 g agar, 1 L water, pH 6.5) or minimal medium (MM) (10 mM K_2_HPO_4_, 10 mM KH_2_PO_4_, 4 mM (NH_4_)_2_SO_4_, 2.5 mM NaCl, 2 mM MgSO_4_, 0.45 mM CaCl_2_, 9 mM FeSO_4_, 10 mM glucose, 1% agar, pH 6.9) (Yun et al. 2015) at 25°C.

Sexual reproduction analysis involved pressing down the aerial hyphae of 7‐day‐old cultures of each strain grown on carrot agar plates supplemented with 0.1% Tween 20. Perithecium formation and cirrhi production were assayed after incubation under black light at 25°C for 15 days (Cavinder et al. 2012). Conidia were obtained by culturing conidia in liquid carboxymethyl cellulose (CMC, 0.5 g/L MgSO_4_.7H_2_O, 1 g/L NH_4_NO_3_, 1 g/L KH_2_PO_4_, 1 g/L yeast extract, 15 g/L carboxymethylcellulose sodium) medium at 25°C and 180 rpm for 4 days.

To test DON production, strains were cultured in yeast extract peptone glucose medium (YEPD, 3 g/L yeast extract, 10 g/L peptone, 20 g/L d‐glucose) at 25°C for 24 h at 180 rpm. The hyphae were collected and transferred to trichothecene biosynthesis induction (TBI, 30 g sucrose, 1 g KH_2_PO_4_, 10 mg FeSO_4_·7H_2_O, 0.5 g MgSO_4_·7H_2_O, 0.5 g KCl, 1.47 g putrescine hydrochloride, 200 μL trace element solution, 1 L water) medium and shaken in the dark for 2 days, then the DON yield of each sample was extracted and quantified by the DON kit (Wise Science). The dry weight of mycelium is used as an internal reference.

### 4.2 Generation of Gene Deletion and Complementation

Gene deletion was performed using double‐joint PCR, as previously described (Zhao et al. 2024). Targeted gene replacements were carried out using the *HPH* gene, conferring resistance to hygromycin. Putative gene deletion mutants were identified by PCR assays with the relevant primers and were further analysed by Southern blot analyses. To achieve complementation of mutants, full‐length genes, including their native promoter, were amplified and inserted into the pYF11‐GFP vector. The construct was then transformed into the protoplast of the mutant. Details of the information for all primers were shown in Table S2.

### 4.3 Pathogenicity Assays

Pathogenicity of each strain on wheat spikelet and wheat seedling leaf was assayed as described previously (Gu et al. 2022). Briefly, the pathogenicity on wheat heads was evaluated by point‐inoculation with a conidial suspension at a concentration of 10^6^ conidia/mL. Infected spikelets were then recorded, and images were photographed at 14 days after inoculation. In addition, 5‐mm mycelial plugs from the edge of a 3‐day‐old PDA plate of each strain were inoculated onto wheat seedling leaves. Disease lesions were scored and photographed at 5 dpi. Pathogenicity assays were repeated in triplicate.

### 4.4 ChIP‐Seq and ChIP‐qPCR

ChIP assays were performed as previously described (Gu et al. 2022). The mycelia from Δ*FgDDT‐*C strain were first fixed with 1% formaldehyde and stopped with 125 mM glycine for 5 min. Subsequently, fixed culture samples were ground using liquid nitrogen and resuspended in 10 mL of nuclear extraction buffer I (0.4 M sucrose, 10 mM Tris–HCl pH 8.0, 10 mM MgCl_2_, 5 mM β‐mercaptoethanol, 0.1 mM PMSF, 1× protease inhibitor). Then, chromatin was sheared into 500 bp fragments using an E220 DNA Sonicator (Covaris). The particulate matter was resuspended in a 3 mL aliquot of nuclear extraction buffer II (1.7 M sucrose, 10 mM Tris–HCl pH 8.0, 10 mM MgCl_2_, 1% Triton X‐100, 5 mM β‐mercaptoethanol and a protease inhibitor cocktail). Following resuspension, the samples underwent centrifugation at 13,000 *g* for 15 min at 4°C. Subsequently, the precipitated material was redispersed in 300 μL of a nuclear lysis buffer (50 mM Tris–HCl pH 8.0, 10 mM EDTA, 1% SDS and a protease inhibitor cocktail). The samples were then subjected to sonication, involving two 30 s pulses interspersed with a 1 min rest interval. After centrifugation at 4000 *g* for 5 min at 4°C, the clarified supernatant was transferred to a transparent vessel and diluted with 10× ChIP dilution buffer (1.1% Triton X‐100, 1.2 mM EDTA, 16.7 mM Tris–HCl pH 8.0, 167 mM NaCl). For immunoprecipitation, a monoclonal anti‐GFP antibody (ab290, Abcam; 1:500 dilution) was employed in conjunction with protein A‐conjugated agarose beads (sc‐2003; Santa Cruz). The bead‐bound complexes were sequentially washed with buffers of varying salt concentrations (low and high salt), LiCl wash buffer and finally Tris‐EDTA buffer. Following the wash steps, the immunoprecipitated material was eluted, cross‐linking was reversed, and all proteins were removed. The final pellets were resuspended in 50 μL distilled water and DNA was extracted using the phenol/chloroform method. Half of the extracted DNA was then subjected to high‐throughput sequencing on the Illumina Hiseq4000 platform at BGI (Shenzhen, China), while the other half was used for ChIP‐qPCR.

For ChIP‐seq analysis, the raw sequencing reads were quality‐checked using SOAPnuke (v. 2.1.7) (Chen et al. 2018) and then aligned to the reference genome of *F. graminearum* PH‐1 by Bowtie2 (v. 2.4.5) with default parameters (Langmead and Salzberg 2012). The reads per kilobase per million mapped reads (RPKM) were calculated to normalise reads using the bamCompare tool in Deeptools (v. 2.4.1) with input DNA as the control (Ramírez et al. 2014). Two biological replicates were included for the ChIP‐seq and sequential ChIP‐seq assays. The resulting bigwig files were then loaded into the genome browser IGV (v. 2.8.9) (Robinson et al. 2011) and visually analysed. MACS2 (v. 2.1.4) (Zhang et al. 2008) was used to call peaks as previously described (You et al. 2017). The ChIP‐seq data have been deposited in the NCBI BioProject database with accession code GSE213962 (https://www.ncbi.nlm.nih.gov/geo/query/acc.cgi?acc=GSE265948, Enter token ojwpqwgcvrizhmt into the box).

For ChIP‐qPCR assays, relative enrichment values were calculated by dividing the amount of immunoprecipitated DNA by the amount of input DNA. PCR primers were designed to amplify 110–160 bp fragments from the selected genomic regions (Table S2). ChIP‐qPCR assays were repeated independently three times.

### 4.5 Co‐IP Assay

To evaluate the interaction between FgDDT and FgISW1, the *FgDDT* and *FgISW1* genes were ligated to a pHZ126‐FLAG and pYF11‐GFP vector, respectively. The constructs were co‐transformed into the wild‐type PH‐1 strain. Total proteins were extracted from the resulting transformants by first grinding 5 g of mycelia into a fine powder using liquid nitrogen and then adding an extraction buffer consisting of 50 mM Tris–HCl (pH 7.5), 100 mM NaCl, 5 mM EDTA, 1% Triton X‐100 and 0.1% (vol/vol) protease inhibitor cocktail (Sigma). The mixture was incubated with gentle agitation at 4°C for 25 min to facilitate efficient protein extraction. After centrifugation, the supernatant containing the total proteins was collected. For Co‐IP, FLAG beads (Sigma) were added for Co‐IP, followed by incubation at 4°C overnight. The beads immunoprecipitated proteins were washed with Tris‐buffered saline (TBS) three times and collected. Then, TBS was added to mix with immunoprecipitated proteins and boiled at 98°C for 10 min. The boiled samples were separated on a 10% SDS‐PAGE gel and blotted with corresponding antibodies (anti‐FLAG; Sigma‐Aldrich, 1:10,000 dilution; anti‐GFP, Abcam, 1:10,000 dilution). The monoclonal anti‐GAPDH antibody (HuaAn Biotech, 1:10,000 dilution) was used as a reference.

### 4.6 Y2H Assays

Y2H assay was performed as previously described (Gu et al. 2022). Briefly, the *FgDDT*, *FgISW2* and *FgISW1* genes were ligated into pGADT7 and pGBKT7 vectors. The constructs were co‐transformed into the AH109 reporter strain, and the transformed strains were grown on a synthetic defined (SD) medium without leucine and tryptophan. The vectors pGBKT7‐Lam and pGBKT7 served as a negative control, while the vectors pGBKT7‐53 and pGADT7 served as a positive control. Protein–protein interactions were demonstrated via the growth of transformants on SD without histidine, leucine, tryptophan and adenine. The experiments were repeated in triplicate.

### 4.7 BiFC Assay

For BiFC assays, the *FgDDT* and *FgISW1* genes were ligated to pHZ65 and pHZ68 vectors, respectively, and the constructs were co‐transformed into the wild‐type PH‐1 strain. Transformants resistant to hygromycin and zeocin were verified by PCR and examined for yellow fluorescent protein (YFP) signals by epifluorescence microscopy after staining with 4,6‐diamidino‐2‐phenylindole (DAPI) as described (Gu et al. 2022).

### 4.8 Protein Purification

The full‐length open reading frame (ORF) of *FgDDT* was cloned into the pET28a vector and transformed into *Escherichia coli* BL21 (DE3). Subsequently, bacteria were inoculated on 20 mL Luria Bertani (LB) medium at 37°C and 180 rpm for 12 h. Following this, 1% of the bacterial volume was transferred to 200 mL LB medium and cultured at 37°C and 180 rpm for 2–3 h until OD_600_ reached 0.6. At this point, isopropyl β‐d‐1‐thiogalactopyranoside (IPTG) was added to the culture medium at a final concentration of 0.1 mM at 16°C and 180 rpm with shaking overnight. The bacteria were centrifuged at 5000 *g* at 4°C for 30 min and resuspended in Buffer A or phosphate‐buffered saline (PBS). After ultrasonic crushing, the supernatant was harvested at 4°C at 10,000 *g* for 20 min, and the protein was purified using the AKTA avant 25 (GE Healthcare). The expression of proteins was evaluated using SDS‐PAGE.

### 4.9 EMSA

DNA fragments of *FgHog1* and *FgNdpk* were amplified using primers labelled with FAM. The purified PCR product (100 ng) was mixed with different concentrations of FgDDT protein in a 20‐μL reaction mixture containing 10 mM Tris (pH 7.5), 50 mM KCl, 1 mM dithiothreitol and 0.4% glycerol. Subsequently, the reaction mixture was incubated at room temperature for 30 min, followed by electrophoresis on a 6% native‐PAGE gel at 4°C in 0.5 × TBE buffer (5.4 g Tris base, 2.75 g boric acid, 0.465 g EDTA disodium salt and 1 L water, pH 8.3). The DNA fragments were visualised using a ChemiDoc MP system (Bio‐Rad) at an excitation wavelength of 492 nm. In this assay, different amounts of unlabelled DNA were added to the reaction as cold excess competitors.

### 4.10 RT‐qPCR

For RT‐qPCR tests, PH‐1 and transformants were grown on PDA plates at 25°C for 3 days. Subsequently, 5 mm mycelial discs were excised from the edges of these colonies and transferred to YEPD medium. The discs were incubated in YEPD for 36 h with agitation. Total RNA was then extracted from the samples using the TRIzol reagent (TaKaRa). The first‐strand cDNA was synthesised using the Primescript first‐strand cDNA synthesis system (TaKaRa). qPCR was performed using iTaq SYBR Green Master Mix (Bio‐Rad). *FgACTIN* was used as the internal control. The relative expression levels were calculated using the 2^−ΔΔ*C*t^ method. Sequences of primers used for RT‐qPCR are provided in Table S2.

### 4.11 Phylogenetic Analysis

Phylogenetic trees were constructed from the genomes analysed in this study using the MEGA X software program (https://www.megasoftware.net/) and neighbour‐joining methods with parameter settings including 1000 bootstraps, p‐distance as the substitution matrix, and pairwise deletion of sites. In this study, all genomes were obtained from the National Center for Biotechnology Information (NCBI; http://www.ncbi.nlm.nih.gov/). Accession numbers for all the proteins used in this article are listed in Table S3.

### 4.12 Statistical Analysis

Data were analysed using SPSS v. 21 software. Experiments were repeated in triplicate, and one‐way analysis of variance (ANOVA, *p* < 0.05) was used to analyse the data.

## Conflicts of Interest

The authors declare no conflicts of interest.

## Supporting information


**Figure S1.** Generation and confirmation of targeted gene deletion. Gene disruption strategy for targeted gene. In the left panel, the targeted gene and hygromycin resistance cassette (*HPH*) are indicated by large green and blue arrows, respectively. Gene deletion was confirmed by PCR and Southern blot assays in the right panel.

**Figure S2.** FgDDT directly bound to the promoters of FgHog1 and FgNdpk. Different concentrations of FgDDT were added to mixtures containing 100 ng of FAM probe. Unlabelled probes were added as cold excess competitors.

**Figure S3.** Sequence alignment of ISW1 and ISW2 protein from *Fusarium graminearum*.

**Figure S4.** Mutual regulation of FgDDT and FgISW1 expression levels. (a) Comparisons in relative gene expression of *FgDDT* and *FgISW1* among the PH‐1 (wild‐type [WT]), mutants and complementary strains. Data are presented as the mean ± SD from three repeated experiments. Different letters indicate a significant difference (*p* < 0.05, one‐way ANOVA). (b) Western blot analysis of FgDDT‐GFP and FgISW1‐GFP protein levels in WT PH‐1, the Δ*FgISW1* mutant expressing FgDDT‐GFP and the Δ*FgDDT* mutant expressing FgISW1‐GFP. An anti‐GFP antibody was used for detection, with GAPDH serving as the loading control. Band intensities were quantified using ImageJ software. (c) Fluorescence signals of FgISW1‐GFP in WT PH‐1 and Δ*FgDDT* mutant, and FgDDT‐GFP in WT PH‐1 and Δ*FgISW1* mutant. The constructs were stained with 4,6‐diamidino‐2‐phenylindole (DAPI) and examined by epifluorescence microscopy. Bar, 10 μm (upper panel). White arrows highlight areas analysed by line‐scan graph analysis. *y* axis: The intensity of GFP and DAPI signals quantified by ImageJ; *x* axis: The distance (μm) (lower panel).

**Table S1.** The 2429 FgDDT‐targeted genes.

**Table S2.** Primers used in this study.

**Table S3.** Accession numbers for proteins used in the study.

## Data Availability

The ChIP‐seq data have been deposited in the NCBI BioProject database with accession code GSE213962 (https://www.ncbi.nlm.nih.gov/geo/query/acc.cgi?acc=GSE265948, Enter token ojwpqwgcvrizhmt into the box). Other data that support the findings of this article are available from the corresponding author upon request.
