# Supplementary material for: Transcription Activator FgDDT Interacts With FgISW1 to Regulate Fungal Development and Pathogenicity in the Global Pathogen Fusarium graminearum
Source: Mol Plant Pathol. 2025 Mar 28;26(4):e70076. doi: 10.1111/mpp.70076 (PMC11950633; doi:10.1111/mpp.70076)
Supplement: Supplementary file 1 — Figure S1. Generation and confirmation of targeted gene deletion. Gene disruption strategy for targeted gene. In the left panel, the targeted gene and hygromycin resistance cassette (HPH) are indicated by large green and blue arrows, respectively. Gene deletion was confirmed by PCR and Southern blot assays in the right panel. [file MPP-26-e70076-s004.pdf]

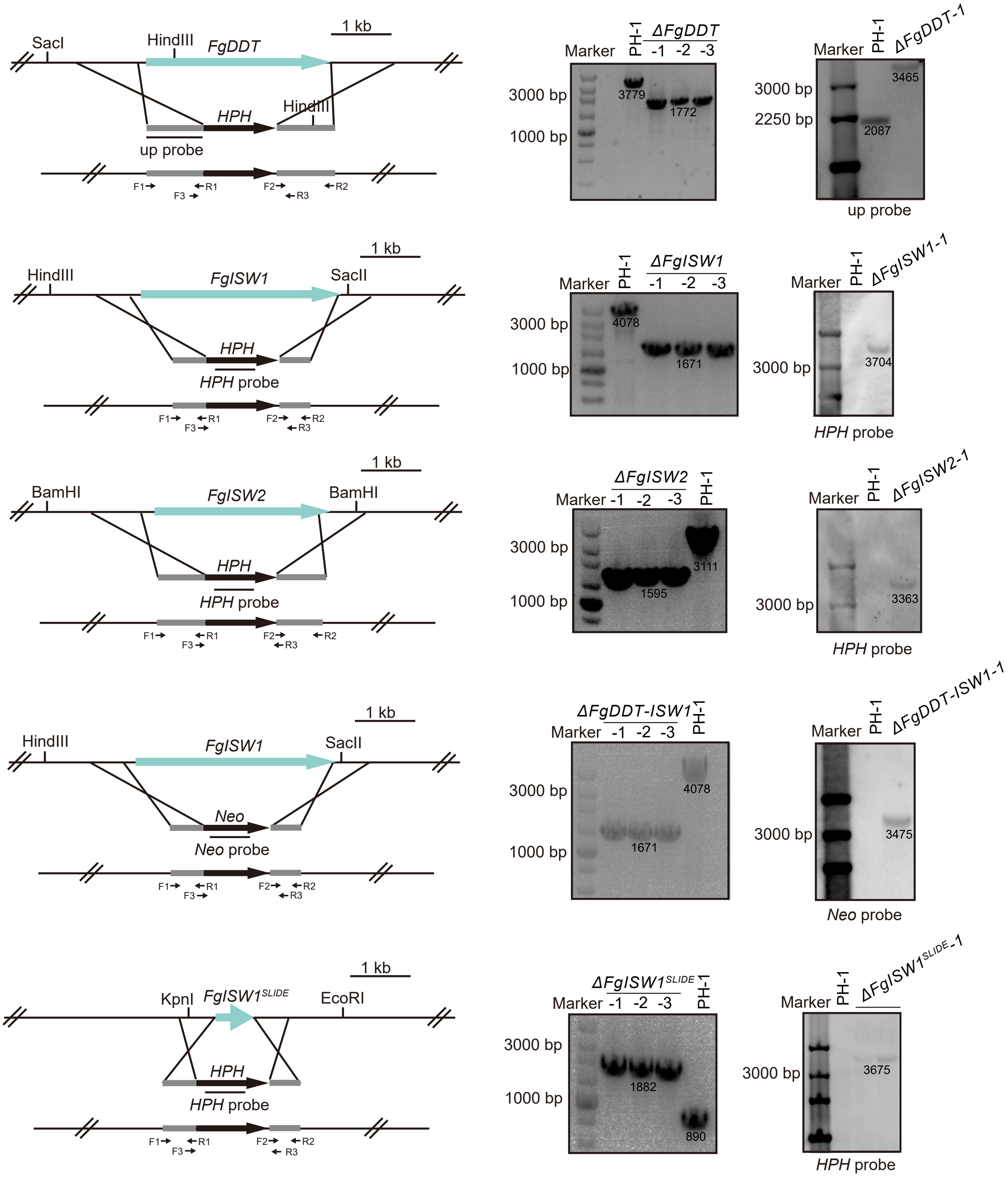

Figure S1 Generation and confirmation of targeted gene deletion. Gene disruption strategy for targeted gene. In the left panel, the targeted gene and hygromycin resistance cassette (*HPH*) are indicated by large green and blue arrows, respectively. Gene deletion was confirmed by PCR and Southern blot assays in the right panel.
