# Supplementary material for: Transcription Activator FgDDT Interacts With FgISW1 to Regulate Fungal Development and Pathogenicity in the Global Pathogen Fusarium graminearum
Source: Mol Plant Pathol. 2025 Mar 28;26(4):e70076. doi: 10.1111/mpp.70076 (PMC11950633; doi:10.1111/mpp.70076)
Supplement: Supplementary file 2 — Figure S2. FgDDT directly bound to the promoters of FgHog1 and FgNdpk. Different concentrations of FgDDT were added to mixtures containing 100 ng of FAM probe. Unlabelled probes were added as cold excess competitors. [file MPP-26-e70076-s007.pdf]

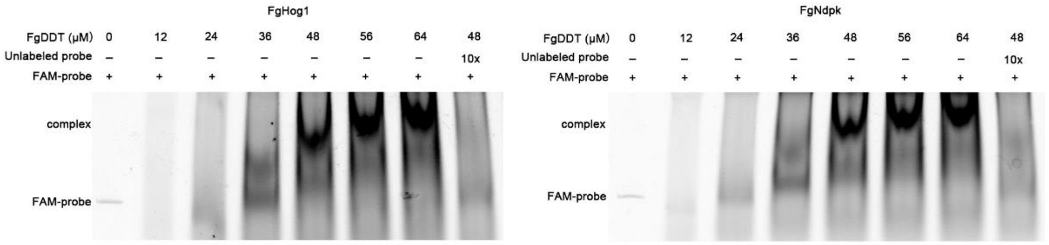

Figure S2 FgDDT directly bound to the promoters of FgHog1, and FgNdpk. Different concentrations of FgDDT were added to mixtures containing 100 ng of FAM probe. Unlabelled probes were added as cold excess competitors.
