# Supplementary material for: Transcription Activator FgDDT Interacts With FgISW1 to Regulate Fungal Development and Pathogenicity in the Global Pathogen Fusarium graminearum
Source: Mol Plant Pathol. 2025 Mar 28;26(4):e70076. doi: 10.1111/mpp.70076 (PMC11950633; doi:10.1111/mpp.70076)
Supplement: Supplementary file 3 — Figure S3. Sequence alignment of ISW1 and ISW2 protein from Fusarium graminearum. [file MPP-26-e70076-s002.pdf]

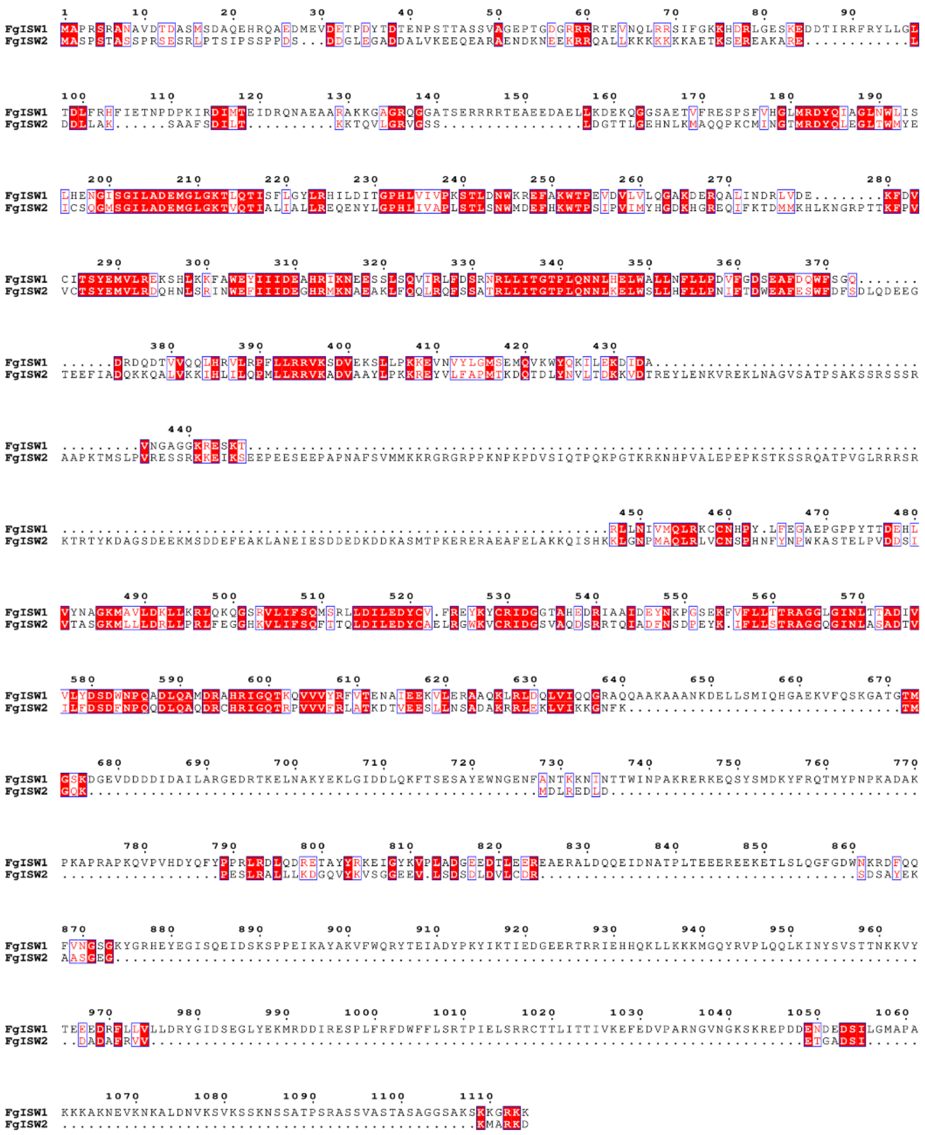

Figure S3 Sequence alignment of ISW1 and ISW2 protein from *F. graminearum*.
