# Supplementary material for: Transcription Activator FgDDT Interacts With FgISW1 to Regulate Fungal Development and Pathogenicity in the Global Pathogen Fusarium graminearum
Source: Mol Plant Pathol. 2025 Mar 28;26(4):e70076. doi: 10.1111/mpp.70076 (PMC11950633; doi:10.1111/mpp.70076)
Supplement: Supplementary file 4 — Figure S4. Mutual regulation of FgDDT and FgISW1 expression levels. (a) Comparisons in relative gene expression of FgDDT and FgISW1 among the PH‐1 (wild‐type [WT]), mutants and complementary strains. Data are presented as the mean ± SD from three repeated experiments. Different letters indicate a significant difference (p < 0.05, one‐way ANOVA). (b) Western blot analysis of FgDDT‐GFP and FgISW1‐GFP protein levels in WT PH‐1, the ΔFgISW1 mutant expressing FgDDT‐GFP and the ΔFgDDT mutant expressing FgISW1‐GFP. An anti‐GFP antibody was used for detection, with GAPDH serving as the loading control. Band intensities were quantified using ImageJ software. (c) Fluorescence signals of FgISW1‐GFP in WT PH‐1 and ΔFgDDT mutant, and FgDDT‐GFP in WT PH‐1 and ΔFgISW1 mutant. The constructs were stained with 4,6‐diamidino‐2‐phenylindole (DAPI) and examined by epifluorescence microscopy. Bar, 10 μm (upper panel). White arrows highlight areas analysed by line‐scan graph analysis. y axis: The intensity of GFP and DAPI signals quantified by ImageJ; x axis: The distance (μm) (lower panel). [file MPP-26-e70076-s001.pdf]

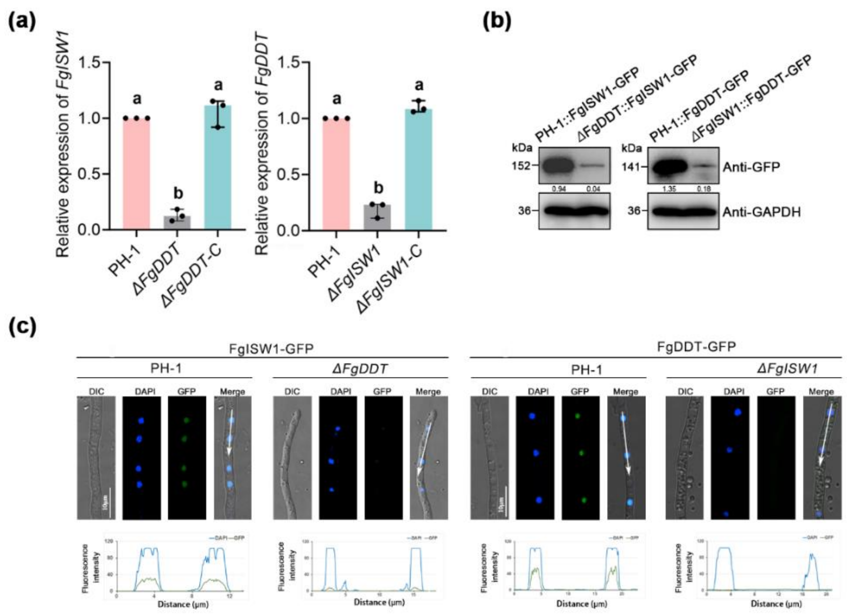


**Figure S4 Mutual regulation of FgDDT and FgISW1 expression levels.** (a) Comparisons in relative gene expression of *FgDDT* and *FgISW1* among the PH-1 (wild-type (WT)), mutants and complementary strains. Data are presented as the means ± standard deviation from three repeated experiments. Different letters indicate a significant difference ($p < 0.05$, one-way ANOVA). (b) Western blot analysis of FgDDT-GFP and FgISW1-GFP protein levels in WT PH-1, the *ΔFgISW1* mutant expressing FgDDT-GFP, and the *ΔFgDDT* mutant expressing FgISW1-GFP. An anti-GFP antibody was used for detection, with GAPDH serving as the loading control. Band intensities were quantified using ImageJ software. (c) Fluorescence signals of FgISW1-GFP in WT PH-1 and *ΔFgDDT* mutant, and FgDDT-GFP in WT PH-1 and *ΔFgISW1* mutant. The constructs were stained with 4,6-diamidino-2-phenylindole (DAPI) and examined by epifluorescence microscopy. Bar, 10 μm (upper panel). White arrows highlight areas analyzed by line-scan graph analysis. y axis: the intensity of GFP and DAPI signals quantified by ImageJ; x axis: the distance (μm) (lower panel).
