# Supplementary material for: Transcription Activator FgDDT Interacts With FgISW1 to Regulate Fungal Development and Pathogenicity in the Global Pathogen Fusarium graminearum
Source: Mol Plant Pathol. 2025 Mar 28;26(4):e70076. doi: 10.1111/mpp.70076 (PMC11950633; doi:10.1111/mpp.70076)
Supplement: Supplementary file 5 — Table S1. The 2429 FgDDT‐targeted genes. [file MPP-26-e70076-s003.pdf]

Table S1 The 2429 FgDDT-targeted genes

Gene Id	Transcript Id	Fold_enrichment	-Log10(p value)	Annotation	Gene Chr	Gene Start	Gene End	Gene Length	Gene Strand
FGRAMPH1_01G00009	FGRAMPH1_01T00009	1.32049	3.24221	Promoter	1	23519	25108	1590	2
FGRAMPH1_01G00035	FGRAMPH1_01T00035	2.13149	16.96381	Promoter	1	59208	59741	534	2
FGRAMPH1_01G00085	FGRAMPH1_01T00085	1.83786	10.2759	Promoter	1	111446	112928	1483	1
FGRAMPH1_01G00119	FGRAMPH1_01T00119	1.70077	7.71086	Promoter	1	145853	146762	910	2
FGRAMPH1_01G00183	FGRAMPH1_01T00183	1.65859	7.00562	Promoter	1	250039	252583	2545	1
FGRAMPH1_01G00193	FGRAMPH1_01T00193	1.73241	8.44624	Promoter	1	260087	261759	1673	2
FGRAMPH1_01G00311	FGRAMPH1_01T00311	1.5621	6.09943	Exon	1	383200	384898	1699	2
FGRAMPH1_01G00331	FGRAMPH1_01T00331	2.58657	29.90888	Promoter	1	404163	405681	1519	2
FGRAMPH1_01G00349	FGRAMPH1_01T00349	1.64804	6.77735	Promoter	1	427648	430213	2566	1
FGRAMPH1_01G00373	FGRAMPH1_01T00373	2.09094	15.99497	Promoter	1	451081	453033	1953	2
FGRAMPH1_01G00407	FGRAMPH1_01T00407	1.61641	6.33116	Promoter	1	502213	502690	478	1

FGRAMPH1_01G00413	FGRAMPH1_01T00413	1.61745	8.08176	Promoter	1	504709	507093	2385	2
ENSRNA049558238	ENSRNA049558238-T1	1.74697	11.36834	Promoter	1	773680	773762	83	1
FGRAMPH1_01G00693	FGRAMPH1_01T00693	1.44603	5.27338	Promoter	1	812457	813662	1206	2
FGRAMPH1_01G00695	FGRAMPH1_01T00695	2.23858	19.7708	Promoter	1	814840	815369	530	2
FGRAMPH1_01G00723	FGRAMPH1_01T00723	1.66519	9.20118	Promoter	1	849900	850115	216	2
FGRAMPH1_01G00725	FGRAMPH1_01T00725	1.45088	5.85903	Intergenic	1	850164	850346	183	1
FGRAMPH1_01G00727	FGRAMPH1_01T00727	1.92043	13.29476	Promoter	1	852499	852618	120	1
FGRAMPH1_01G00729	FGRAMPH1_01T00729	1.51623	5.31223	Promoter	1	853724	856294	2571	1
FGRAMPH1_01G00731	FGRAMPH1_01T00731	1.89058	11.39068	Promoter	1	856788	858004	1217	1
FGRAMPH1_01G00743	FGRAMPH1_01T00743	1.65859	7.00562	Promoter	1	866021	867216	1196	2
FGRAMPH1_01G00745	FGRAMPH1_01T00745	1.85895	10.82712	Promoter	1	868298	871181	2884	1
FGRAMPH1_01G00747	FGRAMPH1_01T00747	2.15127	20.41103	Promoter	1	871843	873838	1996	1
FGRAMPH1_01G00749	FGRAMPH1_01T00749	1.87102	13.22794	Promoter	1	875098	876060	963	1

FGRAMPH1_01G00751	FGRAMPH1_01T00751	1.7533	9.40954	Exon	1	876258	876988	731	2
ENSRNA049559570	ENSRNA049559570-T1	2.66373	34.9205	Promoter	1	877084	877191	108	2
ENSRNA049559551	ENSRNA049559551-T1	2.59911	31.60721	Promoter	1	898248	898342	95	2
FGRAMPH1_01G00773	FGRAMPH1_01T00773	1.59214	6.15086	Promoter	1	903751	905961	2211	2
FGRAMPH1_01G00797	FGRAMPH1_01T00797	1.45533	4.38936	Promoter	1	941020	942773	1754	2
FGRAMPH1_01G00799	FGRAMPH1_01T00799	1.92222	12.62466	Exon	1	944042	944738	697	1
FGRAMPH1_01G00809	FGRAMPH1_01T00809	1.59034	6.41693	UTR	1	955296	956278	983	2
FGRAMPH1_01G00811	FGRAMPH1_01T00811	1.39956	4.19374	Promoter	1	956524	958461	1938	1
FGRAMPH1_01G00821	FGRAMPH1_01T00821	1.41582	4.64413	Promoter	1	967305	969217	1913	1
FGRAMPH1_01G00843	FGRAMPH1_01T00843	1.63189	8.61239	Promoter	1	1004423	1008772	4350	1
FGRAMPH1_01G00869	FGRAMPH1_01T00869	1.75606	8.83689	Promoter	1	1044183	1046989	2807	1
FGRAMPH1_01G00875	FGRAMPH1_01T00875	1.63001	8.01086	Promoter	1	1057588	1059386	1799	1
FGRAMPH1_01G00887	FGRAMPH1_01T00887	1.65281	9.89002	Exon	1	1072562	1072993	432	1

FGRAMPH1_01G00889	FGRAMPH1_01T00889	2.1378	17.65139	Intergenic	1	1074844	1075050	207	2
FGRAMPH1_01G00891	FGRAMPH1_01T00891	2.09918	16.69219	Exon	1	1076497	1076971	475	1
FGRAMPH1_01G00897	FGRAMPH1_01T00897	1.90161	12.08188	Promoter	1	1085519	1085720	202	1
FGRAMPH1_01G00963	FGRAMPH1_01T00963	1.59169	9.47847	Promoter	1	1180864	1182071	1208	2
FGRAMPH1_01G00975	FGRAMPH1_01T00975	1.64418	7.71088	Promoter	1	1195398	1195808	411	2
ENSRNA049558251	ENSRNA049558251-T1	2.4826	28.16008	Promoter	1	1205702	1205783	82	1
FGRAMPH1_01G01007	FGRAMPH1_01T01007	1.65618	8.31919	Promoter	1	1239346	1240563	1218	2
FGRAMPH1_01G01019	FGRAMPH1_01T01019	1.57804	7.1389	Promoter	1	1253823	1255873	2051	2
FGRAMPH1_01G01021	FGRAMPH1_01T01021	1.89585	12.15326	Promoter	1	1259131	1259289	159	1
FGRAMPH1_01G01025	FGRAMPH1_01T01025	1.48521	4.74017	Intergenic	1	1262462	1262779	318	2
FGRAMPH1_01G01031	FGRAMPH1_01T01031	1.52213	4.99144	UTR	1	1267837	1268325	489	1
FGRAMPH1_01G01057	FGRAMPH1_01T01057	2.72285	34.88578	Promoter	1	1303886	1305611	1726	2
FGRAMPH1_01G01091	FGRAMPH1_01T01091	1.84389	13.08026	Promoter	1	1354389	1357193	2805	2

FGRAMPH1_01G01097	FGRAMPH1_01T01097	1.91167	11.96643	UTR	1	1361102	1361552	451	2
FGRAMPH1_01G01099	FGRAMPH1_01T01099	1.57074	6.49577	Promoter	1	1365853	1366494	642	1
FGRAMPH1_01G01127	FGRAMPH1_01T01127	1.89094	12.46223	Promoter	1	1396395	1403067	6673	2
FGRAMPH1_01G01129	FGRAMPH1_01T01129	1.84993	10.91785	Promoter	1	1404676	1405422	747	1
FGRAMPH1_01G01131	FGRAMPH1_01T01131	1.57423	5.68813	Promoter	1	1406393	1408210	1818	1
FGRAMPH1_01G01139	FGRAMPH1_01T01139	2.05337	18.01651	Promoter	1	1415984	1418271	2288	1
FGRAMPH1_01G01147	FGRAMPH1_01T01147	1.29728	3.24372	Promoter	1	1424950	1426996	2047	1
FGRAMPH1_01G01149	FGRAMPH1_01T01149	1.63231	6.94274	Promoter	1	1427617	1428835	1219	2
FGRAMPH1_01G01153	FGRAMPH1_01T01153	1.46314	5.67897	Promoter	1	1434789	1437914	3126	1
FGRAMPH1_01G01169	FGRAMPH1_01T01169	1.4068	4.84032	Promoter	1	1452781	1453575	795	2
FGRAMPH1_01G01175	FGRAMPH1_01T01175	1.40792	4.97173	Promoter	1	1458219	1459958	1740	2
FGRAMPH1_01G01191	FGRAMPH1_01T01191	1.34692	3.23188	Promoter	1	1474808	1476019	1212	2
FGRAMPH1_01G01193	FGRAMPH1_01T01193	1.64136	10.15819	Intergenic	1	1478537	1478968	432	2

FGRAMPH1_01G01197	FGRAMPH1_01T01197	1.50041	4.49906	Promoter	1	1483415	1485202	1788	1
ENSRNA049513750	ENSRNA049513750-T1	2.37033	24.78893	Exon	1	1486663	1486855	193	2
FGRAMPH1_01G01205	FGRAMPH1_01T01205	1.43844	5.77825	Promoter	1	1492870	1494408	1539	1
FGRAMPH1_01G01215	FGRAMPH1_01T01215	1.7676	9.26589	Promoter	1	1504419	1505009	591	2
FGRAMPH1_01G01217	FGRAMPH1_01T01217	1.84391	10.8898	Promoter	1	1505598	1506218	621	2
ENSRNA049559536	ENSRNA049559536-T1	2.68148	32.90722	Promoter	1	1519927	1519998	72	2
ENSRNA049559522	ENSRNA049559522-T1	2.32628	26.20987	Promoter	1	1532711	1532805	95	2
FGRAMPH1_01G01253	FGRAMPH1_01T01253	2.68148	32.90722	Promoter	1	1544479	1547795	3317	1
FGRAMPH1_01G01255	FGRAMPH1_01T01255	1.46225	5.93382	Promoter	1	1548490	1550845	2356	2
FGRAMPH1_01G01259	FGRAMPH1_01T01259	1.28202	3.61625	Exon	1	1554766	1555821	1056	2
FGRAMPH1_01G01261	FGRAMPH1_01T01261	1.80102	12.64722	Promoter	1	1558559	1558780	222	1
FGRAMPH1_01G01263	FGRAMPH1_01T01263	1.88106	11.79056	Promoter	1	1558780	1560202	1423	1
FGRAMPH1_01G01279	FGRAMPH1_01T01279	1.58312	6.31612	Promoter	1	1571819	1572588	770	1

FGRAMPH1_01G01283	FGRAMPH1_01T01283	1.57049	6.5916	Promoter	1	1574839	1576161	1323	2
FGRAMPH1_01G01313	FGRAMPH1_01T01313	1.27499	3.09399	Promoter	1	1613391	1613711	321	2
FGRAMPH1_01G01315	FGRAMPH1_01T01315	1.83978	11.94666	Promoter	1	1615417	1615933	517	1
FGRAMPH1_01G01341	FGRAMPH1_01T01341	1.69022	7.4724	Promoter	1	1644330	1645224	895	1
FGRAMPH1_01G01345	FGRAMPH1_01T01345	1.35204	4.39531	Promoter	1	1653436	1656575	3140	1
FGRAMPH1_01G01353	FGRAMPH1_01T01353	2.21749	19.06036	Promoter	1	1665833	1668192	2360	1
FGRAMPH1_01G01361	FGRAMPH1_01T01361	1.69941	8.42869	UTR	1	1677252	1682507	5256	2
FGRAMPH1_01G01369	FGRAMPH1_01T01369	1.68091	10.57715	Promoter	1	1692561	1694381	1821	2
FGRAMPH1_01G01371	FGRAMPH1_01T01371	1.92028	17.69771	Exon	1	1695850	1697364	1515	1
FGRAMPH1_01G01373	FGRAMPH1_01T01373	1.8574	10.8725	Promoter	1	1697979	1700024	2046	1
FGRAMPH1_01G01391	FGRAMPH1_01T01391	1.79074	11.41036	Promoter	1	1721711	1722595	885	1
FGRAMPH1_01G01413	FGRAMPH1_01T01413	1.46415	5.42383	Promoter	1	1762437	1763462	1026	2
ENSRNA049559502	ENSRNA049559502-T1	2.37167	24.81595	Promoter	1	1768031	1768134	104	2

FGRAMPH1_01G01459	FGRAMPH1_01T01459	1.79703	9.93306	Promoter	1	1826318	1829999	3682	2
FGRAMPH1_01G01467	FGRAMPH1_01T01467	1.44388	5.17555	Promoter	1	1841779	1843302	1524	2
FGRAMPH1_01G01481	FGRAMPH1_01T01481	1.92855	13.76897	Promoter	1	1855839	1859648	3810	2
FGRAMPH1_01G01485	FGRAMPH1_01T01485	1.60261	6.58876	Promoter	1	1868212	1869559	1348	1
FGRAMPH1_01G01491	FGRAMPH1_01T01491	2.0804	15.66799	Promoter	1	1873878	1874318	441	2
FGRAMPH1_01G01493	FGRAMPH1_01T01493	2.0483	17.18208	Promoter	1	1876371	1876553	183	2
FGRAMPH1_01G01499	FGRAMPH1_01T01499	1.60404	8.6379	Promoter	1	1881206	1882526	1321	1
FGRAMPH1_01G01501	FGRAMPH1_01T01501	1.70731	10.87856	Exon	1	1882871	1883786	916	1
FGRAMPH1_01G01515	FGRAMPH1_01T01515	1.77866	9.69768	Promoter	1	1903712	1904098	387	1
FGRAMPH1_01G01517	FGRAMPH1_01T01517	1.78513	9.21114	Promoter	1	1904741	1906096	1356	2
FGRAMPH1_01G01531	FGRAMPH1_01T01531	1.7847	9.95343	Promoter	1	1924169	1925743	1575	2
FGRAMPH1_01G01533	FGRAMPH1_01T01533	1.63412	8.36519	Promoter	1	1926311	1928652	2342	2
FGRAMPH1_01G01535	FGRAMPH1_01T01535	1.7539	10.43614	UTR	1	1928983	1932615	3633	2

ENSRNA049558298	ENSRNA049558298-T1	1.78066	10.6957	Promoter	1	1966655	1966744	90	1
FGRAMPH1_01G01673	FGRAMPH1_01T01673	1.91271	12.50975	Promoter	1	2144296	2148029	3734	1
FGRAMPH1_01G01679	FGRAMPH1_01T01679	1.99967	16.46139	Promoter	1	2151804	2154047	2244	2
FGRAMPH1_01G01683	FGRAMPH1_01T01683	1.63207	7.77901	Promoter	1	2159028	2159360	333	1
FGRAMPH1_01G01687	FGRAMPH1_01T01687	1.61258	8.60579	Promoter	1	2163707	2169744	6038	1
ENSRNA049559477	ENSRNA049559477-T1	1.90998	12.219	Promoter	1	2169764	2169837	74	2
FGRAMPH1_01G01717	FGRAMPH1_01T01717	1.83462	13.54475	Promoter	1	2202034	2202261	228	2
FGRAMPH1_01G01741	FGRAMPH1_01T01741	1.98549	13.45803	Promoter	1	2232170	2233258	1089	1
FGRAMPH1_01G01743	FGRAMPH1_01T01743	2.05647	20.71819	Exon	1	2233765	2234858	1094	1
FGRAMPH1_01G01745	FGRAMPH1_01T01745	2.55494	28.6574	Promoter	1	2237433	2237781	349	1
FGRAMPH1_01G01747	FGRAMPH1_01T01747	1.83128	10.57745	Promoter	1	2246549	2248257	1709	1
FGRAMPH1_01G01761	FGRAMPH1_01T01761	2.10154	18.62733	Promoter	1	2263682	2266314	2633	2
FGRAMPH1_01G01769	FGRAMPH1_01T01769	1.47725	7.70307	Promoter	1	2281954	2282445	492	2

FGRAMPH1_01G01771	FGRAMPH1_01T01771	1.3664	4.12323	UTR	1	2286159	2291995	5837	2
FGRAMPH1_01G01773	FGRAMPH1_01T01773	1.26291	3.16663	Promoter	1	2294123	2294335	213	1
FGRAMPH1_01G01775	FGRAMPH1_01T01775	1.91841	13.1355	Promoter	1	2294383	2294604	222	2
FGRAMPH1_01G01777	FGRAMPH1_01T01777	1.76404	8.95294	Promoter	1	2294934	2295116	183	2
FGRAMPH1_01G01779	FGRAMPH1_01T01779	1.88193	14.08118	Promoter	1	2302119	2303525	1407	1
FGRAMPH1_01G01797	FGRAMPH1_01T01797	2.33199	23.15146	Promoter	1	2319403	2322385	2983	2
FGRAMPH1_01G01809	FGRAMPH1_01T01809	2.03822	14.70381	Intergenic	1	2336293	2336638	346	1
FGRAMPH1_01G01811	FGRAMPH1_01T01811	1.83018	12.63985	Promoter	1	2337900	2340381	2482	1
FGRAMPH1_01G01823	FGRAMPH1_01T01823	2.07605	18.8406	Promoter	1	2356890	2357380	491	1
FGRAMPH1_01G01825	FGRAMPH1_01T01825	2.18396	24.19732	Intergenic	1	2358378	2358792	415	1
FGRAMPH1_01G01827	FGRAMPH1_01T01827	2.33651	25.92894	Promoter	1	2362323	2365120	2798	1
FGRAMPH1_01G01829	FGRAMPH1_01T01829	2.08764	18.60641	Promoter	1	2367336	2370839	3504	1
FGRAMPH1_01G01831	FGRAMPH1_01T01831	1.5186	6.6748	Promoter	1	2370818	2372667	1850	2

FGRAMPH1_01G01841	FGRAMPH1_01T01841	1.93806	14.14959	Promoter	1	2387325	2390744	3420	2
FGRAMPH1_01G01849	FGRAMPH1_01T01849	1.47011	4.78095	Promoter	1	2403471	2404757	1287	1
FGRAMPH1_01G01919	FGRAMPH1_01T01919	1.73241	8.44624	Promoter	1	2488178	2489029	852	2
FGRAMPH1_01G01929	FGRAMPH1_01T01929	1.60154	7.65248	Promoter	1	2495481	2496854	1374	2
FGRAMPH1_01G01945	FGRAMPH1_01T01945	1.97885	13.3491	Promoter	1	2518101	2518741	641	1
FGRAMPH1_01G01973	FGRAMPH1_01T01973	1.6512	7.41582	Promoter	1	2546951	2547148	198	2
FGRAMPH1_01G01975	FGRAMPH1_01T01975	1.67968	7.4724	Promoter	1	2548318	2551118	2801	2
FGRAMPH1_01G01987	FGRAMPH1_01T01987	1.44516	5.39686	Intergenic	1	2564128	2564292	165	1
FGRAMPH1_01G01991	FGRAMPH1_01T01991	2.45816	30.44989	Promoter	1	2568260	2568980	721	2
FGRAMPH1_01G02007	FGRAMPH1_01T02007	2.34817	24.9952	Promoter	1	2595712	2599433	3722	1
ENSRNA049559461	ENSRNA049559461-T1	2.21175	23.06511	Promoter	1	2601476	2601558	83	2
ENSRNA049558325	ENSRNA049558325-T1	2.20694	19.06036	Promoter	1	2620722	2620793	72	1
FGRAMPH1_01G02033	FGRAMPH1_01T02033	1.86949	11.10737	UTR	1	2637259	2641878	4620	1

FGRAMPH1_01G02035	FGRAMPH1_01T02035	1.51346	4.80153	UTR	1	2640800	2641681	882	2
FGRAMPH1_01G02037	FGRAMPH1_01T02037	1.6371	7.48347	Promoter	1	2642541	2643572	1032	1
FGRAMPH1_01G02045	FGRAMPH1_01T02045	2.22803	19.41428	Promoter	1	2656082	2656270	189	1
FGRAMPH1_01G02047	FGRAMPH1_01T02047	1.79568	9.47257	Promoter	1	2658559	2661415	2857	1
FGRAMPH1_01G02049	FGRAMPH1_01T02049	1.5857	6.97653	Promoter	1	2661844	2664816	2973	1
FGRAMPH1_01G02101	FGRAMPH1_01T02101	1.70351	8.17186	Promoter	1	2739055	2739561	507	1
FGRAMPH1_01G02127	FGRAMPH1_01T02127	1.59101	8.02827	UTR	1	2779623	2780347	725	1
FGRAMPH1_01G02133	FGRAMPH1_01T02133	1.67968	7.4724	Promoter	1	2787984	2791582	3599	1
ENSRNA049559450	ENSRNA049559450-T1	2.17835	22.29883	Promoter	1	2794323	2794407	85	2
FGRAMPH1_01G02141	FGRAMPH1_01T02141	1.62726	8.70912	Promoter	1	2797475	2798447	973	2
FGRAMPH1_01G02143	FGRAMPH1_01T02143	1.9193	13.80581	Promoter	1	2802256	2808924	6669	1
FGRAMPH1_01G02145	FGRAMPH1_01T02145	1.52667	5.5751	Promoter	1	2809569	2811722	2154	2
FGRAMPH1_01G02147	FGRAMPH1_01T02147	1.6256	6.84981	Promoter	1	2815301	2815969	669	2

FGRAMPH1_01G02159	FGRAMPH1_01T02159	1.58175	6.16922	Promoter	1	2827488	2828725	1238	1
FGRAMPH1_01G02161	FGRAMPH1_01T02161	1.6375	6.77735	Promoter	1	2829388	2831231	1844	2
FGRAMPH1_01G02169	FGRAMPH1_01T02169	1.4276	4.15813	Promoter	1	2841773	2844574	2802	2
FGRAMPH1_01G02173	FGRAMPH1_01T02173	2.17531	18.0144	Promoter	1	2846394	2847230	837	1
ENSRNA049559431	ENSRNA049559431-T1	1.90113	11.67704	Promoter	1	2878814	2878885	72	2
FGRAMPH1_01G02199	FGRAMPH1_01T02199	1.56368	5.48089	Promoter	1	2879911	2881528	1618	1
FGRAMPH1_01G02207	FGRAMPH1_01T02207	2.06545	16.23389	Promoter	1	2888953	2891609	2657	2
FGRAMPH1_01G02209	FGRAMPH1_01T02209	1.49639	4.87899	Promoter	1	2892738	2892902	165	2
FGRAMPH1_01G02211	FGRAMPH1_01T02211	1.87293	12.27217	Exon	1	2893641	2894193	553	2
FGRAMPH1_01G02213	FGRAMPH1_01T02213	1.66671	7.76614	Promoter	1	2895238	2896742	1505	1
FGRAMPH1_01G02225	FGRAMPH1_01T02225	1.52086	5.01379	Intergenic	1	2914469	2914696	228	1
FGRAMPH1_01G02229	FGRAMPH1_01T02229	1.73241	8.44624	Intergenic	1	2918399	2918566	168	1
FGRAMPH1_01G02231	FGRAMPH1_01T02231	1.68647	9.75292	Promoter	1	2920707	2920943	237	1

FGRAMPH1_01G02297	FGRAMPH1_01T02297	1.64126	7.10047	Promoter	1	2997965	2999595	1631	2
FGRAMPH1_01G02317	FGRAMPH1_01T02317	1.69022	7.4724	Promoter	1	3028618	3031077	2460	1
FGRAMPH1_01G02331	FGRAMPH1_01T02331	2.18585	18.36041	Exon	1	3051992	3052362	371	2
FGRAMPH1_01G02343	FGRAMPH1_01T02343	1.65859	7.00562	Promoter	1	3064915	3065133	219	2
FGRAMPH1_01G02349	FGRAMPH1_01T02349	1.33139	3.21359	Promoter	1	3072436	3076394	3959	1
FGRAMPH1_01G02357	FGRAMPH1_01T02357	1.30885	3.0199	Promoter	1	3082372	3084033	1662	2
ENSRNA049558347	ENSRNA049558347-T1	2.71312	33.78359	Promoter	1	3084948	3085047	100	1
FGRAMPH1_01G02403	FGRAMPH1_01T02403	1.80622	9.73718	Promoter	1	3148920	3151862	2943	2
FGRAMPH1_01G02429	FGRAMPH1_01T02429	1.51858	7.69071	Promoter	1	3187728	3190181	2454	1
ENSRNA049558370	ENSRNA049558370-T1	2.35368	25.46886	Promoter	1	3192987	3193086	100	1
FGRAMPH1_01G02441	FGRAMPH1_01T02441	1.93917	15.47043	Promoter	1	3202435	3204204	1770	2
FGRAMPH1_01G02443	FGRAMPH1_01T02443	2.09701	18.64901	Exon	1	3206292	3206704	413	2
FGRAMPH1_01G02445	FGRAMPH1_01T02445	1.61883	9.83617	Promoter	1	3209262	3213267	4006	1

FGRAMPH1_01G02459	FGRAMPH1_01T02459	1.85565	12.11211	Intergenic	1	3235302	3235969	668	1
FGRAMPH1_01G02461	FGRAMPH1_01T02461	1.85224	11.2051	Promoter	1	3238230	3240119	1890	1
FGRAMPH1_01G02475	FGRAMPH1_01T02475	2.02995	16.5475	Promoter	1	3263501	3265347	1847	2
FGRAMPH1_01G02477	FGRAMPH1_01T02477	1.33008	3.16437	Promoter	1	3266972	3267997	1026	1
FGRAMPH1_01G02507	FGRAMPH1_01T02507	1.40904	4.15783	Promoter	1	3299505	3301856	2352	1
FGRAMPH1_01G02517	FGRAMPH1_01T02517	1.69348	8.06783	Promoter	1	3311346	3316105	4760	2
FGRAMPH1_01G02525	FGRAMPH1_01T02525	2.32293	24.31315	Promoter	1	3321320	3325231	3912	2
FGRAMPH1_01G02527	FGRAMPH1_01T02527	2.01248	17.08379	Promoter	1	3326163	3327475	1313	2
FGRAMPH1_01G02573	FGRAMPH1_01T02573	2.17985	21.03601	Promoter	1	3373552	3374678	1127	2
FGRAMPH1_01G02579	FGRAMPH1_01T02579	1.46764	5.47705	Promoter	1	3380527	3383420	2894	2
FGRAMPH1_01G02581	FGRAMPH1_01T02581	1.65166	7.9132	Promoter	1	3385227	3387241	2015	2
FGRAMPH1_01G02597	FGRAMPH1_01T02597	2.06937	16.30444	Promoter	1	3401597	3403412	1816	2
FGRAMPH1_01G02599	FGRAMPH1_01T02599	2.14694	19.65057	Promoter	1	3404593	3405795	1203	1

FGRAMPH1_01G02629	FGRAMPH1_01T02629	1.48701	5.36407	Promoter	1	3480355	3482505	2151	2
FGRAMPH1_01G02643	FGRAMPH1_01T02643	1.55329	6.23999	UTR	1	3495842	3498281	2440	1
FGRAMPH1_01G02667	FGRAMPH1_01T02667	1.37053	4.75638	Promoter	1	3528343	3529221	879	1
FGRAMPH1_01G02671	FGRAMPH1_01T02671	1.78874	10.67171	Promoter	1	3532801	3534067	1267	2
FGRAMPH1_01G02673	FGRAMPH1_01T02673	1.61403	6.41261	Promoter	1	3536514	3536846	333	2
FGRAMPH1_01G02695	FGRAMPH1_01T02695	1.63048	7.87017	Promoter	1	3564375	3567519	3145	2
FGRAMPH1_01G02707	FGRAMPH1_01T02707	1.58733	7.15882	Promoter	1	3587330	3590503	3174	2
ENSRNA049558394	ENSRNA049558394-T1	2.05931	15.34379	Promoter	1	3617936	3618007	72	1
FGRAMPH1_01G02745	FGRAMPH1_01T02745	1.76133	9.0164	Promoter	1	3633867	3636686	2820	1
FGRAMPH1_01G02755	FGRAMPH1_01T02755	1.67458	8.61953	Promoter	1	3646603	3646941	339	1
FGRAMPH1_01G02767	FGRAMPH1_01T02767	1.52657	5.74794	Promoter	1	3662768	3663228	461	2
FGRAMPH1_01G02771	FGRAMPH1_01T02771	1.47196	7.17866	Promoter	1	3668212	3670705	2494	1
FGRAMPH1_01G02777	FGRAMPH1_01T02777	1.78226	11.15141	Promoter	1	3678815	3680950	2136	2

FGRAMPH1_01G02783	FGRAMPH1_01T02783	1.59282	5.8676	Promoter	1	3684180	3685728	1549	2
FGRAMPH1_01G02829	FGRAMPH1_01T02829	2.00511	16.66456	Promoter	1	3729595	3731658	2064	2
FGRAMPH1_01G02833	FGRAMPH1_01T02833	1.55138	6.95461	Promoter	1	3732475	3735096	2622	2
FGRAMPH1_01G02839	FGRAMPH1_01T02839	1.60102	9.01073	Exon	1	3741354	3742386	1033	1
FGRAMPH1_01G02841	FGRAMPH1_01T02841	1.355	4.48793	UTR	1	3744931	3750030	5100	1
FGRAMPH1_01G02849	FGRAMPH1_01T02849	1.32422	3.59466	UTR	1	3757352	3757940	589	1
FGRAMPH1_01G02851	FGRAMPH1_01T02851	1.59532	5.89894	Promoter	1	3758475	3758874	400	1
FGRAMPH1_01G02871	FGRAMPH1_01T02871	1.72748	8.37218	Promoter	1	3781660	3782919	1260	2
FGRAMPH1_01G02875	FGRAMPH1_01T02875	1.68606	8.76694	Promoter	1	3787409	3790289	2881	2
FGRAMPH1_01G02877	FGRAMPH1_01T02877	2.40049	26.28749	Promoter	1	3793854	3794115	262	1
FGRAMPH1_01G02883	FGRAMPH1_01T02883	2.56352	29.03292	Promoter	1	3802892	3807304	4413	2
FGRAMPH1_01G02885	FGRAMPH1_01T02885	1.55822	5.6606	Promoter	1	3811809	3812269	461	2
FGRAMPH1_01G02893	FGRAMPH1_01T02893	1.61016	7.32518	Promoter	1	3825656	3830693	5038	1

FGRAMPH1_01G02915	FGRAMPH1_01T02915	1.76274	11.21295	Promoter	1	3855998	3858494	2497	2
FGRAMPH1_01G02919	FGRAMPH1_01T02919	1.70739	9.44325	Promoter	1	3859996	3862813	2818	2
FGRAMPH1_01G02921	FGRAMPH1_01T02921	1.92195	13.85451	Promoter	1	3864732	3865500	769	2
FGRAMPH1_01G02927	FGRAMPH1_01T02927	2.05614	17.54413	Promoter	1	3873303	3875169	1867	1
FGRAMPH1_01G02935	FGRAMPH1_01T02935	2.05498	17.52108	Promoter	1	3890458	3896157	5700	2
FGRAMPH1_01G02947	FGRAMPH1_01T02947	2.16706	19.0546	Promoter	1	3915885	3918601	2717	2
FGRAMPH1_01G02951	FGRAMPH1_01T02951	1.46227	4.59027	Promoter	1	3920359	3921360	1002	2
FGRAMPH1_01G02953	FGRAMPH1_01T02953	2.24912	20.12992	Exon	1	3922835	3923644	810	1
FGRAMPH1_01G02955	FGRAMPH1_01T02955	2.42277	26.54725	Promoter	1	3928285	3928990	706	1
FGRAMPH1_01G02969	FGRAMPH1_01T02969	1.86205	11.96853	Promoter	1	3954249	3956660	2412	1
FGRAMPH1_01G02979	FGRAMPH1_01T02979	1.63721	6.77357	Promoter	1	3963880	3969002	5123	2
FGRAMPH1_01G02985	FGRAMPH1_01T02985	1.77459	9.21114	Promoter	1	3978419	3979189	771	1
FGRAMPH1_01G03001	FGRAMPH1_01T03001	2.02218	15.04312	Promoter	1	4001970	4005248	3279	2

FGRAMPH1_01G03009	FGRAMPH1_01T03009	1.86422	11.39892	Promoter	1	4009748	4014597	4850	2
FGRAMPH1_01G03015	FGRAMPH1_01T03015	1.40556	4.55054	Promoter	1	4018001	4019953	1953	2
FGRAMPH1_01G03037	FGRAMPH1_01T03037	1.53743	6.33253	Promoter	1	4040179	4043643	3465	1
FGRAMPH1_01G03047	FGRAMPH1_01T03047	2.50221	27.42648	Intergenic	1	4049510	4049694	185	1
FGRAMPH1_01G03049	FGRAMPH1_01T03049	1.66913	7.23732	Promoter	1	4052922	4057324	4403	1
FGRAMPH1_01G03063	FGRAMPH1_01T03063	1.60586	6.11329	UTR	1	4076611	4077407	797	2
FGRAMPH1_01G03077	FGRAMPH1_01T03077	1.87434	11.25487	Promoter	1	4098489	4100289	1801	1
FGRAMPH1_01G03081	FGRAMPH1_01T03081	1.6371	7.68731	Promoter	1	4104042	4106184	2143	1
FGRAMPH1_01G03089	FGRAMPH1_01T03089	1.7535	8.69796	Intron	1	4113763	4114409	647	1
FGRAMPH1_01G03091	FGRAMPH1_01T03091	1.74295	8.44624	Intron	1	4115363	4119581	4219	1
FGRAMPH1_01G03101	FGRAMPH1_01T03101	2.00658	14.07517	Promoter	1	4137374	4138904	1531	2
FGRAMPH1_01G03103	FGRAMPH1_01T03103	1.48953	5.07957	Promoter	1	4140691	4140852	162	1
FGRAMPH1_01G03105	FGRAMPH1_01T03105	1.99604	13.76516	Promoter	1	4141080	4141589	510	2

FGRAMPH1_01G03107	FGRAMPH1_01T03107	2.4073	24.63614	Promoter	1	4143262	4143447	186	1
FGRAMPH1_01G03127	FGRAMPH1_01T03127	1.52265	5.03545	Promoter	1	4161140	4161427	288	2
FGRAMPH1_01G03155	FGRAMPH1_01T03155	1.64886	7.41681	Promoter	1	4201628	4203149	1522	2
FGRAMPH1_01G03157	FGRAMPH1_01T03157	1.90847	13.80756	Promoter	1	4207424	4208173	750	1
FGRAMPH1_01G03163	FGRAMPH1_01T03163	2.38806	25.12787	Promoter	1	4214333	4217373	3041	2
FGRAMPH1_01G03165	FGRAMPH1_01T03165	1.75734	9.00996	Intergenic	1	4222026	4222760	735	2
FGRAMPH1_01G03167	FGRAMPH1_01T03167	1.58477	5.89894	Intron	1	4222804	4224070	1267	2
FGRAMPH1_01G03169	FGRAMPH1_01T03169	1.9861	13.73363	Promoter	1	4225891	4227614	1724	1
FGRAMPH1_01G03191	FGRAMPH1_01T03191	1.37635	4.32169	Promoter	1	4255624	4257964	2341	2
FGRAMPH1_01G03193	FGRAMPH1_01T03193	1.66576	8.38632	Intergenic	1	4258972	4259250	279	1
ENSRNA049558408	ENSRNA049558408-T1	2.14864	19.40951	Promoter	1	4271987	4272069	83	1
FGRAMPH1_01G03209	FGRAMPH1_01T03209	1.85259	14.93778	Exon	1	4281084	4283117	2034	1
FGRAMPH1_01G03211	FGRAMPH1_01T03211	1.47471	4.89442	Exon	1	4283325	4283633	309	2

FGRAMPH1_01G03213	FGRAMPH1_01T03213	1.28424	3.54284	Promoter	1	4283873	4284763	891	2
FGRAMPH1_01G03215	FGRAMPH1_01T03215	1.85218	13.35508	Promoter	1	4287082	4288172	1091	1
FGRAMPH1_01G03221	FGRAMPH1_01T03221	1.50556	7.21988	Promoter	1	4299425	4301569	2145	2
ENSRNA049513860	ENSRNA049513860-T1	2.47057	26.21645	Promoter	1	4307639	4307758	120	2
FGRAMPH1_01G03231	FGRAMPH1_01T03231	1.40643	5.32559	Promoter	1	4314996	4317005	2010	2
FGRAMPH1_01G03237	FGRAMPH1_01T03237	1.80023	10.30651	Promoter	1	4327702	4330455	2754	1
FGRAMPH1_01G03239	FGRAMPH1_01T03239	1.47303	5.15367	Promoter	1	4332737	4333241	505	2
FGRAMPH1_01G03245	FGRAMPH1_01T03245	2.08429	20.50248	Promoter	1	4338632	4341836	3205	2
FGRAMPH1_01G03257	FGRAMPH1_01T03257	1.42921	5.25938	Promoter	1	4364941	4366732	1792	1
FGRAMPH1_01G03269	FGRAMPH1_01T03269	1.78346	11.43973	Promoter	1	4386102	4386341	240	1
FGRAMPH1_01G03275	FGRAMPH1_01T03275	2.63583	32.38525	Promoter	1	4391276	4393625	2350	2
FGRAMPH1_01G03277	FGRAMPH1_01T03277	1.33124	4.52045	Promoter	1	4397801	4400601	2801	1
FGRAMPH1_01G03283	FGRAMPH1_01T03283	1.71602	8.73401	Promoter	1	4406247	4407365	1119	2

FGRAMPH1_01G03307	FGRAMPH1_01T03307	1.72186	8.19781	Promoter	1	4444561	4450082	5522	2
FGRAMPH1_01G03319	FGRAMPH1_01T03319	1.72089	10.3852	Promoter	1	4462181	4462730	550	2
FGRAMPH1_01G03321	FGRAMPH1_01T03321	1.43138	5.3564	Promoter	1	4463193	4465665	2473	1
FGRAMPH1_01G03323	FGRAMPH1_01T03323	1.65367	7.99683	Promoter	1	4465910	4469786	3877	1
FGRAMPH1_01G03329	FGRAMPH1_01T03329	1.93264	13.25799	Promoter	1	4474934	4476775	1842	2
FGRAMPH1_01G03331	FGRAMPH1_01T03331	1.59596	8.67177	Intergenic	1	4479903	4480198	296	2
FGRAMPH1_01G03333	FGRAMPH1_01T03333	1.77459	9.21114	Promoter	1	4484635	4487643	3009	1
FGRAMPH1_01G03335	FGRAMPH1_01T03335	1.74983	9.25298	Promoter	1	4488402	4490320	1919	1
FGRAMPH1_01G03351	FGRAMPH1_01T03351	1.86949	11.10737	Promoter	1	4507113	4507850	738	2
FGRAMPH1_01G03355	FGRAMPH1_01T03355	1.68622	7.60263	Promoter	1	4509945	4510589	645	2
FGRAMPH1_01G03357	FGRAMPH1_01T03357	1.82778	10.63431	Promoter	1	4512063	4514632	2570	2
FGRAMPH1_01G03359	FGRAMPH1_01T03359	1.49628	4.45211	Promoter	1	4516138	4517786	1649	1
ENSRNA049558430	ENSRNA049558430-T1	2.81857	37.37431	Promoter	1	4585575	4585646	72	1

FGRAMPH1_01G03413	FGRAMPH1_01T03413	1.3463	3.79341	Promoter	1	4588910	4593794	4885	2
FGRAMPH1_01G03419	FGRAMPH1_01T03419	1.66913	7.23732	Promoter	1	4598593	4600127	1535	1
FGRAMPH1_01G03451	FGRAMPH1_01T03451	1.45954	5.48325	Promoter	1	4632212	4634216	2005	2
FGRAMPH1_01G03459	FGRAMPH1_01T03459	1.77142	9.95724	Promoter	1	4644660	4645882	1223	2
FGRAMPH1_01G03461	FGRAMPH1_01T03461	1.5248	6.62438	UTR	1	4648157	4649219	1063	1
FGRAMPH1_01G03471	FGRAMPH1_01T03471	2.13961	17.80405	Promoter	1	4662939	4663805	867	1
FGRAMPH1_01G03473	FGRAMPH1_01T03473	2.13872	17.94013	Promoter	1	4665268	4669047	3780	1
FGRAMPH1_01G03479	FGRAMPH1_01T03479	1.60241	7.55874	Promoter	1	4674450	4676062	1613	2
FGRAMPH1_01G03485	FGRAMPH1_01T03485	1.89058	11.39068	Exon	1	4681755	4682153	399	1
FGRAMPH1_01G03487	FGRAMPH1_01T03487	2.57603	29.48946	Promoter	1	4682114	4683292	1179	2
FGRAMPH1_01G03489	FGRAMPH1_01T03489	1.79568	9.47257	Intergenic	1	4684160	4684369	210	1
FGRAMPH1_01G03491	FGRAMPH1_01T03491	1.83018	12.63985	Promoter	1	4685417	4688287	2871	1
FGRAMPH1_01G03495	FGRAMPH1_01T03495	2.08562	16.5964	Promoter	1	4692886	4694890	2005	2

FGRAMPH1_01G03497	FGRAMPH1_01T03497	1.9476	14.11213	Promoter	1	4695773	4698267	2495	2
FGRAMPH1_01G03499	FGRAMPH1_01T03499	1.91336	12.92449	Promoter	1	4700761	4704574	3814	1
FGRAMPH1_01G03501	FGRAMPH1_01T03501	1.71609	10.62979	Exon	1	4710394	4712629	2236	2
FGRAMPH1_01G03503	FGRAMPH1_01T03503	1.66802	10.83771	Promoter	1	4712939	4713292	354	2
FGRAMPH1_01G03505	FGRAMPH1_01T03505	1.92718	17.98702	Exon	1	4717645	4721309	3665	1
FGRAMPH1_01G03507	FGRAMPH1_01T03507	1.86133	16.26229	Promoter	1	4721436	4723382	1947	1
FGRAMPH1_01G03523	FGRAMPH1_01T03523	1.81963	11.19765	Promoter	1	4751062	4759032	7971	2
FGRAMPH1_01G03525	FGRAMPH1_01T03525	1.73078	11.44321	Exon	1	4762220	4762694	475	2
FGRAMPH1_01G03527	FGRAMPH1_01T03527	1.68406	8.11428	UTR	1	4762754	4765408	2655	1
FGRAMPH1_01G03535	FGRAMPH1_01T03535	1.71451	10.22268	Promoter	1	4769808	4771773	1966	2
FGRAMPH1_01G03541	FGRAMPH1_01T03541	1.56111	9.1913	Promoter	1	4783822	4784591	770	1
FGRAMPH1_01G03543	FGRAMPH1_01T03543	1.62862	9.56132	Exon	1	4785278	4785553	276	2
FGRAMPH1_01G03545	FGRAMPH1_01T03545	1.65015	7.13825	Promoter	1	4785915	4788505	2591	1

FGRAMPH1_01G03551	FGRAMPH1_01T03551	1.67224	7.71876	Exon	1	4798376	4799082	707	2
FGRAMPH1_01G03557	FGRAMPH1_01T03557	1.76404	8.95294	Promoter	1	4810417	4814702	4286	1
FGRAMPH1_01G03565	FGRAMPH1_01T03565	1.72184	9.5906	Promoter	1	4820245	4822297	2053	1
FGRAMPH1_01G03567	FGRAMPH1_01T03567	1.5149	6.59689	Exon	1	4824906	4825673	768	1
FGRAMPH1_01G03577	FGRAMPH1_01T03577	2.25967	20.49161	Promoter	1	4838726	4839997	1272	1
FGRAMPH1_01G03609	FGRAMPH1_01T03609	1.60586	6.11329	UTR	1	4873922	4874476	555	2
ENSRNA049559400	ENSRNA049559400-T1	2.58657	29.90888	Promoter	1	4908448	4908529	82	2
ENSRNA049558473	ENSRNA049558473-T1	2.71312	33.78359	Promoter	1	4918760	4918857	98	1
FGRAMPH1_01G03667	FGRAMPH1_01T03667	1.7144	9.32484	Promoter	1	4954563	4957291	2729	2
FGRAMPH1_01G03675	FGRAMPH1_01T03675	1.61164	6.65719	Exon	1	4973019	4973570	552	1
FGRAMPH1_01G03729	FGRAMPH1_01T03729	1.92605	12.85702	Promoter	1	5043155	5046372	3218	2
FGRAMPH1_01G03731	FGRAMPH1_01T03731	1.50283	4.82435	Promoter	1	5048867	5052266	3400	1
ENSRNA049558520	ENSRNA049558520-T1	2.10149	16.32472	Promoter	1	5060261	5060358	98	1

ENSRNA049558582	ENSRNA049558582-T1	2.29131	21.22266	Promoter	1	5076016	5076087	72	1
FGRAMPH1_01G03777	FGRAMPH1_01T03777	1.37705	4.52605	Promoter	1	5109006	5110370	1365	1
FGRAMPH1_01G03793	FGRAMPH1_01T03793	2.04498	17.70494	Promoter	1	5130035	5132369	2335	1
FGRAMPH1_01G03795	FGRAMPH1_01T03795	1.81135	10.71544	Promoter	1	5132822	5134718	1897	1
FGRAMPH1_01G03813	FGRAMPH1_01T03813	1.35621	4.41904	Exon	1	5167352	5167979	628	2
FGRAMPH1_01G03821	FGRAMPH1_01T03821	1.66682	7.58829	Promoter	1	5179065	5180654	1590	2
ENSRNA049558613	ENSRNA049558613-T1	2.78693	36.46401	Promoter	1	5189527	5189612	86	1
FGRAMPH1_01G03851	FGRAMPH1_01T03851	1.89072	11.70814	Promoter	1	5230876	5231778	903	2
ENSRNA049559382	ENSRNA049559382-T1	2.4378	25.38465	Promoter	1	5235534	5235615	82	2
FGRAMPH1_01G03879	FGRAMPH1_01T03879	1.62482	6.47151	Promoter	1	5271617	5271916	300	1
ENSRNA049558644	ENSRNA049558644-T1	2.399	26.59171	Promoter	1	5308239	5308317	79	1
ENSRNA049559362	ENSRNA049559362-T1	2.47359	28.16583	Promoter	1	5329758	5329848	91	2
FGRAMPH1_01G03921	FGRAMPH1_01T03921	1.42406	4.64483	Promoter	1	5334744	5337791	3048	2

FGRAMPH1_01G03923	FGRAMPH1_01T03923	1.9644	13.15382	Promoter	1	5339445	5343104	3660	2
FGRAMPH1_01G03925	FGRAMPH1_01T03925	1.51705	6.48025	Promoter	1	5349144	5350849	1706	2
ENSRNA049513719	ENSRNA049513719-T1	2.10753	21.66285	Promoter	1	5365462	5365740	279	2
ENSRNA049559344	ENSRNA049559344-T1	2.53385	28.2448	Promoter	1	5379443	5379514	72	2
ENSRNA049558683	ENSRNA049558683-T1	2.21749	19.06036	Promoter	1	5382956	5383027	72	1
FGRAMPH1_01G03977	FGRAMPH1_01T03977	1.53294	5.2667	Promoter	1	5413645	5417734	4090	1
ENSRNA049559318	ENSRNA049559318-T1	2.09892	19.31872	Promoter	1	5421800	5421871	72	2
FGRAMPH1_01G03993	FGRAMPH1_01T03993	1.88105	13.06038	Exon	1	5441057	5441386	330	1
FGRAMPH1_01G03995	FGRAMPH1_01T03995	1.36696	3.37857	Promoter	1	5443917	5446683	2767	1
FGRAMPH1_01G04003	FGRAMPH1_01T04003	1.94853	15.31922	Promoter	1	5455599	5460655	5057	2
FGRAMPH1_01G04005	FGRAMPH1_01T04005	2.03133	16.47427	Promoter	1	5461973	5462414	442	2
ENSRNA049558709	ENSRNA049558709-T1	2.36987	25.99208	Promoter	1	5535113	5535184	72	1
FGRAMPH1_01G04057	FGRAMPH1_01T04057	1.71721	9.46218	Promoter	1	5540454	5541619	1166	2

FGRAMPH1_01G04061	FGRAMPH1_01T04061	1.38493	3.8448	Promoter	1	5545453	5546210	758	1
FGRAMPH1_01G04063	FGRAMPH1_01T04063	1.78513	9.21114	Promoter	1	5546977	5547990	1014	1
FGRAMPH1_01G04065	FGRAMPH1_01T04065	2.35458	23.09437	Promoter	1	5548316	5550775	2460	2
FGRAMPH1_01G04067	FGRAMPH1_01T04067	2.45351	26.62173	Exon	1	5553166	5553655	490	2
FGRAMPH1_01G04077	FGRAMPH1_01T04077	1.50041	4.49906	Promoter	1	5561112	5564372	3261	2
FGRAMPH1_01G04107	FGRAMPH1_01T04107	1.69022	7.4724	Promoter	1	5607045	5607965	921	1
FGRAMPH1_01G04141	FGRAMPH1_01T04141	1.73845	9.57235	Promoter	1	5646552	5648773	2222	1
FGRAMPH1_01G04147	FGRAMPH1_01T04147	1.85895	10.82712	Promoter	1	5653781	5654578	798	2
ENSRNA049559305	ENSRNA049559305-T1	2.47057	26.21645	Promoter	1	5660626	5660699	74	2
FGRAMPH1_01G04177	FGRAMPH1_01T04177	2.08718	16.99043	Promoter	1	5683012	5683975	964	2
FGRAMPH1_01G04219	FGRAMPH1_01T04219	1.54321	8.36352	Promoter	1	5731834	5735470	3637	1
FGRAMPH1_01G04241	FGRAMPH1_01T04241	1.88391	12.10374	Promoter	1	5766500	5769318	2819	2
FGRAMPH1_01G04249	FGRAMPH1_01T04249	2.11204	16.65722	Promoter	1	5776446	5777096	651	2

FGRAMPH1_01G04251	FGRAMPH1_01T04251	2.17833	19.83659	Promoter	1	5777725	5778750	1026	2
FGRAMPH1_01G04295	FGRAMPH1_01T04295	1.97531	14.21759	Promoter	1	5829478	5833356	3879	2
FGRAMPH1_01G04297	FGRAMPH1_01T04297	1.96559	13.47974	Exon	1	5834920	5835831	912	2
FGRAMPH1_01G04301	FGRAMPH1_01T04301	1.48297	5.44533	Promoter	1	5840481	5841557	1077	2
FGRAMPH1_01G04443	FGRAMPH1_01T04443	1.43563	6.38677	Promoter	1	6002353	6002808	456	2
FGRAMPH1_01G04447	FGRAMPH1_01T04447	1.48772	7.88093	Promoter	1	6012774	6013046	273	1
ENSRNA049558727	ENSRNA049558727-T1	2.68148	32.90722	Promoter	1	6029202	6029291	90	1
FGRAMPH1_01G04455	FGRAMPH1_01T04455	1.56368	5.48089	Promoter	1	6030475	6031254	780	2
FGRAMPH1_01G04459	FGRAMPH1_01T04459	1.86153	14.76452	Promoter	1	6036667	6036885	219	2
FGRAMPH1_01G04461	FGRAMPH1_01T04461	2.0848	19.85929	Promoter	1	6039273	6043225	3953	1
FGRAMPH1_01G04465	FGRAMPH1_01T04465	1.78471	11.24398	Promoter	1	6045516	6049624	4109	2
FGRAMPH1_01G04485	FGRAMPH1_01T04485	1.44809	5.11417	Promoter	1	6090307	6093283	2977	1
FGRAMPH1_01G04487	FGRAMPH1_01T04487	1.4478	5.12363	Promoter	1	6094169	6098214	4046	2

FGRAMPH1_01G04489	FGRAMPH1_01T04489	1.87361	14.64586	Exon	1	6100715	6101474	760	2
FGRAMPH1_01G04491	FGRAMPH1_01T04491	1.57759	6.21473	Promoter	1	6109860	6113853	3994	2
FGRAMPH1_01G04499	FGRAMPH1_01T04499	1.92995	14.49656	Promoter	1	6119586	6123138	3553	2
FGRAMPH1_01G04523	FGRAMPH1_01T04523	1.68074	7.7896	Promoter	1	6155579	6157429	1851	2
FGRAMPH1_01G04525	FGRAMPH1_01T04525	1.81677	10.00496	Promoter	1	6161127	6163090	1964	2
FGRAMPH1_01G04533	FGRAMPH1_01T04533	1.80168	10.38897	Promoter	1	6172333	6174897	2565	2
FGRAMPH1_01G04543	FGRAMPH1_01T04543	1.54059	7.30219	Promoter	1	6187411	6189097	1687	2
FGRAMPH1_01G04545	FGRAMPH1_01T04545	2.08329	19.02747	Exon	1	6189660	6193274	3615	2
FGRAMPH1_01G04547	FGRAMPH1_01T04547	2.57603	29.48946	Exon	1	6201066	6201605	540	1
FGRAMPH1_01G04555	FGRAMPH1_01T04555	1.98948	14.50784	Promoter	1	6215770	6217116	1347	2
FGRAMPH1_01G04573	FGRAMPH1_01T04573	1.58044	6.51199	Promoter	1	6235016	6236910	1895	1
FGRAMPH1_01G04621	FGRAMPH1_01T04621	2.35995	26.44331	Intergenic	1	6299067	6299291	225	2
FGRAMPH1_01G04623	FGRAMPH1_01T04623	1.93255	13.67224	Intergenic	1	6302823	6303722	900	2

FGRAMPH1_01G04627	FGRAMPH1_01T04627	2.0836	18.99863	UTR	1	6306179	6306724	546	2
FGRAMPH1_01G04671	FGRAMPH1_01T04671	1.97072	13.91679	Promoter	1	6377511	6381077	3567	1
FGRAMPH1_01G04717	FGRAMPH1_01T04717	1.6763	8.03738	Promoter	1	6446786	6450097	3312	2
FGRAMPH1_01G04721	FGRAMPH1_01T04721	1.36974	4.17418	Promoter	1	6453292	6455140	1849	1
FGRAMPH1_01G04729	FGRAMPH1_01T04729	1.62695	6.55252	Promoter	1	6458963	6461657	2695	2
FGRAMPH1_01G04731	FGRAMPH1_01T04731	2.29131	21.22266	Promoter	1	6464647	6465483	837	1
FGRAMPH1_01G04739	FGRAMPH1_01T04739	2.20885	22.38526	Promoter	1	6473196	6479535	6340	2
FGRAMPH1_01G04741	FGRAMPH1_01T04741	1.99111	15.89247	Promoter	1	6482911	6489329	6419	1
FGRAMPH1_01G04743	FGRAMPH1_01T04743	1.51982	4.96332	Promoter	1	6489550	6493055	3506	2
FGRAMPH1_01G04745	FGRAMPH1_01T04745	1.50556	7.21988	Promoter	1	6496241	6497926	1686	1
FGRAMPH1_01G04761	FGRAMPH1_01T04761	2.13065	18.48234	Promoter	1	6514217	6520729	6513	2
FGRAMPH1_01G04773	FGRAMPH1_01T04773	1.98272	15.19131	Promoter	1	6533499	6536396	2898	1
ENSRNA049559291	ENSRNA049559291-T1	2.33349	22.3382	Promoter	1	6547261	6547353	93	2

FGRAMPH1_01G04787	FGRAMPH1_01T04787	1.57973	6.75921	UTR	1	6554761	6556733	1973	2
FGRAMPH1_01G04801	FGRAMPH1_01T04801	1.91167	11.96643	Promoter	1	6571170	6572480	1311	2
FGRAMPH1_01G04805	FGRAMPH1_01T04805	2.39676	24.24705	Promoter	1	6576961	6579510	2550	1
ENSRNA049558742	ENSRNA049558742-T1	2.60766	30.33056	Promoter	1	6601083	6601172	90	1
FGRAMPH1_01G04833	FGRAMPH1_01T04833	1.5258	5.6503	Promoter	1	6612926	6613207	282	1
FGRAMPH1_01G04835	FGRAMPH1_01T04835	2.01558	14.36208	Promoter	1	6613528	6613977	450	2
ENSRNA049559273	ENSRNA049559273-T1	2.50221	27.42648	Promoter	1	6628148	6628237	90	2
FGRAMPH1_01G04849	FGRAMPH1_01T04849	1.67968	7.4724	Promoter	1	6635276	6637431	2156	2
ENSRNA049559250	ENSRNA049559250-T1	2.76584	35.56207	Promoter	1	6667478	6667580	103	2
ENSRNA049558754	ENSRNA049558754-T1	2.40557	24.75462	Promoter	1	6671766	6671855	90	1
ENSRNA049559232	ENSRNA049559232-T1	2.55494	28.6574	Promoter	1	6672529	6672625	97	2
FGRAMPH1_01G04879	FGRAMPH1_01T04879	1.64804	6.77735	Promoter	1	6673088	6675482	2395	2
FGRAMPH1_01G04885	FGRAMPH1_01T04885	1.30772	3.24596	Promoter	1	6679428	6681794	2367	2

FGRAMPH1_01G04887	FGRAMPH1_01T04887	1.57729	5.80429	Exon	1	6682612	6683084	473	2
ENSRNA049558781	ENSRNA049558781-T1	2.01888	16.3343	Promoter	1	6698401	6698490	90	1
ENSRNA049558802	ENSRNA049558802-T1	2.59712	30.33056	Promoter	1	6726660	6726749	90	1
FGRAMPH1_01G04933	FGRAMPH1_01T04933	1.70929	11.85201	Promoter	1	6736806	6740160	3355	2
FGRAMPH1_01G04945	FGRAMPH1_01T04945	1.31755	3.53398	Promoter	1	6759317	6760483	1167	2
FGRAMPH1_01G04959	FGRAMPH1_01T04959	1.83529	11.04384	Promoter	1	6774830	6776715	1886	2
FGRAMPH1_01G05001	FGRAMPH1_01T05001	1.83655	10.66032	Promoter	1	6819690	6822467	2778	1
FGRAMPH1_01G05005	FGRAMPH1_01T05005	2.02767	14.38806	Promoter	1	6824629	6826229	1601	2
FGRAMPH1_01G05007	FGRAMPH1_01T05007	1.84648	11.83745	Promoter	1	6826761	6827546	786	1
FGRAMPH1_01G05009	FGRAMPH1_01T05009	2.21749	19.06036	Promoter	1	6829917	6831410	1494	1
FGRAMPH1_01G05023	FGRAMPH1_01T05023	1.95178	12.90481	Promoter	1	6848530	6850696	2167	1
FGRAMPH1_01G05025	FGRAMPH1_01T05025	1.74549	11.93876	UTR	1	6851043	6853922	2880	2
FGRAMPH1_01G05027	FGRAMPH1_01T05027	2.33349	22.3382	Promoter	1	6855631	6856884	1254	2

FGRAMPH1_01G05029	FGRAMPH1_01T05029	2.32312	24.47108	Promoter	1	6859820	6859999	180	1
FGRAMPH1_01G05031	FGRAMPH1_01T05031	1.73241	8.44624	Exon	1	6862390	6863748	1359	1
FGRAMPH1_01G05033	FGRAMPH1_01T05033	1.59101	8.02827	Exon	1	6864667	6865301	635	2
FGRAMPH1_01G05035	FGRAMPH1_01T05035	1.689	7.73436	Promoter	1	6866976	6868729	1754	1
FGRAMPH1_01G05037	FGRAMPH1_01T05037	2.32294	21.96385	Promoter	1	6870550	6875592	5043	1
FGRAMPH1_01G05101	FGRAMPH1_01T05101	1.47953	6.57802	Promoter	1	6942009	6943631	1623	1
ENSRNA049558817	ENSRNA049558817-T1	2.01766	17.65681	Promoter	1	6957180	6957261	82	1
FGRAMPH1_01G05125	FGRAMPH1_01T05125	1.79073	9.99115	Promoter	1	6962222	6964029	1808	2
FGRAMPH1_01G05149	FGRAMPH1_01T05149	1.6448	8.34086	Promoter	1	6998283	6999700	1418	2
FGRAMPH1_01G05151	FGRAMPH1_01T05151	1.69022	7.4724	Promoter	1	7000573	7002278	1706	1
FGRAMPH1_01G05165	FGRAMPH1_01T05165	1.81341	12.10327	Promoter	1	7018177	7018696	520	1
FGRAMPH1_01G05167	FGRAMPH1_01T05167	1.64142	7.63461	Promoter	1	7019348	7021129	1782	1
FGRAMPH1_01G05195	FGRAMPH1_01T05195	1.61581	7.24671	Promoter	1	7048971	7051532	2562	2

FGRAMPH1_01G05209	FGRAMPH1_01T05209	2.16476	18.0144	Intergenic	1	7067124	7067566	443	2
ENSRNA049558825	ENSRNA049558825-T1	2.13313	16.99246	Promoter	1	7088560	7088655	96	1
FGRAMPH1_01G05305	FGRAMPH1_01T05305	1.86949	11.10737	Promoter	1	7161797	7163179	1383	1
FGRAMPH1_01G05365	FGRAMPH1_01T05365	1.66361	8.70717	Promoter	1	7219316	7219708	393	1
FGRAMPH1_01G05373	FGRAMPH1_01T05373	2.0095	14.21998	Promoter	1	7228718	7230918	2201	2
FGRAMPH1_01G05387	FGRAMPH1_01T05387	1.68249	7.72329	Promoter	1	7247928	7250715	2788	1
FGRAMPH1_01G05395	FGRAMPH1_01T05395	1.57185	6.11264	Promoter	1	7264428	7265066	639	1
FGRAMPH1_01G05435	FGRAMPH1_01T05435	1.8484	10.54995	Promoter	1	7318691	7319697	1007	1
FGRAMPH1_01G05437	FGRAMPH1_01T05437	1.53204	5.0772	Promoter	1	7319794	7320585	792	2
FGRAMPH1_01G05457	FGRAMPH1_01T05457	1.77309	10.24709	Promoter	1	7336796	7337575	780	2
FGRAMPH1_01G05465	FGRAMPH1_01T05465	1.69955	9.67764	Promoter	1	7346080	7347114	1035	1
FGRAMPH1_01G05467	FGRAMPH1_01T05467	2.12281	20.53473	Promoter	1	7347180	7351949	4770	1
FGRAMPH1_01G05483	FGRAMPH1_01T05483	2.29737	23.63281	Promoter	1	7365689	7367350	1662	1

FGRAMPH1_01G05485	FGRAMPH1_01T05485	2.1964	18.70907	Promoter	1	7367714	7369642	1929	1
FGRAMPH1_01G05505	FGRAMPH1_01T05505	1.44768	3.95439	Exon	1	7388319	7390501	2183	1
FGRAMPH1_01G05507	FGRAMPH1_01T05507	1.72342	11.1989	Promoter	1	7391378	7392718	1341	2
FGRAMPH1_01G05521	FGRAMPH1_01T05521	1.63587	8.9502	Promoter	1	7407855	7413845	5991	2
FGRAMPH1_01G05523	FGRAMPH1_01T05523	2.48112	26.61745	Promoter	1	7415996	7417231	1236	1
FGRAMPH1_01G05525	FGRAMPH1_01T05525	1.95386	12.85253	Promoter	1	7417544	7419331	1788	1
FGRAMPH1_01G05577	FGRAMPH1_01T05577	1.91167	11.96643	Promoter	1	7500705	7505942	5238	1
FGRAMPH1_01G05585	FGRAMPH1_01T05585	2.28753	22.94026	Promoter	1	7513514	7514710	1197	2
FGRAMPH1_01G05587	FGRAMPH1_01T05587	2.67565	36.05865	Promoter	1	7516008	7517824	1817	1
FGRAMPH1_01G05589	FGRAMPH1_01T05589	2.19071	22.86008	Promoter	1	7518141	7520294	2154	2
FGRAMPH1_01G05615	FGRAMPH1_01T05615	1.58477	5.89894	Promoter	1	7550432	7551856	1425	2
FGRAMPH1_01G05667	FGRAMPH1_01T05667	1.78513	9.21114	Promoter	1	7619696	7621577	1882	1
ENSRNA049558847	ENSRNA049558847-T1	2.34403	22.71505	Promoter	1	7652706	7652795	90	1

ENSRNA049513877	ENSRNA049513877-T1	2.84802	38.24644	Promoter	1	7659562	7659681	120	1
FGRAMPH1_01G05759	FGRAMPH1_01T05759	1.92584	14.66658	Promoter	1	7738630	7743072	4443	2
ENSRNA049559211	ENSRNA049559211-T1	2.64984	31.60897	Promoter	1	7747060	7747158	99	2
FGRAMPH1_01G05767	FGRAMPH1_01T05767	2.1013	20.21791	Promoter	1	7753825	7755852	2028	2
FGRAMPH1_01G05769	FGRAMPH1_01T05769	2.18606	19.67091	Promoter	1	7758194	7758559	366	1
FGRAMPH1_01G05779	FGRAMPH1_01T05779	1.97495	13.45803	Promoter	1	7768041	7769735	1695	1
FGRAMPH1_01G05801	FGRAMPH1_01T05801	1.8484	10.54995	Promoter	1	7795850	7797090	1241	1
FGRAMPH1_01G05809	FGRAMPH1_01T05809	1.96654	14.32316	Intergenic	1	7804071	7804570	500	2
FGRAMPH1_01G05853	FGRAMPH1_01T05853	1.70439	9.06521	Promoter	1	7856042	7858029	1988	1
ENSRNA049559170	ENSRNA049559170-T1	2.02663	17.33224	Promoter	1	7858464	7858537	74	2
FGRAMPH1_01G05869	FGRAMPH1_01T05869	1.51095	4.68809	Promoter	1	7875956	7876191	236	2
FGRAMPH1_01G05913	FGRAMPH1_01T05913	1.47252	5.91321	Promoter	1	7924350	7926264	1915	2
FGRAMPH1_01G05915	FGRAMPH1_01T05915	1.6956	8.42204	Promoter	1	7928129	7929919	1791	1

FGRAMPH1_01G05929	FGRAMPH1_01T05929	1.82004	11.42521	Intergenic	1	7949263	7949484	222	1
FGRAMPH1_01G05933	FGRAMPH1_01T05933	2.09592	16.63402	Promoter	1	7958426	7959076	651	1
FGRAMPH1_01G05937	FGRAMPH1_01T05937	1.61714	7.15407	Promoter	1	7965644	7966998	1355	2
FGRAMPH1_01G05955	FGRAMPH1_01T05955	1.598	8.18019	Promoter	1	7983424	7989341	5918	1
FGRAMPH1_01G05971	FGRAMPH1_01T05971	1.74069	9.55966	Promoter	1	8008221	8009654	1434	2
FGRAMPH1_01G05973	FGRAMPH1_01T05973	2.25412	23.96943	Promoter	1	8013876	8014287	412	1
FGRAMPH1_01G05975	FGRAMPH1_01T05975	1.6914	11.05622	Promoter	1	8015027	8015899	873	2
FGRAMPH1_01G05977	FGRAMPH1_01T05977	1.72022	8.50729	Promoter	1	8017409	8018779	1371	2
FGRAMPH1_01G05981	FGRAMPH1_01T05981	2.24334	21.26967	Promoter	1	8027401	8030073	2673	1
FGRAMPH1_01G06001	FGRAMPH1_01T06001	1.37329	4.85058	Promoter	1	8046753	8049515	2763	2
FGRAMPH1_01G06003	FGRAMPH1_01T06003	1.41397	4.67802	Promoter	1	8049583	8050495	913	2
FGRAMPH1_01G06009	FGRAMPH1_01T06009	1.86949	11.10737	Promoter	1	8055062	8056512	1451	1
FGRAMPH1_01G06011	FGRAMPH1_01T06011	1.57866	6.2832	Promoter	1	8058318	8060144	1827	1

FGRAMPH1_01G06031	FGRAMPH1_01T06031	1.78781	10.32915	Promoter	1	8082514	8082759	246	2
FGRAMPH1_01G06033	FGRAMPH1_01T06033	1.68693	11.46146	Promoter	1	8083958	8086089	2132	1
FGRAMPH1_01G06035	FGRAMPH1_01T06035	1.72819	11.19972	Exon	1	8087874	8088855	982	2
FGRAMPH1_01G06037	FGRAMPH1_01T06037	1.36875	4.909	Promoter	1	8090074	8092485	2412	1
FGRAMPH1_01G06039	FGRAMPH1_01T06039	1.3223	4.06333	UTR	1	8093521	8098027	4507	2
FGRAMPH1_01G06041	FGRAMPH1_01T06041	1.91064	16.34281	Promoter	1	8097774	8098171	398	2
FGRAMPH1_01G06051	FGRAMPH1_01T06051	1.41389	3.76107	Promoter	1	8112453	8112653	201	1
FGRAMPH1_01G06061	FGRAMPH1_01T06061	1.3421	3.39624	Promoter	1	8125169	8127724	2556	2
FGRAMPH1_01G06063	FGRAMPH1_01T06063	2.22675	26.44012	Promoter	1	8128128	8129043	916	2
FGRAMPH1_01G06065	FGRAMPH1_01T06065	2.23983	21.80801	Promoter	1	8129412	8133090	3679	2
FGRAMPH1_01G06073	FGRAMPH1_01T06073	1.55484	8.08125	Promoter	1	8141841	8146254	4414	2
FGRAMPH1_01G06075	FGRAMPH1_01T06075	2.77639	36.01199	Promoter	1	8149328	8149732	405	1
FGRAMPH1_01G06077	FGRAMPH1_01T06077	1.60586	6.11329	Promoter	1	8150462	8152535	2074	1

FGRAMPH1_01G06103	FGRAMPH1_01T06103	1.4882	5.16247	Exon	1	8181964	8183621	1658	1
FGRAMPH1_01G06105	FGRAMPH1_01T06105	2.01693	16.76481	UTR	1	8183621	8184759	1139	2
FGRAMPH1_01G06111	FGRAMPH1_01T06111	1.67229	7.62891	Promoter	1	8189937	8190938	1002	2
FGRAMPH1_01G06125	FGRAMPH1_01T06125	1.56743	5.85534	Intergenic	1	8206967	8207317	351	1
FGRAMPH1_01G06127	FGRAMPH1_01T06127	1.98408	15.30821	Promoter	1	8208828	8212156	3329	2
FGRAMPH1_01G06147	FGRAMPH1_01T06147	1.29705	3.08628	Promoter	1	8239202	8242724	3523	2
FGRAMPH1_01G06163	FGRAMPH1_01T06163	1.96908	14.49917	Promoter	1	8265350	8269652	4303	1
FGRAMPH1_01G06173	FGRAMPH1_01T06173	1.43115	4.56962	Promoter	1	8280189	8289602	9414	1
FGRAMPH1_01G06175	FGRAMPH1_01T06175	1.93277	12.25882	Promoter	1	8289629	8290768	1140	2
FGRAMPH1_01G06179	FGRAMPH1_01T06179	2.44525	28.30344	Promoter	1	8295553	8297564	2012	1
FGRAMPH1_01G06183	FGRAMPH1_01T06183	1.52657	5.74794	Promoter	1	8301564	8303363	1800	2
FGRAMPH1_01G06219	FGRAMPH1_01T06219	1.9276	15.36192	Promoter	1	8340796	8343703	2908	1
FGRAMPH1_01G06223	FGRAMPH1_01T06223	1.7431	8.74928	Promoter	1	8347781	8348809	1029	2

ENSRNA049559157	ENSRNA049559157-T1	2.13494	18.17496	Promoter	1	8350815	8350897	83	2
ENSRNA049513814	ENSRNA049513814-T1	2.01218	15.50797	Promoter	1	8355771	8355890	120	2
FGRAMPH1_01G06235	FGRAMPH1_01T06235	1.58203	6.01693	Promoter	1	8363824	8367866	4043	1
FGRAMPH1_01G06239	FGRAMPH1_01T06239	1.50464	6.42494	Promoter	1	8370624	8371957	1334	2
FGRAMPH1_01G06261	FGRAMPH1_01T06261	1.62545	7.44538	Promoter	1	8390942	8392262	1321	1
FGRAMPH1_01G06293	FGRAMPH1_01T06293	2.15002	18.42384	Promoter	1	8441948	8443332	1385	1
FGRAMPH1_01G06303	FGRAMPH1_01T06303	2.08206	17.15039	Promoter	1	8452555	8454584	2030	1
FGRAMPH1_01G06305	FGRAMPH1_01T06305	1.56931	7.42414	Promoter	1	8454312	8455613	1302	2
FGRAMPH1_01G06309	FGRAMPH1_01T06309	1.60353	9.83946	Promoter	1	8461042	8461293	252	1
FGRAMPH1_01G06311	FGRAMPH1_01T06311	1.44457	4.44718	Promoter	1	8462960	8465084	2125	1
FGRAMPH1_01G06313	FGRAMPH1_01T06313	1.77694	10.57572	Promoter	1	8465582	8466257	676	2
FGRAMPH1_01G06315	FGRAMPH1_01T06315	1.45105	5.43257	Promoter	1	8469300	8470335	1036	1
FGRAMPH1_01G06323	FGRAMPH1_01T06323	1.80569	10.17605	Promoter	1	8482431	8485585	3155	2

FGRAMPH1_01G06327	FGRAMPH1_01T06327	1.48665	5.78089	UTR	1	8488106	8492817	4712	1
FGRAMPH1_01G06339	FGRAMPH1_01T06339	2.05931	15.34379	Promoter	1	8503580	8505616	2037	2
FGRAMPH1_01G06341	FGRAMPH1_01T06341	1.74841	13.8029	Promoter	1	8508673	8509656	984	1
FGRAMPH1_01G06343	FGRAMPH1_01T06343	1.77459	9.21114	Promoter	1	8510259	8513156	2898	2
FGRAMPH1_01G06347	FGRAMPH1_01T06347	1.73639	9.48969	Promoter	1	8518546	8521551	3006	1
FGRAMPH1_01G06349	FGRAMPH1_01T06349	1.60448	6.39605	Promoter	1	8521156	8524833	3678	2
FGRAMPH1_01G06353	FGRAMPH1_01T06353	1.5624	5.56293	Promoter	1	8526255	8528094	1840	2
FGRAMPH1_01G06419	FGRAMPH1_01T06419	1.84968	10.8667	Promoter	1	8607051	8608436	1386	2
ENSRNA049559140	ENSRNA049559140-T1	2.17531	18.0144	Promoter	1	8626076	8626181	106	2
FGRAMPH1_01G06439	FGRAMPH1_01T06439	1.71838	8.62779	Promoter	1	8626471	8628321	1851	2
FGRAMPH1_01G06457	FGRAMPH1_01T06457	2.56548	29.0723	Exon	1	8647078	8648280	1203	2
FGRAMPH1_01G06461	FGRAMPH1_01T06461	1.44969	6.49126	Promoter	1	8651402	8653118	1717	2
FGRAMPH1_01G06465	FGRAMPH1_01T06465	1.9124	12.78528	Promoter	1	8654851	8657024	2174	2

FGRAMPH1_01G06469	FGRAMPH1_01T06469	1.70793	9.35722	Promoter	1	8660410	8662388	1979	2
FGRAMPH1_01G06471	FGRAMPH1_01T06471	1.31528	3.49493	UTR	1	8662827	8668100	5274	2
FGRAMPH1_01G06475	FGRAMPH1_01T06475	1.77658	11.88889	Exon	1	8668272	8668927	656	1
FGRAMPH1_01G06477	FGRAMPH1_01T06477	2.31767	22.36812	Promoter	1	8670067	8672225	2159	1
FGRAMPH1_01G06521	FGRAMPH1_01T06521	1.80622	9.73718	Promoter	1	8719870	8723122	3253	1
FGRAMPH1_01G06523	FGRAMPH1_01T06523	2.11204	16.65722	Promoter	1	8724989	8727973	2985	1
FGRAMPH1_01G06545	FGRAMPH1_01T06545	2.07888	16.72863	Intron	1	8742692	8745345	2654	2
FGRAMPH1_01G06551	FGRAMPH1_01T06551	1.58905	6.24238	Exon	1	8755147	8755650	504	2
FGRAMPH1_01G06555	FGRAMPH1_01T06555	1.3794	4.41672	Promoter	1	8758743	8762671	3929	2
FGRAMPH1_01G06569	FGRAMPH1_01T06569	1.7576	9.16827	Promoter	1	8789774	8790814	1041	1
FGRAMPH1_01G06571	FGRAMPH1_01T06571	1.68622	7.60263	Promoter	1	8791426	8793790	2365	1
FGRAMPH1_01G06577	FGRAMPH1_01T06577	1.93277	12.25882	Exon	1	8802837	8803205	369	1
FGRAMPH1_01G06587	FGRAMPH1_01T06587	1.3241	3.40442	Promoter	1	8809902	8814698	4797	2

FGRAMPH1_01G06589	FGRAMPH1_01T06589	1.94573	14.45945	Exon	1	8815448	8816210	763	1
FGRAMPH1_01G06591	FGRAMPH1_01T06591	1.90113	11.67704	Promoter	1	8825180	8825791	612	1
FGRAMPH1_01G06621	FGRAMPH1_01T06621	1.63193	8.24336	UTR	1	8873194	8874810	1617	2
FGRAMPH1_01G06625	FGRAMPH1_01T06625	1.93972	16.65548	Promoter	1	8875562	8876909	1348	1
ENSRNA049514005	ENSRNA049514005-T1	1.52568	5.57826	UTR	1	8882926	8883022	97	1
ENSRNA049559110	ENSRNA049559110-T1	2.39315	26.65178	Promoter	1	8884289	8884370	82	2
FGRAMPH1_01G06635	FGRAMPH1_01T06635	1.52626	5.25368	Promoter	1	8884823	8886873	2051	2
ENSRNA049559092	ENSRNA049559092-T1	2.07118	18.73875	Promoter	1	8902247	8902341	95	2
FGRAMPH1_01G06647	FGRAMPH1_01T06647	1.51189	6.62881	Intron	1	8903168	8908213	5046	1
FGRAMPH1_01G06655	FGRAMPH1_01T06655	1.63703	7.04111	Promoter	1	8912735	8913793	1059	1
FGRAMPH1_01G06665	FGRAMPH1_01T06665	1.25091	3.0025	Promoter	1	8923407	8925311	1905	2
ENSRNA049513904	ENSRNA049513904-T1	2.61821	30.75447	Promoter	1	8927397	8927516	120	1
FGRAMPH1_01G06671	FGRAMPH1_01T06671	1.54463	6.23999	Promoter	1	8931448	8933209	1762	1

FGRAMPH1_01G06675	FGRAMPH1_01T06675	1.87814	11.934	Promoter	1	8937281	8938657	1377	1
FGRAMPH1_01G06677	FGRAMPH1_01T06677	2.11783	17.97107	Promoter	1	8939759	8943176	3418	1
FGRAMPH1_01G06733	FGRAMPH1_01T06733	2.09094	15.99497	Promoter	1	9075423	9078650	3228	2
FGRAMPH1_01G06753	FGRAMPH1_01T06753	1.97917	17.3865	Promoter	1	9093024	9093353	330	2
FGRAMPH1_01G06759	FGRAMPH1_01T06759	1.8976	13.48446	Promoter	1	9100760	9104299	3540	1
FGRAMPH1_01G06761	FGRAMPH1_01T06761	1.46767	4.87295	Promoter	1	9104580	9106049	1470	2
FGRAMPH1_01G06769	FGRAMPH1_01T06769	1.40744	5.45658	Promoter	1	9119835	9120307	473	2
FGRAMPH1_01G06805	FGRAMPH1_01T06805	1.80943	10.79291	Intergenic	1	9165224	9165653	430	2
FGRAMPH1_01G06811	FGRAMPH1_01T06811	1.48074	6.18797	Promoter	1	9168142	9172106	3965	2
FGRAMPH1_01G06819	FGRAMPH1_01T06819	1.79735	12.07641	Promoter	1	9184440	9185879	1440	1
FGRAMPH1_01G06821	FGRAMPH1_01T06821	1.54393	5.84688	Promoter	1	9186503	9188826	2324	1
FGRAMPH1_01G06823	FGRAMPH1_01T06823	1.59695	8.42604	Exon	1	9191221	9191419	199	2
FGRAMPH1_01G06825	FGRAMPH1_01T06825	1.81972	10.28563	Promoter	1	9193016	9197082	4067	1

FGRAMPH1_01G06831	FGRAMPH1_01T06831	1.53766	6.47929	Promoter	1	9204155	9209931	5777	1
FGRAMPH1_01G06839	FGRAMPH1_01T06839	1.97964	13.36197	UTR	1	9215800	9219613	3814	2
FGRAMPH1_01G06841	FGRAMPH1_01T06841	1.47509	5.44533	Promoter	1	9219440	9219920	481	2
FGRAMPH1_01G06843	FGRAMPH1_01T06843	2.13862	17.24136	Promoter	1	9220846	9222103	1258	1
FGRAMPH1_01G06855	FGRAMPH1_01T06855	1.54966	6.26027	Promoter	1	9235870	9238051	2182	2
FGRAMPH1_01G06867	FGRAMPH1_01T06867	1.68807	8.34812	Promoter	1	9252194	9254506	2313	2
FGRAMPH1_01G06887	FGRAMPH1_01T06887	1.50041	4.49906	Promoter	1	9287247	9287438	192	1
FGRAMPH1_01G06893	FGRAMPH1_01T06893	1.6041	6.28153	Promoter	1	9298569	9302288	3720	1
FGRAMPH1_01G06895	FGRAMPH1_01T06895	2.12258	16.65722	Promoter	1	9301640	9304754	3115	2
FGRAMPH1_01G06897	FGRAMPH1_01T06897	1.7535	8.69796	Promoter	1	9305989	9306264	276	1
FGRAMPH1_01G06899	FGRAMPH1_01T06899	1.70077	7.71086	Promoter	1	9309487	9310743	1257	2
FGRAMPH1_01G06903	FGRAMPH1_01T06903	1.44196	4.52458	Promoter	1	9313872	9316809	2938	1
FGRAMPH1_01G06905	FGRAMPH1_01T06905	1.43857	3.99536	Exon	1	9317363	9318280	918	2

FGRAMPH1_01G06907	FGRAMPH1_01T06907	1.70636	11.36835	Promoter	1	9318672	9319614	943	1
FGRAMPH1_01G06917	FGRAMPH1_01T06917	1.55391	6.92239	Promoter	1	9330824	9332082	1259	2
FGRAMPH1_01G06935	FGRAMPH1_01T06935	1.74375	11.85342	Promoter	1	9347013	9348326	1314	1
FGRAMPH1_01G06951	FGRAMPH1_01T06951	1.34256	3.70013	Promoter	1	9369444	9370074	631	2
FGRAMPH1_01G06977	FGRAMPH1_01T06977	1.73934	9.34011	Promoter	1	9400905	9403136	2232	2
FGRAMPH1_01G06993	FGRAMPH1_01T06993	1.66913	7.23732	Promoter	1	9427513	9428388	876	1
FGRAMPH1_01G06995	FGRAMPH1_01T06995	1.86223	11.48321	Promoter	1	9428524	9431107	2584	1
FGRAMPH1_01G07001	FGRAMPH1_01T07001	1.66716	7.5182	Promoter	1	9437623	9437880	258	2
FGRAMPH1_01G07009	FGRAMPH1_01T07009	1.50807	6.87548	UTR	1	9451802	9452329	528	2
FGRAMPH1_01G07011	FGRAMPH1_01T07011	1.83925	10.52342	Promoter	1	9459488	9459853	366	1
FGRAMPH1_01G07045	FGRAMPH1_01T07045	1.64804	6.77735	Promoter	1	9501560	9504327	2768	2
FGRAMPH1_01G07051	FGRAMPH1_01T07051	2.01566	14.69437	UTR	1	9509505	9511861	2357	1
FGRAMPH1_01G07053	FGRAMPH1_01T07053	1.99604	13.76516	Intergenic	1	9513831	9514184	354	2

FGRAMPH1_01G07061	FGRAMPH1_01T07061	2.14457	21.12455	UTR	1	9527579	9527791	213	2
FGRAMPH1_01G07075	FGRAMPH1_01T07075	1.9644	13.15382	Promoter	1	9542783	9543622	840	2
ENSRNA049513793	ENSRNA049513793-T1	1.78606	10.56903	Promoter	1	9566346	9566465	120	2
FGRAMPH1_01G07089	FGRAMPH1_01T07089	1.7729	12.60853	Promoter	1	9569379	9571334	1956	1
FGRAMPH1_01G07095	FGRAMPH1_01T07095	1.54024	7.20573	Promoter	1	9575649	9579293	3645	1
FGRAMPH1_01G07109	FGRAMPH1_01T07109	1.62865	7.03993	Promoter	1	9597133	9597372	240	2
FGRAMPH1_01G07133	FGRAMPH1_01T07133	1.73944	10.97512	Promoter	1	9634785	9635573	789	2
FGRAMPH1_01G07135	FGRAMPH1_01T07135	1.75818	12.76013	Promoter	1	9637003	9637776	774	1
FGRAMPH1_01G07141	FGRAMPH1_01T07141	1.57185	6.11264	Promoter	1	9643782	9646147	2366	2
ENSRNA049559081	ENSRNA049559081-T1	2.23579	21.28687	Promoter	1	9646541	9646622	82	2
FGRAMPH1_01G07143	FGRAMPH1_01T07143	2.04462	15.3355	Promoter	1	9646818	9651835	5018	2
FGRAMPH1_01G07155	FGRAMPH1_01T07155	1.57423	5.68813	Promoter	1	9669388	9671293	1906	2
FGRAMPH1_01G07157	FGRAMPH1_01T07157	1.51673	5.45687	Promoter	1	9672169	9674449	2281	2

FGRAMPH1_01G07159	FGRAMPH1_01T07159	1.60197	7.7121	UTR	1	9674869	9677611	2743	1
FGRAMPH1_01G07161	FGRAMPH1_01T07161	1.7306	9.29734	Promoter	1	9678195	9679154	960	1
FGRAMPH1_01G07163	FGRAMPH1_01T07163	1.44475	4.88164	Promoter	1	9680252	9681498	1247	2
FGRAMPH1_01G07177	FGRAMPH1_01T07177	1.41853	4.44939	Promoter	1	9693329	9694378	1050	2
FGRAMPH1_01G07179	FGRAMPH1_01T07179	1.39491	5.85143	Promoter	1	9699112	9704442	5331	1
FGRAMPH1_01G07181	FGRAMPH1_01T07181	1.40405	5.2296	Promoter	1	9704191	9704670	480	2
FGRAMPH1_01G07201	FGRAMPH1_01T07201	1.33578	3.67974	Promoter	1	9730275	9733671	3397	1
FGRAMPH1_01G07209	FGRAMPH1_01T07209	1.91128	13.08983	Promoter	1	9737800	9738165	366	2
FGRAMPH1_01G07211	FGRAMPH1_01T07211	2.13603	20.81978	Promoter	1	9744655	9748513	3859	1
FGRAMPH1_01G07233	FGRAMPH1_01T07233	1.41238	5.48387	Promoter	1	9770068	9772372	2305	1
FGRAMPH1_01G07253	FGRAMPH1_01T07253	1.80687	11.19726	Promoter	1	9806338	9809178	2841	2
FGRAMPH1_01G07255	FGRAMPH1_01T07255	2.35458	23.09437	Promoter	1	9811745	9811834	90	1
FGRAMPH1_01G07269	FGRAMPH1_01T07269	1.85918	12.22028	Promoter	1	9830015	9830350	336	2

FGRAMPH1_01G07273	FGRAMPH1_01T07273	1.4845	8.0745	Promoter	1	9835770	9835936	167	1
FGRAMPH1_01G07277	FGRAMPH1_01T07277	2.11204	16.65722	Promoter	1	9845494	9846744	1251	2
FGRAMPH1_01G07297	FGRAMPH1_01T07297	1.85221	12.28101	Exon	1	9870773	9870961	189	2
FGRAMPH1_01G07301	FGRAMPH1_01T07301	1.43935	4.81085	Exon	1	9873425	9873580	156	2
FGRAMPH1_01G07311	FGRAMPH1_01T07311	1.68277	9.99905	Promoter	1	9886931	9887453	523	1
FGRAMPH1_01G07319	FGRAMPH1_01T07319	2.3124	21.592	Promoter	1	9892583	9892815	233	2
FGRAMPH1_01G07321	FGRAMPH1_01T07321	2.12258	16.65722	Promoter	1	9896707	9897747	1041	1
FGRAMPH1_01G07325	FGRAMPH1_01T07325	1.49112	5.17813	Promoter	1	9903440	9905869	2430	2
FGRAMPH1_01G07327	FGRAMPH1_01T07327	1.48522	5.68288	Promoter	1	9910266	9913114	2849	1
FGRAMPH1_01G07329	FGRAMPH1_01T07329	1.58459	6.83167	Promoter	1	9913814	9914586	773	1
FGRAMPH1_01G07331	FGRAMPH1_01T07331	1.73646	11.60626	Promoter	1	9915958	9917719	1762	1
FGRAMPH1_01G07333	FGRAMPH1_01T07333	1.96765	15.91438	Promoter	1	9918040	9919552	1513	1
ENSRNA049559047	ENSRNA049559047-T1	2.04876	15.02239	Promoter	1	9922459	9922530	72	2

FGRAMPH1_01G07351	FGRAMPH1_01T07351	2.18429	19.66681	Promoter	1	9942826	9943387	562	2
FGRAMPH1_01G07353	FGRAMPH1_01T07353	1.92707	13.36779	Promoter	1	9947770	9949639	1870	2
FGRAMPH1_01G07355	FGRAMPH1_01T07355	1.89253	15.04081	Promoter	1	9954456	9957664	3209	1
FGRAMPH1_01G07357	FGRAMPH1_01T07357	1.67075	7.8637	Promoter	1	9958162	9960258	2097	1
FGRAMPH1_01G07369	FGRAMPH1_01T07369	1.77044	13.85843	UTR	1	9968255	9968758	504	1
FGRAMPH1_01G07367	FGRAMPH1_01T07367	1.50915	7.7488	Promoter	1	9968141	9970440	2300	2
FGRAMPH1_01G07371	FGRAMPH1_01T07371	2.13202	21.15421	Promoter	1	9974118	9974487	370	1
FGRAMPH1_01G07385	FGRAMPH1_01T07385	1.73989	10.3902	Promoter	1	9987955	9991243	3289	2
FGRAMPH1_01G07389	FGRAMPH1_01T07389	1.67389	10.34582	Promoter	1	9999419	10000946	1528	1
FGRAMPH1_01G07407	FGRAMPH1_01T07407	1.5215	4.88081	UTR	1	10016273	10018119	1847	2
FGRAMPH1_01G07439	FGRAMPH1_01T07439	1.59193	7.78237	Promoter	1	10062788	10063510	723	1
FGRAMPH1_01G07443	FGRAMPH1_01T07443	1.94803	13.94974	Promoter	1	10069523	10072869	3347	2
FGRAMPH1_01G07445	FGRAMPH1_01T07445	1.64692	7.14578	Promoter	1	10076091	10078742	2652	2

FGRAMPH1_01G07449	FGRAMPH1_01T07449	1.97495	13.45803	Promoter	1	10084116	10085855	1740	1
FGRAMPH1_01G07467	FGRAMPH1_01T07467	2.13735	18.88242	UTR	1	10106670	10107945	1276	1
ENSRNA049559019	ENSRNA049559019-T1	2.68127	33.56064	Promoter	1	10115819	10115899	81	2
FGRAMPH1_01G07489	FGRAMPH1_01T07489	1.7535	8.69796	Promoter	1	10129950	10133116	3167	2
FGRAMPH1_01G07527	FGRAMPH1_01T07527	1.87069	12.8705	Promoter	1	10179628	10181497	1870	1
FGRAMPH1_01G07531	FGRAMPH1_01T07531	1.96823	14.92126	Promoter	1	10183923	10185139	1217	2
ENSRNA049558859	ENSRNA049558859-T1	2.51402	29.01273	Promoter	1	10187792	10187873	82	1
ENSRNA049559000	ENSRNA049559000-T1	2.59712	30.33056	Promoter	1	10206816	10206905	90	2
FGRAMPH1_01G07551	FGRAMPH1_01T07551	2.18585	18.36041	Promoter	1	10208754	10210444	1691	2
FGRAMPH1_01G07553	FGRAMPH1_01T07553	1.90321	16.05654	Promoter	1	10212614	10213441	828	1
FGRAMPH1_01G07575	FGRAMPH1_01T07575	1.59532	5.89894	UTR	1	10231670	10232107	438	1
FGRAMPH1_01G07577	FGRAMPH1_01T07577	1.71131	7.95267	Promoter	1	10232109	10233471	1363	2
FGRAMPH1_01G07579	FGRAMPH1_01T07579	1.58562	7.44186	Promoter	1	10235316	10237242	1927	2

FGRAMPH1_01G07581	FGRAMPH1_01T07581	1.35939	3.70221	Promoter	1	10237559	10238491	933	1
FGRAMPH1_01G07591	FGRAMPH1_01T07591	1.63615	9.04017	Promoter	1	10249538	10254862	5325	1
FGRAMPH1_01G07607	FGRAMPH1_01T07607	1.42855	5.30489	Exon	1	10271119	10272015	897	2
FGRAMPH1_01G07611	FGRAMPH1_01T07611	2.05464	16.28532	UTR	1	10273194	10273393	200	1
FGRAMPH1_01G07633	FGRAMPH1_01T07633	1.83113	11.21728	UTR	1	10307380	10308271	892	2
FGRAMPH1_01G07635	FGRAMPH1_01T07635	2.11204	16.65722	UTR	1	10308376	10309495	1120	1
FGRAMPH1_01G07637	FGRAMPH1_01T07637	1.88004	11.39068	Promoter	1	10311490	10313199	1710	1
FGRAMPH1_01G07639	FGRAMPH1_01T07639	1.94376	13.15472	Promoter	1	10313528	10313920	393	2
FGRAMPH1_01G07655	FGRAMPH1_01T07655	1.72294	9.8959	Promoter	1	10331250	10332192	943	1
FGRAMPH1_01G07665	FGRAMPH1_01T07665	1.65732	6.9886	UTR	1	10341007	10342444	1438	2
FGRAMPH1_01G07679	FGRAMPH1_01T07679	2.01623	20.91193	UTR	1	10360224	10362102	1879	2
FGRAMPH1_01G07681	FGRAMPH1_01T07681	1.52012	8.02699	Promoter	1	10362914	10363294	381	2
FGRAMPH1_01G07697	FGRAMPH1_01T07697	1.63796	7.29219	Promoter	1	10388557	10392787	4231	1

ENSRNA049512251	ENSRNA049512251-T1	2.13313	16.99246	Exon	1	10419979	10420432	454	2
FGRAMPH1_01G07717	FGRAMPH1_01T07717	2.35458	23.09437	UTR	1	10422427	10423779	1353	2
FGRAMPH1_01G07719	FGRAMPH1_01T07719	1.76122	10.56439	Promoter	1	10424691	10424945	255	1
FGRAMPH1_01G07721	FGRAMPH1_01T07721	1.69022	7.4724	Exon	1	10426154	10426459	306	2
FGRAMPH1_01G07757	FGRAMPH1_01T07757	1.45737	5.85903	Promoter	1	10469280	10470610	1331	1
FGRAMPH1_01G07767	FGRAMPH1_01T07767	1.83358	12.23857	Promoter	1	10475437	10477062	1626	2
FGRAMPH1_01G07769	FGRAMPH1_01T07769	1.34222	3.81676	Promoter	1	10477889	10479971	2083	2
FGRAMPH1_01G07771	FGRAMPH1_01T07771	1.74559	8.88634	Promoter	1	10481124	10481448	325	1
FGRAMPH1_01G07773	FGRAMPH1_01T07773	1.5319	6.67199	Promoter	1	10481857	10484543	2687	1
FGRAMPH1_01G07775	FGRAMPH1_01T07775	1.67081	8.39168	Promoter	1	10486096	10486942	847	1
FGRAMPH1_01G07785	FGRAMPH1_01T07785	1.71993	9.07955	Promoter	1	10494233	10496177	1945	1
FGRAMPH1_01G07787	FGRAMPH1_01T07787	1.98492	14.96756	Promoter	1	10497344	10498042	699	1
FGRAMPH1_01G07795	FGRAMPH1_01T07795	1.92441	13.24004	Promoter	1	10503820	10506205	2386	1

FGRAMPH1_01G07811	FGRAMPH1_01T07811	1.84313	13.31216	Promoter	1	10518950	10519504	555	2
ENSRNA049558877	ENSRNA049558877-T1	2.73421	34.66857	Promoter	1	10532118	10532208	91	1
FGRAMPH1_01G07827	FGRAMPH1_01T07827	1.47034	4.15941	Promoter	1	10536139	10540417	4279	1
FGRAMPH1_01G07857	FGRAMPH1_01T07857	1.99604	13.76516	Promoter	1	10566260	10568672	2413	2
FGRAMPH1_01G07867	FGRAMPH1_01T07867	1.91167	11.96643	Promoter	1	10578137	10578781	645	1
FGRAMPH1_01G07869	FGRAMPH1_01T07869	1.34146	3.63172	Promoter	1	10579532	10581959	2428	1
FGRAMPH1_01G07873	FGRAMPH1_01T07873	2.14161	20.82021	Promoter	1	10586302	10587525	1224	1
FGRAMPH1_01G07883	FGRAMPH1_01T07883	2.16843	21.67339	Promoter	1	10602509	10602679	171	2
FGRAMPH1_01G07899	FGRAMPH1_01T07899	1.49714	6.3	Promoter	1	10632395	10633558	1164	2
FGRAMPH1_01G07915	FGRAMPH1_01T07915	2.15258	19.45533	Promoter	1	10657651	10659064	1414	1
FGRAMPH1_01G07929	FGRAMPH1_01T07929	1.75641	10.12336	Promoter	1	10671847	10673991	2145	2
FGRAMPH1_01G07939	FGRAMPH1_01T07939	1.67968	7.4724	Promoter	1	10682416	10683162	747	2
FGRAMPH1_01G07945	FGRAMPH1_01T07945	1.65726	8.38887	UTR	1	10689562	10693370	3809	2

FGRAMPH1_01G07955	FGRAMPH1_01T07955	1.47428	4.66904	Promoter	1	10703733	10705864	2132	1
FGRAMPH1_01G07957	FGRAMPH1_01T07957	2.57128	31.83686	Promoter	1	10706301	10706808	508	1
ENSRNA049558887	ENSRNA049558887-T1	2.20694	19.06036	Promoter	1	10714220	10714314	95	1
FGRAMPH1_01G07961	FGRAMPH1_01T07961	1.67968	7.4724	Promoter	1	10714905	10715167	263	2
FGRAMPH1_01G07963	FGRAMPH1_01T07963	2.05277	16.98671	Promoter	1	10717109	10717315	207	1
ENSRNA049558908	ENSRNA049558908-T1	2.20457	22.29341	Promoter	1	10722515	10722609	95	1
FGRAMPH1_01G07967	FGRAMPH1_01T07967	2.05867	15.43774	Promoter	1	10725234	10726163	930	2
FGRAMPH1_01G07969	FGRAMPH1_01T07969	1.98753	15.59803	Promoter	1	10729887	10730243	357	1
FGRAMPH1_01G07975	FGRAMPH1_01T07975	1.85916	11.61818	Promoter	1	10737762	10738178	417	2
FGRAMPH1_01G07977	FGRAMPH1_01T07977	2.22336	20.58	Promoter	1	10740452	10742724	2273	2
FGRAMPH1_01G08011	FGRAMPH1_01T08011	1.57826	6.70814	Promoter	1	10780703	10784266	3564	1
ENSRNA049558922	ENSRNA049558922-T1	2.19279	22.20001	Promoter	1	10821755	10821847	93	1
FGRAMPH1_01G08059	FGRAMPH1_01T08059	1.78513	9.21114	Promoter	1	10830054	10832251	2198	2

FGRAMPH1_01G08087	FGRAMPH1_01T08087	2.24912	20.12992	Promoter	1	10858661	10861327	2667	2
FGRAMPH1_01G08093	FGRAMPH1_01T08093	1.7535	8.69796	Promoter	1	10866790	10869589	2800	1
FGRAMPH1_01G08175	FGRAMPH1_01T08175	1.77459	9.21114	Promoter	1	10963710	10965275	1566	1
FGRAMPH1_01G08227	FGRAMPH1_01T08227	1.91167	11.96643	Intergenic	1	11032946	11033198	253	1
FGRAMPH1_01G08253	FGRAMPH1_01T08253	2.21749	19.06036	Promoter	1	11064065	11064551	487	2
FGRAMPH1_01G08255	FGRAMPH1_01T08255	1.47936	6.09031	Promoter	1	11067080	11069588	2509	1
FGRAMPH1_01G08275	FGRAMPH1_01T08275	1.72186	8.19781	Promoter	1	11089580	11091622	2043	2
FGRAMPH1_01G08279	FGRAMPH1_01T08279	1.58477	5.89894	Exon	1	11094339	11095196	858	1
ENSRNA049558973	ENSRNA049558973-T1	2.06685	18.43514	Promoter	1	11110419	11110508	90	2
FGRAMPH1_01G08303	FGRAMPH1_01T08303	1.29933	3.71503	Promoter	1	11119201	11121496	2296	2
FGRAMPH1_01G08307	FGRAMPH1_01T08307	1.43569	6.14987	UTR	1	11122383	11122627	245	1
FGRAMPH1_01G08305	FGRAMPH1_01T08305	1.77651	10.19372	Promoter	1	11122080	11125954	3875	2
FGRAMPH1_01G08309	FGRAMPH1_01T08309	1.97376	15.68573	Promoter	1	11127404	11127808	405	2

FGRAMPH1_01G08311	FGRAMPH1_01T08311	2.02954	16.77752	Promoter	1	11130786	11131049	264	2
FGRAMPH1_01G08321	FGRAMPH1_01T08321	2.55494	28.6574	Promoter	1	11147255	11147666	412	1
FGRAMPH1_01G08323	FGRAMPH1_01T08323	1.87739	13.34721	Promoter	1	11147792	11151585	3794	1
FGRAMPH1_01G08343	FGRAMPH1_01T08343	2.07635	18.08936	Promoter	1	11192462	11195229	2768	1
ENSRNA049558937	ENSRNA049558937-T1	2.76584	35.56207	Promoter	1	11199590	11199674	85	1
FGRAMPH1_01G08383	FGRAMPH1_01T08383	1.50041	4.49906	Promoter	1	11266101	11273705	7605	1
FGRAMPH1_01G08387	FGRAMPH1_01T08387	2.69203	33.34432	Promoter	1	11275284	11277957	2674	1
FGRAMPH1_01G08385	FGRAMPH1_01T08385	1.83786	10.2759	Promoter	1	11273597	11275332	1736	2
FGRAMPH1_01G08395	FGRAMPH1_01T08395	2.08228	16.13632	Promoter	1	11283215	11283947	733	2
FGRAMPH1_01G08399	FGRAMPH1_01T08399	1.64804	6.77735	Exon	1	11284933	11285665	733	2
FGRAMPH1_01G08427	FGRAMPH1_01T08427	1.78376	9.19115	Promoter	1	11334588	11335530	943	2
ENSRNA049512342	ENSRNA049512342-T1	2.05857	15.82566	Promoter	1	11436728	11436847	120	2
FGRAMPH1_01G08519	FGRAMPH1_01T08519	1.77401	9.83989	Promoter	1	11448012	11449604	1593	2

FGRAMPH1_01G08547	FGRAMPH1_01T08547	1.79568	9.47257	Promoter	1	11485461	11485890	430	1
FGRAMPH1_01G08621	FGRAMPH1_01T08621	2.23858	19.7708	Promoter	1	11568603	11570786	2184	2
FGRAMPH1_01G08623	FGRAMPH1_01T08623	1.63325	6.78533	Promoter	1	11572261	11573740	1480	1
FGRAMPH1_01G08631	FGRAMPH1_01T08631	2.74475	34.66857	Promoter	1	11580415	11582355	1941	2
FGRAMPH1_01G08633	FGRAMPH1_01T08633	1.6375	6.77735	Promoter	1	11584504	11586231	1728	1
FGRAMPH1_01G08743	FGRAMPH1_01T08743	1.65859	7.00562	Promoter	1	11704849	11709715	4867	2
FGRAMPH1_01G08915	FGRAMPH1_01T08915	2.32293	24.31315	Exon	2	175870	176262	393	2
FGRAMPH1_01G08955	FGRAMPH1_01T08955	1.76404	8.95294	Promoter	2	219947	221766	1820	2
FGRAMPH1_01G08959	FGRAMPH1_01T08959	2.01062	15.07178	Promoter	2	223708	227654	3947	2
FGRAMPH1_01G08961	FGRAMPH1_01T08961	1.90113	11.67704	Promoter	2	228191	228804	614	2
FGRAMPH1_01G08963	FGRAMPH1_01T08963	1.73241	8.44624	Promoter	2	230158	230759	602	1
ENSRNA049559590	ENSRNA049559590-T1	2.47087	26.41592	Promoter	2	246720	246793	74	1
FGRAMPH1_01G09007	FGRAMPH1_01T09007	2.16476	18.0144	UTR	2	273869	275112	1244	1

FGRAMPH1_01G09009	FGRAMPH1_01T09009	2.39676	24.24705	Promoter	2	275146	276916	1771	2
FGRAMPH1_01G09011	FGRAMPH1_01T09011	2.59712	30.33056	Promoter	2	277477	277806	330	1
FGRAMPH1_01G09161	FGRAMPH1_01T09161	1.44627	5.78639	Promoter	2	441220	444810	3591	2
FGRAMPH1_01G09163	FGRAMPH1_01T09163	1.61641	6.33116	Exon	2	446772	447710	939	2
FGRAMPH1_01G09165	FGRAMPH1_01T09165	1.89268	16.84167	Promoter	2	450028	452595	2568	2
FGRAMPH1_01G09167	FGRAMPH1_01T09167	2.97675	43.00728	Promoter	2	453737	453946	210	2
FGRAMPH1_01G09169	FGRAMPH1_01T09169	2.82911	37.83256	Promoter	2	456506	456658	153	1
FGRAMPH1_01G09175	FGRAMPH1_01T09175	1.8484	10.54995	Exon	2	461002	461894	893	2
FGRAMPH1_01G09177	FGRAMPH1_01T09177	1.65859	7.00562	Promoter	2	462777	463597	821	2
FGRAMPH1_01G09187	FGRAMPH1_01T09187	2.04104	15.89655	Promoter	2	479471	480609	1139	1
FGRAMPH1_01G09207	FGRAMPH1_01T09207	1.83786	10.2759	Promoter	2	505285	507530	2246	2
ENSRNA049562130	ENSRNA049562130-T1	2.49571	27.8653	Promoter	2	559823	559911	89	2
FGRAMPH1_01G09269	FGRAMPH1_01T09269	2.01713	14.38806	Promoter	2	581066	583776	2711	2

ENSRNA049562082	ENSRNA049562082-T1	2.12864	19.7035	Promoter	2	594448	594534	87	2
FGRAMPH1_01G09363	FGRAMPH1_01T09363	1.71131	7.95267	Promoter	2	680023	681564	1542	1
FGRAMPH1_01G09399	FGRAMPH1_01T09399	1.56368	5.48089	Promoter	2	735413	736582	1170	2
FGRAMPH1_01G09401	FGRAMPH1_01T09401	2.32294	21.96385	Promoter	2	737454	739410	1957	2
FGRAMPH1_01G09403	FGRAMPH1_01T09403	1.85565	12.11211	Promoter	2	740440	741917	1478	2
FGRAMPH1_01G09441	FGRAMPH1_01T09441	2.60766	30.33056	Promoter	2	781897	782709	813	2
FGRAMPH1_01G09525	FGRAMPH1_01T09525	1.91682	15.95299	Promoter	2	870398	871106	709	1
FGRAMPH1_01G09527	FGRAMPH1_01T09527	1.88689	12.34777	Promoter	2	872966	875050	2085	1
FGRAMPH1_01G09531	FGRAMPH1_01T09531	1.64665	7.55885	Promoter	2	877180	879472	2293	2
ENSRNA049559617	ENSRNA049559617-T1	2.27021	20.49161	Promoter	2	905361	905432	72	1
ENSRNA049559648	ENSRNA049559648-T1	2.26004	21.09255	Promoter	2	925786	925857	72	1
FGRAMPH1_01G09575	FGRAMPH1_01T09575	1.67968	7.4724	Promoter	2	930873	933505	2633	1
FGRAMPH1_01G09581	FGRAMPH1_01T09581	1.78409	9.61928	Promoter	2	935435	935689	255	2

FGRAMPH1_01G09589	FGRAMPH1_01T09589	1.9644	13.15382	Promoter	2	941783	942936	1154	1
FGRAMPH1_01G09601	FGRAMPH1_01T09601	1.66557	7.96568	Promoter	2	953181	954272	1092	2
FGRAMPH1_01G09609	FGRAMPH1_01T09609	2.11839	18.36343	Exon	2	962845	963633	789	2
FGRAMPH1_01G09611	FGRAMPH1_01T09611	1.45035	6.87809	Intergenic	2	966473	966784	312	2
FGRAMPH1_01G09613	FGRAMPH1_01T09613	1.65188	11.3659	Exon	2	967458	967989	532	1
FGRAMPH1_01G09615	FGRAMPH1_01T09615	1.64173	9.26251	Promoter	2	967922	968158	237	2
FGRAMPH1_01G09617	FGRAMPH1_01T09617	1.2942	3.59323	Promoter	2	971753	973018	1266	1
FGRAMPH1_01G09623	FGRAMPH1_01T09623	2.05931	15.34379	Promoter	2	983793	988208	4416	1
FGRAMPH1_01G09633	FGRAMPH1_01T09633	1.65237	7.67837	Promoter	2	999215	1004785	5571	2
FGRAMPH1_01G09635	FGRAMPH1_01T09635	1.84561	11.82237	Intergenic	2	1005752	1005971	220	2
FGRAMPH1_01G09655	FGRAMPH1_01T09655	2.78693	36.46401	Promoter	2	1032052	1034510	2459	2
FGRAMPH1_01G09663	FGRAMPH1_01T09663	2.13656	20.01796	Intergenic	2	1043110	1043214	105	1
FGRAMPH1_01G09671	FGRAMPH1_01T09671	1.85218	13.35508	Promoter	2	1057112	1060422	3311	1

ENSRNA049513589	ENSRNA049513589-T1	2.53353	31.40496	Promoter	2	1074971	1075131	161	1
FGRAMPH1_01G09691	FGRAMPH1_01T09691	1.74581	12.34476	Promoter	2	1077093	1079696	2604	2
FGRAMPH1_01G09693	FGRAMPH1_01T09693	2.00465	17.38478	Promoter	2	1081356	1083611	2256	1
FGRAMPH1_01G09719	FGRAMPH1_01T09719	1.54699	7.1596	Promoter	2	1108395	1109532	1138	1
ENSRNA049562018	ENSRNA049562018-T1	2.81857	37.37431	Promoter	2	1109911	1109984	74	2
FGRAMPH1_01G09721	FGRAMPH1_01T09721	2.53385	28.2448	Promoter	2	1111015	1111383	369	1
FGRAMPH1_01G09723	FGRAMPH1_01T09723	1.81677	10.00496	Promoter	2	1112137	1113732	1596	1
FGRAMPH1_01G09731	FGRAMPH1_01T09731	1.80719	10.36905	UTR	2	1119043	1121375	2333	1
FGRAMPH1_01G09735	FGRAMPH1_01T09735	1.94647	14.1763	Promoter	2	1123225	1126389	3165	2
FGRAMPH1_01G09737	FGRAMPH1_01T09737	1.55777	5.92179	Promoter	2	1127863	1128487	625	1
FGRAMPH1_01G09751	FGRAMPH1_01T09751	1.6375	6.77735	Promoter	2	1138869	1141504	2636	2
FGRAMPH1_01G09755	FGRAMPH1_01T09755	1.93277	12.25882	Promoter	2	1141687	1143394	1708	2
FGRAMPH1_01G09757	FGRAMPH1_01T09757	1.6349	8.20899	Intergenic	2	1144952	1145199	248	2

FGRAMPH1_01G09799	FGRAMPH1_01T09799	1.64837	8.90314	Promoter	2	1186230	1191065	4836	2
ENSRNA049559675	ENSRNA049559675-T1	2.23858	19.7708	Promoter	2	1194021	1194102	82	1
ENSRNA049561969	ENSRNA049561969-T1	2.02767	14.38806	Promoter	2	1195595	1195678	84	2
FGRAMPH1_01G09813	FGRAMPH1_01T09813	1.63922	7.89154	Exon	2	1209316	1210134	819	2
FGRAMPH1_01G09815	FGRAMPH1_01T09815	1.56142	7.94224	Promoter	2	1210999	1214685	3687	1
FGRAMPH1_01G09839	FGRAMPH1_01T09839	1.59833	6.87653	Promoter	2	1240481	1242478	1998	2
FGRAMPH1_01G09843	FGRAMPH1_01T09843	1.41504	6.31745	UTR	2	1249408	1253362	3955	2
FGRAMPH1_01G09845	FGRAMPH1_01T09845	1.33595	4.41968	Promoter	2	1256682	1256822	141	2
FGRAMPH1_01G09847	FGRAMPH1_01T09847	2.39557	28.12662	Promoter	2	1262343	1263899	1557	1
FGRAMPH1_01G09853	FGRAMPH1_01T09853	1.54479	6.1887	Promoter	2	1270244	1270456	213	1
FGRAMPH1_01G09859	FGRAMPH1_01T09859	1.47837	5.64159	Promoter	2	1276198	1278186	1989	1
ENSRNA049561954	ENSRNA049561954-T1	1.87534	13.57069	Promoter	2	1287107	1287196	90	2
FGRAMPH1_01G09867	FGRAMPH1_01T09867	1.73576	11.19972	UTR	2	1287279	1291829	4551	2

FGRAMPH1_01G09929	FGRAMPH1_01T09929	1.80215	11.66615	Promoter	2	1362031	1363664	1634	2
FGRAMPH1_01G09931	FGRAMPH1_01T09931	1.88825	12.37111	UTR	2	1367085	1369839	2755	1
FGRAMPH1_01G09933	FGRAMPH1_01T09933	1.9482	14.76042	Promoter	2	1370076	1371497	1422	1
FGRAMPH1_01G09941	FGRAMPH1_01T09941	2.18452	19.64112	Promoter	2	1377445	1379830	2386	1
FGRAMPH1_01G09951	FGRAMPH1_01T09951	1.44324	4.48866	Promoter	2	1388930	1393597	4668	1
FGRAMPH1_01G09955	FGRAMPH1_01T09955	1.67982	8.40417	Promoter	2	1393792	1395261	1470	1
FGRAMPH1_01G09971	FGRAMPH1_01T09971	1.66913	7.23732	Promoter	2	1415477	1416729	1253	2
FGRAMPH1_01G09973	FGRAMPH1_01T09973	1.32348	3.47489	Promoter	2	1418129	1418281	153	2
FGRAMPH1_01G09983	FGRAMPH1_01T09983	1.55408	5.60756	Promoter	2	1428907	1432360	3454	1
FGRAMPH1_01G09985	FGRAMPH1_01T09985	1.65933	12.08279	Promoter	2	1435742	1439028	3287	1
FGRAMPH1_01G09987	FGRAMPH1_01T09987	1.64304	7.03916	Promoter	2	1440005	1442439	2435	1
ENSRNA049513501	ENSRNA049513501-T1	1.45823	3.95439	Intergenic	2	1445900	1445980	81	2
ENSRNA049513413	ENSRNA049513413-T1	1.49616	5.61503	Promoter	2	1446144	1446231	88	2

FGRAMPH1_01G09995	FGRAMPH1_01T09995	1.98549	13.45803	Promoter	2	1449244	1450918	1675	2
FGRAMPH1_01G10003	FGRAMPH1_01T10003	2.0088	16.60322	Promoter	2	1458957	1461284	2328	1
FGRAMPH1_01G10009	FGRAMPH1_01T10009	2.14022	20.48551	Promoter	2	1469226	1469606	381	2
FGRAMPH1_01G10013	FGRAMPH1_01T10013	1.81092	10.3674	Promoter	2	1475876	1477153	1278	1
FGRAMPH1_01G10011	FGRAMPH1_01T10011	1.25684	3.02902	Promoter	2	1474721	1480772	6052	2
FGRAMPH1_01G10021	FGRAMPH1_01T10021	1.6038	7.52556	Promoter	2	1486599	1489639	3041	1
ENSRNA049559688	ENSRNA049559688-T1	2.48691	29.21589	Promoter	2	1491878	1491961	84	1
ENSRNA049559735	ENSRNA049559735-T1	2.62354	31.49517	Promoter	2	1492525	1492596	72	1
FGRAMPH1_01G10025	FGRAMPH1_01T10025	1.86677	12.72784	Promoter	2	1493925	1497122	3198	2
FGRAMPH1_01G10039	FGRAMPH1_01T10039	1.30975	3.3543	Promoter	2	1514691	1515827	1137	2
ENSRNA049561932	ENSRNA049561932-T1	2.49523	27.85552	Promoter	2	1527083	1527164	82	2
FGRAMPH1_01G10047	FGRAMPH1_01T10047	1.83994	11.88356	Promoter	2	1529887	1533547	3661	2
FGRAMPH1_01G10049	FGRAMPH1_01T10049	1.90951	14.22782	Promoter	2	1535000	1535961	962	1

FGRAMPH1_01G10063	FGRAMPH1_01T10063	1.80723	10.75646	Promoter	2	1557303	1557586	284	2
FGRAMPH1_01G10081	FGRAMPH1_01T10081	1.96791	13.29985	Promoter	2	1572234	1576719	4486	1
FGRAMPH1_01G10121	FGRAMPH1_01T10121	1.80768	14.54937	Promoter	2	1632319	1632786	468	2
FGRAMPH1_01G10123	FGRAMPH1_01T10123	1.76157	13.08926	Promoter	2	1637796	1639009	1214	2
FGRAMPH1_01G10131	FGRAMPH1_01T10131	1.84694	12.9119	Promoter	2	1657110	1658622	1513	1
FGRAMPH1_01G10141	FGRAMPH1_01T10141	1.49771	6.25673	Promoter	2	1671457	1673494	2038	1
FGRAMPH1_01G10185	FGRAMPH1_01T10185	2.1383	19.8689	Promoter	2	1728837	1730273	1437	1
FGRAMPH1_01G10197	FGRAMPH1_01T10197	1.69876	10.89542	Exon	2	1756120	1756301	182	2
FGRAMPH1_01G10201	FGRAMPH1_01T10201	1.5215	4.88081	Promoter	2	1758795	1760628	1834	2
FGRAMPH1_01G10205	FGRAMPH1_01T10205	1.89058	11.39068	Promoter	2	1765774	1768352	2579	1
ENSRNA049559773	ENSRNA049559773-T1	2.36164	23.20886	Promoter	2	1771274	1771367	94	1
FGRAMPH1_01G10233	FGRAMPH1_01T10233	2.01958	17.42358	Promoter	2	1807301	1807639	339	1
FGRAMPH1_01G10235	FGRAMPH1_01T10235	1.55349	6.48508	Promoter	2	1813194	1817169	3976	1

ENSRNA049513367	ENSRNA049513367-T1	1.62934	7.33498	Promoter	2	1817291	1817410	120	1
FGRAMPH1_01G10249	FGRAMPH1_01T10249	1.69018	8.1537	Promoter	2	1824488	1825581	1094	2
FGRAMPH1_01G10251	FGRAMPH1_01T10251	1.53301	6.01624	Promoter	2	1827314	1829369	2056	2
ENSRNA049513654	ENSRNA049513654-T1	1.82731	10.2759	UTR	2	1845836	1845936	101	1
FGRAMPH1_01G10295	FGRAMPH1_01T10295	1.68275	11.63255	Promoter	2	1884011	1884248	238	2
FGRAMPH1_01G10321	FGRAMPH1_01T10321	1.80622	9.73718	Promoter	2	1917707	1920806	3100	2
FGRAMPH1_01G10339	FGRAMPH1_01T10339	1.85895	10.82712	Promoter	2	1951909	1957900	5992	1
FGRAMPH1_01G10357	FGRAMPH1_01T10357	1.86249	10.95196	Promoter	2	1983647	1984941	1295	1
FGRAMPH1_01G10355	FGRAMPH1_01T10355	1.30792	3.17541	Promoter	2	1982729	1984284	1556	2
FGRAMPH1_01G10371	FGRAMPH1_01T10371	1.76884	9.91538	Promoter	2	2003960	2004415	456	1
FGRAMPH1_01G10373	FGRAMPH1_01T10373	1.53204	5.0772	Intergenic	2	2006288	2006587	300	2
ENSRNA049561864	ENSRNA049561864-T1	2.35458	23.09437	Promoter	2	2008674	2008763	90	2
ENSRNA049559794	ENSRNA049559794-T1	2.57603	29.48946	Promoter	2	2019382	2019471	90	1

FGRAMPH1_01G10385	FGRAMPH1_01T10385	1.66913	7.23732	Promoter	2	2022391	2024958	2568	1
FGRAMPH1_01G10391	FGRAMPH1_01T10391	1.66853	8.95427	Promoter	2	2030252	2030569	318	2
FGRAMPH1_01G10393	FGRAMPH1_01T10393	1.90961	12.21301	Intergenic	2	2031831	2032432	602	2
FGRAMPH1_01G10405	FGRAMPH1_01T10405	1.8484	10.54995	Promoter	2	2043909	2045375	1467	2
FGRAMPH1_01G10419	FGRAMPH1_01T10419	1.44916	5.07765	Promoter	2	2060796	2061047	252	1
FGRAMPH1_01G10429	FGRAMPH1_01T10429	1.72186	8.19781	Promoter	2	2071499	2071921	423	2
FGRAMPH1_01G10437	FGRAMPH1_01T10437	1.88272	13.78618	Exon	2	2088888	2089527	640	2
FGRAMPH1_01G10451	FGRAMPH1_01T10451	1.68195	8.21428	Promoter	2	2110680	2114257	3578	2
FGRAMPH1_01G10457	FGRAMPH1_01T10457	1.77459	9.21114	Intergenic	2	2120993	2121409	417	1
FGRAMPH1_01G10473	FGRAMPH1_01T10473	1.83516	11.21947	Promoter	2	2138540	2140512	1973	2
FGRAMPH1_01G10505	FGRAMPH1_01T10505	1.53204	5.0772	UTR	2	2182220	2183636	1417	1
FGRAMPH1_01G10507	FGRAMPH1_01T10507	1.3661	4.81569	UTR	2	2183983	2189596	5614	2
FGRAMPH1_01G10509	FGRAMPH1_01T10509	1.8484	10.54995	Promoter	2	2188508	2189908	1401	2

FGRAMPH1_01G10511	FGRAMPH1_01T10511	1.76863	11.6321	Intergenic	2	2194573	2195063	491	2
FGRAMPH1_01G10521	FGRAMPH1_01T10521	1.95594	15.76671	Promoter	2	2201926	2205528	3603	2
FGRAMPH1_01G10537	FGRAMPH1_01T10537	1.51995	5.89536	Promoter	2	2231949	2232140	192	2
FGRAMPH1_01G10547	FGRAMPH1_01T10547	1.42024	3.95959	Promoter	2	2239038	2239640	603	2
ENSRNA049561804	ENSRNA049561804-T1	2.79748	36.46401	Promoter	2	2250263	2250353	91	2
FGRAMPH1_01G10581	FGRAMPH1_01T10581	1.84324	12.4813	Promoter	2	2283416	2285225	1810	2
FGRAMPH1_01G10583	FGRAMPH1_01T10583	1.9742	16.13553	Promoter	2	2286933	2287919	987	1
FGRAMPH1_01G10585	FGRAMPH1_01T10585	1.73388	8.66261	Promoter	2	2289096	2290640	1545	1
FGRAMPH1_01G10587	FGRAMPH1_01T10587	1.67968	7.4724	Promoter	2	2290799	2292101	1303	2
FGRAMPH1_01G10589	FGRAMPH1_01T10589	2.30257	22.72476	Promoter	2	2294155	2294818	664	2
FGRAMPH1_01G10591	FGRAMPH1_01T10591	1.98277	17.8596	Promoter	2	2295295	2296295	1001	1
FGRAMPH1_01G10593	FGRAMPH1_01T10593	1.5189	7.64487	Promoter	2	2297508	2299853	2346	1
FGRAMPH1_01G10599	FGRAMPH1_01T10599	2.25726	21.51566	Promoter	2	2308797	2311608	2812	2

FGRAMPH1_01G10601	FGRAMPH1_01T10601	2.28459	21.58643	Exon	2	2311857	2313509	1653	1
FGRAMPH1_01G10603	FGRAMPH1_01T10603	1.51098	7.30885	Exon	2	2319882	2321408	1527	2
FGRAMPH1_01G10619	FGRAMPH1_01T10619	1.79702	10.69508	Promoter	2	2355760	2357525	1766	1
FGRAMPH1_01G10641	FGRAMPH1_01T10641	1.55073	6.68382	Promoter	2	2384379	2386049	1671	1
FGRAMPH1_01G10645	FGRAMPH1_01T10645	1.91731	13.31943	Promoter	2	2388594	2391612	3019	1
FGRAMPH1_01G10657	FGRAMPH1_01T10657	1.7429	12.82881	Promoter	2	2411145	2412720	1576	1
FGRAMPH1_01G10661	FGRAMPH1_01T10661	1.60287	6.34654	Promoter	2	2416909	2417133	225	1
FGRAMPH1_01G10681	FGRAMPH1_01T10681	1.85227	11.20564	Promoter	2	2444551	2444733	183	2
FGRAMPH1_01G10683	FGRAMPH1_01T10683	1.57604	5.78854	Exon	2	2445118	2448611	3494	2
FGRAMPH1_01G10685	FGRAMPH1_01T10685	1.76414	9.68294	Intergenic	2	2449706	2450286	581	1
FGRAMPH1_01G10689	FGRAMPH1_01T10689	1.53951	7.01275	UTR	2	2454929	2459839	4911	2
FGRAMPH1_01G10693	FGRAMPH1_01T10693	2.0131	15.89382	Intergenic	2	2468778	2469082	305	1
FGRAMPH1_01G10699	FGRAMPH1_01T10699	1.60364	8.79109	Promoter	2	2478525	2480940	2416	2

FGRAMPH1_01G10701	FGRAMPH1_01T10701	2.15891	19.7353	Promoter	2	2481308	2484323	3016	2
FGRAMPH1_01G10703	FGRAMPH1_01T10703	1.96446	14.28603	Promoter	2	2485122	2485319	198	2
FGRAMPH1_01G10705	FGRAMPH1_01T10705	1.94811	14.07857	Promoter	2	2486229	2490261	4033	1
FGRAMPH1_01G10707	FGRAMPH1_01T10707	1.73078	11.44321	Promoter	2	2490249	2493248	3000	2
ENSRNA049559830	ENSRNA049559830-T1	2.18585	18.36041	Promoter	2	2500915	2500996	82	1
FGRAMPH1_01G10719	FGRAMPH1_01T10719	1.51366	5.95526	Promoter	2	2502858	2503732	875	2
FGRAMPH1_01G10731	FGRAMPH1_01T10731	2.02636	16.37936	Promoter	2	2517294	2520136	2843	1
FGRAMPH1_01G10761	FGRAMPH1_01T10761	1.35874	3.42297	Intergenic	2	2555947	2556404	458	2
FGRAMPH1_01G10775	FGRAMPH1_01T10775	2.00371	15.5825	Promoter	2	2569368	2571942	2575	2
FGRAMPH1_01G10777	FGRAMPH1_01T10777	1.27929	3.00209	Exon	2	2572694	2573437	744	2
FGRAMPH1_01G10783	FGRAMPH1_01T10783	1.59532	5.89894	Promoter	2	2585373	2586449	1077	2
FGRAMPH1_01G10789	FGRAMPH1_01T10789	1.79568	9.47257	Promoter	2	2601536	2604087	2552	1
FGRAMPH1_01G10791	FGRAMPH1_01T10791	1.73552	12.17556	Promoter	2	2605131	2610597	5467	1

FGRAMPH1_01G10819	FGRAMPH1_01T10819	2.1071	17.35782	Promoter	2	2645467	2646576	1110	2
FGRAMPH1_01G10821	FGRAMPH1_01T10821	1.65883	7.59816	Promoter	2	2649909	2654096	4188	2
ENSRNA049559865	ENSRNA049559865-T1	1.75665	11.45478	Promoter	2	2657173	2657272	100	1
FGRAMPH1_01G10825	FGRAMPH1_01T10825	1.43445	4.61499	Promoter	2	2658746	2662277	3532	1
FGRAMPH1_01G10831	FGRAMPH1_01T10831	1.64804	6.77735	Promoter	2	2666532	2666792	261	1
ENSRNA049561749	ENSRNA049561749-T1	2.72366	34.22501	Promoter	2	2691816	2691897	82	2
FGRAMPH1_01G10853	FGRAMPH1_01T10853	1.33882	3.35036	Promoter	2	2704984	2707769	2786	1
FGRAMPH1_01G10857	FGRAMPH1_01T10857	2.02488	15.46508	UTR	2	2709037	2712905	3869	1
FGRAMPH1_01G10877	FGRAMPH1_01T10877	1.50689	8.09212	Exon	2	2739541	2741828	2288	1
FGRAMPH1_01G10893	FGRAMPH1_01T10893	1.82404	11.43157	Exon	2	2760577	2761294	718	1
FGRAMPH1_01G10899	FGRAMPH1_01T10899	1.59023	8.406	Promoter	2	2766739	2770450	3712	2
FGRAMPH1_01G10903	FGRAMPH1_01T10903	1.65859	7.00562	Promoter	2	2776198	2780190	3993	2
FGRAMPH1_01G10935	FGRAMPH1_01T10935	1.90113	11.67704	Promoter	2	2816718	2818308	1591	2

FGRAMPH1_01G10937	FGRAMPH1_01T10937	1.53204	5.0772	Promoter	2	2819205	2823521	4317	2
ENSRNA049561716	ENSRNA049561716-T1	2.46003	25.8178	Promoter	2	2840179	2840260	82	2
FGRAMPH1_01G10971	FGRAMPH1_01T10971	1.57321	5.97714	Promoter	2	2864367	2866522	2156	1
FGRAMPH1_01G10977	FGRAMPH1_01T10977	2.11071	17.57325	Promoter	2	2878417	2878701	285	1
FGRAMPH1_01G10993	FGRAMPH1_01T10993	2.73707	34.95806	Promoter	2	2896962	2900452	3491	1
FGRAMPH1_01G10995	FGRAMPH1_01T10995	1.56368	5.48089	Promoter	2	2900842	2901425	584	1
ENSRNA049559885	ENSRNA049559885-T1	2.51276	27.42648	Promoter	2	2918821	2918893	73	1
FGRAMPH1_01G11059	FGRAMPH1_01T11059	1.53647	6.13839	Promoter	2	2955111	2957831	2721	2
FGRAMPH1_01G11069	FGRAMPH1_01T11069	1.75945	9.14374	Promoter	2	2975542	2977080	1539	2
FGRAMPH1_01G11073	FGRAMPH1_01T11073	1.56368	5.48089	Promoter	2	2980445	2982185	1741	2
FGRAMPH1_01G11113	FGRAMPH1_01T11113	1.48216	4.92879	UTR	2	3027499	3030196	2698	1
FGRAMPH1_01G11119	FGRAMPH1_01T11119	1.31565	3.55393	Promoter	2	3036554	3038548	1995	2
FGRAMPH1_01G11131	FGRAMPH1_01T11131	1.72186	8.19781	Promoter	2	3048632	3048933	302	2

FGRAMPH1_01G11139	FGRAMPH1_01T11139	1.82598	10.43067	Promoter	2	3066996	3067508	513	1
FGRAMPH1_01G11151	FGRAMPH1_01T11151	1.4708	5.46304	Promoter	2	3083252	3085616	2365	1
FGRAMPH1_01G11155	FGRAMPH1_01T11155	1.76118	9.27263	Promoter	2	3089657	3094313	4657	2
ENSRNA049513096	ENSRNA049513096-T1	2.28489	23.20419	Promoter	2	3111486	3111605	120	1
FGRAMPH1_01G11193	FGRAMPH1_01T11193	1.71059	11.69065	Promoter	2	3143070	3143405	336	2
FGRAMPH1_01G11195	FGRAMPH1_01T11195	1.97652	16.70647	Exon	2	3145094	3145743	650	2
FGRAMPH1_01G11197	FGRAMPH1_01T11197	1.27013	3.4406	Promoter	2	3150756	3151665	910	1
FGRAMPH1_01G11199	FGRAMPH1_01T11199	1.4586	5.34736	Promoter	2	3153053	3153339	287	2
FGRAMPH1_01G11217	FGRAMPH1_01T11217	1.73704	9.35217	UTR	2	3180545	3181554	1010	2
FGRAMPH1_01G11219	FGRAMPH1_01T11219	1.65426	7.86588	Promoter	2	3182180	3185381	3202	1
FGRAMPH1_01G11221	FGRAMPH1_01T11221	1.75497	9.94556	Promoter	2	3185612	3187762	2151	1
FGRAMPH1_01G11231	FGRAMPH1_01T11231	1.83313	12.00628	Promoter	2	3199561	3202959	3399	2
FGRAMPH1_01G11261	FGRAMPH1_01T11261	1.93277	12.25882	Promoter	2	3245077	3245353	277	2

FGRAMPH1_01G11269	FGRAMPH1_01T11269	2.33014	23.29179	Promoter	2	3257823	3258098	276	2
FGRAMPH1_01G11271	FGRAMPH1_01T11271	1.31941	3.38355	UTR	2	3258442	3261304	2863	1
FGRAMPH1_01G11273	FGRAMPH1_01T11273	1.99159	13.82513	Promoter	2	3261726	3264501	2776	1
ENSRNA049513331	ENSRNA049513331-T1	2.66039	32.03953	Promoter	2	3267145	3267264	120	1
FGRAMPH1_01G11285	FGRAMPH1_01T11285	1.31406	3.37367	Exon	2	3340455	3341284	830	1
FGRAMPH1_01G11287	FGRAMPH1_01T11287	1.59503	7.2531	Promoter	2	3342165	3345385	3221	1
FGRAMPH1_01G11289	FGRAMPH1_01T11289	2.20769	20.70331	Promoter	2	3345643	3347191	1549	2
FGRAMPH1_01G11295	FGRAMPH1_01T11295	2.13313	16.99246	Promoter	2	3350800	3352593	1794	2
FGRAMPH1_01G11297	FGRAMPH1_01T11297	1.75866	8.92723	Promoter	2	3356977	3357650	674	1
FGRAMPH1_01G11313	FGRAMPH1_01T11313	1.81903	10.27481	Promoter	2	3376628	3380223	3596	1
FGRAMPH1_01G11321	FGRAMPH1_01T11321	1.95386	12.85253	Promoter	2	3389810	3390103	294	2
FGRAMPH1_01G11323	FGRAMPH1_01T11323	1.81568	10.72464	Promoter	2	3393360	3394502	1143	1
ENSRNA049559910	ENSRNA049559910-T1	1.96075	15.21453	Promoter	2	3396295	3396385	91	1

FGRAMPH1_01G11351	FGRAMPH1_01T11351	1.60484	6.51052	Promoter	2	3437977	3438762	786	2
FGRAMPH1_01G11353	FGRAMPH1_01T11353	1.83503	11.39512	Promoter	2	3439142	3439551	410	2
FGRAMPH1_01G11361	FGRAMPH1_01T11361	1.57512	8.19911	Promoter	2	3451541	3454449	2909	1
FGRAMPH1_01G11375	FGRAMPH1_01T11375	2.39676	24.24705	Intergenic	2	3467999	3468656	658	1
FGRAMPH1_01G11377	FGRAMPH1_01T11377	2.43894	25.42153	Promoter	2	3472167	3474326	2160	1
FGRAMPH1_01G11391	FGRAMPH1_01T11391	1.5299	5.86563	Promoter	2	3495471	3496868	1398	2
FGRAMPH1_01G11399	FGRAMPH1_01T11399	1.98549	13.45803	Promoter	2	3503826	3506425	2600	1
FGRAMPH1_01G11405	FGRAMPH1_01T11405	2.16476	18.0144	Promoter	2	3511639	3512541	903	1
FGRAMPH1_01G11415	FGRAMPH1_01T11415	2.73421	34.66857	Promoter	2	3521793	3523488	1696	1
FGRAMPH1_01G11417	FGRAMPH1_01T11417	1.66834	8.30045	Promoter	2	3523778	3524395	618	1
FGRAMPH1_01G11435	FGRAMPH1_01T11435	1.66913	7.23732	Promoter	2	3551324	3552100	777	1
FGRAMPH1_01G11441	FGRAMPH1_01T11441	1.62695	6.55252	Promoter	2	3556402	3557835	1434	2
FGRAMPH1_01G11445	FGRAMPH1_01T11445	2.81857	37.37431	Promoter	2	3560654	3560921	268	1

FGRAMPH1_01G11453	FGRAMPH1_01T11453	1.36479	3.96005	Promoter	2	3571849	3572817	969	1
FGRAMPH1_01G11489	FGRAMPH1_01T11489	1.70414	8.181	Promoter	2	3601355	3601897	543	1
ENSRNA049512858	ENSRNA049512858-T1	2.66039	32.03953	Promoter	2	3649760	3649879	120	1
FGRAMPH1_01G11555	FGRAMPH1_01T11555	1.70077	7.71086	Promoter	2	3678046	3680044	1999	1
FGRAMPH1_01G11559	FGRAMPH1_01T11559	1.90113	11.67704	Promoter	2	3682962	3684925	1964	2
FGRAMPH1_01G11567	FGRAMPH1_01T11567	1.5215	4.88081	Promoter	2	3699075	3700452	1378	2
FGRAMPH1_01G11571	FGRAMPH1_01T11571	2.6162	30.71375	Promoter	2	3706302	3707114	813	2
FGRAMPH1_01G11573	FGRAMPH1_01T11573	2.55494	28.6574	Promoter	2	3710307	3712886	2580	1
FGRAMPH1_01G11585	FGRAMPH1_01T11585	2.06985	15.66799	Promoter	2	3723984	3724726	743	2
FGRAMPH1_01G11589	FGRAMPH1_01T11589	1.84786	12.68049	Intergenic	2	3728285	3728413	129	1
FGRAMPH1_01G11591	FGRAMPH1_01T11591	1.45504	4.24061	Promoter	2	3729331	3730845	1515	1
FGRAMPH1_01G11631	FGRAMPH1_01T11631	1.83905	11.10505	Intergenic	2	3772699	3772892	194	2
FGRAMPH1_01G11643	FGRAMPH1_01T11643	1.95386	12.85253	Promoter	2	3784030	3786187	2158	1

ENSRNA049512548	ENSRNA049512548-T1	2.50029	27.38845	Promoter	2	3793716	3793835	120	2
ENSRNA049561620	ENSRNA049561620-T1	2.43894	25.42153	Promoter	2	3797711	3797782	72	2
FGRAMPH1_01G11661	FGRAMPH1_01T11661	1.59532	5.89894	Exon	2	3800934	3802649	1716	1
FGRAMPH1_01G11663	FGRAMPH1_01T11663	2.03832	15.18776	Promoter	2	3803824	3804644	821	1
FGRAMPH1_01G11671	FGRAMPH1_01T11671	1.60529	8.39176	Promoter	2	3812182	3813662	1481	2
FGRAMPH1_01G11689	FGRAMPH1_01T11689	2.58335	31.89267	Promoter	2	3834568	3836040	1473	2
ENSRNA049513128	ENSRNA049513128-T1	2.49167	27.0208	Promoter	2	3849777	3849896	120	2
FGRAMPH1_01G11725	FGRAMPH1_01T11725	1.90113	11.67704	Promoter	2	3865591	3867408	1818	2
ENSRNA049559938	ENSRNA049559938-T1	2.27272	22.01315	Promoter	2	3896816	3896916	101	1
FGRAMPH1_01G11757	FGRAMPH1_01T11757	2.24912	20.12992	Promoter	2	3903954	3906211	2258	1
FGRAMPH1_01G11835	FGRAMPH1_01T11835	2.14367	17.33041	Promoter	2	3976595	3978528	1934	2
FGRAMPH1_01G11837	FGRAMPH1_01T11837	1.69022	7.4724	Exon	2	3979637	3981186	1550	1
FGRAMPH1_01G11841	FGRAMPH1_01T11841	1.62695	6.55252	Promoter	2	3983897	3985596	1700	2

FGRAMPH1_01G11857	FGRAMPH1_01T11857	2.22803	19.41428	Promoter	2	3999360	4001111	1752	1
FGRAMPH1_01G11885	FGRAMPH1_01T11885	1.67493	10.41781	Promoter	2	4029755	4030732	978	2
FGRAMPH1_01G11895	FGRAMPH1_01T11895	1.71131	7.95267	Promoter	2	4042694	4045114	2421	1
FGRAMPH1_01G11897	FGRAMPH1_01T11897	1.59532	5.89894	Promoter	2	4045215	4047274	2060	2
FGRAMPH1_01G11921	FGRAMPH1_01T11921	1.85895	10.82712	Promoter	2	4070955	4072809	1855	2
FGRAMPH1_01G11953	FGRAMPH1_01T11953	1.71131	7.95267	Promoter	2	4104342	4105226	885	2
FGRAMPH1_01G11955	FGRAMPH1_01T11955	1.91717	14.37537	Promoter	2	4106303	4106534	232	1
FGRAMPH1_01G11957	FGRAMPH1_01T11957	2.57603	29.48946	Promoter	2	4106796	4108446	1651	1
ENSRNA049559960	ENSRNA049559960-T1	2.61821	30.75447	Promoter	2	4108601	4108672	72	1
FGRAMPH1_01G11979	FGRAMPH1_01T11979	1.81677	10.00496	Promoter	2	4135351	4136779	1429	2
FGRAMPH1_01G12007	FGRAMPH1_01T12007	1.57311	5.92936	Promoter	2	4164278	4164925	648	2
FGRAMPH1_01G12019	FGRAMPH1_01T12019	1.58733	7.15882	Promoter	2	4176156	4178753	2598	1
FGRAMPH1_01G12033	FGRAMPH1_01T12033	1.88004	11.39068	Promoter	2	4191475	4191660	186	2

FGRAMPH1_01G12109	FGRAMPH1_01T12109	1.58644	9.08315	Promoter	2	4275610	4276979	1370	1
FGRAMPH1_01G12125	FGRAMPH1_01T12125	1.69022	7.4724	Promoter	2	4297312	4298313	1002	1
FGRAMPH1_01G12161	FGRAMPH1_01T12161	1.66785	7.21997	Promoter	2	4337259	4337758	500	1
FGRAMPH1_01G12207	FGRAMPH1_01T12207	1.85895	10.82712	Promoter	2	4393362	4395482	2121	2
FGRAMPH1_01G12217	FGRAMPH1_01T12217	2.04102	18.72945	Promoter	2	4405400	4405729	330	2
FGRAMPH1_01G12219	FGRAMPH1_01T12219	1.96698	13.9822	Exon	2	4406953	4408949	1997	1
ENSRNA049559984	ENSRNA049559984-T1	2.70257	33.34432	Promoter	2	4431045	4431127	83	1
FGRAMPH1_01G12237	FGRAMPH1_01T12237	1.99604	13.76516	Promoter	2	4432959	4435013	2055	1
FGRAMPH1_01G12245	FGRAMPH1_01T12245	1.98672	14.2859	Promoter	2	4441655	4443218	1564	2
FGRAMPH1_01G12261	FGRAMPH1_01T12261	1.28738	3.10493	Promoter	2	4455985	4458297	2313	2
FGRAMPH1_01G12263	FGRAMPH1_01T12263	2.3351	26.25159	Promoter	2	4458590	4459390	801	2
ENSRNA049560009	ENSRNA049560009-T1	2.40098	28.24835	Promoter	2	4460188	4460277	90	1
FGRAMPH1_01G12303	FGRAMPH1_01T12303	1.59532	5.89894	Promoter	2	4496988	4499218	2231	2

FGRAMPH1_01G12359	FGRAMPH1_01T12359	1.9863	18.02951	Promoter	2	4554202	4557916	3715	2
ENSRNA049561584	ENSRNA049561584-T1	2.48112	26.61745	Promoter	2	4580330	4580423	94	2
FGRAMPH1_01G12381	FGRAMPH1_01T12381	1.76404	8.95294	Promoter	2	4580809	4582261	1453	2
ENSRNA049560055	ENSRNA049560055-T1	2.6393	31.60897	Promoter	2	4587894	4587975	82	1
FGRAMPH1_01G12389	FGRAMPH1_01T12389	1.3117	3.80654	Promoter	2	4591507	4593062	1556	2
FGRAMPH1_01G12391	FGRAMPH1_01T12391	1.44671	4.20283	Intergenic	2	4593475	4593837	363	1
FGRAMPH1_01G12395	FGRAMPH1_01T12395	1.78513	9.21114	Promoter	2	4596764	4597072	309	1
FGRAMPH1_01G12409	FGRAMPH1_01T12409	1.65474	7.07824	Promoter	2	4611344	4613202	1859	1
FGRAMPH1_01G12415	FGRAMPH1_01T12415	1.52822	5.78832	Promoter	2	4621220	4623631	2412	2
FGRAMPH1_01G12421	FGRAMPH1_01T12421	1.68836	9.56241	Promoter	2	4630975	4633085	2111	1
ENSRNA049560084	ENSRNA049560084-T1	2.54439	28.6574	Promoter	2	4674532	4674629	98	1
FGRAMPH1_01G12473	FGRAMPH1_01T12473	1.69022	7.4724	Promoter	2	4687834	4690188	2355	2
FGRAMPH1_01G12477	FGRAMPH1_01T12477	1.70077	7.71086	Promoter	2	4693252	4694968	1717	2

FGRAMPH1_01G12489	FGRAMPH1_01T12489	1.93277	12.25882	Promoter	2	4707691	4710591	2901	2
FGRAMPH1_01G12491	FGRAMPH1_01T12491	1.7529	12.34476	Promoter	2	4711737	4712863	1127	2
FGRAMPH1_01G12493	FGRAMPH1_01T12493	2.53385	28.2448	UTR	2	4714296	4715868	1573	2
FGRAMPH1_01G12495	FGRAMPH1_01T12495	1.88847	13.12189	Promoter	2	4716468	4718316	1849	2
FGRAMPH1_01G12521	FGRAMPH1_01T12521	1.77459	9.21114	Promoter	2	4740154	4742384	2231	2
FGRAMPH1_01G12525	FGRAMPH1_01T12525	2.06985	15.66799	Promoter	2	4743683	4745004	1322	1
FGRAMPH1_01G12527	FGRAMPH1_01T12527	1.55314	5.27724	Intergenic	2	4745890	4746429	540	2
ENSRNA049512576	ENSRNA049512576-T1	2.64984	31.60897	Promoter	2	4747762	4747881	120	1
ENSRNA049561571	ENSRNA049561571-T1	2.55494	28.6574	Promoter	2	4749714	4749803	90	2
ENSRNA049561507	ENSRNA049561507-T1	2.70257	33.34432	Promoter	2	4752022	4752095	74	2
FGRAMPH1_01G12557	FGRAMPH1_01T12557	1.38089	4.53928	Promoter	2	4771829	4772309	481	1
ENSRNA049560125	ENSRNA049560125-T1	2.46003	25.8178	Promoter	2	4781458	4781560	103	1
FGRAMPH1_01G12573	FGRAMPH1_01T12573	1.65024	6.92932	Promoter	2	4790974	4792485	1512	1

FGRAMPH1_01G12575	FGRAMPH1_01T12575	1.64255	7.08496	Promoter	2	4793731	4795466	1736	2
FGRAMPH1_01G12593	FGRAMPH1_01T12593	1.62803	9.71617	Exon	2	4813039	4815840	2802	1
FGRAMPH1_01G12599	FGRAMPH1_01T12599	1.80622	9.73718	Promoter	2	4819591	4822212	2622	2
FGRAMPH1_01G12605	FGRAMPH1_01T12605	1.40432	3.60276	Promoter	2	4831525	4834763	3239	1
ENSRNA049513248	ENSRNA049513248-T1	2.10149	16.32472	Promoter	2	4838530	4838649	120	1
ENSRNA049561480	ENSRNA049561480-T1	2.37567	23.47615	Promoter	2	4843048	4843143	96	2
ENSRNA049560140	ENSRNA049560140-T1	2.45431	28.69126	Promoter	2	4852277	4852348	72	1
ENSRNA049512668	ENSRNA049512668-T1	2.28076	20.85586	Promoter	2	4869264	4869383	120	1
FGRAMPH1_01G12663	FGRAMPH1_01T12663	2.48638	29.20447	Promoter	2	4900688	4902890	2203	1
FGRAMPH1_01G12681	FGRAMPH1_01T12681	1.53204	5.0772	Promoter	2	4921274	4922467	1194	1
ENSRNA049560177	ENSRNA049560177-T1	2.34403	22.71505	Promoter	2	4926678	4926759	82	1
FGRAMPH1_01G12705	FGRAMPH1_01T12705	1.74548	8.7842	Promoter	2	4943773	4945620	1848	2
ENSRNA049561460	ENSRNA049561460-T1	2.45898	26.37422	Promoter	2	4947643	4947738	96	2

ENSRNA049561427	ENSRNA049561427-T1	2.50221	27.42648	Promoter	2	4969617	4969688	72	2
FGRAMPH1_01G12739	FGRAMPH1_01T12739	1.54214	7.97944	Promoter	2	4982018	4982431	414	1
FGRAMPH1_01G12741	FGRAMPH1_01T12741	2.02124	15.77202	Promoter	2	4983776	4986824	3049	1
ENSRNA049512423	ENSRNA049512423-T1	2.72366	34.22501	Promoter	2	4987856	4987975	120	2
FGRAMPH1_01G12759	FGRAMPH1_01T12759	1.95386	12.85253	Promoter	2	5002051	5004693	2643	2
ENSRNA049561405	ENSRNA049561405-T1	2.47057	26.21645	Promoter	2	5027893	5027978	86	2
FGRAMPH1_01G12779	FGRAMPH1_01T12779	1.85895	10.82712	Promoter	2	5028500	5029999	1500	1
FGRAMPH1_01G12781	FGRAMPH1_01T12781	1.95221	13.5107	Promoter	2	5031663	5033144	1482	1
FGRAMPH1_01G12787	FGRAMPH1_01T12787	1.74295	8.44624	Promoter	2	5035919	5037851	1933	2
FGRAMPH1_01G12789	FGRAMPH1_01T12789	1.64838	6.99132	Promoter	2	5038478	5039962	1485	2
ENSRNA049512603	ENSRNA049512603-T1	2.35458	23.09437	Promoter	2	5050999	5051118	120	1
ENSRNA049561375	ENSRNA049561375-T1	1.92222	12.25882	Promoter	2	5056168	5056249	82	2
FGRAMPH1_01G12823	FGRAMPH1_01T12823	1.72186	8.19781	Promoter	2	5077709	5080191	2483	2

FGRAMPH1_01G12839	FGRAMPH1_01T12839	1.88091	11.23952	Promoter	2	5108548	5109148	601	1
FGRAMPH1_01G12859	FGRAMPH1_01T12859	1.54259	5.27724	Promoter	2	5127662	5130657	2996	2
FGRAMPH1_01G12861	FGRAMPH1_01T12861	1.35171	3.16219	Promoter	2	5134720	5136739	2020	1
FGRAMPH1_01G12889	FGRAMPH1_01T12889	1.56368	5.48089	Promoter	2	5162553	5164310	1758	2
ENSRNA049512864	ENSRNA049512864-T1	2.7553	35.11426	Promoter	2	5169930	5170049	120	1
FGRAMPH1_01G12903	FGRAMPH1_01T12903	2.72177	34.86292	Promoter	2	5178102	5178545	444	1
FGRAMPH1_01G12917	FGRAMPH1_01T12917	1.95163	16.61678	Promoter	2	5194293	5197547	3255	1
FGRAMPH1_01G12939	FGRAMPH1_01T12939	2.00658	14.07517	Promoter	2	5218047	5220929	2883	1
FGRAMPH1_01G12969	FGRAMPH1_01T12969	1.83786	10.2759	Promoter	2	5246395	5247481	1087	2
FGRAMPH1_01G12983	FGRAMPH1_01T12983	1.75477	9.17578	Promoter	2	5260956	5264677	3722	1
FGRAMPH1_01G13013	FGRAMPH1_01T13013	1.38845	3.94135	Promoter	2	5290559	5291838	1280	1
ENSRNA049561338	ENSRNA049561338-T1	2.8502	38.29288	Promoter	2	5294917	5295007	91	2
FGRAMPH1_01G13021	FGRAMPH1_01T13021	2.44948	25.8178	Promoter	2	5296231	5299713	3483	2

ENSRNA049560219	ENSRNA049560219-T1	2.28076	20.85586	Promoter	2	5322508	5322590	83	1
ENSRNA049512748	ENSRNA049512748-T1	2.25211	21.10731	Promoter	2	5326208	5326327	120	2
ENSRNA049561311	ENSRNA049561311-T1	2.57603	29.48946	Promoter	2	5344842	5344931	90	2
FGRAMPH1_01G13097	FGRAMPH1_01T13097	1.8484	10.54995	Promoter	2	5386480	5388213	1734	1
FGRAMPH1_01G13145	FGRAMPH1_01T13145	1.60984	6.66109	Promoter	2	5443401	5445434	2034	2
FGRAMPH1_01G13147	FGRAMPH1_01T13147	1.52468	7.88083	Exon	2	5446647	5447858	1212	2
ENSRNA049512622	ENSRNA049512622-T1	2.46003	25.8178	Promoter	2	5450903	5451022	120	1
FGRAMPH1_01G13153	FGRAMPH1_01T13153	2.01713	14.38806	Promoter	2	5453555	5454589	1035	1
FGRAMPH1_01G13157	FGRAMPH1_01T13157	2.01713	14.38806	Promoter	2	5456531	5458059	1529	2
FGRAMPH1_01G13173	FGRAMPH1_01T13173	1.86373	13.34762	UTR	2	5471158	5472215	1058	1
FGRAMPH1_01G13175	FGRAMPH1_01T13175	1.55814	5.41294	Promoter	2	5473010	5473690	681	2
FGRAMPH1_01G13179	FGRAMPH1_01T13179	2.20525	19.0297	Intergenic	2	5478887	5479087	201	1
FGRAMPH1_01G13185	FGRAMPH1_01T13185	2.34522	27.16869	Promoter	2	5484000	5486039	2040	2

ENSRNA049561226	ENSRNA049561226-T1	2.28076	20.85586	Promoter	2	5498766	5498859	94	2
FGRAMPH1_01G13199	FGRAMPH1_01T13199	1.58477	5.89894	Promoter	2	5500620	5502487	1868	2
ENSRNA049561194	ENSRNA049561194-T1	2.3124	21.592	Promoter	2	5503261	5503353	93	2
FGRAMPH1_01G13203	FGRAMPH1_01T13203	1.85895	10.82712	Promoter	2	5508084	5509775	1692	2
FGRAMPH1_01G13205	FGRAMPH1_01T13205	1.43367	4.05952	Promoter	2	5511455	5512032	578	2
ENSRNA049561177	ENSRNA049561177-T1	2.58657	29.90888	Promoter	2	5513012	5513101	90	2
ENSRNA049561155	ENSRNA049561155-T1	2.67094	32.47228	Promoter	2	5518246	5518344	99	2
ENSRNA049561116	ENSRNA049561116-T1	2.54439	28.6574	Promoter	2	5519000	5519071	72	2
FGRAMPH1_01G13217	FGRAMPH1_01T13217	1.8944	11.88788	Promoter	2	5521535	5523590	2056	1
ENSRNA049512635	ENSRNA049512635-T1	1.97495	13.45803	Promoter	2	5541503	5541622	120	1
ENSRNA049560233	ENSRNA049560233-T1	2.52092	27.97779	Promoter	2	5549911	5550020	110	1
FGRAMPH1_01G13247	FGRAMPH1_01T13247	2.01713	14.38806	Promoter	2	5555226	5556775	1550	2
ENSRNA049560263	ENSRNA049560263-T1	2.47057	26.21645	Promoter	2	5560926	5561021	96	1

ENSRNA049561078	ENSRNA049561078-T1	2.44205	26.77021	Promoter	2	5583177	5583259	83	2
ENSRNA049513161	ENSRNA049513161-T1	2.02767	14.38806	Promoter	2	5636571	5636690	120	2
FGRAMPH1_01G13325	FGRAMPH1_01T13325	1.89515	11.58275	Promoter	2	5642156	5644205	2050	2
FGRAMPH1_01G13331	FGRAMPH1_01T13331	1.73073	8.42226	Promoter	2	5650332	5652940	2609	2
FGRAMPH1_01G13333	FGRAMPH1_01T13333	1.77511	9.90689	Promoter	2	5654672	5655961	1290	1
FGRAMPH1_01G13339	FGRAMPH1_01T13339	1.78226	10.50521	Promoter	2	5664469	5666739	2271	1
ENSRNA049512912	ENSRNA049512912-T1	2.67094	32.47228	Promoter	2	5674473	5674592	120	1
FGRAMPH1_01G13401	FGRAMPH1_01T13401	1.95386	12.85253	Promoter	2	5725588	5726919	1332	1
FGRAMPH1_01G13409	FGRAMPH1_01T13409	2.6437	32.12572	Promoter	2	5733296	5733877	582	1
FGRAMPH1_01G13411	FGRAMPH1_01T13411	2.4073	24.63614	Promoter	2	5734094	5736175	2082	2
FGRAMPH1_01G13413	FGRAMPH1_01T13413	1.96254	13.47049	Promoter	2	5737889	5738176	288	1
FGRAMPH1_01G13431	FGRAMPH1_01T13431	2.23857	21.92068	Promoter	2	5761183	5763429	2247	2
ENSRNA049560294	ENSRNA049560294-T1	2.67094	32.47228	Promoter	2	5764030	5764101	72	1

ENSRNA049560341	ENSRNA049560341-T1	2.47057	26.21645	Promoter	2	5769981	5770080	100	1
ENSRNA049560347	ENSRNA049560347-T1	2.24912	20.12992	Promoter	2	5775128	5775199	72	1
FGRAMPH1_01G13455	FGRAMPH1_01T13455	1.31604	3.48365	Exon	2	5793976	5794719	744	2
FGRAMPH1_01G13461	FGRAMPH1_01T13461	1.90113	11.67704	Promoter	2	5801001	5802524	1524	1
FGRAMPH1_01G13465	FGRAMPH1_01T13465	2.58657	29.90888	Promoter	2	5805619	5807495	1877	2
FGRAMPH1_01G13479	FGRAMPH1_01T13479	2.34403	22.71505	Promoter	2	5822555	5823121	567	1
FGRAMPH1_01G13481	FGRAMPH1_01T13481	1.35628	3.25755	Promoter	2	5823459	5826173	2715	1
ENSRNA049512950	ENSRNA049512950-T1	2.76584	35.56207	Promoter	2	5828448	5828567	120	1
ENSRNA049560380	ENSRNA049560380-T1	2.77639	36.01199	Promoter	2	5851701	5851785	85	1
FGRAMPH1_01G13531	FGRAMPH1_01T13531	1.55088	7.20648	Promoter	2	5879573	5879899	327	2
FGRAMPH1_01G13535	FGRAMPH1_01T13535	1.62571	6.53616	Promoter	2	5880813	5883497	2685	2
ENSRNA049561065	ENSRNA049561065-T1	1.94331	12.55419	Promoter	2	5900165	5900238	74	2
FGRAMPH1_01G13555	FGRAMPH1_01T13555	2.21174	20.19388	Promoter	2	5900769	5902577	1809	2

FGRAMPH1_01G13581	FGRAMPH1_01T13581	2.0483	17.18208	Exon	2	5932150	5936818	4669	1
FGRAMPH1_01G13583	FGRAMPH1_01T13583	2.38621	23.86038	Exon	2	5937948	5939699	1752	1
FGRAMPH1_01G13587	FGRAMPH1_01T13587	1.9644	13.15382	Promoter	2	5942021	5943911	1891	1
ENSRNA049561040	ENSRNA049561040-T1	2.57206	30.83143	Promoter	2	5955176	5955275	100	2
FGRAMPH1_01G13601	FGRAMPH1_01T13601	2.17543	18.48849	Promoter	2	5957213	5959762	2550	2
FGRAMPH1_01G13603	FGRAMPH1_01T13603	1.59299	9.13999	Promoter	2	5961222	5962508	1287	2
FGRAMPH1_01G13605	FGRAMPH1_01T13605	1.93972	15.14546	Promoter	2	5964891	5965763	873	1
FGRAMPH1_01G13613	FGRAMPH1_01T13613	2.03915	19.06852	Promoter	2	5976554	5976691	138	2
FGRAMPH1_01G13619	FGRAMPH1_01T13619	1.36198	4.74741	UTR	2	5979315	5979733	419	2
FGRAMPH1_01G13623	FGRAMPH1_01T13623	2.14948	18.44609	Promoter	2	5981196	5983559	2364	1
FGRAMPH1_01G13625	FGRAMPH1_01T13625	1.57026	6.89272	Promoter	2	5983664	5986123	2460	2
ENSRNA049560421	ENSRNA049560421-T1	1.91806	16.63122	Promoter	2	6002398	6002480	83	1
FGRAMPH1_01G13647	FGRAMPH1_01T13647	2.03822	14.70381	Promoter	2	6006180	6008272	2093	1

ENSRNA049561022	ENSRNA049561022-T1	2.48112	26.61745	Promoter	2	6010840	6010939	100	2
ENSRNA049560438	ENSRNA049560438-T1	1.84192	11.80473	Promoter	2	6026421	6026493	73	1
FGRAMPH1_01G13683	FGRAMPH1_01T13683	1.99323	16.91956	UTR	2	6039967	6040977	1011	1
FGRAMPH1_01G13685	FGRAMPH1_01T13685	2.20155	22.20001	Promoter	2	6041066	6043216	2151	2
ENSRNA049512499	ENSRNA049512499-T1	2.56548	29.0723	Promoter	2	6047117	6047236	120	2
ENSRNA049560997	ENSRNA049560997-T1	2.54439	28.6574	Promoter	2	6069031	6069124	94	2
FGRAMPH1_01G13713	FGRAMPH1_01T13713	2.29131	21.22266	Promoter	2	6069426	6069776	351	2
ENSRNA049560959	ENSRNA049560959-T1	2.56548	29.0723	Promoter	2	6072562	6072652	91	2
FGRAMPH1_01G13733	FGRAMPH1_01T13733	1.98397	13.43306	Promoter	2	6085749	6088447	2699	1
FGRAMPH1_01G13741	FGRAMPH1_01T13741	1.30975	3.3543	Promoter	2	6096263	6098035	1773	2
FGRAMPH1_01G13745	FGRAMPH1_01T13745	1.41419	5.7408	UTR	2	6101152	6101487	336	2
FGRAMPH1_01G13757	FGRAMPH1_01T13757	1.94033	14.52772	Promoter	2	6110827	6113039	2213	2
FGRAMPH1_01G13775	FGRAMPH1_01T13775	1.70077	7.71086	Promoter	2	6130915	6132777	1863	2

ENSRNA049512352	ENSRNA049512352-T1	1.87062	16.50861	Promoter	2	6141056	6141326	271	2
ENSRNA049512978	ENSRNA049512978-T1	2.76584	35.56207	Promoter	2	6142237	6142356	120	1
FGRAMPH1_01G13799	FGRAMPH1_01T13799	2.04069	15.16563	Promoter	2	6153747	6156165	2419	1
ENSRNA049560464	ENSRNA049560464-T1	2.6393	31.60897	Promoter	2	6162490	6162572	83	1
ENSRNA049560508	ENSRNA049560508-T1	2.66237	32.72871	Promoter	2	6163388	6163481	94	1
FGRAMPH1_01G13807	FGRAMPH1_01T13807	1.86153	14.76452	Promoter	2	6165590	6167125	1536	1
FGRAMPH1_01G13819	FGRAMPH1_01T13819	1.93277	12.25882	Promoter	2	6180321	6182841	2521	2
FGRAMPH1_01G13849	FGRAMPH1_01T13849	1.40424	3.93261	Promoter	2	6215267	6216564	1298	1
FGRAMPH1_01G13873	FGRAMPH1_01T13873	1.8786	11.36803	Promoter	2	6236614	6238392	1779	2
FGRAMPH1_01G13877	FGRAMPH1_01T13877	2.4073	24.63614	Promoter	2	6239994	6240578	585	1
FGRAMPH1_01G13883	FGRAMPH1_01T13883	1.80622	9.73718	Promoter	2	6247387	6249874	2488	2
FGRAMPH1_01G13917	FGRAMPH1_01T13917	2.20458	22.8784	Promoter	2	6280508	6281881	1374	1
FGRAMPH1_01G13931	FGRAMPH1_01T13931	2.14461	20.1514	Promoter	2	6298246	6300207	1962	2

FGRAMPH1_01G13965	FGRAMPH1_01T13965	2.12258	16.65722	Promoter	2	6342111	6343529	1419	1
ENSRNA049560560	ENSRNA049560560-T1	2.35458	23.09437	Promoter	2	6352988	6353059	72	1
FGRAMPH1_01G13997	FGRAMPH1_01T13997	2.14367	17.33041	Promoter	2	6376947	6378251	1305	2
FGRAMPH1_01G14015	FGRAMPH1_01T14015	1.40547	4.71319	Exon	2	6396892	6397098	207	1
FGRAMPH1_01G14101	FGRAMPH1_01T14101	1.74295	8.44624	Promoter	2	6474345	6477544	3200	1
FGRAMPH1_01G14105	FGRAMPH1_01T14105	1.71131	7.95267	Promoter	2	6478845	6481965	3121	1
FGRAMPH1_01G14123	FGRAMPH1_01T14123	1.89058	11.39068	Promoter	2	6494267	6497044	2778	2
ENSRNA049512700	ENSRNA049512700-T1	2.0804	15.66799	Intergenic	2	6516280	6516399	120	2
FGRAMPH1_01G14185	FGRAMPH1_01T14185	1.81677	10.00496	Promoter	2	6586841	6588720	1880	2
ENSRNA049560579	ENSRNA049560579-T1	2.04585	16.22698	Promoter	2	6605435	6605516	82	1
FGRAMPH1_01G14211	FGRAMPH1_01T14211	1.51352	5.16235	Promoter	2	6613533	6615609	2077	2
FGRAMPH1_01G14215	FGRAMPH1_01T14215	1.73677	10.08839	Promoter	2	6620648	6623277	2630	2
FGRAMPH1_01G14217	FGRAMPH1_01T14217	1.4281	5.29298	Exon	2	6625186	6625636	451	2

ENSRNA049560937	ENSRNA049560937-T1	2.39164	26.45337	Promoter	2	6630281	6630353	73	2
FGRAMPH1_01G14221	FGRAMPH1_01T14221	2.06196	15.60065	Promoter	2	6631047	6632672	1626	2
ENSRNA049560606	ENSRNA049560606-T1	2.58657	29.90888	Promoter	2	6678600	6678686	87	1
FGRAMPH1_01G14279	FGRAMPH1_01T14279	1.46993	5.32574	Promoter	2	6681814	6683579	1766	2
FGRAMPH1_01G14321	FGRAMPH1_01T14321	1.51101	6.53147	Promoter	2	6736612	6740838	4227	2
FGRAMPH1_01G14347	FGRAMPH1_01T14347	1.97971	13.58603	Exon	2	6771232	6774660	3429	1
ENSRNA049560654	ENSRNA049560654-T1	1.75873	11.34089	Promoter	2	6793524	6793617	94	1
FGRAMPH1_01G14365	FGRAMPH1_01T14365	1.44196	4.52458	Promoter	2	6799751	6801237	1487	1
FGRAMPH1_01G14387	FGRAMPH1_01T14387	1.6757	7.4957	Intergenic	2	6822800	6823255	456	1
FGRAMPH1_01G14391	FGRAMPH1_01T14391	1.95912	15.74377	Promoter	2	6826212	6826559	348	2
FGRAMPH1_01G14407	FGRAMPH1_01T14407	1.75256	10.31176	Promoter	2	6843575	6844147	573	1
ENSRNA049560675	ENSRNA049560675-T1	2.57603	29.48946	Promoter	2	6878740	6878838	99	1
FGRAMPH1_01G14439	FGRAMPH1_01T14439	2.67094	32.47228	Promoter	2	6884407	6884587	181	1

FGRAMPH1_01G14441	FGRAMPH1_01T14441	2.05931	15.34379	Promoter	2	6884870	6888009	3140	2
FGRAMPH1_01G14443	FGRAMPH1_01T14443	1.45374	6.56313	Promoter	2	6889237	6889440	204	1
FGRAMPH1_01G14447	FGRAMPH1_01T14447	1.48562	5.1916	Promoter	2	6892428	6894998	2571	2
FGRAMPH1_01G14457	FGRAMPH1_01T14457	1.82659	11.36381	Exon	2	6903789	6904206	418	1
ENSRNA049560892	ENSRNA049560892-T1	2.61841	32.64704	Promoter	2	6915448	6915519	72	2
FGRAMPH1_01G14495	FGRAMPH1_01T14495	1.9644	13.15382	Promoter	2	6950382	6950951	570	1
FGRAMPH1_01G14499	FGRAMPH1_01T14499	1.9644	13.15382	Promoter	2	6958821	6961772	2952	1
FGRAMPH1_01G14501	FGRAMPH1_01T14501	1.84328	10.76602	Promoter	2	6964939	6966714	1776	1
FGRAMPH1_01G14529	FGRAMPH1_01T14529	1.85408	12.42954	Promoter	2	7009014	7013718	4705	1
FGRAMPH1_01G14531	FGRAMPH1_01T14531	1.51305	5.57431	Promoter	2	7014054	7014371	318	1
FGRAMPH1_01G14537	FGRAMPH1_01T14537	1.58986	8.21658	Promoter	2	7021038	7024639	3602	1
FGRAMPH1_01G14565	FGRAMPH1_01T14565	2.05348	18.36837	Intergenic	2	7066215	7066790	576	2
FGRAMPH1_01G14567	FGRAMPH1_01T14567	2.11993	18.99322	Promoter	2	7066973	7067514	542	2

FGRAMPH1_01G14569	FGRAMPH1_01T14569	1.79595	11.77747	Exon	2	7069272	7070649	1378	2
FGRAMPH1_01G14573	FGRAMPH1_01T14573	2.12927	20.82519	UTR	2	7073039	7077477	4439	1
FGRAMPH1_01G14583	FGRAMPH1_01T14583	1.63705	8.06095	Exon	2	7087984	7090021	2038	2
FGRAMPH1_01G14609	FGRAMPH1_01T14609	1.59721	6.70015	Promoter	2	7114859	7115834	976	1
FGRAMPH1_01G14629	FGRAMPH1_01T14629	2.21749	19.06036	Promoter	2	7139600	7140337	738	2
FGRAMPH1_01G14631	FGRAMPH1_01T14631	2.35013	28.13368	Promoter	2	7141838	7143534	1697	1
FGRAMPH1_01G14635	FGRAMPH1_01T14635	1.82731	10.2759	Promoter	2	7145576	7146991	1416	1
FGRAMPH1_01G14665	FGRAMPH1_01T14665	1.48297	5.44533	Promoter	2	7186621	7187562	942	2
FGRAMPH1_01G14675	FGRAMPH1_01T14675	1.34153	3.09014	Promoter	2	7207569	7211720	4152	1
ENSRNA049560870	ENSRNA049560870-T1	2.33684	24.60181	Promoter	2	7212130	7212229	100	2
FGRAMPH1_01G14705	FGRAMPH1_01T14705	1.6375	6.77735	Promoter	2	7252874	7254950	2077	2
FGRAMPH1_01G14707	FGRAMPH1_01T14707	1.70548	10.47209	Promoter	2	7259535	7261562	2028	1
FGRAMPH1_01G14721	FGRAMPH1_01T14721	1.4714	4.71515	Promoter	2	7276673	7280187	3515	1

FGRAMPH1_01G14725	FGRAMPH1_01T14725	2.00658	14.07517	Promoter	2	7283113	7285654	2542	1
FGRAMPH1_01G14737	FGRAMPH1_01T14737	2.17531	18.0144	Promoter	2	7291878	7292671	794	2
FGRAMPH1_01G14739	FGRAMPH1_01T14739	1.60586	6.11329	Exon	2	7293088	7294731	1644	2
FGRAMPH1_01G14749	FGRAMPH1_01T14749	1.92222	12.25882	Promoter	2	7300836	7301498	663	2
FGRAMPH1_01G14751	FGRAMPH1_01T14751	1.80622	9.73718	Exon	2	7302690	7302846	157	1
FGRAMPH1_01G14753	FGRAMPH1_01T14753	2.12258	16.65722	Intergenic	2	7303500	7304000	501	1
FGRAMPH1_01G14773	FGRAMPH1_01T14773	1.35447	3.32783	Promoter	2	7325048	7326638	1591	2
FGRAMPH1_01G14803	FGRAMPH1_01T14803	1.88004	11.39068	Promoter	2	7359019	7360843	1825	2
FGRAMPH1_01G14805	FGRAMPH1_01T14805	1.81677	10.00496	Promoter	2	7363787	7366193	2407	1
FGRAMPH1_01G14811	FGRAMPH1_01T14811	1.30885	3.0199	Promoter	2	7370638	7373032	2395	2
FGRAMPH1_01G14813	FGRAMPH1_01T14813	1.62579	6.93687	Promoter	2	7374186	7376598	2413	1
FGRAMPH1_01G14831	FGRAMPH1_01T14831	2.34403	22.71505	Exon	2	7398259	7399069	811	2
FGRAMPH1_01G14899	FGRAMPH1_01T14899	1.71583	9.58594	UTR	2	7466642	7467499	858	1

FGRAMPH1_01G14901	FGRAMPH1_01T14901	1.62269	7.00779	Promoter	2	7467822	7470740	2919	2
ENSRNA049513612	ENSRNA049513612-T1	1.83583	11.23056	Promoter	2	7473320	7473400	81	1
FGRAMPH1_01G14905	FGRAMPH1_01T14905	2.09532	17.25242	Promoter	2	7474369	7476923	2555	1
FGRAMPH1_01G14927	FGRAMPH1_01T14927	2.23823	19.9026	Promoter	2	7504816	7507523	2708	2
FGRAMPH1_01G14929	FGRAMPH1_01T14929	1.874	12.66952	Promoter	2	7513424	7514453	1030	2
FGRAMPH1_01G14931	FGRAMPH1_01T14931	2.0828	16.50961	UTR	2	7515591	7516500	910	1
FGRAMPH1_01G14937	FGRAMPH1_01T14937	1.88819	12.87794	Promoter	2	7524625	7525423	799	2
FGRAMPH1_01G14939	FGRAMPH1_01T14939	1.64654	10.24799	Promoter	2	7527174	7527674	501	2
FGRAMPH1_01G14941	FGRAMPH1_01T14941	1.97762	15.31795	Promoter	2	7528994	7531514	2521	1
FGRAMPH1_01G14947	FGRAMPH1_01T14947	1.40694	4.67802	Promoter	2	7540122	7543936	3815	1
FGRAMPH1_01G14953	FGRAMPH1_01T14953	1.7423	8.43702	Promoter	2	7552931	7556317	3387	1
FGRAMPH1_01G14957	FGRAMPH1_01T14957	1.91754	13.07675	Promoter	2	7563676	7564089	414	2
ENSRNA049513058	ENSRNA049513058-T1	2.6393	31.60897	Promoter	2	7632988	7633107	120	2

FGRAMPH1_01G15031	FGRAMPH1_01T15031	1.62403	7.4021	Exon	2	7678919	7681703	2785	1
ENSRNA049560842	ENSRNA049560842-T1	2.66039	32.03953	Promoter	2	7683618	7683699	82	2
FGRAMPH1_01G15039	FGRAMPH1_01T15039	1.67968	7.4724	Promoter	2	7692357	7694397	2041	2
ENSRNA049513676	ENSRNA049513676-T1	1.42508	4.53889	Promoter	2	7712364	7712471	108	2
FGRAMPH1_01G15063	FGRAMPH1_01T15063	1.57526	8.25454	Exon	2	7727880	7728679	800	1
FGRAMPH1_01G15081	FGRAMPH1_01T15081	2.10168	19.11599	Promoter	2	7747142	7747360	219	1
FGRAMPH1_01G15083	FGRAMPH1_01T15083	1.54776	7.59519	Promoter	2	7748151	7753288	5138	1
FGRAMPH1_01G15087	FGRAMPH1_01T15087	1.99447	15.1834	Promoter	2	7753720	7754424	705	2
FGRAMPH1_01G15089	FGRAMPH1_01T15089	1.54092	7.39863	Promoter	2	7755013	7757306	2294	2
ENSRNA049512785	ENSRNA049512785-T1	2.27021	20.49161	Promoter	2	7761494	7761613	120	2
ENSRNA049560825	ENSRNA049560825-T1	2.13741	19.73118	Promoter	2	7782102	7782197	96	2
FGRAMPH1_01G15113	FGRAMPH1_01T15113	1.75362	9.10808	Promoter	2	7794256	7797177	2922	1
FGRAMPH1_01G15127	FGRAMPH1_01T15127	2.16476	18.0144	Promoter	2	7812321	7814993	2673	1

FGRAMPH1_01G15153	FGRAMPH1_01T15153	1.64176	7.84545	Promoter	2	7845219	7848247	3029	1
FGRAMPH1_01G15187	FGRAMPH1_01T15187	2.02767	14.38806	Promoter	2	7881462	7884268	2807	1
FGRAMPH1_01G15193	FGRAMPH1_01T15193	1.70714	8.92016	Promoter	2	7888598	7890915	2318	2
FGRAMPH1_01G15195	FGRAMPH1_01T15195	1.81098	12.5067	Promoter	2	7893914	7895446	1533	1
FGRAMPH1_01G15233	FGRAMPH1_01T15233	1.46953	4.27632	Promoter	2	7931168	7933510	2343	2
FGRAMPH1_01G15235	FGRAMPH1_01T15235	2.71969	35.26396	Promoter	2	7935204	7938333	3130	1
ENSRNA049512816	ENSRNA049512816-T1	2.11204	16.65722	Promoter	2	7978287	7978406	120	1
FGRAMPH1_01G15265	FGRAMPH1_01T15265	1.57141	6.15334	Exon	2	7983322	7983633	312	1
FGRAMPH1_01G15281	FGRAMPH1_01T15281	1.62695	6.55252	Promoter	2	8007914	8009559	1646	1
FGRAMPH1_01G15325	FGRAMPH1_01T15325	1.95386	12.85253	Promoter	2	8071306	8073320	2015	2
FGRAMPH1_01G15339	FGRAMPH1_01T15339	1.48438	4.79496	Promoter	2	8095180	8096427	1248	1
ENSRNA049512656	ENSRNA049512656-T1	2.62325	32.96554	Promoter	2	8123092	8123211	120	1
ENSRNA049512712	ENSRNA049512712-T1	1.67968	7.4724	Promoter	2	8128033	8128152	120	1

FGRAMPH1_01G15375	FGRAMPH1_01T15375	2.10149	16.32472	Promoter	2	8139317	8140783	1467	2
FGRAMPH1_01G15387	FGRAMPH1_01T15387	1.61111	6.98297	Promoter	2	8157147	8158891	1745	1
FGRAMPH1_01G15425	FGRAMPH1_01T15425	1.82731	10.2759	Promoter	2	8204782	8205448	667	1
FGRAMPH1_01G15427	FGRAMPH1_01T15427	1.65859	7.00562	Promoter	2	8206195	8213846	7652	1
ENSRNA049512687	ENSRNA049512687-T1	2.04719	14.99574	Promoter	2	8225719	8225838	120	2
FGRAMPH1_01G15483	FGRAMPH1_01T15483	1.65496	7.8934	Promoter	2	8271844	8273131	1288	2
FGRAMPH1_01G15485	FGRAMPH1_01T15485	2.25534	23.35425	Promoter	2	8273961	8277254	3294	2
FGRAMPH1_01G15487	FGRAMPH1_01T15487	1.65764	8.00615	Promoter	2	8280071	8282140	2070	1
ENSRNA049560794	ENSRNA049560794-T1	2.15422	17.67106	Promoter	2	8311313	8311402	90	2
FGRAMPH1_01G15521	FGRAMPH1_01T15521	1.65859	7.00562	Promoter	2	8328212	8329741	1530	2
ENSRNA049560694	ENSRNA049560694-T1	2.47204	27.56455	Promoter	2	8346713	8346786	74	1
FGRAMPH1_01G15559	FGRAMPH1_01T15559	1.88004	11.39068	Promoter	2	8370279	8370974	696	2
FGRAMPH1_01G15577	FGRAMPH1_01T15577	1.6537	7.39913	Promoter	2	8391316	8393341	2026	2

ENSRNA049560767	ENSRNA049560767-T1	2.6684	35.45807	Promoter	2	8393844	8393934	91	2
FGRAMPH1_01G15579	FGRAMPH1_01T15579	1.8484	10.54995	Promoter	2	8395666	8396337	672	2
FGRAMPH1_01G15581	FGRAMPH1_01T15581	1.68535	7.81585	Promoter	2	8397580	8399369	1790	2
FGRAMPH1_01G15583	FGRAMPH1_01T15583	1.44768	3.95439	Promoter	2	8401900	8403612	1713	1
ENSRNA049560712	ENSRNA049560712-T1	2.28136	21.67081	Promoter	2	8446943	8447025	83	1
FGRAMPH1_01G15633	FGRAMPH1_01T15633	2.06015	17.66057	Exon	2	8472231	8472677	447	1
ENSRNA049560738	ENSRNA049560738-T1	2.67094	32.47228	Promoter	2	8545744	8545828	85	2
FGRAMPH1_01G15701	FGRAMPH1_01T15701	1.88004	11.39068	Promoter	2	8552113	8553123	1011	2
FGRAMPH1_01G15725	FGRAMPH1_01T15725	1.57423	5.68813	Promoter	2	8576946	8577503	558	2
FGRAMPH1_01G15771	FGRAMPH1_01T15771	1.59275	6.13221	Promoter	2	8639933	8640910	978	1
FGRAMPH1_01G15877	FGRAMPH1_01T15877	2.22803	19.41428	Promoter	2	8751338	8753309	1972	1
FGRAMPH1_01G15879	FGRAMPH1_01T15879	1.66359	9.9409	Promoter	2	8754153	8755367	1215	1
FGRAMPH1_01G15917	FGRAMPH1_01T15917	1.98549	13.45803	Promoter	2	8810120	8811001	882	2

FGRAMPH1_01G15927	FGRAMPH1_01T15927	2.39676	24.24705	Promoter	2	8821078	8821550	473	1
FGRAMPH1_01G15941	FGRAMPH1_01T15941	2.38621	23.86038	Promoter	2	8833408	8835467	2060	1
FGRAMPH1_01G15949	FGRAMPH1_01T15949	1.67968	7.4724	Promoter	2	8842106	8844954	2849	1
FGRAMPH1_01G16017	FGRAMPH1_01T16017	2.00658	14.07517	Promoter	2	8918897	8920403	1507	1
ENSRNA049511796	ENSRNA049511796-T1	2.47057	26.21645	Promoter	3	69062	69181	120	2
ENSRNA049555945	ENSRNA049555945-T1	2.36512	23.09437	Promoter	3	96768	96839	72	1
FGRAMPH1_01G16179	FGRAMPH1_01T16179	1.55233	5.65968	Promoter	3	140731	142465	1735	2
FGRAMPH1_01G16181	FGRAMPH1_01T16181	1.93277	12.25882	Exon	3	143293	144141	849	1
FGRAMPH1_01G16193	FGRAMPH1_01T16193	2.05063	21.73299	Exon	3	156221	156679	459	2
FGRAMPH1_01G16215	FGRAMPH1_01T16215	1.61641	6.33116	Promoter	3	182382	184511	2130	2
FGRAMPH1_01G16217	FGRAMPH1_01T16217	2.21749	19.06036	Promoter	3	185563	185947	385	2
FGRAMPH1_01G16259	FGRAMPH1_01T16259	1.6375	6.77735	Promoter	3	227265	229666	2402	2
ENSRNA049557942	ENSRNA049557942-T1	2.38621	23.86038	Promoter	3	269754	269827	74	2

FGRAMPH1_01G16299	FGRAMPH1_01T16299	1.57423	5.68813	Promoter	3	270308	272458	2151	1
FGRAMPH1_01G16355	FGRAMPH1_01T16355	1.72186	8.19781	Promoter	3	333619	334239	621	1
FGRAMPH1_01G16399	FGRAMPH1_01T16399	2.09094	15.99497	Promoter	3	377030	379126	2097	2
FGRAMPH1_01G16403	FGRAMPH1_01T16403	2.17862	21.01041	Promoter	3	386585	387850	1266	2
FGRAMPH1_01G16405	FGRAMPH1_01T16405	1.80041	10.8107	Promoter	3	388713	390842	2130	2
FGRAMPH1_01G16553	FGRAMPH1_01T16553	2.24912	20.12992	Promoter	3	544503	545373	871	1
FGRAMPH1_01G16555	FGRAMPH1_01T16555	1.35075	3.63018	UTR	3	545424	546057	634	2
FGRAMPH1_01G16561	FGRAMPH1_01T16561	1.72338	10.43115	UTR	3	550199	551718	1520	1
FGRAMPH1_01G16565	FGRAMPH1_01T16565	1.73419	10.38566	Promoter	3	553135	554562	1428	1
FGRAMPH1_01G16563	FGRAMPH1_01T16563	1.93261	15.87823	UTR	3	552694	555914	3221	2
FGRAMPH1_01G16567	FGRAMPH1_01T16567	1.86035	15.26996	Promoter	3	557070	560862	3793	1
FGRAMPH1_01G16613	FGRAMPH1_01T16613	1.94317	14.96026	Promoter	3	614177	615942	1766	1
FGRAMPH1_01G16625	FGRAMPH1_01T16625	1.58236	8.31082	Promoter	3	629094	630275	1182	2

FGRAMPH1_01G16629	FGRAMPH1_01T16629	2.31046	24.53113	Promoter	3	632512	633646	1135	2
FGRAMPH1_01G16637	FGRAMPH1_01T16637	1.27261	3.10209	Promoter	3	643056	643406	351	1
FGRAMPH1_01G16647	FGRAMPH1_01T16647	1.66317	7.44658	Promoter	3	650805	652521	1717	1
FGRAMPH1_01G16669	FGRAMPH1_01T16669	1.65211	8.49829	Promoter	3	674469	674948	480	1
FGRAMPH1_01G16705	FGRAMPH1_01T16705	2.08323	17.46304	Promoter	3	712289	717997	5709	2
FGRAMPH1_01G16717	FGRAMPH1_01T16717	1.84024	13.48028	Promoter	3	731121	733608	2488	2
ENSRNA049555985	ENSRNA049555985-T1	2.13313	16.99246	Promoter	3	767460	767531	72	1
ENSRNA049557928	ENSRNA049557928-T1	2.43829	26.15361	Promoter	3	770060	770154	95	2
FGRAMPH1_01G16829	FGRAMPH1_01T16829	1.99017	16.72373	Promoter	3	842560	844106	1547	1
ENSRNA049557900	ENSRNA049557900-T1	1.67245	9.40702	Promoter	3	862283	862382	100	2
FGRAMPH1_01G16843	FGRAMPH1_01T16843	1.34372	3.63018	Promoter	3	861956	863253	1298	2
ENSRNA049556016	ENSRNA049556016-T1	2.16476	18.0144	Promoter	3	863817	863911	95	1
FGRAMPH1_01G16865	FGRAMPH1_01T16865	1.93264	13.25799	Promoter	3	882350	882541	192	1

FGRAMPH1_01G16883	FGRAMPH1_01T16883	1.66913	7.23732	Promoter	3	902289	906140	3852	2
FGRAMPH1_01G16905	FGRAMPH1_01T16905	1.80111	9.99645	Intergenic	3	931670	931891	222	1
FGRAMPH1_01G16911	FGRAMPH1_01T16911	1.86914	15.60694	Intron	3	938165	940868	2704	1
FGRAMPH1_01G16913	FGRAMPH1_01T16913	1.56782	5.70743	Intergenic	3	941277	942155	879	2
FGRAMPH1_01G16925	FGRAMPH1_01T16925	2.20694	19.06036	Promoter	3	952317	954842	2526	1
FGRAMPH1_01G16943	FGRAMPH1_01T16943	1.69678	7.79228	UTR	3	970629	972428	1800	2
FGRAMPH1_01G16945	FGRAMPH1_01T16945	1.50164	6.14871	UTR	3	972691	974542	1852	1
FGRAMPH1_01G16949	FGRAMPH1_01T16949	1.90209	18.13924	Promoter	3	977193	978053	861	2
FGRAMPH1_01G16957	FGRAMPH1_01T16957	2.23858	19.7708	Promoter	3	988124	990907	2784	2
FGRAMPH1_01G16959	FGRAMPH1_01T16959	2.56377	29.84416	Intergenic	3	992831	993273	443	2
FGRAMPH1_01G16961	FGRAMPH1_01T16961	1.57038	6.74195	Exon	3	994457	995743	1287	1
FGRAMPH1_01G16963	FGRAMPH1_01T16963	1.88728	11.96647	Promoter	3	997090	998028	939	1
FGRAMPH1_01G16981	FGRAMPH1_01T16981	1.43584	3.95787	Exon	3	1017922	1018916	995	1

FGRAMPH1_01G16985	FGRAMPH1_01T16985	1.29165	3.26179	Promoter	3	1020395	1022619	2225	1
FGRAMPH1_01G16983	FGRAMPH1_01T16983	1.46683	5.48325	Promoter	3	1020218	1020418	201	2
FGRAMPH1_01G16987	FGRAMPH1_01T16987	1.78513	9.21114	Promoter	3	1024376	1025172	797	1
FGRAMPH1_01G16993	FGRAMPH1_01T16993	1.65791	8.52357	Exon	3	1028817	1029167	351	1
FGRAMPH1_01G17001	FGRAMPH1_01T17001	1.62874	7.00942	Intergenic	3	1037824	1038171	348	2
FGRAMPH1_01G17003	FGRAMPH1_01T17003	1.80667	13.3134	Promoter	3	1038462	1038719	258	1
FGRAMPH1_01G17007	FGRAMPH1_01T17007	1.66984	10.09968	Promoter	3	1042995	1046885	3891	1
ENSRNA049557852	ENSRNA049557852-T1	2.21852	21.99909	Promoter	3	1064261	1064350	90	2
FGRAMPH1_01G17027	FGRAMPH1_01T17027	1.67968	7.4724	Promoter	3	1071473	1073478	2006	1
FGRAMPH1_01G17031	FGRAMPH1_01T17031	1.29698	3.01552	Promoter	3	1075560	1078457	2898	2
FGRAMPH1_01G17041	FGRAMPH1_01T17041	1.59144	9.62952	Exon	3	1087158	1087938	781	1
FGRAMPH1_01G17043	FGRAMPH1_01T17043	1.32476	3.33384	Intergenic	3	1089784	1089942	159	2
FGRAMPH1_01G17049	FGRAMPH1_01T17049	1.31736	3.86418	Promoter	3	1104171	1107168	2998	2

FGRAMPH1_01G17051	FGRAMPH1_01T17051	1.7863	10.73588	Promoter	3	1109280	1112585	3306	1
FGRAMPH1_01G17063	FGRAMPH1_01T17063	1.59926	6.78263	Promoter	3	1121288	1123780	2493	1
FGRAMPH1_01G17107	FGRAMPH1_01T17107	1.89058	11.39068	Promoter	3	1165058	1171069	6012	2
FGRAMPH1_01G17109	FGRAMPH1_01T17109	1.93176	12.57624	Intron	3	1173457	1174233	777	1
FGRAMPH1_01G17111	FGRAMPH1_01T17111	1.52186	5.11203	Promoter	3	1176376	1177741	1366	1
FGRAMPH1_01G17141	FGRAMPH1_01T17141	1.29926	3.13871	Promoter	3	1211591	1212292	702	2
FGRAMPH1_01G17143	FGRAMPH1_01T17143	1.51294	5.01704	Promoter	3	1214109	1214626	518	1
FGRAMPH1_01G17145	FGRAMPH1_01T17145	1.60658	6.47771	Exon	3	1215267	1215763	497	1
FGRAMPH1_01G17147	FGRAMPH1_01T17147	1.6709	10.46476	Promoter	3	1217124	1218491	1368	2
FGRAMPH1_01G17149	FGRAMPH1_01T17149	2.13086	19.47969	Promoter	3	1221158	1221567	410	1
FGRAMPH1_01G17163	FGRAMPH1_01T17163	1.76989	10.8197	Promoter	3	1238620	1239579	960	2
FGRAMPH1_01G17183	FGRAMPH1_01T17183	1.51018	7.21184	Promoter	3	1263277	1264013	737	2
FGRAMPH1_01G17197	FGRAMPH1_01T17197	1.64859	8.16102	Promoter	3	1279749	1281645	1897	2

FGRAMPH1_01G17201	FGRAMPH1_01T17201	1.75476	9.58399	Promoter	3	1283594	1287054	3461	2
FGRAMPH1_01G17207	FGRAMPH1_01T17207	2.34173	26.91145	Exon	3	1290983	1291336	354	2
FGRAMPH1_01G17231	FGRAMPH1_01T17231	1.61492	6.5072	Promoter	3	1325863	1327856	1994	2
FGRAMPH1_01G17233	FGRAMPH1_01T17233	2.52933	28.54725	Exon	3	1329348	1330632	1285	2
FGRAMPH1_01G17235	FGRAMPH1_01T17235	1.97495	13.45803	Promoter	3	1332775	1333872	1098	1
FGRAMPH1_01G17239	FGRAMPH1_01T17239	1.92222	12.25882	Promoter	3	1339820	1341724	1905	1
FGRAMPH1_01G17245	FGRAMPH1_01T17245	1.61518	8.26665	Promoter	3	1346102	1349924	3823	1
FGRAMPH1_01G17253	FGRAMPH1_01T17253	1.5262	5.49814	Exon	3	1356281	1356564	284	1
FGRAMPH1_01G17261	FGRAMPH1_01T17261	1.46853	5.75699	Promoter	3	1365709	1367129	1421	2
FGRAMPH1_01G17271	FGRAMPH1_01T17271	1.33109	4.09855	Promoter	3	1376506	1378985	2480	2
FGRAMPH1_01G17273	FGRAMPH1_01T17273	2.05306	16.07769	Promoter	3	1381914	1383437	1524	1
FGRAMPH1_01G17285	FGRAMPH1_01T17285	2.56548	29.0723	Promoter	3	1399544	1399948	405	2
FGRAMPH1_01G17287	FGRAMPH1_01T17287	2.21579	19.0297	Promoter	3	1400748	1402941	2194	1

FGRAMPH1_01G17305	FGRAMPH1_01T17305	1.62632	9.96222	Exon	3	1430348	1430939	592	2
FGRAMPH1_01G17307	FGRAMPH1_01T17307	2.24912	20.12992	Exon	3	1434394	1435265	872	1
FGRAMPH1_01G17309	FGRAMPH1_01T17309	2.16214	18.65032	Intergenic	3	1438537	1439007	471	2
FGRAMPH1_01G17311	FGRAMPH1_01T17311	1.93226	16.5354	Exon	3	1440914	1441450	537	2
FGRAMPH1_01G17313	FGRAMPH1_01T17313	2.42839	25.02763	Promoter	3	1443828	1444028	201	2
FGRAMPH1_01G17315	FGRAMPH1_01T17315	2.15422	17.67106	Promoter	3	1445809	1446216	408	2
FGRAMPH1_01G17325	FGRAMPH1_01T17325	1.91167	11.96643	Promoter	3	1468873	1470682	1810	1
FGRAMPH1_01G17363	FGRAMPH1_01T17363	1.3878	3.41725	Promoter	3	1508049	1512227	4179	1
FGRAMPH1_01G17367	FGRAMPH1_01T17367	1.53205	8.36186	Promoter	3	1514317	1516248	1932	2
FGRAMPH1_01G17369	FGRAMPH1_01T17369	1.39399	5.36805	Intergenic	3	1519578	1519847	270	2
FGRAMPH1_01G17371	FGRAMPH1_01T17371	1.55314	5.27724	Promoter	3	1521890	1522680	791	1
FGRAMPH1_01G17397	FGRAMPH1_01T17397	1.58157	8.49947	Promoter	3	1562321	1562560	240	1
ENSRNA049557834	ENSRNA049557834-T1	1.88644	15.99717	Promoter	3	1579838	1579918	81	2

FGRAMPH1_01G17421	FGRAMPH1_01T17421	1.58477	5.89894	Promoter	3	1586356	1588895	2540	2
FGRAMPH1_01G17423	FGRAMPH1_01T17423	1.69807	8.92016	Promoter	3	1592543	1596563	4021	1
FGRAMPH1_01G17469	FGRAMPH1_01T17469	1.54584	6.62653	Promoter	3	1656298	1658677	2380	2
FGRAMPH1_01G17475	FGRAMPH1_01T17475	1.58645	7.19548	UTR	3	1663529	1666429	2901	2
FGRAMPH1_01G17479	FGRAMPH1_01T17479	1.57423	5.68813	Promoter	3	1669427	1672484	3058	1
FGRAMPH1_01G17515	FGRAMPH1_01T17515	1.83786	10.2759	Promoter	3	1720806	1723233	2428	1
ENSRNA049557812	ENSRNA049557812-T1	2.55494	28.6574	Promoter	3	1723749	1723820	72	2
FGRAMPH1_01G17519	FGRAMPH1_01T17519	1.7377	9.16614	Promoter	3	1729583	1732305	2723	1
FGRAMPH1_01G17521	FGRAMPH1_01T17521	1.72186	8.19781	Promoter	3	1733523	1737145	3623	2
FGRAMPH1_01G17541	FGRAMPH1_01T17541	2.03822	14.70381	Promoter	3	1757458	1758140	683	1
ENSRNA049556045	ENSRNA049556045-T1	2.58657	29.90888	Promoter	3	1767733	1767827	95	1
FGRAMPH1_01G17551	FGRAMPH1_01T17551	2.61821	30.75447	Exon	3	1778526	1778758	233	2
FGRAMPH1_01G17581	FGRAMPH1_01T17581	1.8584	11.3743	Promoter	3	1810914	1812074	1161	2

FGRAMPH1_01G17583	FGRAMPH1_01T17583	1.83856	12.63985	Promoter	3	1823659	1824429	771	2
FGRAMPH1_01G17585	FGRAMPH1_01T17585	1.91403	13.01548	Promoter	3	1826014	1826484	471	2
FGRAMPH1_01G17587	FGRAMPH1_01T17587	2.05184	15.31979	Promoter	3	1827754	1828436	683	1
FGRAMPH1_01G17591	FGRAMPH1_01T17591	1.39378	4.69119	Promoter	3	1831879	1834296	2418	1
FGRAMPH1_01G17593	FGRAMPH1_01T17593	1.50865	5.26534	Promoter	3	1834334	1835560	1227	2
FGRAMPH1_01G17615	FGRAMPH1_01T17615	1.71128	10.81781	Promoter	3	1862269	1868641	6373	2
FGRAMPH1_01G17619	FGRAMPH1_01T17619	1.54776	7.59519	Promoter	3	1871590	1878747	7158	1
FGRAMPH1_01G17623	FGRAMPH1_01T17623	1.56204	6.19585	Promoter	3	1879031	1880887	1857	1
FGRAMPH1_01G17661	FGRAMPH1_01T17661	1.5381	6.57609	Promoter	3	1942557	1943279	723	2
ENSRNA049557796	ENSRNA049557796-T1	2.58657	29.90888	Promoter	3	1974910	1974981	72	2
FGRAMPH1_01G17711	FGRAMPH1_01T17711	2.03576	15.66131	Promoter	3	1997677	1998190	514	2
FGRAMPH1_01G17737	FGRAMPH1_01T17737	1.35752	3.72701	Promoter	3	2020659	2022564	1906	1
FGRAMPH1_01G17745	FGRAMPH1_01T17745	1.88004	11.39068	Promoter	3	2029285	2030364	1080	2

FGRAMPH1_01G17769	FGRAMPH1_01T17769	2.00617	14.62604	Promoter	3	2082978	2086565	3588	2
FGRAMPH1_01G17771	FGRAMPH1_01T17771	1.37494	4.78282	Promoter	3	2089228	2091592	2365	1
FGRAMPH1_01G17775	FGRAMPH1_01T17775	1.62216	6.88614	Promoter	3	2091984	2093841	1858	1
FGRAMPH1_01G17779	FGRAMPH1_01T17779	1.51189	6.62881	Promoter	3	2094848	2096449	1602	2
FGRAMPH1_01G17781	FGRAMPH1_01T17781	1.46034	5.22756	Promoter	3	2097109	2099878	2770	2
FGRAMPH1_01G17809	FGRAMPH1_01T17809	1.43611	4.45341	Promoter	3	2129570	2133297	3728	2
FGRAMPH1_01G17815	FGRAMPH1_01T17815	1.56683	5.84759	Promoter	3	2137824	2142952	5129	2
FGRAMPH1_01G17819	FGRAMPH1_01T17819	1.91243	14.20546	Promoter	3	2146423	2146755	333	1
FGRAMPH1_01G17821	FGRAMPH1_01T17821	1.36308	3.98523	Promoter	3	2148379	2152559	4181	1
FGRAMPH1_01G17837	FGRAMPH1_01T17837	2.24912	20.12992	Promoter	3	2167737	2169404	1668	2
FGRAMPH1_01G17853	FGRAMPH1_01T17853	2.08395	15.87437	Promoter	3	2188000	2190968	2969	1
FGRAMPH1_01G17891	FGRAMPH1_01T17891	1.87272	11.96157	Promoter	3	2246894	2251147	4254	1
FGRAMPH1_01G17907	FGRAMPH1_01T17907	1.5232	5.52842	Promoter	3	2266289	2266657	369	1

FGRAMPH1_01G17905	FGRAMPH1_01T17905	2.2986	22.52067	Promoter	3	2266149	2269353	3205	2
FGRAMPH1_01G17919	FGRAMPH1_01T17919	2.23858	19.7708	Promoter	3	2285620	2287531	1912	2
FGRAMPH1_01G17957	FGRAMPH1_01T17957	1.94288	14.57558	Promoter	3	2341915	2343240	1326	2
FGRAMPH1_01G17959	FGRAMPH1_01T17959	1.90901	12.57083	Promoter	3	2344195	2344757	563	2
FGRAMPH1_01G17961	FGRAMPH1_01T17961	1.81446	9.92212	Promoter	3	2344379	2344757	379	2
FGRAMPH1_01G17989	FGRAMPH1_01T17989	1.64804	6.77735	Promoter	3	2383799	2384527	729	2
FGRAMPH1_01G17991	FGRAMPH1_01T17991	1.60949	7.86579	Promoter	3	2386489	2387951	1463	1
FGRAMPH1_01G17997	FGRAMPH1_01T17997	1.8786	11.36803	Promoter	3	2390484	2393668	3185	2
FGRAMPH1_01G17999	FGRAMPH1_01T17999	1.57569	8.00953	Promoter	3	2393777	2394757	981	2
FGRAMPH1_01G18001	FGRAMPH1_01T18001	1.44388	5.17555	Promoter	3	2397130	2398125	996	1
FGRAMPH1_01G18003	FGRAMPH1_01T18003	1.60364	8.79109	Promoter	3	2399083	2401221	2139	2
FGRAMPH1_01G18009	FGRAMPH1_01T18009	1.54007	7.0597	Promoter	3	2408389	2408978	590	2
FGRAMPH1_01G18021	FGRAMPH1_01T18021	2.51276	27.42648	Promoter	3	2422703	2425684	2982	2

FGRAMPH1_01G18029	FGRAMPH1_01T18029	1.89058	11.39068	Promoter	3	2432670	2432986	317	2
FGRAMPH1_01G18031	FGRAMPH1_01T18031	1.80622	9.73718	Promoter	3	2433838	2437420	3583	1
FGRAMPH1_01G18085	FGRAMPH1_01T18085	1.86651	11.01471	Promoter	3	2494165	2495367	1203	2
FGRAMPH1_01G18125	FGRAMPH1_01T18125	1.56615	6.50391	Promoter	3	2566499	2568835	2337	1
FGRAMPH1_01G18127	FGRAMPH1_01T18127	1.61179	6.54793	Promoter	3	2570046	2572572	2527	1
FGRAMPH1_01G18129	FGRAMPH1_01T18129	1.42473	6.06108	UTR	3	2572735	2573576	842	1
FGRAMPH1_01G18131	FGRAMPH1_01T18131	1.30865	3.57838	Promoter	3	2573697	2578531	4835	2
FGRAMPH1_01G18135	FGRAMPH1_01T18135	2.02767	14.38806	Promoter	3	2579891	2580427	537	2
FGRAMPH1_01G18139	FGRAMPH1_01T18139	1.75267	9.3996	Promoter	3	2591281	2592796	1516	1
FGRAMPH1_01G18145	FGRAMPH1_01T18145	2.00658	14.07517	Promoter	3	2599645	2600430	786	1
FGRAMPH1_01G18151	FGRAMPH1_01T18151	1.79922	11.67189	Promoter	3	2608079	2612817	4739	1
FGRAMPH1_01G18173	FGRAMPH1_01T18173	1.75227	9.08761	Promoter	3	2641725	2643885	2161	1
FGRAMPH1_01G18175	FGRAMPH1_01T18175	1.97917	17.3865	Promoter	3	2645069	2645592	524	1

FGRAMPH1_01G18179	FGRAMPH1_01T18179	1.92693	12.74673	UTR	3	2647508	2653341	5834	1
FGRAMPH1_01G18187	FGRAMPH1_01T18187	1.98549	13.45803	Promoter	3	2658663	2660414	1752	1
FGRAMPH1_01G18221	FGRAMPH1_01T18221	1.61257	7.05748	Promoter	3	2710675	2713302	2628	1
FGRAMPH1_01G18223	FGRAMPH1_01T18223	1.77225	10.65396	Promoter	3	2713295	2717749	4455	2
FGRAMPH1_01G18229	FGRAMPH1_01T18229	1.50235	4.8724	Exon	3	2718803	2720257	1455	2
FGRAMPH1_01G18231	FGRAMPH1_01T18231	1.6617	7.38851	Exon	3	2720878	2721798	921	1
FGRAMPH1_01G18233	FGRAMPH1_01T18233	1.86776	11.45628	Promoter	3	2724345	2729699	5355	2
FGRAMPH1_01G18249	FGRAMPH1_01T18249	2.7353	36.28284	Promoter	3	2747696	2749010	1315	2
FGRAMPH1_01G18271	FGRAMPH1_01T18271	2.40306	28.81761	Promoter	3	2777371	2779345	1975	2
FGRAMPH1_01G18277	FGRAMPH1_01T18277	1.69022	7.4724	Promoter	3	2784780	2786945	2166	1
FGRAMPH1_01G18285	FGRAMPH1_01T18285	1.73009	8.75276	Promoter	3	2796491	2798730	2240	2
FGRAMPH1_01G18287	FGRAMPH1_01T18287	1.34692	3.53681	Promoter	3	2803205	2804736	1532	2
FGRAMPH1_01G18291	FGRAMPH1_01T18291	1.69176	9.58175	Promoter	3	2809313	2809912	600	2

FGRAMPH1 _01G18315	FGRAMPH1 _01T18315	1.88316	12.71402	UTR	3	2837388	2838239	852	1
FGRAMPH1 _01G18317	FGRAMPH1 _01T18317	1.77556	10.4969	Promoter	3	2838970	2840998	2029	2
FGRAMPH1 _01G18327	FGRAMPH1 _01T18327	1.92222	12.25882	Promoter	3	2850710	2852516	1807	1
FGRAMPH1 _01G18335	FGRAMPH1 _01T18335	1.50914	6.14871	Promoter	3	2859036	2860385	1350	1
FGRAMPH1 _01G18353	FGRAMPH1 _01T18353	2.14367	17.33041	Promoter	3	2881539	2883785	2247	2
FGRAMPH1 _01G18355	FGRAMPH1 _01T18355	1.78513	9.21114	Promoter	3	2885041	2886653	1613	2
FGRAMPH1 _01G18371	FGRAMPH1 _01T18371	2.04374	15.84348	UTR	3	2904341	2908911	4571	2
FGRAMPH1 _01G18375	FGRAMPH1 _01T18375	1.94202	12.87145	Promoter	3	2912305	2913300	996	2
FGRAMPH1 _01G18401	FGRAMPH1 _01T18401	1.27316	3.17121	Promoter	3	2954872	2957496	2625	1
FGRAMPH1 _01G18419	FGRAMPH1 _01T18419	1.81116	10.03015	Promoter	3	2978680	2980620	1941	2
FGRAMPH1 _01G18421	FGRAMPH1 _01T18421	1.90113	11.67704	Promoter	3	2982966	2986527	3562	1
FGRAMPH1 _01G18423	FGRAMPH1 _01T18423	1.54472	6.00347	Promoter	3	2986597	2986952	356	2
FGRAMPH1 _01G18429	FGRAMPH1 _01T18429	1.63289	7.62231	Promoter	3	2992857	2993906	1050	2

FGRAMPH1_01G18431	FGRAMPH1_01T18431	1.99604	13.76516	UTR	3	2999167	3002587	3421	1
ENSRNA049556056	ENSRNA049556056-T1	1.67809	9.2886	Promoter	3	3002934	3003005	72	1
FGRAMPH1_01G18437	FGRAMPH1_01T18437	2.156	18.56768	Promoter	3	3015360	3016298	939	2
FGRAMPH1_01G18439	FGRAMPH1_01T18439	1.47594	5.40477	Promoter	3	3017024	3017235	212	2
FGRAMPH1_01G18441	FGRAMPH1_01T18441	1.53594	7.73561	Promoter	3	3018438	3018875	438	1
FGRAMPH1_01G18447	FGRAMPH1_01T18447	2.60766	30.33056	Promoter	3	3025273	3028268	2996	2
FGRAMPH1_01G18453	FGRAMPH1_01T18453	1.49998	5.17813	Intergenic	3	3033382	3033859	478	1
FGRAMPH1_01G18485	FGRAMPH1_01T18485	2.05931	15.34379	Promoter	3	3064072	3066415	2344	2
FGRAMPH1_01G18493	FGRAMPH1_01T18493	1.9644	13.15382	Intergenic	3	3070829	3071672	844	2
FGRAMPH1_01G18519	FGRAMPH1_01T18519	1.59926	6.78263	Promoter	3	3101536	3102892	1357	1
FGRAMPH1_01G18523	FGRAMPH1_01T18523	1.6448	8.34086	Promoter	3	3106222	3111932	5711	1
FGRAMPH1_01G18577	FGRAMPH1_01T18577	1.51095	4.68809	Promoter	3	3166278	3167097	820	1
FGRAMPH1_01G18585	FGRAMPH1_01T18585	1.62695	6.55252	Promoter	3	3170781	3172745	1965	1

ENSRNA049556079	ENSRNA049556079-T1	2.39676	24.24705	Promoter	3	3184724	3184826	103	1
ENSRNA049556109	ENSRNA049556109-T1	1.5176	7.45118	Promoter	3	3195646	3195741	96	1
FGRAMPH1_01G18625	FGRAMPH1_01T18625	1.76404	8.95294	Promoter	3	3211576	3211904	329	2
FGRAMPH1_01G18627	FGRAMPH1_01T18627	2.03822	14.70381	Promoter	3	3212473	3213582	1110	1
FGRAMPH1_01G18635	FGRAMPH1_01T18635	2.27776	24.97313	Promoter	3	3222329	3223746	1418	2
FGRAMPH1_01G18659	FGRAMPH1_01T18659	1.86371	13.46342	Promoter	3	3258084	3260359	2276	2
ENSRNA049556142	ENSRNA049556142-T1	2.52316	31.94636	Promoter	3	3270856	3270938	83	1
FGRAMPH1_01G18685	FGRAMPH1_01T18685	1.3421	3.39624	Exon	3	3289283	3289841	559	2
ENSRNA049556183	ENSRNA049556183-T1	2.78693	36.46401	Promoter	3	3297564	3297653	90	1
FGRAMPH1_01G18695	FGRAMPH1_01T18695	2.23858	19.7708	Promoter	3	3299810	3301083	1274	1
ENSRNA049557767	ENSRNA049557767-T1	2.17531	18.0144	Promoter	3	3301226	3301299	74	2
ENSRNA049556212	ENSRNA049556212-T1	2.55494	28.6574	Promoter	3	3326660	3326731	72	1
FGRAMPH1_01G18753	FGRAMPH1_01T18753	1.90651	16.1734	Promoter	3	3374941	3375360	420	2

FGRAMPH1_01G18755	FGRAMPH1_01T18755	2.2474	20.09826	Promoter	3	3377107	3379338	2232	1
FGRAMPH1_01G18757	FGRAMPH1_01T18757	1.29493	3.05022	Promoter	3	3380437	3380952	516	1
ENSRNA049556229	ENSRNA049556229-T1	2.46003	25.8178	Promoter	3	3381178	3381260	83	1
FGRAMPH1_01G18761	FGRAMPH1_01T18761	1.8807	11.78471	Promoter	3	3389472	3392118	2647	2
FGRAMPH1_01G18789	FGRAMPH1_01T18789	2.4073	24.63614	Promoter	3	3427846	3430570	2725	2
ENSRNA049557737	ENSRNA049557737-T1	2.53385	28.2448	Promoter	3	3436917	3437006	90	2
FGRAMPH1_01G18795	FGRAMPH1_01T18795	1.65859	7.00562	Promoter	3	3437999	3439254	1256	1
FGRAMPH1_01G18805	FGRAMPH1_01T18805	1.81677	10.00496	Exon	3	3447114	3447779	666	1
FGRAMPH1_01G18807	FGRAMPH1_01T18807	2.17531	18.0144	Promoter	3	3448536	3449811	1276	1
FGRAMPH1_01G18849	FGRAMPH1_01T18849	2.11204	16.65722	Promoter	3	3488708	3491929	3222	2
FGRAMPH1_01G18885	FGRAMPH1_01T18885	1.92222	12.25882	Promoter	3	3524409	3524879	471	1
FGRAMPH1_01G18895	FGRAMPH1_01T18895	2.34403	22.71505	Promoter	3	3528846	3530183	1338	2
FGRAMPH1_01G18897	FGRAMPH1_01T18897	1.99604	13.76516	Promoter	3	3530792	3532489	1698	2

FGRAMPH1_01G18899	FGRAMPH1_01T18899	1.72314	8.55089	Promoter	3	3536408	3538697	2290	2
FGRAMPH1_01G18907	FGRAMPH1_01T18907	1.45112	3.94547	Promoter	3	3549004	3550475	1472	2
FGRAMPH1_01G18909	FGRAMPH1_01T18909	2.15422	17.67106	Promoter	3	3551696	3553450	1755	2
FGRAMPH1_01G18919	FGRAMPH1_01T18919	1.66917	11.7496	Promoter	3	3562710	3564728	2019	2
FGRAMPH1_01G18937	FGRAMPH1_01T18937	1.50706	5.31223	Exon	3	3591197	3592422	1226	2
FGRAMPH1_01G18957	FGRAMPH1_01T18957	1.51095	4.68809	Promoter	3	3614597	3616291	1695	2
FGRAMPH1_01G19031	FGRAMPH1_01T19031	1.54024	7.20573	Intergenic	3	3703257	3703876	620	2
FGRAMPH1_01G19045	FGRAMPH1_01T19045	1.58806	7.0385	UTR	3	3730367	3732986	2620	1
FGRAMPH1_01G19063	FGRAMPH1_01T19063	1.53893	6.76962	Promoter	3	3754281	3756701	2421	2
FGRAMPH1_01G19067	FGRAMPH1_01T19067	1.63671	6.83235	Promoter	3	3757940	3758362	423	1
FGRAMPH1_01G19091	FGRAMPH1_01T19091	1.77714	10.57911	Promoter	3	3775942	3779175	3234	2
FGRAMPH1_01G19101	FGRAMPH1_01T19101	1.82258	11.58009	Promoter	3	3798687	3800522	1836	2
FGRAMPH1_01G19121	FGRAMPH1_01T19121	1.76392	9.99179	Exon	3	3818613	3820754	2142	1

FGRAMPH1_01G19161	FGRAMPH1_01T19161	1.98549	13.45803	Promoter	3	3856862	3857756	895	1
FGRAMPH1_01G19163	FGRAMPH1_01T19163	1.63569	7.98647	Promoter	3	3857936	3860216	2281	1
FGRAMPH1_01G19191	FGRAMPH1_01T19191	1.63709	6.89045	Promoter	3	3885114	3887206	2093	2
FGRAMPH1_01G19199	FGRAMPH1_01T19199	1.56198	6.49577	Intergenic	3	3893504	3893920	417	2
ENSRNA049556244	ENSRNA049556244-T1	1.91318	15.47199	Promoter	3	3904553	3904635	83	1
FGRAMPH1_01G19229	FGRAMPH1_01T19229	2.40418	26.53269	Promoter	3	3930506	3932881	2376	1
FGRAMPH1_01G19239	FGRAMPH1_01T19239	1.70947	8.6338	Promoter	3	3949967	3950957	991	1
FGRAMPH1_01G19251	FGRAMPH1_01T19251	2.04876	15.02239	Intergenic	3	3977125	3977484	360	2
FGRAMPH1_01G19257	FGRAMPH1_01T19257	1.71065	8.8364	Promoter	3	3997223	3999597	2375	1
FGRAMPH1_01G19259	FGRAMPH1_01T19259	1.63993	6.67032	UTR	3	3999687	4000858	1172	2
FGRAMPH1_01G19269	FGRAMPH1_01T19269	1.66913	7.23732	Promoter	3	4007319	4009542	2224	2
FGRAMPH1_01G19283	FGRAMPH1_01T19283	1.91762	12.79497	Promoter	3	4031260	4032079	820	1
FGRAMPH1_01G19285	FGRAMPH1_01T19285	2.03607	15.52783	Promoter	3	4031451	4033149	1699	2

FGRAMPH1_01G19307	FGRAMPH1_01T19307	1.47509	5.44533	Promoter	3	4064006	4065730	1725	1
FGRAMPH1_01G19309	FGRAMPH1_01T19309	2.20498	23.7647	Promoter	3	4065962	4066372	411	1
FGRAMPH1_01G19343	FGRAMPH1_01T19343	1.57324	7.33789	Promoter	3	4101669	4104388	2720	2
ENSRNA049556273	ENSRNA049556273-T1	1.9073	16.83255	Promoter	3	4126594	4126698	105	1
FGRAMPH1_01G19363	FGRAMPH1_01T19363	1.80105	11.92093	Promoter	3	4134281	4136560	2280	1
FGRAMPH1_01G19365	FGRAMPH1_01T19365	1.6375	6.77735	Promoter	3	4139580	4142354	2775	2
FGRAMPH1_01G19367	FGRAMPH1_01T19367	1.88004	11.39068	Exon	3	4142711	4143559	849	2
FGRAMPH1_01G19377	FGRAMPH1_01T19377	1.65679	8.04526	Promoter	3	4158216	4158533	318	2
FGRAMPH1_01G19379	FGRAMPH1_01T19379	1.75707	9.82646	Promoter	3	4161639	4164048	2410	2
FGRAMPH1_01G19389	FGRAMPH1_01T19389	1.85895	10.82712	Promoter	3	4174171	4175193	1023	2
FGRAMPH1_01G19391	FGRAMPH1_01T19391	1.37004	4.22877	Exon	3	4176221	4178197	1977	2
FGRAMPH1_01G19393	FGRAMPH1_01T19393	2.10969	17.29145	Promoter	3	4178777	4179923	1147	1
FGRAMPH1_01G19395	FGRAMPH1_01T19395	2.42839	25.02763	Promoter	3	4180802	4181923	1122	1

FGRAMPH1_01G19399	FGRAMPH1_01T19399	1.59532	5.89894	Promoter	3	4182521	4183441	921	1
FGRAMPH1_01G19417	FGRAMPH1_01T19417	1.40274	4.61677	Promoter	3	4214937	4218194	3258	1
ENSRNA049557713	ENSRNA049557713-T1	2.35458	23.09437	Promoter	3	4235740	4235829	90	2
FGRAMPH1_01G19437	FGRAMPH1_01T19437	2.07594	18.33354	Promoter	3	4242713	4243627	915	2
FGRAMPH1_01G19439	FGRAMPH1_01T19439	1.70548	10.47209	Promoter	3	4245601	4246472	872	1
FGRAMPH1_01G19455	FGRAMPH1_01T19455	1.51952	5.75071	Promoter	3	4266939	4267340	402	1
FGRAMPH1_01G19457	FGRAMPH1_01T19457	1.97495	13.45803	Promoter	3	4267202	4267468	267	2
ENSRNA049511517	ENSRNA049511517-T1	2.52455	31.19634	Promoter	3	4293127	4293260	134	1
FGRAMPH1_01G19503	FGRAMPH1_01T19503	2.38153	25.36183	Promoter	3	4327911	4330095	2185	1
FGRAMPH1_01G19537	FGRAMPH1_01T19537	2.19888	21.88102	Promoter	3	4363399	4364019	621	1
FGRAMPH1_01G19539	FGRAMPH1_01T19539	1.36165	4.28606	Promoter	3	4364770	4367871	3102	1
FGRAMPH1_01G19541	FGRAMPH1_01T19541	1.763	10.54256	UTR	3	4368177	4368840	664	2
FGRAMPH1_01G19545	FGRAMPH1_01T19545	1.93929	15.01099	Promoter	3	4371960	4374386	2427	2

FGRAMPH1_01G19551	FGRAMPH1_01T19551	2.13313	16.99246	Promoter	3	4382706	4383221	516	2
FGRAMPH1_01G19553	FGRAMPH1_01T19553	2.14367	17.33041	Promoter	3	4385690	4386403	714	1
FGRAMPH1_01G19555	FGRAMPH1_01T19555	1.45605	6.15108	Exon	3	4386727	4387071	345	2
FGRAMPH1_01G19577	FGRAMPH1_01T19577	2.09068	19.03558	Promoter	3	4403926	4406558	2633	2
FGRAMPH1_01G19579	FGRAMPH1_01T19579	1.32079	3.69417	Promoter	3	4407509	4408558	1050	2
FGRAMPH1_01G19581	FGRAMPH1_01T19581	1.76694	9.67374	Intergenic	3	4415567	4415872	306	2
FGRAMPH1_01G19591	FGRAMPH1_01T19591	1.58477	5.89894	UTR	3	4430595	4432348	1754	1
FGRAMPH1_01G19597	FGRAMPH1_01T19597	1.35204	4.39531	Promoter	3	4438045	4439532	1488	1
FGRAMPH1_01G19603	FGRAMPH1_01T19603	1.77298	11.55733	UTR	3	4446240	4447209	970	1
FGRAMPH1_01G19605	FGRAMPH1_01T19605	1.85442	12.61883	Exon	3	4448736	4452302	3567	2
FGRAMPH1_01G19609	FGRAMPH1_01T19609	2.44948	25.8178	Intergenic	3	4455704	4455947	244	1
FGRAMPH1_01G19613	FGRAMPH1_01T19613	1.78513	9.21114	Promoter	3	4465753	4466937	1185	2
FGRAMPH1_01G19615	FGRAMPH1_01T19615	1.95386	12.85253	Promoter	3	4467546	4469366	1821	1

FGRAMPH1_01G19617	FGRAMPH1_01T19617	1.86949	11.10737	Exon	3	4470479	4470932	454	1
FGRAMPH1_01G19619	FGRAMPH1_01T19619	1.6763	8.03738	Promoter	3	4474475	4476991	2517	1
FGRAMPH1_01G19623	FGRAMPH1_01T19623	1.83922	12.22843	Promoter	3	4480680	4482938	2259	1
FGRAMPH1_01G19643	FGRAMPH1_01T19643	1.53492	5.95473	Exon	3	4510083	4511094	1012	2
FGRAMPH1_01G19655	FGRAMPH1_01T19655	2.02772	16.26727	Promoter	3	4520110	4521238	1129	2
FGRAMPH1_01G19657	FGRAMPH1_01T19657	2.16171	19.35629	Intergenic	3	4523541	4524035	495	1
FGRAMPH1_01G19659	FGRAMPH1_01T19659	1.83094	12.36659	Promoter	3	4528352	4532858	4507	1
FGRAMPH1_01G19691	FGRAMPH1_01T19691	1.76104	9.83867	Promoter	3	4571117	4571848	732	2
FGRAMPH1_01G19703	FGRAMPH1_01T19703	1.86677	12.72784	Promoter	3	4592725	4595500	2776	1
FGRAMPH1_01G19709	FGRAMPH1_01T19709	1.92222	12.25882	Promoter	3	4600694	4601650	957	2
FGRAMPH1_01G19711	FGRAMPH1_01T19711	2.0314	17.90596	UTR	3	4601893	4603165	1273	1
FGRAMPH1_01G19713	FGRAMPH1_01T19713	1.94228	13.88935	Promoter	3	4604950	4606056	1107	1
FGRAMPH1_01G19717	FGRAMPH1_01T19717	1.54124	7.49504	Promoter	3	4607535	4612013	4479	1

FGRAMPH1_01G19735	FGRAMPH1_01T19735	1.85396	11.30166	Promoter	3	4636796	4640686	3891	1
FGRAMPH1_01G19761	FGRAMPH1_01T19761	1.72186	8.19781	Promoter	3	4661881	4664988	3108	1
FGRAMPH1_01G19763	FGRAMPH1_01T19763	1.67451	7.34915	Promoter	3	4665431	4666246	816	1
FGRAMPH1_01G19809	FGRAMPH1_01T19809	1.30537	3.40556	Intergenic	3	4718748	4718960	213	1
FGRAMPH1_01G19811	FGRAMPH1_01T19811	1.39363	5.20648	Promoter	3	4721055	4721569	515	2
FGRAMPH1_01G19813	FGRAMPH1_01T19813	1.97376	15.68573	Promoter	3	4724089	4727880	3792	1
FGRAMPH1_01G19881	FGRAMPH1_01T19881	2.03822	14.70381	Promoter	3	4806572	4807023	452	1
FGRAMPH1_01G19883	FGRAMPH1_01T19883	1.29287	3.01521	Exon	3	4807724	4809213	1490	1
FGRAMPH1_01G19885	FGRAMPH1_01T19885	1.83928	12.52116	Promoter	3	4809797	4813315	3519	2
FGRAMPH1_01G19895	FGRAMPH1_01T19895	2.3124	21.592	Promoter	3	4825848	4829011	3164	1
ENSRNA049511465	ENSRNA049511465-T1	2.83193	39.58591	Promoter	3	4859213	4859496	284	2
FGRAMPH1_01G19947	FGRAMPH1_01T19947	1.89058	11.39068	Exon	3	4888927	4891047	2121	2
FGRAMPH1_01G19955	FGRAMPH1_01T19955	1.72469	10.74302	Promoter	3	4898190	4901920	3731	2

FGRAMPH1_01G19957	FGRAMPH1_01T19957	2.21315	23.22906	Promoter	3	4905345	4905581	237	1
ENSRNA049557679	ENSRNA049557679-T1	2.61821	30.75447	Promoter	3	4909373	4909493	121	2
ENSRNA049557661	ENSRNA049557661-T1	2.56548	29.0723	Promoter	3	4910015	4910134	120	2
FGRAMPH1_01G19959	FGRAMPH1_01T19959	1.78784	12.22071	Promoter	3	4910817	4913218	2402	1
FGRAMPH1_01G19975	FGRAMPH1_01T19975	1.97996	14.7856	Promoter	3	4931368	4934087	2720	2
FGRAMPH1_01G19979	FGRAMPH1_01T19979	1.8679	11.88061	Promoter	3	4937251	4940018	2768	1
FGRAMPH1_01G19987	FGRAMPH1_01T19987	1.37789	4.29501	Promoter	3	4945746	4948407	2662	1
ENSRNA049556349	ENSRNA049556349-T1	2.4605	28.26238	Promoter	3	4960316	4960400	85	1
FGRAMPH1_01G20049	FGRAMPH1_01T20049	1.60586	6.11329	Promoter	3	5026102	5030121	4020	1
FGRAMPH1_01G20059	FGRAMPH1_01T20059	1.92289	12.96525	Promoter	3	5044292	5044702	411	1
FGRAMPH1_01G20065	FGRAMPH1_01T20065	1.74552	9.08608	Promoter	3	5052073	5053623	1551	1
FGRAMPH1_01G20081	FGRAMPH1_01T20081	2.47707	31.27451	Promoter	3	5071463	5072082	620	1
FGRAMPH1_01G20097	FGRAMPH1_01T20097	2.0878	19.23189	Promoter	3	5091403	5094830	3428	2

ENSRNA049556369	ENSRNA049556369-T1	2.23269	23.52535	Promoter	3	5102748	5102833	86	1
FGRAMPH1_01G20111	FGRAMPH1_01T20111	1.90452	13.01548	Intergenic	3	5109144	5109497	354	1
FGRAMPH1_01G20113	FGRAMPH1_01T20113	1.5732	9.16102	Promoter	3	5112752	5118248	5497	2
FGRAMPH1_01G20115	FGRAMPH1_01T20115	1.92466	13.86058	Exon	3	5120026	5120676	651	2
FGRAMPH1_01G20123	FGRAMPH1_01T20123	1.93619	13.23639	Intergenic	3	5130863	5131401	539	2
FGRAMPH1_01G20129	FGRAMPH1_01T20129	1.76635	10.08119	Intergenic	3	5136706	5137400	695	2
FGRAMPH1_01G20139	FGRAMPH1_01T20139	2.20381	25.38621	Promoter	3	5151012	5155543	4532	2
FGRAMPH1_01G20147	FGRAMPH1_01T20147	1.93165	14.03219	Promoter	3	5174136	5177768	3633	1
FGRAMPH1_01G20161	FGRAMPH1_01T20161	1.69602	9.33557	Promoter	3	5196468	5198364	1897	1
FGRAMPH1_01G20173	FGRAMPH1_01T20173	1.71737	11.93111	Promoter	3	5212638	5216113	3476	1
FGRAMPH1_01G20195	FGRAMPH1_01T20195	1.59131	8.0864	Promoter	3	5237268	5238793	1526	2
FGRAMPH1_01G20205	FGRAMPH1_01T20205	1.54675	7.06328	Promoter	3	5255129	5255434	306	2
FGRAMPH1_01G20209	FGRAMPH1_01T20209	1.50007	4.62946	Intergenic	3	5261098	5261466	369	1

FGRAMPH1_01G20213	FGRAMPH1_01T20213	1.6805	7.96942	Promoter	3	5266513	5267271	759	1
FGRAMPH1_01G20215	FGRAMPH1_01T20215	2.50741	27.519	Promoter	3	5267822	5270283	2462	1
FGRAMPH1_01G20231	FGRAMPH1_01T20231	1.63637	7.20318	Promoter	3	5285117	5285785	669	1
FGRAMPH1_01G20243	FGRAMPH1_01T20243	2.0804	15.66799	Promoter	3	5300388	5301191	804	1
FGRAMPH1_01G20245	FGRAMPH1_01T20245	2.16476	18.0144	Promoter	3	5302211	5302569	359	1
FGRAMPH1_01G20255	FGRAMPH1_01T20255	1.62695	6.55252	Intergenic	3	5319742	5319966	225	1
FGRAMPH1_01G20261	FGRAMPH1_01T20261	1.85534	11.32418	Promoter	3	5326684	5329492	2809	1
FGRAMPH1_01G20265	FGRAMPH1_01T20265	1.45566	5.11417	Promoter	3	5333553	5335145	1593	1
FGRAMPH1_01G20277	FGRAMPH1_01T20277	1.80199	10.16703	Promoter	3	5346780	5348728	1949	1
FGRAMPH1_01G20283	FGRAMPH1_01T20283	1.47838	4.46376	Promoter	3	5405864	5406339	476	2
FGRAMPH1_01G20311	FGRAMPH1_01T20311	1.80134	9.94027	Promoter	3	5456138	5456986	849	1
FGRAMPH1_01G20313	FGRAMPH1_01T20313	1.6678	7.51282	Exon	3	5457207	5457777	571	2
FGRAMPH1_01G20331	FGRAMPH1_01T20331	1.4305	4.38488	Promoter	3	5476239	5476998	760	1

FGRAMPH1_01G20335	FGRAMPH1_01T20335	1.48058	5.38837	Promoter	3	5481608	5483498	1891	2
FGRAMPH1_01G20341	FGRAMPH1_01T20341	1.83517	10.9947	UTR	3	5487173	5489518	2346	2
FGRAMPH1_01G20345	FGRAMPH1_01T20345	1.54411	5.99484	Promoter	3	5491486	5493028	1543	2
FGRAMPH1_01G20357	FGRAMPH1_01T20357	1.68628	9.26024	Promoter	3	5504163	5505840	1678	2
FGRAMPH1_01G20361	FGRAMPH1_01T20361	1.7943	9.45224	Promoter	3	5507109	5510029	2921	2
FGRAMPH1_01G20369	FGRAMPH1_01T20369	1.43713	4.03119	Exon	3	5520965	5521978	1014	1
FGRAMPH1_01G20371	FGRAMPH1_01T20371	1.74066	9.40999	Promoter	3	5522091	5524747	2657	1
FGRAMPH1_01G20379	FGRAMPH1_01T20379	2.17531	18.0144	Promoter	3	5533952	5534233	282	2
FGRAMPH1_01G20381	FGRAMPH1_01T20381	1.73253	11.08849	Promoter	3	5535364	5536795	1432	2
FGRAMPH1_01G20383	FGRAMPH1_01T20383	1.64279	9.84572	Promoter	3	5538615	5539228	614	2
FGRAMPH1_01G20389	FGRAMPH1_01T20389	1.53926	5.23675	Intergenic	3	5549366	5549808	443	2
ENSRNA049511862	ENSRNA049511862-T1	2.21473	19.95546	Promoter	3	5606415	5606534	120	1
FGRAMPH1_01G20429	FGRAMPH1_01T20429	1.46877	4.13218	UTR	3	5606693	5609314	2622	2

FGRAMPH1_01G20431	FGRAMPH1_01T20431	2.4073	24.63614	Promoter	3	5609002	5609688	687	2
FGRAMPH1_01G20467	FGRAMPH1_01T20467	2.11204	16.65722	Promoter	3	5643785	5643922	138	1
FGRAMPH1_01G20469	FGRAMPH1_01T20469	2.31009	22.24576	Promoter	3	5647601	5650041	2441	1
FGRAMPH1_01G20479	FGRAMPH1_01T20479	1.67751	8.84906	Promoter	3	5664260	5669286	5027	1
FGRAMPH1_01G20485	FGRAMPH1_01T20485	1.51454	5.66375	Promoter	3	5670815	5674241	3427	2
FGRAMPH1_01G20487	FGRAMPH1_01T20487	1.79122	13.69317	Promoter	3	5675686	5676951	1266	2
FGRAMPH1_01G20489	FGRAMPH1_01T20489	2.61821	30.75447	Promoter	3	5682365	5683249	885	1
FGRAMPH1_01G20491	FGRAMPH1_01T20491	2.14266	23.33034	Exon	3	5687095	5687603	509	2
FGRAMPH1_01G20503	FGRAMPH1_01T20503	1.62876	7.12585	Promoter	3	5705996	5707726	1731	1
FGRAMPH1_01G20513	FGRAMPH1_01T20513	1.77401	9.83989	Promoter	3	5714420	5716503	2084	1
FGRAMPH1_01G20515	FGRAMPH1_01T20515	1.4662	6.26461	Promoter	3	5716919	5718763	1845	1
FGRAMPH1_01G20517	FGRAMPH1_01T20517	1.91167	11.96643	Promoter	3	5718855	5719280	426	2
FGRAMPH1_01G20519	FGRAMPH1_01T20519	1.65859	7.00562	Promoter	3	5720267	5722992	2726	1

FGRAMPH1_01G20521	FGRAMPH1_01T20521	2.29131	21.22266	Promoter	3	5723385	5724236	852	1
FGRAMPH1_01G20523	FGRAMPH1_01T20523	1.94139	13.07192	Promoter	3	5725895	5727478	1584	1
FGRAMPH1_01G20527	FGRAMPH1_01T20527	1.81201	11.39556	Promoter	3	5731737	5732180	444	1
FGRAMPH1_01G20533	FGRAMPH1_01T20533	1.66198	8.84182	Exon	3	5738347	5738946	600	1
FGRAMPH1_01G20535	FGRAMPH1_01T20535	1.80622	9.73718	Promoter	3	5741873	5742081	209	1
ENSRNA049556385	ENSRNA049556385-T1	2.70257	33.34432	Promoter	3	5764794	5764865	72	1
FGRAMPH1_01G20563	FGRAMPH1_01T20563	1.79742	10.6531	Promoter	3	5786741	5786902	162	1
FGRAMPH1_01G20583	FGRAMPH1_01T20583	1.61201	9.77447	Promoter	3	5817349	5818106	758	2
FGRAMPH1_01G20585	FGRAMPH1_01T20585	1.47793	5.99259	Intergenic	3	5818365	5818829	465	1
ENSRNA049556408	ENSRNA049556408-T1	2.14367	17.33041	Promoter	3	5823083	5823166	84	1
FGRAMPH1_01G20589	FGRAMPH1_01T20589	1.51009	6.43408	Intergenic	3	5828084	5828296	213	2
ENSRNA049511493	ENSRNA049511493-T1	1.50966	8.66313	Promoter	3	5836584	5836878	295	2
ENSRNA049511533	ENSRNA049511533-T1	1.57767	7.23394	Promoter	3	5855153	5855274	122	2

FGRAMPH1_01G20613	FGRAMPH1_01T20613	1.98506	14.20611	UTR	3	5861581	5863292	1712	2
FGRAMPH1_01G20621	FGRAMPH1_01T20621	1.90846	14.12968	Promoter	3	5867826	5869931	2106	2
FGRAMPH1_01G20623	FGRAMPH1_01T20623	2.2494	24.97669	Promoter	3	5870778	5872431	1654	1
FGRAMPH1_01G20637	FGRAMPH1_01T20637	2.88184	39.6861	Promoter	3	5890152	5890917	766	1
FGRAMPH1_01G20639	FGRAMPH1_01T20639	2.72366	34.22501	Promoter	3	5894586	5896715	2130	1
FGRAMPH1_01G20669	FGRAMPH1_01T20669	1.95041	13.13833	Promoter	3	5929027	5929452	426	1
FGRAMPH1_01G20671	FGRAMPH1_01T20671	1.89737	11.86019	Promoter	3	5931153	5931560	408	1
FGRAMPH1_01G20687	FGRAMPH1_01T20687	1.79568	9.47257	Promoter	3	5950147	5951347	1201	2
FGRAMPH1_01G20711	FGRAMPH1_01T20711	2.49167	27.0208	Promoter	3	5972078	5972326	249	2
FGRAMPH1_01G20713	FGRAMPH1_01T20713	1.49477	5.92079	Promoter	3	5974147	5976677	2531	2
FGRAMPH1_01G20717	FGRAMPH1_01T20717	2.03189	14.87495	Promoter	3	5978411	5979709	1299	2
FGRAMPH1_01G20723	FGRAMPH1_01T20723	1.27261	3.10209	Promoter	3	5990901	5991257	357	2
FGRAMPH1_01G20727	FGRAMPH1_01T20727	1.7479	8.92093	Exon	3	5993979	5994380	402	2

ENSRNA049556428	ENSRNA049556428-T1	2.29648	24.57747	Promoter	3	6007079	6007159	81	1
FGRAMPH1_01G20751	FGRAMPH1_01T20751	1.77081	10.10067	Exon	3	6035392	6037752	2361	1
FGRAMPH1_01G20775	FGRAMPH1_01T20775	2.43894	25.42153	Promoter	3	6070655	6072865	2211	2
ENSRNA049556450	ENSRNA049556450-T1	2.59712	30.33056	Promoter	3	6075020	6075103	84	1
ENSRNA049556473	ENSRNA049556473-T1	2.4073	24.63614	Promoter	3	6075679	6075762	84	1
FGRAMPH1_01G20817	FGRAMPH1_01T20817	1.51354	5.9004	Promoter	3	6123799	6125656	1858	2
ENSRNA049557640	ENSRNA049557640-T1	2.53385	28.2448	Promoter	3	6128272	6128354	83	2
ENSRNA049511672	ENSRNA049511672-T1	2.18585	18.36041	Promoter	3	6136191	6136310	120	2
FGRAMPH1_01G20837	FGRAMPH1_01T20837	1.59933	6.78367	Promoter	3	6148219	6150563	2345	1
FGRAMPH1_01G20839	FGRAMPH1_01T20839	1.51435	6.92053	Promoter	3	6152056	6156158	4103	1
FGRAMPH1_01G20847	FGRAMPH1_01T20847	1.41708	4.31885	Promoter	3	6164069	6165386	1318	1
FGRAMPH1_01G20851	FGRAMPH1_01T20851	1.60329	7.57309	Exon	3	6168788	6169275	488	1
FGRAMPH1_01G20855	FGRAMPH1_01T20855	1.52461	5.87724	UTR	3	6170437	6171493	1057	1

FGRAMPH1_01G20863	FGRAMPH1_01T20863	1.86511	11.78765	Promoter	3	6184342	6186084	1743	1
FGRAMPH1_01G20887	FGRAMPH1_01T20887	1.65496	7.8934	Promoter	3	6205575	6207156	1582	2
FGRAMPH1_01G20893	FGRAMPH1_01T20893	1.74453	8.82018	Promoter	3	6213744	6214355	612	2
FGRAMPH1_01G20897	FGRAMPH1_01T20897	1.63818	7.46663	Promoter	3	6220048	6222460	2413	2
FGRAMPH1_01G20973	FGRAMPH1_01T20973	1.82731	10.2759	Promoter	3	6343958	6344999	1042	2
FGRAMPH1_01G20975	FGRAMPH1_01T20975	1.76404	8.95294	Promoter	3	6346368	6347951	1584	1
FGRAMPH1_01G21041	FGRAMPH1_01T21041	2.04876	15.02239	Promoter	3	6413463	6415920	2458	1
ENSRNA049556504	ENSRNA049556504-T1	1.74295	8.44624	Promoter	3	6419012	6419083	72	1
ENSRNA049557618	ENSRNA049557618-T1	2.48112	26.61745	Promoter	3	6424826	6424920	95	2
FGRAMPH1_01G21101	FGRAMPH1_01T21101	1.66913	7.23732	Promoter	3	6488364	6489554	1191	2
FGRAMPH1_01G21103	FGRAMPH1_01T21103	1.93277	12.25882	Promoter	3	6490807	6491618	812	1
ENSRNA049511507	ENSRNA049511507-T1	1.72186	8.19781	Promoter	3	6491632	6491736	105	2
FGRAMPH1_01G21121	FGRAMPH1_01T21121	1.81121	10.93185	UTR	3	6506395	6509022	2628	2

FGRAMPH1_01G21129	FGRAMPH1_01T21129	1.48986	4.49906	Promoter	3	6516028	6517089	1062	2
FGRAMPH1_01G21131	FGRAMPH1_01T21131	1.60586	6.11329	Intergenic	3	6519048	6519245	198	2
FGRAMPH1_01G21141	FGRAMPH1_01T21141	1.47755	6.34304	Promoter	3	6528175	6531881	3707	2
ENSRNA049556530	ENSRNA049556530-T1	2.53385	28.2448	Promoter	3	6599354	6599453	100	1
ENSRNA049556544	ENSRNA049556544-T1	2.69203	33.34432	Promoter	3	6606086	6606157	72	1
FGRAMPH1_01G21211	FGRAMPH1_01T21211	1.79568	9.47257	Promoter	3	6606359	6607474	1116	2
ENSRNA049557581	ENSRNA049557581-T1	2.64984	31.60897	Promoter	3	6609763	6609844	82	2
ENSRNA049557553	ENSRNA049557553-T1	2.78693	36.46401	Promoter	3	6612169	6612286	118	2
ENSRNA049557502	ENSRNA049557502-T1	2.54439	28.6574	Promoter	3	6613220	6613323	104	2
FGRAMPH1_01G21217	FGRAMPH1_01T21217	1.70483	8.51158	Promoter	3	6619359	6621726	2368	2
ENSRNA049557473	ENSRNA049557473-T1	1.98549	13.45803	Promoter	3	6657108	6657181	74	2
FGRAMPH1_01G21267	FGRAMPH1_01T21267	2.12258	16.65722	Promoter	3	6684135	6685073	939	1
ENSRNA049556561	ENSRNA049556561-T1	2.57603	29.48946	Promoter	3	6694724	6694822	99	1

FGRAMPH1_01G21281	FGRAMPH1_01T21281	1.98549	13.45803	Promoter	3	6696195	6699155	2961	2
FGRAMPH1_01G21285	FGRAMPH1_01T21285	1.47932	4.31375	Promoter	3	6701850	6703562	1713	1
FGRAMPH1_01G21287	FGRAMPH1_01T21287	2.08976	20.33394	Promoter	3	6705635	6708223	2589	1
ENSRNA049557444	ENSRNA049557444-T1	2.23858	19.7708	Promoter	3	6729902	6729973	72	2
ENSRNA049556585	ENSRNA049556585-T1	2.11204	16.65722	Promoter	3	6765555	6765647	93	1
ENSRNA049556608	ENSRNA049556608-T1	1.81191	13.98026	Promoter	3	6780048	6780133	86	1
FGRAMPH1_01G21357	FGRAMPH1_01T21357	1.72657	9.81458	Promoter	3	6781662	6783200	1539	1
ENSRNA049557391	ENSRNA049557391-T1	2.41785	24.63614	Promoter	3	6783457	6783528	72	2
FGRAMPH1_01G21365	FGRAMPH1_01T21365	1.64804	6.77735	Promoter	3	6786645	6787469	825	2
ENSRNA049557365	ENSRNA049557365-T1	2.73421	34.66857	Promoter	3	6809935	6810019	85	2
FGRAMPH1_01G21429	FGRAMPH1_01T21429	1.7535	8.69796	Promoter	3	6852136	6853115	980	1
FGRAMPH1_01G21453	FGRAMPH1_01T21453	1.65859	7.00562	Promoter	3	6877664	6879582	1919	1
FGRAMPH1_01G21487	FGRAMPH1_01T21487	1.7802	10.20457	Exon	3	6917738	6918366	629	1

FGRAMPH1_01G21489	FGRAMPH1_01T21489	1.7846	10.43893	Promoter	3	6919253	6921533	2281	1
ENSRNA049556628	ENSRNA049556628-T1	2.51276	27.42648	Promoter	3	6927439	6927510	72	1
ENSRNA049557332	ENSRNA049557332-T1	2.70257	33.34432	Promoter	3	6941084	6941157	74	2
ENSRNA049511364	ENSRNA049511364-T1	2.0848	18.65856	Exon	3	6947780	6948054	275	1
ENSRNA049557321	ENSRNA049557321-T1	2.4073	24.63614	Promoter	3	6973601	6973672	72	2
ENSRNA049557296	ENSRNA049557296-T1	2.23858	19.7708	Promoter	3	6974173	6974245	73	2
ENSRNA049557255	ENSRNA049557255-T1	2.58657	29.90888	Promoter	3	6982234	6982305	72	2
FGRAMPH1_01G21545	FGRAMPH1_01T21545	1.60586	6.11329	Promoter	3	6982594	6984175	1582	2
ENSRNA049556642	ENSRNA049556642-T1	2.39828	27.13093	Promoter	3	6984590	6984671	82	1
FGRAMPH1_01G21549	FGRAMPH1_01T21549	2.0804	15.66799	Promoter	3	6988724	6989107	384	1
FGRAMPH1_01G21553	FGRAMPH1_01T21553	1.46767	4.87295	Promoter	3	6993440	6997039	3600	1
ENSRNA049557233	ENSRNA049557233-T1	2.67094	32.47228	Promoter	3	7063497	7063581	85	2
FGRAMPH1_01G21599	FGRAMPH1_01T21599	1.77459	9.21114	Promoter	3	7065628	7066986	1359	2

ENSRNA049557217	ENSRNA049557217-T1	2.1964	18.70907	Promoter	3	7086053	7086134	82	2
ENSRNA049557190	ENSRNA049557190-T1	2.46003	25.8178	Promoter	3	7098859	7098957	99	2
FGRAMPH1_01G21639	FGRAMPH1_01T21639	2.13313	16.99246	Promoter	3	7106458	7108209	1752	2
ENSRNA049556657	ENSRNA049556657-T1	1.9644	13.15382	Promoter	3	7132342	7132432	91	1
FGRAMPH1_01G21659	FGRAMPH1_01T21659	1.66913	7.23732	Promoter	3	7136360	7138940	2581	1
FGRAMPH1_01G21667	FGRAMPH1_01T21667	2.18585	18.36041	Promoter	3	7149378	7149644	267	2
ENSRNA049511700	ENSRNA049511700-T1	2.21881	20.32953	Promoter	3	7167157	7167276	120	1
FGRAMPH1_01G21691	FGRAMPH1_01T21691	2.43326	26.22686	Promoter	3	7172605	7173108	504	2
FGRAMPH1_01G21693	FGRAMPH1_01T21693	1.6872	7.61636	Exon	3	7173602	7174550	949	1
ENSRNA049511745	ENSRNA049511745-T1	2.47057	26.21645	Promoter	3	7233960	7234079	120	1
ENSRNA049511607	ENSRNA049511607-T1	2.41785	24.63614	Promoter	3	7261367	7261486	120	1
ENSRNA049556674	ENSRNA049556674-T1	2.20503	19.77242	Promoter	3	7268186	7268267	82	1
ENSRNA049556697	ENSRNA049556697-T1	2.23858	19.7708	Promoter	3	7281135	7281233	99	1

ENSRNA049557179	ENSRNA049557179-T1	2.77639	36.01199	Promoter	3	7281851	7281922	72	2
ENSRNA049556740	ENSRNA049556740-T1	2.29617	22.79382	Promoter	3	7289569	7289651	83	1
ENSRNA049557145	ENSRNA049557145-T1	1.87766	13.30688	Promoter	3	7292179	7292269	91	2
FGRAMPH1_01G21831	FGRAMPH1_01T21831	1.47661	4.69797	Promoter	3	7319414	7321338	1925	1
ENSRNA049556762	ENSRNA049556762-T1	1.73241	8.44624	Promoter	3	7330012	7330083	72	1
ENSRNA049556789	ENSRNA049556789-T1	2.61821	30.75447	Promoter	3	7333884	7333983	100	1
ENSRNA049556803	ENSRNA049556803-T1	2.11204	16.65722	Promoter	3	7361066	7361137	72	1
FGRAMPH1_01G21881	FGRAMPH1_01T21881	1.72439	8.42494	Exon	3	7367489	7368841	1353	1
FGRAMPH1_01G21883	FGRAMPH1_01T21883	1.51664	6.33663	Exon	3	7369200	7369814	615	1
FGRAMPH1_01G21885	FGRAMPH1_01T21885	2.1916	20.51397	Exon	3	7370041	7370286	246	2
ENSRNA049556831	ENSRNA049556831-T1	2.59712	30.33056	Promoter	3	7372484	7372568	85	1
ENSRNA049557123	ENSRNA049557123-T1	2.51276	27.42648	Promoter	3	7377713	7377816	104	2
ENSRNA049557119	ENSRNA049557119-T1	2.3124	21.592	Promoter	3	7380327	7380420	94	2

ENSRNA049511835	ENSRNA049511835-T1	2.56201	30.41459	Promoter	3	7387002	7387121	120	1
ENSRNA049556853	ENSRNA049556853-T1	2.59712	30.33056	Promoter	3	7398672	7398789	118	1
FGRAMPH1_01G21909	FGRAMPH1_01T21909	1.82731	10.2759	Promoter	3	7400202	7400396	195	2
FGRAMPH1_01G21911	FGRAMPH1_01T21911	1.46765	4.11973	Promoter	3	7401058	7403227	2170	2
ENSRNA049557077	ENSRNA049557077-T1	2.38621	23.86038	Promoter	3	7419810	7419908	99	2
FGRAMPH1_01G21935	FGRAMPH1_01T21935	1.82923	13.61036	Promoter	3	7428683	7430400	1718	2
FGRAMPH1_01G21939	FGRAMPH1_01T21939	2.10608	18.97933	Promoter	3	7431985	7434053	2069	1
ENSRNA049557052	ENSRNA049557052-T1	2.09094	15.99497	Promoter	3	7440381	7440452	72	2
ENSRNA049557029	ENSRNA049557029-T1	2.37567	23.47615	Promoter	3	7441050	7441149	100	2
FGRAMPH1_01G21949	FGRAMPH1_01T21949	1.88801	12.21815	Promoter	3	7443888	7445519	1632	2
ENSRNA049556880	ENSRNA049556880-T1	2.28076	20.85586	Promoter	3	7449960	7450045	86	1
FGRAMPH1_01G21961	FGRAMPH1_01T21961	2.25315	23.80746	Promoter	3	7463287	7465561	2275	1
FGRAMPH1_01G21965	FGRAMPH1_01T21965	1.9048	15.49564	Promoter	3	7467664	7468443	780	1

FGRAMPH1_01G21967	FGRAMPH1_01T21967	2.01062	15.07178	Promoter	3	7469059	7470806	1748	2
ENSRNA049557002	ENSRNA049557002-T1	2.13313	16.99246	Promoter	3	7473359	7473442	84	2
FGRAMPH1_01G21989	FGRAMPH1_01T21989	1.7535	8.69796	Promoter	3	7496611	7497390	780	1
ENSRNA049556975	ENSRNA049556975-T1	2.04462	15.3355	Promoter	3	7498451	7498536	86	2
FGRAMPH1_01G22003	FGRAMPH1_01T22003	1.86949	11.10737	Promoter	3	7508839	7509804	966	1
FGRAMPH1_01G22005	FGRAMPH1_01T22005	1.88004	11.39068	Promoter	3	7510522	7511418	897	1
FGRAMPH1_01G22027	FGRAMPH1_01T22027	1.50807	6.87548	Promoter	3	7537524	7538980	1457	2
FGRAMPH1_01G22039	FGRAMPH1_01T22039	1.55314	5.27724	Promoter	3	7553121	7554072	952	2
ENSRNA049556963	ENSRNA049556963-T1	2.57603	29.48946	Promoter	3	7627112	7627185	74	2
FGRAMPH1_01G22129	FGRAMPH1_01T22129	1.51409	5.11553	Promoter	3	7643328	7645016	1689	1
ENSRNA049556897	ENSRNA049556897-T1	2.81857	37.37431	Promoter	3	7647791	7647864	74	1
FGRAMPH1_01G22135	FGRAMPH1_01T22135	2.36512	23.09437	Promoter	3	7648310	7649293	984	2
FGRAMPH1_01G22139	FGRAMPH1_01T22139	2.28076	20.85586	Promoter	3	7653921	7655498	1578	1

ENSRNA049556914	ENSRNA049556914-T1	2.64984	31.60897	Promoter	3	7691355	7691428	74	1
FGRAMPH1_01G22279	FGRAMPH1_01T22279	1.80622	9.73718	Promoter	4	45103	47004	1902	1
FGRAMPH1_01G22283	FGRAMPH1_01T22283	2.32116	21.93051	Promoter	4	51849	52909	1061	1
FGRAMPH1_01G22337	FGRAMPH1_01T22337	2.62572	34.49238	Promoter	4	118560	120340	1781	2
FGRAMPH1_01G22355	FGRAMPH1_01T22355	1.75473	10.39919	Exon	4	145301	145968	668	2
FGRAMPH1_01G22363	FGRAMPH1_01T22363	2.51276	27.42648	Promoter	4	153120	155414	2295	2
FGRAMPH1_01G22367	FGRAMPH1_01T22367	2.47204	27.56455	Promoter	4	158436	158591	156	1
FGRAMPH1_01G22393	FGRAMPH1_01T22393	1.88004	11.39068	Promoter	4	187480	188040	561	2
FGRAMPH1_01G22399	FGRAMPH1_01T22399	2.11204	16.65722	Exon	4	194776	196332	1557	2
FGRAMPH1_01G22421	FGRAMPH1_01T22421	1.43863	3.86665	Promoter	4	229841	230101	261	1
FGRAMPH1_01G22423	FGRAMPH1_01T22423	1.73309	8.74983	Promoter	4	229939	230304	366	2
FGRAMPH1_01G22425	FGRAMPH1_01T22425	2.05931	15.34379	Promoter	4	231636	232189	554	2
FGRAMPH1_01G22427	FGRAMPH1_01T22427	1.97847	15.81895	Promoter	4	233829	233957	129	2

FGRAMPH1_01G22429	FGRAMPH1_01T22429	2.00601	14.20128	Intergenic	4	238553	238711	159	2
FGRAMPH1_01G22433	FGRAMPH1_01T22433	1.67499	9.9427	Promoter	4	245427	247259	1833	1
FGRAMPH1_01G22443	FGRAMPH1_01T22443	1.53383	5.84997	Promoter	4	256133	257131	999	2
FGRAMPH1_01G22445	FGRAMPH1_01T22445	2.38621	23.86038	Promoter	4	257972	259045	1074	2
FGRAMPH1_01G22447	FGRAMPH1_01T22447	1.9644	13.15382	Exon	4	261293	261564	272	2
FGRAMPH1_01G22469	FGRAMPH1_01T22469	2.93457	41.09751	Promoter	4	291317	292864	1548	2
FGRAMPH1_01G22477	FGRAMPH1_01T22477	1.62792	7.28273	Promoter	4	307999	310457	2459	1
FGRAMPH1_01G22505	FGRAMPH1_01T22505	2.08814	18.83722	Promoter	4	331802	333478	1677	1
FGRAMPH1_01G22531	FGRAMPH1_01T22531	2.1964	18.70907	Promoter	4	356955	358918	1964	2
FGRAMPH1_01G22541	FGRAMPH1_01T22541	1.6189	7.71536	Promoter	4	368846	371831	2986	2
FGRAMPH1_01G22579	FGRAMPH1_01T22579	2.21312	19.30544	Promoter	4	412388	413383	996	1
FGRAMPH1_01G22585	FGRAMPH1_01T22585	1.71386	8.50862	Promoter	4	421498	422502	1005	2
FGRAMPH1_01G22591	FGRAMPH1_01T22591	2.41911	25.58196	Promoter	4	430539	432912	2374	2

FGRAMPH1_01G22597	FGRAMPH1_01T22597	1.71794	8.95136	Promoter	4	435629	440008	4380	1
FGRAMPH1_01G22611	FGRAMPH1_01T22611	1.86949	11.10737	Exon	4	460613	461153	541	2
FGRAMPH1_01G22615	FGRAMPH1_01T22615	2.00658	14.07517	Promoter	4	464192	465322	1131	1
FGRAMPH1_01G22623	FGRAMPH1_01T22623	1.83266	10.71156	UTR	4	470728	472629	1902	2
FGRAMPH1_01G22625	FGRAMPH1_01T22625	1.26026	3.00154	Promoter	4	475990	477510	1521	1
FGRAMPH1_01G22627	FGRAMPH1_01T22627	1.65947	8.5858	Promoter	4	477911	480407	2497	1
FGRAMPH1_01G22635	FGRAMPH1_01T22635	1.7729	11.92708	Promoter	4	484180	491812	7633	2
ENSRNA049554727	ENSRNA049554727-T1	2.24465	23.01257	Promoter	4	516519	516618	100	1
FGRAMPH1_01G22661	FGRAMPH1_01T22661	1.74295	8.44624	Promoter	4	532999	534780	1782	2
FGRAMPH1_01G22727	FGRAMPH1_01T22727	2.27997	21.47378	Promoter	4	597430	597645	216	2
FGRAMPH1_01G22731	FGRAMPH1_01T22731	1.99604	13.76516	Promoter	4	600262	603392	3131	1
FGRAMPH1_01G22743	FGRAMPH1_01T22743	2.88184	39.6861	Promoter	4	614293	615222	930	2
FGRAMPH1_01G22765	FGRAMPH1_01T22765	1.39013	3.60021	Intron	4	634994	635816	823	2

FGRAMPH1_01G22787	FGRAMPH1_01T22787	1.29088	3.10308	Promoter	4	665051	668387	3337	1
ENSRNA049554778	ENSRNA049554778-T1	2.39294	27.53281	Promoter	4	672052	672142	91	1
FGRAMPH1_01G22795	FGRAMPH1_01T22795	1.57508	6.45889	Promoter	4	675898	676125	228	1
FGRAMPH1_01G22797	FGRAMPH1_01T22797	1.46665	4.73537	Promoter	4	675936	676525	590	2
FGRAMPH1_01G22819	FGRAMPH1_01T22819	1.56618	6.11182	Promoter	4	707727	709847	2121	2
FGRAMPH1_01G22831	FGRAMPH1_01T22831	1.54743	8.2865	Intergenic	4	728666	729267	602	2
FGRAMPH1_01G22863	FGRAMPH1_01T22863	1.93447	14.50018	Promoter	4	789143	792015	2873	2
FGRAMPH1_01G22865	FGRAMPH1_01T22865	1.29067	3.03259	Exon	4	793153	793915	763	2
FGRAMPH1_01G22867	FGRAMPH1_01T22867	1.81762	11.61807	Exon	4	794119	794534	416	1
FGRAMPH1_01G22917	FGRAMPH1_01T22917	1.6989	8.37821	Promoter	4	861148	865425	4278	2
FGRAMPH1_01G22929	FGRAMPH1_01T22929	1.81116	10.26181	UTR	4	876003	877229	1227	1
FGRAMPH1_01G22949	FGRAMPH1_01T22949	1.95279	16.59979	Promoter	4	905516	907665	2150	1
FGRAMPH1_01G22951	FGRAMPH1_01T22951	1.88869	13.3198	Intergenic	4	908956	909117	162	2

FGRAMPH1_01G22965	FGRAMPH1_01T22965	2.28122	25.22062	Promoter	4	925648	925935	288	1
FGRAMPH1_01G22981	FGRAMPH1_01T22981	1.73141	10.14172	Promoter	4	946184	948800	2617	2
FGRAMPH1_01G22983	FGRAMPH1_01T22983	1.58365	7.82012	Promoter	4	950476	950991	516	1
FGRAMPH1_01G22987	FGRAMPH1_01T22987	1.66794	8.21874	Exon	4	956938	957236	299	2
FGRAMPH1_01G22995	FGRAMPH1_01T22995	1.80622	9.73718	Promoter	4	962223	964150	1928	2
FGRAMPH1_01G23013	FGRAMPH1_01T23013	1.4207	5.29044	Promoter	4	983625	984135	511	2
FGRAMPH1_01G23025	FGRAMPH1_01T23025	2.13563	19.30523	Promoter	4	999102	1000196	1095	1
FGRAMPH1_01G23033	FGRAMPH1_01T23033	2.68148	32.90722	Promoter	4	1007432	1008679	1248	1
ENSRNA049554831	ENSRNA049554831-T1	2.62291	31.27047	Promoter	4	1008364	1008453	90	1
FGRAMPH1_01G23045	FGRAMPH1_01T23045	1.33109	4.09855	Promoter	4	1022599	1026585	3987	2
FGRAMPH1_01G23047	FGRAMPH1_01T23047	2.23122	25.59652	Promoter	4	1029419	1033307	3889	1
FGRAMPH1_01G23061	FGRAMPH1_01T23061	1.58477	5.89894	Intergenic	4	1047090	1047482	393	2
FGRAMPH1_01G23063	FGRAMPH1_01T23063	1.65859	7.00562	Intergenic	4	1049741	1050012	272	2

FGRAMPH1_01G23065	FGRAMPH1_01T23065	2.16127	19.65843	Promoter	4	1051582	1054153	2572	1
FGRAMPH1_01G23067	FGRAMPH1_01T23067	1.77503	9.43232	Promoter	4	1055644	1060910	5267	2
FGRAMPH1_01G23069	FGRAMPH1_01T23069	2.32534	22.515	Promoter	4	1062239	1063273	1035	2
FGRAMPH1_01G23075	FGRAMPH1_01T23075	1.75284	11.93876	Promoter	4	1067396	1069317	1922	2
FGRAMPH1_01G23087	FGRAMPH1_01T23087	2.15214	18.30938	Promoter	4	1083607	1085634	2028	1
FGRAMPH1_01G23089	FGRAMPH1_01T23089	2.05318	16.50396	Promoter	4	1086726	1086908	183	1
ENSRNA049555933	ENSRNA049555933-T1	2.80802	36.91812	Promoter	4	1088215	1088317	103	2
ENSRNA049555914	ENSRNA049555914-T1	2.25967	20.49161	Promoter	4	1095274	1095346	73	2
FGRAMPH1_01G23105	FGRAMPH1_01T23105	1.48318	4.7262	Promoter	4	1110600	1112074	1475	2
FGRAMPH1_01G23123	FGRAMPH1_01T23123	2.00865	16.27287	Promoter	4	1134597	1135603	1007	1
FGRAMPH1_01G23125	FGRAMPH1_01T23125	1.71131	7.95267	Promoter	4	1136888	1136998	111	2
FGRAMPH1_01G23133	FGRAMPH1_01T23133	1.73241	8.44624	Promoter	4	1148256	1148524	269	2
FGRAMPH1_01G23135	FGRAMPH1_01T23135	1.66032	8.72494	Promoter	4	1150706	1152085	1380	1

FGRAMPH1_01G23145	FGRAMPH1_01T23145	2.34583	23.58025	Promoter	4	1164755	1169367	4613	1
FGRAMPH1_01G23147	FGRAMPH1_01T23147	1.66913	7.23732	Promoter	4	1171790	1174482	2693	1
FGRAMPH1_01G23149	FGRAMPH1_01T23149	1.79992	10.52777	Exon	4	1175886	1176864	979	1
FGRAMPH1_01G23151	FGRAMPH1_01T23151	2.16476	18.0144	Intergenic	4	1178478	1179160	683	2
FGRAMPH1_01G23155	FGRAMPH1_01T23155	2.20694	19.06036	Promoter	4	1183739	1184018	280	1
FGRAMPH1_01G23169	FGRAMPH1_01T23169	1.59695	8.42604	Exon	4	1203622	1203933	312	1
FGRAMPH1_01G23183	FGRAMPH1_01T23183	1.33354	3.32801	Promoter	4	1221391	1222590	1200	2
FGRAMPH1_01G23197	FGRAMPH1_01T23197	1.61967	7.38801	Promoter	4	1242402	1245123	2722	2
FGRAMPH1_01G23227	FGRAMPH1_01T23227	1.52993	6.23536	Promoter	4	1288592	1291111	2520	1
FGRAMPH1_01G23235	FGRAMPH1_01T23235	2.06985	15.66799	Promoter	4	1300816	1301590	775	1
FGRAMPH1_01G23237	FGRAMPH1_01T23237	1.94239	13.55349	Promoter	4	1302996	1307367	4372	1
FGRAMPH1_01G23239	FGRAMPH1_01T23239	1.6997	8.20206	Promoter	4	1309642	1313502	3861	1
FGRAMPH1_01G23275	FGRAMPH1_01T23275	1.28921	3.13871	Promoter	4	1362046	1364539	2494	1

FGRAMPH1_01G23283	FGRAMPH1_01T23283	1.78977	11.17155	Exon	4	1373509	1373868	360	1
FGRAMPH1_01G23287	FGRAMPH1_01T23287	1.874	12.66952	Promoter	4	1376965	1381352	4388	1
FGRAMPH1_01G23319	FGRAMPH1_01T23319	1.4808	5.60454	UTR	4	1430531	1431662	1132	2
FGRAMPH1_01G23351	FGRAMPH1_01T23351	1.56874	6.11627	Exon	4	1462779	1463718	940	1
FGRAMPH1_01G23355	FGRAMPH1_01T23355	1.71448	9.17939	Promoter	4	1467305	1470851	3547	2
FGRAMPH1_01G23375	FGRAMPH1_01T23375	1.41347	6.06164	Promoter	4	1502604	1505824	3221	2
FGRAMPH1_01G23377	FGRAMPH1_01T23377	1.47635	7.08161	Exon	4	1506124	1508593	2470	1
FGRAMPH1_01G23379	FGRAMPH1_01T23379	1.75665	11.45478	Intergenic	4	1510762	1511043	282	1
FGRAMPH1_01G23395	FGRAMPH1_01T23395	1.52513	6.76901	Promoter	4	1536709	1540508	3800	1
FGRAMPH1_01G23401	FGRAMPH1_01T23401	1.56368	5.48089	Promoter	4	1542339	1544739	2401	2
FGRAMPH1_01G23403	FGRAMPH1_01T23403	1.73241	8.44624	Promoter	4	1545858	1551576	5719	1
FGRAMPH1_01G23407	FGRAMPH1_01T23407	1.47932	4.31375	Promoter	4	1553012	1556347	3336	2
FGRAMPH1_01G23415	FGRAMPH1_01T23415	1.66032	8.72494	Promoter	4	1566170	1571809	5640	1

FGRAMPH1_01G23433	FGRAMPH1_01T23433	2.10175	19.82011	Promoter	4	1591898	1593841	1944	2
FGRAMPH1_01G23435	FGRAMPH1_01T23435	2.0644	21.54452	Promoter	4	1597711	1598184	474	1
FGRAMPH1_01G23439	FGRAMPH1_01T23439	1.38188	3.65464	UTR	4	1605838	1607980	2143	1
FGRAMPH1_01G23445	FGRAMPH1_01T23445	2.10877	16.97703	Promoter	4	1612366	1612671	306	1
FGRAMPH1_01G23447	FGRAMPH1_01T23447	1.80153	10.27862	Promoter	4	1614323	1616212	1890	1
FGRAMPH1_01G23451	FGRAMPH1_01T23451	1.59901	9.50133	Promoter	4	1618983	1619558	576	1
FGRAMPH1_01G23453	FGRAMPH1_01T23453	1.60036	10.51485	Promoter	4	1623411	1624160	750	1
FGRAMPH1_01G23467	FGRAMPH1_01T23467	1.66969	8.79516	Promoter	4	1643983	1645797	1815	2
FGRAMPH1_01G23481	FGRAMPH1_01T23481	1.69872	8.18746	Promoter	4	1666271	1666602	332	1
FGRAMPH1_01G23483	FGRAMPH1_01T23483	2.07341	15.94437	Exon	4	1668553	1669283	731	2
FGRAMPH1_01G23493	FGRAMPH1_01T23493	1.87492	15.54805	Promoter	4	1682047	1683114	1068	2
FGRAMPH1_01G23501	FGRAMPH1_01T23501	1.62021	8.21151	UTR	4	1696499	1697474	976	1
ENSRNA049554849	ENSRNA049554849-T1	2.6393	31.60897	Promoter	4	1720621	1720729	109	1

ENSRNA049554867	ENSRNA049554867-T1	2.48112	26.61745	Promoter	4	1721682	1721790	109	1
FGRAMPH1_01G23539	FGRAMPH1_01T23539	2.0039	17.96286	Promoter	4	1746660	1747133	474	1
FGRAMPH1_01G23541	FGRAMPH1_01T23541	1.94743	14.74582	UTR	4	1747604	1751064	3461	1
FGRAMPH1_01G23545	FGRAMPH1_01T23545	1.48521	4.74017	Promoter	4	1751532	1755050	3519	2
FGRAMPH1_01G23557	FGRAMPH1_01T23557	1.37444	4.38982	Promoter	4	1765497	1767457	1961	2
FGRAMPH1_01G23565	FGRAMPH1_01T23565	1.56173	6.9751	Promoter	4	1774530	1775075	546	1
FGRAMPH1_01G23563	FGRAMPH1_01T23563	1.64037	7.05469	Promoter	4	1774398	1774685	288	2
FGRAMPH1_01G23569	FGRAMPH1_01T23569	1.64804	6.77735	Promoter	4	1784626	1789879	5254	2
FGRAMPH1_01G23575	FGRAMPH1_01T23575	1.44671	4.20283	Promoter	4	1793442	1793756	315	1
FGRAMPH1_01G23577	FGRAMPH1_01T23577	1.37106	3.47431	Promoter	4	1794003	1795586	1584	1
FGRAMPH1_01G23623	FGRAMPH1_01T23623	1.81454	10.03318	Promoter	4	1860625	1863360	2736	2
FGRAMPH1_01G23629	FGRAMPH1_01T23629	1.42696	5.16232	Exon	4	1870178	1871319	1142	1
FGRAMPH1_01G23631	FGRAMPH1_01T23631	1.34256	3.70013	Promoter	4	1873622	1875744	2123	2

FGRAMPH1_01G23635	FGRAMPH1_01T23635	1.61866	7.98953	Promoter	4	1877894	1879975	2082	2
FGRAMPH1_01G23647	FGRAMPH1_01T23647	1.93277	12.25882	Promoter	4	1897136	1902177	5042	2
FGRAMPH1_01G23653	FGRAMPH1_01T23653	2.10149	16.32472	Exon	4	1907060	1907928	869	1
FGRAMPH1_01G23655	FGRAMPH1_01T23655	2.15317	21.15743	Intergenic	4	1909524	1909878	355	2
FGRAMPH1_01G23681	FGRAMPH1_01T23681	1.90113	11.67704	Promoter	4	1938795	1939574	780	2
FGRAMPH1_01G23683	FGRAMPH1_01T23683	1.72713	10.54785	Promoter	4	1942617	1943282	666	1
ENSRNA049518318	ENSRNA049518318-T1	1.99604	13.76516	Promoter	4	1945550	1945669	120	2
FGRAMPH1_01G23711	FGRAMPH1_01T23711	2.33218	23.84098	Promoter	4	1985671	1987671	2001	2
FGRAMPH1_01G23723	FGRAMPH1_01T23723	1.66959	8.37221	Promoter	4	2004063	2007098	3036	2
FGRAMPH1_01G23745	FGRAMPH1_01T23745	1.553	6.04661	Promoter	4	2035874	2039157	3284	1
FGRAMPH1_01G23749	FGRAMPH1_01T23749	2.10266	16.45823	Promoter	4	2041417	2043331	1915	2
FGRAMPH1_01G23757	FGRAMPH1_01T23757	1.59917	7.85279	UTR	4	2050450	2055567	5118	1
FGRAMPH1_01G23765	FGRAMPH1_01T23765	1.88953	12.55804	Exon	4	2063372	2063930	559	2

FGRAMPH1_01G23771	FGRAMPH1_01T23771	1.65859	7.00562	Intergenic	4	2068993	2069249	257	1
FGRAMPH1_01G23777	FGRAMPH1_01T23777	2.0483	17.18208	Promoter	4	2079276	2081234	1959	1
ENSRNA049555896	ENSRNA049555896-T1	2.63654	34.10197	Promoter	4	2096067	2096169	103	2
FGRAMPH1_01G23801	FGRAMPH1_01T23801	2.20968	20.4485	Promoter	4	2114959	2115627	669	1
FGRAMPH1_01G23803	FGRAMPH1_01T23803	2.09094	17.86665	Promoter	4	2116996	2119176	2181	1
FGRAMPH1_01G23817	FGRAMPH1_01T23817	1.79888	10.23628	Promoter	4	2133085	2133247	163	1
FGRAMPH1_01G23835	FGRAMPH1_01T23835	1.7535	8.69796	Promoter	4	2159888	2160250	363	1
FGRAMPH1_01G23837	FGRAMPH1_01T23837	1.56117	9.05355	Promoter	4	2161516	2164297	2782	2
FGRAMPH1_01G23839	FGRAMPH1_01T23839	1.68908	10.15919	Promoter	4	2167557	2168072	516	1
FGRAMPH1_01G23861	FGRAMPH1_01T23861	1.5888	8.61347	Promoter	4	2196345	2198358	2014	2
FGRAMPH1_01G23865	FGRAMPH1_01T23865	2.08414	21.00813	Promoter	4	2200908	2202352	1445	2
FGRAMPH1_01G23867	FGRAMPH1_01T23867	1.66667	7.54854	Promoter	4	2206431	2211002	4572	1
FGRAMPH1_01G23873	FGRAMPH1_01T23873	1.70077	7.71086	Promoter	4	2215474	2215851	378	2

FGRAMPH1_01G23879	FGRAMPH1_01T23879	1.84024	13.48028	Promoter	4	2220256	2220450	195	2
FGRAMPH1_01G23881	FGRAMPH1_01T23881	2.13523	19.1451	Promoter	4	2222271	2223172	902	2
FGRAMPH1_01G23887	FGRAMPH1_01T23887	1.87669	12.71749	Promoter	4	2235883	2237973	2091	1
FGRAMPH1_01G23891	FGRAMPH1_01T23891	1.91167	11.96643	Promoter	4	2241795	2243043	1249	2
FGRAMPH1_01G23895	FGRAMPH1_01T23895	1.9644	13.15382	Promoter	4	2249319	2250877	1559	1
FGRAMPH1_01G23899	FGRAMPH1_01T23899	1.5484	7.11117	Promoter	4	2254484	2258602	4119	1
FGRAMPH1_01G23917	FGRAMPH1_01T23917	1.47528	6.63527	Promoter	4	2289446	2289988	543	1
FGRAMPH1_01G23927	FGRAMPH1_01T23927	1.72186	8.19781	Promoter	4	2303511	2306829	3319	2
FGRAMPH1_01G23929	FGRAMPH1_01T23929	1.69022	7.4724	Exon	4	2307789	2308157	369	2
FGRAMPH1_01G23951	FGRAMPH1_01T23951	2.40898	26.26132	Promoter	4	2327848	2328381	534	1
FGRAMPH1_01G23953	FGRAMPH1_01T23953	2.07061	15.89514	Promoter	4	2330153	2333900	3748	1
FGRAMPH1_01G23955	FGRAMPH1_01T23955	1.72243	12.6607	Promoter	4	2330838	2331071	234	2
FGRAMPH1_01G23995	FGRAMPH1_01T23995	1.75466	13.24231	Promoter	4	2401146	2404629	3484	2

FGRAMPH1_01G23997	FGRAMPH1_01T23997	2.55494	28.6574	Promoter	4	2407128	2407571	444	2
FGRAMPH1_01G23999	FGRAMPH1_01T23999	2.56548	29.0723	Promoter	4	2409541	2409827	287	1
FGRAMPH1_01G24003	FGRAMPH1_01T24003	1.76757	10.72968	Promoter	4	2410453	2418152	7700	2
FGRAMPH1_01G24005	FGRAMPH1_01T24005	1.78545	10.3398	Promoter	4	2420662	2420907	246	1
FGRAMPH1_01G24007	FGRAMPH1_01T24007	2.2466	24.72043	Promoter	4	2422798	2423214	417	2
FGRAMPH1_01G24013	FGRAMPH1_01T24013	1.60197	7.7121	Promoter	4	2436708	2439727	3020	1
FGRAMPH1_01G24023	FGRAMPH1_01T24023	1.29199	3.4015	Promoter	4	2449580	2451499	1920	1
ENSRNA049554884	ENSRNA049554884-T1	2.72366	34.22501	Promoter	4	2460502	2460602	101	1
FGRAMPH1_01G24031	FGRAMPH1_01T24031	2.43923	26.17251	Promoter	4	2460831	2462997	2167	2
FGRAMPH1_01G24033	FGRAMPH1_01T24033	2.38346	24.85201	Promoter	4	2465292	2465930	639	1
FGRAMPH1_01G24037	FGRAMPH1_01T24037	1.46683	5.48325	Exon	4	2470579	2470962	384	2
FGRAMPH1_01G24039	FGRAMPH1_01T24039	1.58617	7.34744	Promoter	4	2472288	2475835	3548	2
FGRAMPH1_01G24047	FGRAMPH1_01T24047	1.43298	6.1599	Promoter	4	2487146	2488600	1455	1

FGRAMPH1_01G24075	FGRAMPH1_01T24075	2.19113	21.68737	Promoter	4	2526465	2527250	786	2
FGRAMPH1_01G24077	FGRAMPH1_01T24077	1.39466	3.97031	Exon	4	2527430	2528473	1044	2
FGRAMPH1_01G24079	FGRAMPH1_01T24079	2.03397	20.08541	Promoter	4	2530216	2530413	198	1
FGRAMPH1_01G24081	FGRAMPH1_01T24081	1.32342	4.11268	Exon	4	2538322	2539631	1310	2
FGRAMPH1_01G24089	FGRAMPH1_01T24089	1.53696	6.23548	Promoter	4	2545910	2546215	306	1
FGRAMPH1_01G24099	FGRAMPH1_01T24099	1.73983	11.52636	Exon	4	2564671	2564934	264	2
FGRAMPH1_01G24109	FGRAMPH1_01T24109	1.55708	5.59229	Promoter	4	2576531	2577869	1339	2
FGRAMPH1_01G24135	FGRAMPH1_01T24135	2.09094	15.99497	Promoter	4	2606725	2609774	3050	2
FGRAMPH1_01G24137	FGRAMPH1_01T24137	1.77459	9.21114	Exon	4	2610846	2611706	861	1
FGRAMPH1_01G24149	FGRAMPH1_01T24149	1.53204	5.0772	Promoter	4	2632785	2633172	388	2
FGRAMPH1_01G24151	FGRAMPH1_01T24151	1.86949	11.10737	Promoter	4	2633424	2636119	2696	2
FGRAMPH1_01G24155	FGRAMPH1_01T24155	2.03822	14.70381	Exon	4	2640563	2641613	1051	1
FGRAMPH1_01G24157	FGRAMPH1_01T24157	1.70461	9.76666	Promoter	4	2646323	2653057	6735	1

FGRAMPH1_01G24189	FGRAMPH1_01T24189	2.05372	20.54903	Promoter	4	2687065	2687223	159	2
FGRAMPH1_01G24191	FGRAMPH1_01T24191	1.6375	6.77735	Promoter	4	2687702	2687917	216	2
FGRAMPH1_01G24193	FGRAMPH1_01T24193	1.65728	7.57541	Promoter	4	2689328	2689960	633	2
FGRAMPH1_01G24195	FGRAMPH1_01T24195	2.32294	21.96385	Promoter	4	2690543	2695242	4700	1
FGRAMPH1_01G24197	FGRAMPH1_01T24197	1.5754	8.1043	Promoter	4	2694341	2694652	312	2
FGRAMPH1_01G24199	FGRAMPH1_01T24199	1.87094	11.4365	Exon	4	2699784	2700096	313	2
FGRAMPH1_01G24211	FGRAMPH1_01T24211	2.27123	21.30674	Promoter	4	2727565	2728247	683	2
FGRAMPH1_01G24221	FGRAMPH1_01T24221	1.52619	5.60212	Promoter	4	2735702	2737311	1610	1
FGRAMPH1_01G24225	FGRAMPH1_01T24225	1.36482	3.63939	Promoter	4	2743382	2747693	4312	1
FGRAMPH1_01G24241	FGRAMPH1_01T24241	1.53359	5.68601	UTR	4	2765485	2766346	862	1
FGRAMPH1_01G24243	FGRAMPH1_01T24243	1.58733	7.15882	Promoter	4	2768394	2771949	3556	1
FGRAMPH1_01G24259	FGRAMPH1_01T24259	1.50437	5.61503	Promoter	4	2783284	2787362	4079	2
FGRAMPH1_01G24261	FGRAMPH1_01T24261	1.41459	5.58011	Promoter	4	2787765	2788159	395	2

FGRAMPH1_01G24263	FGRAMPH1_01T24263	1.65667	7.01631	Exon	4	2789149	2789553	405	2
FGRAMPH1_01G24269	FGRAMPH1_01T24269	2.18088	24.69317	Promoter	4	2797552	2798712	1161	1
FGRAMPH1_01G24271	FGRAMPH1_01T24271	2.47057	26.21645	Promoter	4	2798605	2799587	983	1
FGRAMPH1_01G24273	FGRAMPH1_01T24273	1.69727	7.85076	Promoter	4	2799882	2806134	6253	1
FGRAMPH1_01G24295	FGRAMPH1_01T24295	1.4082	3.69685	Exon	4	2826980	2828895	1916	1
FGRAMPH1_01G24303	FGRAMPH1_01T24303	1.73241	8.44624	Promoter	4	2844321	2846893	2573	1
FGRAMPH1_01G24309	FGRAMPH1_01T24309	1.66444	7.60631	Promoter	4	2848943	2850620	1678	2
FGRAMPH1_01G24327	FGRAMPH1_01T24327	1.78572	9.48877	Promoter	4	2877961	2878450	490	2
FGRAMPH1_01G24349	FGRAMPH1_01T24349	1.53573	5.32003	Promoter	4	2903509	2906480	2972	1
FGRAMPH1_01G24379	FGRAMPH1_01T24379	1.54541	6.38245	Promoter	4	2954276	2957021	2746	2
FGRAMPH1_01G24409	FGRAMPH1_01T24409	1.85102	11.43654	Promoter	4	3001697	3005277	3581	2
FGRAMPH1_01G24435	FGRAMPH1_01T24435	1.76037	10.49695	Promoter	4	3033628	3034957	1330	1
FGRAMPH1_01G24439	FGRAMPH1_01T24439	1.6069	6.67368	Promoter	4	3037285	3039375	2091	2

FGRAMPH1_01G24441	FGRAMPH1_01T24441	1.40243	3.85271	Exon	4	3039817	3041163	1347	2
FGRAMPH1_01G24445	FGRAMPH1_01T24445	1.53878	5.249	Exon	4	3042232	3042747	516	2
ENSRNA049555881	ENSRNA049555881-T1	2.56548	29.0723	Promoter	4	3056461	3056555	95	2
FGRAMPH1_01G24459	FGRAMPH1_01T24459	2.36512	23.09437	Promoter	4	3063094	3063825	732	1
FGRAMPH1_01G24461	FGRAMPH1_01T24461	2.15317	21.15743	Promoter	4	3065491	3065832	342	2
FGRAMPH1_01G24463	FGRAMPH1_01T24463	2.11204	16.65722	Promoter	4	3067629	3068819	1191	1
FGRAMPH1_01G24489	FGRAMPH1_01T24489	1.5618	6.73012	Intergenic	4	3095505	3095917	413	1
ENSRNA049554901	ENSRNA049554901-T1	2.50221	27.42648	Promoter	4	3113080	3113151	72	1
FGRAMPH1_01G24517	FGRAMPH1_01T24517	1.86411	14.02977	Promoter	4	3126145	3127451	1307	2
FGRAMPH1_01G24519	FGRAMPH1_01T24519	1.71406	8.9413	Promoter	4	3129145	3130344	1200	1
FGRAMPH1_01G24523	FGRAMPH1_01T24523	2.32294	21.96385	Promoter	4	3132838	3134371	1534	2
FGRAMPH1_01G24527	FGRAMPH1_01T24527	1.43653	5.00644	Promoter	4	3135881	3140793	4913	1
FGRAMPH1_01G24535	FGRAMPH1_01T24535	2.09311	16.69526	Promoter	4	3150035	3154769	4735	2

FGRAMPH1_01G24543	FGRAMPH1_01T24543	1.54429	5.94409	Promoter	4	3167464	3171132	3669	2
FGRAMPH1_01G24545	FGRAMPH1_01T24545	1.55488	8.22878	Promoter	4	3171506	3172628	1123	2
FGRAMPH1_01G24549	FGRAMPH1_01T24549	1.29535	3.20887	Promoter	4	3175019	3175660	642	2
FGRAMPH1_01G24565	FGRAMPH1_01T24565	1.70077	7.71086	Exon	4	3190908	3191285	378	2
FGRAMPH1_01G24569	FGRAMPH1_01T24569	1.66592	8.38887	Exon	4	3194412	3194624	213	2
FGRAMPH1_01G24571	FGRAMPH1_01T24571	1.90113	11.67704	Promoter	4	3194773	3196164	1392	2
FGRAMPH1_01G24573	FGRAMPH1_01T24573	2.66526	35.60403	Intergenic	4	3205072	3205473	402	2
FGRAMPH1_01G24587	FGRAMPH1_01T24587	1.58781	6.35569	Promoter	4	3221345	3223706	2362	1
FGRAMPH1_01G24593	FGRAMPH1_01T24593	1.90395	12.31946	Promoter	4	3232858	3234599	1742	2
FGRAMPH1_01G24595	FGRAMPH1_01T24595	1.30346	3.22744	Promoter	4	3235933	3237931	1999	1
FGRAMPH1_01G24615	FGRAMPH1_01T24615	1.40934	3.99395	Promoter	4	3265563	3266726	1164	1
FGRAMPH1_01G24619	FGRAMPH1_01T24619	2.79748	36.46401	Promoter	4	3274780	3275445	666	2
FGRAMPH1_01G24629	FGRAMPH1_01T24629	2.0202	14.40023	Promoter	4	3284230	3286524	2295	2

FGRAMPH1_01G24631	FGRAMPH1_01T24631	1.71131	7.95267	Promoter	4	3287580	3290729	3150	2
FGRAMPH1_01G24633	FGRAMPH1_01T24633	1.34445	3.67698	Promoter	4	3292848	3294448	1601	2
FGRAMPH1_01G24635	FGRAMPH1_01T24635	1.85708	11.09881	Promoter	4	3294861	3295834	974	2
FGRAMPH1_01G24637	FGRAMPH1_01T24637	1.94331	12.55419	Promoter	4	3296719	3297243	525	1
FGRAMPH1_01G24639	FGRAMPH1_01T24639	1.78952	9.54602	Promoter	4	3301697	3307319	5623	2
ENSRNA049555824	ENSRNA049555824-T1	2.57603	29.48946	Promoter	4	3309112	3309237	126	2
FGRAMPH1_01G24647	FGRAMPH1_01T24647	2.69203	33.34432	Promoter	4	3315999	3318576	2578	1
FGRAMPH1_01G24649	FGRAMPH1_01T24649	1.68277	9.99905	Promoter	4	3320058	3320279	222	1
FGRAMPH1_01G24651	FGRAMPH1_01T24651	2.08041	18.27971	Promoter	4	3323087	3326878	3792	1
FGRAMPH1_01G24655	FGRAMPH1_01T24655	2.30156	23.86882	Promoter	4	3327867	3329316	1450	1
FGRAMPH1_01G24685	FGRAMPH1_01T24685	2.11204	16.65722	Promoter	4	3365729	3370488	4760	2
ENSRNA049518321	ENSRNA049518321-T1	2.58657	29.90888	Promoter	4	3374545	3374664	120	2
ENSRNA049554923	ENSRNA049554923-T1	2.44948	25.8178	Promoter	4	3382015	3382104	90	1

ENSRNA049555801	ENSRNA049555801-T1	1.99604	13.76516	Promoter	4	3382540	3382613	74	2
FGRAMPH1_01G24703	FGRAMPH1_01T24703	1.45254	5.05318	Promoter	4	3392330	3393269	940	2
FGRAMPH1_01G24705	FGRAMPH1_01T24705	2.49167	27.0208	Promoter	4	3395474	3395686	213	1
ENSRNA049518325	ENSRNA049518325-T1	2.06985	15.66799	Promoter	4	3413682	3413801	120	1
FGRAMPH1_01G24735	FGRAMPH1_01T24735	1.40747	4.18919	Promoter	4	3422572	3423379	808	2
FGRAMPH1_01G24741	FGRAMPH1_01T24741	2.39676	24.24705	Intergenic	4	3430252	3430542	291	2
FGRAMPH1_01G24743	FGRAMPH1_01T24743	1.62749	8.05308	Promoter	4	3435371	3436824	1454	2
FGRAMPH1_01G24749	FGRAMPH1_01T24749	1.7131	9.16281	Promoter	4	3441349	3442389	1041	1
FGRAMPH1_01G24753	FGRAMPH1_01T24753	2.0306	14.57548	Promoter	4	3446150	3448696	2547	2
FGRAMPH1_01G24765	FGRAMPH1_01T24765	1.58477	5.89894	Promoter	4	3465308	3466409	1102	1
FGRAMPH1_01G24769	FGRAMPH1_01T24769	1.46683	5.48325	Promoter	4	3468506	3469482	977	2
FGRAMPH1_01G24773	FGRAMPH1_01T24773	1.64279	9.84572	Promoter	4	3470219	3472506	2288	2
FGRAMPH1_01G24779	FGRAMPH1_01T24779	1.61863	6.86682	UTR	4	3479673	3482496	2824	1

FGRAMPH1_01G24795	FGRAMPH1_01T24795	2.51906	28.73837	UTR	4	3498070	3499617	1548	2
FGRAMPH1_01G24801	FGRAMPH1_01T24801	1.58166	6.63187	Exon	4	3501897	3502564	668	1
FGRAMPH1_01G24885	FGRAMPH1_01T24885	1.32967	3.35508	Promoter	4	3601237	3606015	4779	2
FGRAMPH1_01G24887	FGRAMPH1_01T24887	1.64837	8.90314	Promoter	4	3607566	3609982	2417	1
FGRAMPH1_01G24889	FGRAMPH1_01T24889	1.50235	6.3068	Promoter	4	3611152	3613781	2630	1
FGRAMPH1_01G24905	FGRAMPH1_01T24905	1.91324	14.54526	Promoter	4	3632150	3633251	1102	2
FGRAMPH1_01G24907	FGRAMPH1_01T24907	1.60439	6.85668	Promoter	4	3634321	3637666	3346	1
FGRAMPH1_01G24933	FGRAMPH1_01T24933	1.93277	12.25882	UTR	4	3666956	3668401	1446	2
FGRAMPH1_01G24949	FGRAMPH1_01T24949	1.96329	13.09371	Promoter	4	3683887	3684702	816	2
ENSRNA049518314	ENSRNA049518314-T1	2.33349	22.3382	Promoter	4	3709555	3709674	120	2
FGRAMPH1_01G24981	FGRAMPH1_01T24981	1.89806	11.9472	Promoter	4	3728363	3728654	292	1
ENSRNA049555776	ENSRNA049555776-T1	2.68056	33.3261	Promoter	4	3740577	3740666	90	2
FGRAMPH1_01G25021	FGRAMPH1_01T25021	2.22216	25.08002	Promoter	4	3775564	3778936	3373	2

FGRAMPH1_01G25023	FGRAMPH1_01T25023	1.64205	6.90505	Promoter	4	3781790	3783963	2174	1
ENSRNA049518319	ENSRNA049518319-T1	2.46003	25.8178	Promoter	4	3792181	3792300	120	2
FGRAMPH1_01G25045	FGRAMPH1_01T25045	2.602	31.66864	Promoter	4	3805928	3806402	475	1
ENSRNA049554981	ENSRNA049554981-T1	2.35257	25.28655	Promoter	4	3893966	3894055	90	1
FGRAMPH1_01G25115	FGRAMPH1_01T25115	2.1964	18.70907	Promoter	4	3903882	3907567	3686	2
FGRAMPH1_01G25119	FGRAMPH1_01T25119	2.25967	20.49161	Promoter	4	3909653	3910707	1055	1
FGRAMPH1_01G25157	FGRAMPH1_01T25157	2.60766	30.33056	Promoter	4	3951630	3953315	1686	1
FGRAMPH1_01G25159	FGRAMPH1_01T25159	1.4334	4.35508	Promoter	4	3954125	3955039	915	2
FGRAMPH1_01G25177	FGRAMPH1_01T25177	1.86877	13.63234	Promoter	4	3972168	3975798	3631	2
ENSRNA049554994	ENSRNA049554994-T1	2.61821	30.75447	Promoter	4	3980295	3980389	95	1
FGRAMPH1_01G25211	FGRAMPH1_01T25211	1.35821	3.18717	Promoter	4	4008956	4011283	2328	1
FGRAMPH1_01G25255	FGRAMPH1_01T25255	1.82731	10.2759	Promoter	4	4060120	4062285	2166	1
FGRAMPH1_01G25259	FGRAMPH1_01T25259	1.74295	8.44624	Promoter	4	4065626	4066801	1176	1

FGRAMPH1_01G25295	FGRAMPH1_01T25295	1.54259	5.27724	Promoter	4	4105371	4106288	918	2
FGRAMPH1_01G25335	FGRAMPH1_01T25335	2.14458	22.82989	Promoter	4	4147478	4147780	303	2
FGRAMPH1_01G25341	FGRAMPH1_01T25341	2.0804	15.66799	Promoter	4	4153782	4155554	1773	2
FGRAMPH1_01G25351	FGRAMPH1_01T25351	1.55432	7.30689	Promoter	4	4167282	4168803	1522	1
FGRAMPH1_01G25353	FGRAMPH1_01T25353	1.66913	7.23732	Promoter	4	4169510	4170136	627	1
ENSRNA049555013	ENSRNA049555013-T1	2.8502	38.29288	Promoter	4	4181019	4181092	74	1
ENSRNA049555023	ENSRNA049555023-T1	2.35458	23.09437	Promoter	4	4196503	4196592	90	1
FGRAMPH1_01G25389	FGRAMPH1_01T25389	1.77459	9.21114	Promoter	4	4201234	4202637	1404	2
ENSRNA049555756	ENSRNA049555756-T1	2.21749	19.06036	Promoter	4	4232727	4232816	90	2
ENSRNA049555039	ENSRNA049555039-T1	2.5233	27.83449	Promoter	4	4259876	4259968	93	1
FGRAMPH1_01G25449	FGRAMPH1_01T25449	1.74295	8.44624	Promoter	4	4261045	4261462	418	1
ENSRNA049555073	ENSRNA049555073-T1	2.02638	18.07569	Promoter	4	4261370	4261452	83	1
FGRAMPH1_01G25481	FGRAMPH1_01T25481	1.81896	11.29629	Promoter	4	4288231	4292485	4255	1

ENSRNA049555088	ENSRNA049555088-T1	2.53385	28.2448	Promoter	4	4418032	4418103	72	1
FGRAMPH1_01G25615	FGRAMPH1_01T25615	1.81594	11.46415	Promoter	4	4444319	4444515	197	1
ENSRNA049555110	ENSRNA049555110-T1	2.50221	27.42648	Promoter	4	4452358	4452447	90	1
FGRAMPH1_01G25641	FGRAMPH1_01T25641	1.67968	7.4724	Promoter	4	4472041	4473853	1813	1
FGRAMPH1_01G25651	FGRAMPH1_01T25651	2.27021	20.49161	Promoter	4	4481283	4482777	1495	2
FGRAMPH1_01G25695	FGRAMPH1_01T25695	1.87599	12.16115	Promoter	4	4536444	4537165	722	1
FGRAMPH1_01G25697	FGRAMPH1_01T25697	1.3648	3.87295	Exon	4	4537358	4538671	1314	2
FGRAMPH1_01G25771	FGRAMPH1_01T25771	2.10149	16.32472	Promoter	4	4626839	4627726	888	1
FGRAMPH1_01G25777	FGRAMPH1_01T25777	1.79279	9.7512	Promoter	4	4634874	4637182	2309	2
ENSRNA049555125	ENSRNA049555125-T1	2.50221	27.42648	Promoter	4	4650987	4651068	82	1
FGRAMPH1_01G25795	FGRAMPH1_01T25795	1.46531	5.16839	Promoter	4	4664174	4665453	1280	1
ENSRNA049555742	ENSRNA049555742-T1	2.47057	26.21645	Promoter	4	4672894	4672992	99	2
ENSRNA049555158	ENSRNA049555158-T1	2.60766	30.33056	Promoter	4	4676656	4676727	72	1

FGRAMPH1_01G25843	FGRAMPH1_01T25843	1.88004	11.39068	Exon	4	4719765	4721658	1894	1
FGRAMPH1_01G25871	FGRAMPH1_01T25871	1.66378	7.45539	Promoter	4	4747486	4748619	1134	2
ENSRNA049555160	ENSRNA049555160-T1	2.77639	36.01199	Promoter	4	4768357	4768428	72	1
ENSRNA049555711	ENSRNA049555711-T1	2.40453	28.51369	Promoter	4	4769127	4769227	101	2
FGRAMPH1_01G25911	FGRAMPH1_01T25911	1.6446	7.89	Promoter	4	4788232	4790664	2433	2
FGRAMPH1_01G25915	FGRAMPH1_01T25915	2.1375	17.91772	Promoter	4	4795389	4796850	1462	1
FGRAMPH1_01G25917	FGRAMPH1_01T25917	1.51863	5.65284	Promoter	4	4800039	4800707	669	2
FGRAMPH1_01G25955	FGRAMPH1_01T25955	2.28048	21.02127	Promoter	4	4836453	4837442	990	1
FGRAMPH1_01G25969	FGRAMPH1_01T25969	1.78874	10.67171	Intergenic	4	4852572	4852901	330	1
ENSRNA049518317	ENSRNA049518317-T1	2.22803	19.41428	Promoter	4	4870866	4870985	120	1
FGRAMPH1_01G25995	FGRAMPH1_01T25995	1.41304	3.9276	Promoter	4	4881506	4883154	1649	1
FGRAMPH1_01G25997	FGRAMPH1_01T25997	1.95777	14.81301	Promoter	4	4884291	4884945	655	1
FGRAMPH1_01G25999	FGRAMPH1_01T25999	2.40306	24.55415	Promoter	4	4885785	4889613	3829	1

FGRAMPH1_01G26003	FGRAMPH1_01T26003	1.4113	4.58123	Promoter	4	4893101	4896406	3306	2
FGRAMPH1_01G26005	FGRAMPH1_01T26005	1.78871	12.40103	Exon	4	4896415	4897152	738	1
FGRAMPH1_01G26009	FGRAMPH1_01T26009	1.84817	11.86685	Promoter	4	4903440	4905191	1752	2
FGRAMPH1_01G26011	FGRAMPH1_01T26011	1.28921	3.13871	Promoter	4	4906309	4907628	1320	1
ENSRNA049555186	ENSRNA049555186-T1	2.36691	25.92933	Promoter	4	4913434	4913516	83	1
FGRAMPH1_01G26045	FGRAMPH1_01T26045	2.27021	20.49161	Promoter	4	4972851	4975161	2311	2
FGRAMPH1_01G26047	FGRAMPH1_01T26047	1.49593	5.01882	Exon	4	4975392	4982669	7278	2
FGRAMPH1_01G26049	FGRAMPH1_01T26049	1.33079	3.84661	Promoter	4	4983141	4983753	613	2
FGRAMPH1_01G26051	FGRAMPH1_01T26051	2.4012	24.31531	Exon	4	4983925	4984904	980	1
ENSRNA049518329	ENSRNA049518329-T1	2.71312	33.78359	Promoter	4	4990442	4990556	115	2
FGRAMPH1_01G26061	FGRAMPH1_01T26061	1.72858	9.11924	Promoter	4	4994659	4996462	1804	2
FGRAMPH1_01G26065	FGRAMPH1_01T26065	2.126	23.49626	Promoter	4	4997410	5000809	3400	1
FGRAMPH1_01G26067	FGRAMPH1_01T26067	1.46689	4.51462	Promoter	4	5001005	5002699	1695	1

FGRAMPH1_01G26081	FGRAMPH1_01T26081	1.83579	10.76108	Promoter	4	5016957	5018940	1984	1
FGRAMPH1_01G26101	FGRAMPH1_01T26101	1.68042	10.08525	Promoter	4	5039061	5039529	469	1
FGRAMPH1_01G26105	FGRAMPH1_01T26105	1.61275	7.58715	Promoter	4	5042226	5043960	1735	1
FGRAMPH1_01G26109	FGRAMPH1_01T26109	1.64804	6.77735	UTR	4	5105735	5106734	1000	2
FGRAMPH1_01G26111	FGRAMPH1_01T26111	1.39395	4.32741	Intergenic	4	5108591	5109131	541	1
FGRAMPH1_01G26125	FGRAMPH1_01T26125	2.04869	15.75398	Promoter	4	5129357	5131469	2113	2
FGRAMPH1_01G26135	FGRAMPH1_01T26135	1.63139	6.8772	UTR	4	5142282	5143004	723	2
FGRAMPH1_01G26137	FGRAMPH1_01T26137	1.74287	9.2455	Promoter	4	5144277	5146848	2572	1
FGRAMPH1_01G26145	FGRAMPH1_01T26145	2.20611	20.83307	Promoter	4	5155335	5157747	2413	2
FGRAMPH1_01G26157	FGRAMPH1_01T26157	1.2993	3.43622	Intergenic	4	5171150	5171554	405	1
FGRAMPH1_01G26159	FGRAMPH1_01T26159	1.45036	4.95496	Promoter	4	5174979	5175792	814	1
FGRAMPH1_01G26165	FGRAMPH1_01T26165	1.70077	7.71086	Promoter	4	5182387	5183832	1446	2
FGRAMPH1_01G26167	FGRAMPH1_01T26167	1.72521	12.72385	Promoter	4	5186572	5188634	2063	2

FGRAMPH1_01G26169	FGRAMPH1_01T26169	2.37567	23.47615	Promoter	4	5188845	5191351	2507	2
FGRAMPH1_01G26171	FGRAMPH1_01T26171	1.33291	4.19488	UTR	4	5193065	5194655	1591	2
FGRAMPH1_01G26173	FGRAMPH1_01T26173	1.61641	6.33116	Promoter	4	5196731	5199368	2638	2
FGRAMPH1_01G26177	FGRAMPH1_01T26177	1.97495	13.45803	Promoter	4	5201452	5203205	1754	2
FGRAMPH1_01G26179	FGRAMPH1_01T26179	1.307	3.52704	Promoter	4	5205590	5207907	2318	1
FGRAMPH1_01G26189	FGRAMPH1_01T26189	1.82731	10.2759	Promoter	4	5219132	5219930	799	2
FGRAMPH1_01G26191	FGRAMPH1_01T26191	1.70077	7.71086	Promoter	4	5221023	5222102	1080	1
FGRAMPH1_01G26197	FGRAMPH1_01T26197	2.72366	34.22501	Promoter	4	5226876	5229064	2189	2
FGRAMPH1_01G26213	FGRAMPH1_01T26213	1.64804	6.77735	Promoter	4	5246127	5247173	1047	2
FGRAMPH1_01G26235	FGRAMPH1_01T26235	1.67086	9.55734	Promoter	4	5273035	5273812	778	2
FGRAMPH1_01G26237	FGRAMPH1_01T26237	2.39413	25.41735	Promoter	4	5276916	5279801	2886	1
FGRAMPH1_01G26255	FGRAMPH1_01T26255	1.58288	6.77774	Promoter	4	5313165	5315778	2614	1
FGRAMPH1_01G26277	FGRAMPH1_01T26277	1.65433	9.21902	Promoter	4	5345976	5349473	3498	1

FGRAMPH1_01G26295	FGRAMPH1_01T26295	1.44284	4.22855	Promoter	4	5374861	5376859	1999	2
FGRAMPH1_01G26297	FGRAMPH1_01T26297	1.44671	4.20283	Exon	4	5378347	5378661	315	2
FGRAMPH1_01G26317	FGRAMPH1_01T26317	2.04582	17.37657	Promoter	4	5396574	5398576	2003	1
FGRAMPH1_01G26327	FGRAMPH1_01T26327	1.49414	5.96346	Promoter	4	5413913	5415307	1395	1
ENSRNA049518327	ENSRNA049518327-T1	2.59398	30.67884	Promoter	4	5491911	5492054	144	1
FGRAMPH1_01G26381	FGRAMPH1_01T26381	1.91537	13.16189	Promoter	4	5493571	5495805	2235	2
FGRAMPH1_01G26383	FGRAMPH1_01T26383	1.48808	6.50692	Promoter	4	5499495	5503013	3519	1
FGRAMPH1_01G26389	FGRAMPH1_01T26389	1.60631	7.18483	Promoter	4	5507989	5510212	2224	2
FGRAMPH1_01G26397	FGRAMPH1_01T26397	1.54375	5.89786	Exon	4	5519779	5520904	1126	2
FGRAMPH1_01G26401	FGRAMPH1_01T26401	1.68429	8.19806	Promoter	4	5524002	5525633	1632	1
FGRAMPH1_01G26419	FGRAMPH1_01T26419	1.4887	5.38837	Promoter	4	5543379	5546024	2646	2
FGRAMPH1_01G26421	FGRAMPH1_01T26421	1.57996	6.60709	Promoter	4	5547793	5548495	703	1
FGRAMPH1_01G26443	FGRAMPH1_01T26443	2.71946	36.37998	Promoter	4	5588777	5592420	3644	2

FGRAMPH1_01G26445	FGRAMPH1_01T26445	2.06748	18.8406	Promoter	4	5597936	5598579	644	1
FGRAMPH1_01G26447	FGRAMPH1_01T26447	1.87629	15.88547	Exon	4	5599681	5600832	1152	1
FGRAMPH1_01G26457	FGRAMPH1_01T26457	1.57774	6.11506	Promoter	4	5618520	5621339	2820	1
FGRAMPH1_01G26459	FGRAMPH1_01T26459	1.8704	13.40454	Promoter	4	5620746	5621497	752	2
FGRAMPH1_01G26481	FGRAMPH1_01T26481	1.69022	7.4724	Promoter	4	5658331	5660494	2164	2
FGRAMPH1_01G26491	FGRAMPH1_01T26491	1.60408	6.20006	Promoter	4	5674944	5675666	723	1
FGRAMPH1_01G26493	FGRAMPH1_01T26493	2.59121	30.41372	Promoter	4	5676521	5679176	2656	1
FGRAMPH1_01G26541	FGRAMPH1_01T26541	2.11215	17.30197	Promoter	4	5734128	5736123	1996	1
FGRAMPH1_01G26551	FGRAMPH1_01T26551	1.66913	7.23732	Promoter	4	5749038	5749541	504	1
ENSRNA049555204	ENSRNA049555204-T1	2.67094	32.47228	Promoter	4	5795223	5795318	96	1
ENSRNA049555215	ENSRNA049555215-T1	2.43482	25.51577	Promoter	4	5801288	5801359	72	1
FGRAMPH1_01G26613	FGRAMPH1_01T26613	1.60067	9.16291	Promoter	4	5824059	5825857	1799	1
FGRAMPH1_01G26623	FGRAMPH1_01T26623	1.65982	7.36202	Promoter	4	5843187	5844830	1644	2

FGRAMPH1_01G26625	FGRAMPH1_01T26625	2.02725	15.78392	Promoter	4	5846844	5850300	3457	1
FGRAMPH1_01G26649	FGRAMPH1_01T26649	2.17531	18.0144	Intergenic	4	5878578	5879285	708	1
FGRAMPH1_01G26661	FGRAMPH1_01T26661	1.56495	5.84504	Promoter	4	5890571	5892233	1663	1
FGRAMPH1_01G26669	FGRAMPH1_01T26669	1.74295	8.44624	Promoter	4	5907822	5912064	4243	1
FGRAMPH1_01G26671	FGRAMPH1_01T26671	1.76404	8.95294	Promoter	4	5914638	5918548	3911	1
ENSRNA049555255	ENSRNA049555255-T1	2.8502	38.29288	Promoter	4	5925108	5925179	72	1
FGRAMPH1_01G26683	FGRAMPH1_01T26683	2.1964	18.70907	Promoter	4	5930613	5933608	2996	1
FGRAMPH1_01G26691	FGRAMPH1_01T26691	1.87706	11.98857	Promoter	4	5957905	5962648	4744	2
FGRAMPH1_01G26693	FGRAMPH1_01T26693	1.91595	13.29526	Promoter	4	5963343	5963775	433	1
FGRAMPH1_01G26699	FGRAMPH1_01T26699	1.57269	6.85383	Promoter	4	5967613	5970248	2636	2
FGRAMPH1_01G26717	FGRAMPH1_01T26717	2.59172	30.42399	Promoter	4	5988717	5992760	4044	1
FGRAMPH1_01G26723	FGRAMPH1_01T26723	1.70441	9.57406	Promoter	4	6001676	6002421	746	1
FGRAMPH1_01G26727	FGRAMPH1_01T26727	1.30487	3.55995	Promoter	4	6004172	6007366	3195	2

FGRAMPH1_01G26737	FGRAMPH1_01T26737	1.70731	10.87856	Promoter	4	6014592	6015242	651	2
ENSRNA049518328	ENSRNA049518328-T1	2.21357	23.66715	Promoter	4	6015998	6016146	149	1
FGRAMPH1_01G26743	FGRAMPH1_01T26743	2.50025	32.00057	Promoter	4	6024895	6027826	2932	2
FGRAMPH1_01G26749	FGRAMPH1_01T26749	1.99741	18.50462	Promoter	4	6037582	6039638	2057	2
FGRAMPH1_01G26759	FGRAMPH1_01T26759	1.48986	4.49906	Promoter	4	6051381	6061287	9907	1
FGRAMPH1_01G26761	FGRAMPH1_01T26761	1.79633	12.98022	Exon	4	6063379	6066153	2775	1
FGRAMPH1_01G26765	FGRAMPH1_01T26765	2.06985	15.66799	Promoter	4	6069412	6072443	3032	2
ENSRNA049555276	ENSRNA049555276-T1	2.58657	29.90888	Promoter	4	6078455	6078549	95	1
FGRAMPH1_01G26779	FGRAMPH1_01T26779	1.76891	10.01988	Promoter	4	6086093	6090276	4184	2
FGRAMPH1_01G26781	FGRAMPH1_01T26781	2.11172	19.73471	Promoter	4	6093324	6096735	3412	2
FGRAMPH1_01G26783	FGRAMPH1_01T26783	1.80491	12.43702	Promoter	4	6097474	6099587	2114	1
FGRAMPH1_01G26799	FGRAMPH1_01T26799	1.57423	5.68813	Promoter	4	6125208	6128286	3079	1
FGRAMPH1_01G26801	FGRAMPH1_01T26801	1.91047	12.7963	Promoter	4	6128543	6130691	2149	2

FGRAMPH1_01G26803	FGRAMPH1_01T26803	1.40737	3.80238	Promoter	4	6132767	6135600	2834	1
FGRAMPH1_01G26805	FGRAMPH1_01T26805	1.62701	7.1323	Promoter	4	6136047	6137301	1255	1
FGRAMPH1_01G26809	FGRAMPH1_01T26809	1.54981	6.83595	Promoter	4	6140429	6142595	2167	1
ENSRNA049555308	ENSRNA049555308-T1	1.96273	14.60174	Promoter	4	6151679	6151773	95	1
FGRAMPH1_01G26843	FGRAMPH1_01T26843	1.74629	8.64515	Promoter	4	6180398	6182200	1803	1
FGRAMPH1_01G26845	FGRAMPH1_01T26845	1.76666	9.56591	UTR	4	6183390	6186796	3407	1
FGRAMPH1_01G26855	FGRAMPH1_01T26855	1.72201	9.54296	Promoter	4	6192856	6195088	2233	2
FGRAMPH1_01G26857	FGRAMPH1_01T26857	1.9558	16.10681	Promoter	4	6196500	6202159	5660	1
FGRAMPH1_01G26883	FGRAMPH1_01T26883	1.73419	10.38566	Intergenic	4	6243566	6244340	775	2
FGRAMPH1_01G26885	FGRAMPH1_01T26885	1.58196	8.40512	Promoter	4	6246269	6248352	2084	2
FGRAMPH1_01G26887	FGRAMPH1_01T26887	2.14463	19.48373	Promoter	4	6251103	6251270	168	1
FGRAMPH1_01G26901	FGRAMPH1_01T26901	2.0804	15.66799	Promoter	4	6270596	6275383	4788	1
FGRAMPH1_01G26923	FGRAMPH1_01T26923	1.50041	4.49906	Promoter	4	6307004	6308401	1398	2

FGRAMPH1_01G26927	FGRAMPH1_01T26927	1.77379	11.15141	Promoter	4	6310498	6312475	1978	2
FGRAMPH1_01G26931	FGRAMPH1_01T26931	1.62503	7.58451	Promoter	4	6314652	6314864	213	1
FGRAMPH1_01G26947	FGRAMPH1_01T26947	1.42915	4.54714	Exon	4	6328559	6329062	504	1
FGRAMPH1_01G26969	FGRAMPH1_01T26969	1.43504	4.6179	Promoter	4	6362489	6363959	1471	1
FGRAMPH1_01G26993	FGRAMPH1_01T26993	2.00912	15.41228	Promoter	4	6391532	6393950	2419	1
FGRAMPH1_01G27037	FGRAMPH1_01T27037	1.77571	12.29417	Promoter	4	6448084	6448345	262	2
FGRAMPH1_01G27039	FGRAMPH1_01T27039	1.40915	5.09868	Promoter	4	6448485	6451276	2792	2
FGRAMPH1_01G27041	FGRAMPH1_01T27041	1.80806	12.17265	Promoter	4	6452497	6452706	210	1
FGRAMPH1_01G27043	FGRAMPH1_01T27043	2.12325	19.32616	Promoter	4	6455018	6458408	3391	1
ENSRNA049555699	ENSRNA049555699-T1	2.35307	25.63532	Promoter	4	6459445	6459539	95	2
FGRAMPH1_01G27047	FGRAMPH1_01T27047	1.5381	5.69453	Promoter	4	6461293	6463676	2384	1
FGRAMPH1_01G27053	FGRAMPH1_01T27053	1.6772	8.53226	Promoter	4	6475090	6476688	1599	1
ENSRNA049555317	ENSRNA049555317-T1	2.60636	30.51383	Promoter	4	6493100	6493194	95	1

FGRAMPH1_01G27067	FGRAMPH1_01T27067	1.48734	5.92079	Promoter	4	6493548	6495611	2064	2
ENSRNA049555663	ENSRNA049555663-T1	2.33789	25.2915	Promoter	4	6496311	6496393	83	2
FGRAMPH1_01G27089	FGRAMPH1_01T27089	2.49167	27.0208	Intergenic	4	6549290	6549660	371	1
FGRAMPH1_01G27091	FGRAMPH1_01T27091	1.46149	6.57733	Exon	4	6551980	6552535	556	2
FGRAMPH1_01G27093	FGRAMPH1_01T27093	2.00658	14.07517	Promoter	4	6553078	6554727	1650	2
FGRAMPH1_01G27119	FGRAMPH1_01T27119	1.70137	8.59619	UTR	4	6579847	6581988	2142	2
FGRAMPH1_01G27121	FGRAMPH1_01T27121	1.93453	13.3341	Promoter	4	6581952	6582394	443	2
FGRAMPH1_01G27155	FGRAMPH1_01T27155	1.80622	9.73718	Promoter	4	6623322	6627732	4411	1
FGRAMPH1_01G27157	FGRAMPH1_01T27157	1.99604	13.76516	Promoter	4	6627128	6627637	510	2
FGRAMPH1_01G27165	FGRAMPH1_01T27165	1.31539	4.28121	Promoter	4	6644275	6645929	1655	1
FGRAMPH1_01G27177	FGRAMPH1_01T27177	1.68116	7.57242	Promoter	4	6675607	6677526	1920	2
FGRAMPH1_01G27179	FGRAMPH1_01T27179	1.3629	3.70866	Promoter	4	6678253	6679945	1693	2
FGRAMPH1_01G27181	FGRAMPH1_01T27181	1.79543	13.62129	Promoter	4	6682252	6683956	1705	1

FGRAMPH1_01G27183	FGRAMPH1_01T27183	2.22803	19.41428	Promoter	4	6684667	6687307	2641	1
FGRAMPH1_01G27239	FGRAMPH1_01T27239	2.43894	25.42153	Promoter	4	6762991	6763250	260	2
FGRAMPH1_01G27241	FGRAMPH1_01T27241	1.92222	12.25882	Promoter	4	6766083	6767767	1685	1
FGRAMPH1_01G27243	FGRAMPH1_01T27243	2.04876	15.02239	Promoter	4	6768209	6769747	1539	1
ENSRNA049555638	ENSRNA049555638-T1	1.88524	13.6421	Promoter	4	6793481	6793571	91	2
FGRAMPH1_01G27281	FGRAMPH1_01T27281	1.62695	6.55252	Promoter	4	6814061	6815756	1696	1
FGRAMPH1_01G27321	FGRAMPH1_01T27321	1.74029	10.34701	Promoter	4	6854438	6855522	1085	1
FGRAMPH1_01G27341	FGRAMPH1_01T27341	2.77639	36.01199	Promoter	4	6884367	6884947	581	2
FGRAMPH1_01G27343	FGRAMPH1_01T27343	1.8484	10.54995	Promoter	4	6887055	6888881	1827	1
ENSRNA049555621	ENSRNA049555621-T1	2.71312	33.78359	Promoter	4	6889230	6889311	82	2
FGRAMPH1_01G27381	FGRAMPH1_01T27381	1.59729	8.48501	Exon	4	6935186	6935696	511	2
FGRAMPH1_01G27383	FGRAMPH1_01T27383	1.75256	10.31176	Exon	4	6935891	6937637	1747	2
FGRAMPH1_01G27385	FGRAMPH1_01T27385	1.49571	7.18813	Promoter	4	6937911	6938373	463	1

FGRAMPH1_01G27411	FGRAMPH1_01T27411	1.26595	3.07248	Promoter	4	6968143	6970162	2020	2
FGRAMPH1_01G27421	FGRAMPH1_01T27421	1.64302	8.43115	Promoter	4	6986181	6988705	2525	2
FGRAMPH1_01G27439	FGRAMPH1_01T27439	1.69191	9.08926	Promoter	4	7013684	7014424	741	2
FGRAMPH1_01G27455	FGRAMPH1_01T27455	1.77459	9.21114	Promoter	4	7030532	7031944	1413	2
FGRAMPH1_01G27457	FGRAMPH1_01T27457	1.83786	10.2759	Promoter	4	7033248	7035653	2406	2
FGRAMPH1_01G27459	FGRAMPH1_01T27459	2.53385	28.2448	Promoter	4	7035806	7036586	781	2
FGRAMPH1_01G27465	FGRAMPH1_01T27465	1.39875	5.71955	Promoter	4	7042746	7045897	3152	2
FGRAMPH1_01G27467	FGRAMPH1_01T27467	2.27021	20.49161	Promoter	4	7046436	7048439	2004	2
FGRAMPH1_01G27481	FGRAMPH1_01T27481	1.43348	5.45338	UTR	4	7062724	7065155	2432	1
FGRAMPH1_01G27483	FGRAMPH1_01T27483	1.52053	4.88564	Promoter	4	7065522	7066905	1384	2
FGRAMPH1_01G27485	FGRAMPH1_01T27485	1.93766	13.42834	Promoter	4	7068764	7068943	180	2
FGRAMPH1_01G27487	FGRAMPH1_01T27487	1.85483	15.33052	Promoter	4	7070067	7070681	615	1
FGRAMPH1_01G27489	FGRAMPH1_01T27489	2.46111	29.78778	Intergenic	4	7075243	7075852	610	2

FGRAMPH1_01G27491	FGRAMPH1_01T27491	2.22212	20.39288	Intergenic	4	7077949	7078498	550	2
FGRAMPH1_01G27493	FGRAMPH1_01T27493	1.64901	6.99999	Promoter	4	7081647	7083621	1975	1
FGRAMPH1_01G27513	FGRAMPH1_01T27513	1.71434	8.75322	Promoter	4	7108370	7111422	3053	2
FGRAMPH1_01G27517	FGRAMPH1_01T27517	2.03238	20.42427	Promoter	4	7117550	7119544	1995	1
FGRAMPH1_01G27537	FGRAMPH1_01T27537	1.57423	5.68813	Exon	4	7142564	7143162	599	1
FGRAMPH1_01G27545	FGRAMPH1_01T27545	1.70077	7.71086	Promoter	4	7149464	7153285	3822	2
FGRAMPH1_01G27549	FGRAMPH1_01T27549	1.81424	10.48163	Promoter	4	7156972	7158172	1201	1
FGRAMPH1_01G27571	FGRAMPH1_01T27571	2.12064	18.29047	Promoter	4	7173371	7173962	592	1
FGRAMPH1_01G27573	FGRAMPH1_01T27573	1.68541	8.92929	Promoter	4	7174058	7176555	2498	2
ENSRNA049555380	ENSRNA049555380-T1	2.61821	30.75447	Promoter	4	7248898	7248981	84	1
ENSRNA049518315	ENSRNA049518315-T1	2.13313	16.99246	Promoter	4	7250368	7250487	120	1
FGRAMPH1_01G27641	FGRAMPH1_01T27641	1.95214	13.85173	Promoter	4	7267642	7268467	826	2
FGRAMPH1_01G27643	FGRAMPH1_01T27643	1.49933	5.76761	Exon	4	7269898	7271188	1291	2

FGRAMPH1_01G27655	FGRAMPH1_01T27655	2.10516	17.47029	Promoter	4	7280694	7281825	1132	2
FGRAMPH1_01G27667	FGRAMPH1_01T27667	1.97587	14.62148	UTR	4	7293457	7293645	189	1
FGRAMPH1_01G27669	FGRAMPH1_01T27669	1.73975	10.18891	Promoter	4	7294308	7296138	1831	2
ENSRNA049555607	ENSRNA049555607-T1	2.02394	16.09714	Promoter	4	7299074	7299156	83	2
FGRAMPH1_01G27673	FGRAMPH1_01T27673	1.77459	9.21114	Promoter	4	7299586	7303503	3918	2
FGRAMPH1_01G27685	FGRAMPH1_01T27685	1.62876	7.12585	Promoter	4	7313795	7316203	2409	2
ENSRNA049555580	ENSRNA049555580-T1	2.01219	14.73115	Promoter	4	7325003	7325085	83	2
ENSRNA049555388	ENSRNA049555388-T1	2.43894	25.42153	Promoter	4	7329870	7329969	100	1
ENSRNA049518322	ENSRNA049518322-T1	2.03218	15.35781	Promoter	4	7395116	7395235	120	2
ENSRNA049555552	ENSRNA049555552-T1	2.25967	20.49161	Promoter	4	7395568	7395649	82	2
FGRAMPH1_01G27765	FGRAMPH1_01T27765	1.46067	4.97128	Promoter	4	7399441	7402227	2787	1
FGRAMPH1_01G27775	FGRAMPH1_01T27775	1.8346	11.50031	Promoter	4	7409003	7410925	1923	1
FGRAMPH1_01G27779	FGRAMPH1_01T27779	1.69368	8.43448	Promoter	4	7413181	7413507	327	2

ENSRNA049555412	ENSRNA049555412-T1	2.52203	29.17997	Promoter	4	7435087	7435190	104	1
ENSRNA049555542	ENSRNA049555542-T1	2.27021	20.49161	Promoter	4	7470028	7470131	104	2
ENSRNA049555507	ENSRNA049555507-T1	2.48112	26.61745	Promoter	4	7477567	7477666	100	2
FGRAMPH1_01G27843	FGRAMPH1_01T27843	1.6287	6.83989	Promoter	4	7484849	7488712	3864	1
ENSRNA049518324	ENSRNA049518324-T1	2.5233	27.83449	Promoter	4	7616888	7617007	120	2
FGRAMPH1_01G27975	FGRAMPH1_01T27975	1.89058	11.39068	Promoter	4	7633555	7634624	1070	2
FGRAMPH1_01G27977	FGRAMPH1_01T27977	1.77669	9.93203	Promoter	4	7635871	7637364	1494	1
FGRAMPH1_01G28001	FGRAMPH1_01T28001	1.58703	7.10083	Intergenic	4	7662992	7663516	525	1
FGRAMPH1_01G28067	FGRAMPH1_01T28067	2.19683	24.21486	Promoter	4	7741035	7741694	660	1
FGRAMPH1_01G28065	FGRAMPH1_01T28065	1.71613	10.06031	Promoter	4	7738883	7741426	2544	2
FGRAMPH1_01G28073	FGRAMPH1_01T28073	1.80291	10.3493	Promoter	4	7744727	7746142	1416	1
FGRAMPH1_01G28075	FGRAMPH1_01T28075	1.45405	6.30861	Promoter	4	7747089	7748600	1512	2
ENSRNA049555427	ENSRNA049555427-T1	2.39676	24.24705	Promoter	4	7777984	7778055	72	1

FGRAMPH1_01G28121	FGRAMPH1_01T28121	2.08209	15.98753	Promoter	4	7828617	7831042	2426	1
FGRAMPH1_01G28149	FGRAMPH1_01T28149	1.99604	13.76516	Promoter	4	7854186	7856609	2424	2
FGRAMPH1_01G28151	FGRAMPH1_01T28151	2.59712	30.33056	Promoter	4	7858886	7861250	2365	1
FGRAMPH1_01G28195	FGRAMPH1_01T28195	2.00658	14.07517	Promoter	4	7906102	7910083	3982	1
FGRAMPH1_01G28197	FGRAMPH1_01T28197	1.5357	5.89433	UTR	4	7906591	7907310	720	1
FGRAMPH1_01G28205	FGRAMPH1_01T28205	1.66853	8.95427	Promoter	4	7916383	7916679	297	2
FGRAMPH1_01G28207	FGRAMPH1_01T28207	1.57085	6.12206	Promoter	4	7917617	7920297	2681	1
FGRAMPH1_01G28225	FGRAMPH1_01T28225	1.36992	4.13173	Promoter	4	7932150	7932605	456	2
FGRAMPH1_01G28231	FGRAMPH1_01T28231	2.00658	14.07517	Promoter	4	7937578	7938429	852	1
ENSRNA049555462	ENSRNA049555462-T1	2.53385	28.2448	Promoter	4	7938595	7938679	85	1
FGRAMPH1_01G28235	FGRAMPH1_01T28235	1.6375	6.77735	Promoter	4	7940688	7942550	1863	2
FGRAMPH1_01G28257	FGRAMPH1_01T28257	1.54816	7.78734	Promoter	4	7963922	7964143	222	1
FGRAMPH1_01G28259	FGRAMPH1_01T28259	1.53366	5.45483	Promoter	4	7964198	7965196	999	2

FGRAMPH1_01G28277	FGRAMPH1_01T28277	2.30302	23.25327	Promoter	4	7987852	7989045	1194	1
FGRAMPH1_01G28279	FGRAMPH1_01T28279	1.66913	7.23732	Promoter	4	7991712	7996758	5047	2
ENSRNA049512110	ENSRNA049512110-T1	1.54458	9.13387	Promoter	5	3184	3350	167	1
ENSRNA049511957	ENSRNA049511957-T1	1.53909	8.93926	Promoter	5	5384	5491	108	1
ENSRNA049557971	ENSRNA049557971-T1	1.34937	4.95131	Promoter	5	7027	7099	73	1
ENSRNA049558024	ENSRNA049558024-T1	1.64772	11.42534	Intergenic	5	8604	8674	71	1
ENSRNA049558045	ENSRNA049558045-T1	1.43412	6.59334	Promoter	5	15545	15616	72	1
ENSRNA049558110	ENSRNA049558110-T1	1.26101	3.06995	Intergenic	5	17768	17840	73	1
ENSRNA049512177	ENSRNA049512177-T1	1.89868	16.78691	Intergenic	5	39890	40117	228	1
ENSRNA049511925	ENSRNA049511925-T1	1.2518	3.07035	Exon	5	54508	54802	295	1
ENSRNA049558136	ENSRNA049558136-T1	1.48074	7.66496	Promoter	5	76168	76238	71	1
ENSRNA049558172	ENSRNA049558172-T1	1.49218	7.10641	Intergenic	5	88872	88942	71	1
ENSRNA049558206	ENSRNA049558206-T1	1.3564	5.1764	Exon	5	95276	95363	88	1
