# Supplementary material for: Transcription Activator FgDDT Interacts With FgISW1 to Regulate Fungal Development and Pathogenicity in the Global Pathogen Fusarium graminearum
Source: Mol Plant Pathol. 2025 Mar 28;26(4):e70076. doi: 10.1111/mpp.70076 (PMC11950633; doi:10.1111/mpp.70076)
Supplement: Supplementary file 6 — Table S2. Primers used in this study. [file MPP-26-e70076-s006.pdf]

Table S2 Primers used in this study

Primer name	Sequence (5'→3')
FgDDT-F1	CGAGATGACGGTATGCCGAGT
FgDDT-R1	CAAAATAGGCATTGATGTGTTGACCTCCACCACCTGCAAGGCCGAAGGT
FgDDT-F2	CTCGTCCGAGGGCAAAGGAATAGAGTAGGCTTGAGTGGTTATTGTCGA
FgDDT-R2	GAGTTGGATCACAGGCGACTTG
FgDDT-F3	TGCACTTCTTGATCCGCCCAC
FgDDT-R3	GAGAACCAAACGCCATGTGT
FgISW1-F1	CTGCTTCCCAAGTCTGAAC
FgISW1-R1	CAAAATAGGCATTGATGTGTTGACCTCCGGAGCGTTTGATTCTCTGC
FgISW1-F2	CTCGTCCGAGGGCAAAGGAATAGAGTAGGAGAAGTCATCGTGCAGTAC
FgISW1-R2	TGTGCGAGGTCACTTCGGT
FgISW1-F3	CTCGCCGACACCTTAGCAAA
FgISW1-R3	CCCTCTTGATGCGATTGCGG
FgISW2-F1	CTCGAGGATGGTGGACTTG
FgISW2-R1	CAAAATAGGCATTGATGTGTTGACCTCCGTGAAAGACAACAAGAGAG
FgISW2-F2	CTCGTCCGAGGGCAAAGGAATAGAGTAGCGATTGGAGGAATCATGACTGG
FgISW2-R2	GGATGTAGAGGCCCAGCTT
FgISW2-F3	GTCAACTACATCATTCTTCCC
FgISW2-R3	TCCATCCTCGCATGTCGAC
FgDDT-ISW1-R1	CCAAAATAGCATTGATGTGTTGACCTCCGGAGCGTTTGATTCTCTGC
FgDDT-ISW1-F2	CTATCGCCTTCTTGACGAGTTCTTCTGAGAGAAGTCATCGTGCAGTAC
FgISW1^{SLIDE}-F1	ACGACTTGATCAGCTGGT
FgISW1^{SLIDE}-R1	CAAAATAGGCATTGATGTGTTGACCTCCGGCAATTTCAGTGTACCTTTGC
FgISW1^{SLIDE}-F2	CTCGTCCGAGGGCAAAGGAATAGAGTAGGAGGATGTTCCTGCTCGTAA
FgISW1^{SLIDE}-R2	ATAGTACGGCGTTTCGGG
FgISW1^{SLIDE}-F3	AAACCGCCTACTACCGCA

FgISW1^{SLIDE}-R3	GACATTGTCAAGCGCCTT
HPH-F	GGAGGTCAACACATCAATGC
HPH-R	CTACTCTATTCCTTTGCCCTCGGACGAG
Neo-F	GGAGGTCAACACATCAATGCT
Neo-R	TCAGAAGAACTCGTCAAGAAG
FgDDT-GFP-F	ACTCACTATAGGGCGAATTGGGTACTCAAATTGGTTGAT GGATCTAAGGCCGTGAG
FgDDT-GFP-R	CACCACCCCGGTGAACAGCTCCTCGCCCTTGCTCACTC GTCGTCGAGTCTTAGCTGCT
FgDDT-Flag-F	CTATAGGGCGAATTGGGTACTCAAATTGGTTGATGGATC TAAGGCCGTGAG
FgDDT-Flag-R	CTTTATAATCACCGTCATGGTCTTTGTAGTCTCGTCGTCG AGTCTTAGCTGCT
FgISW1-GFP-F	TTTCGTAGGAACCCAATCTTCAAAATGGCTCCTCGCTCT CGAGCC
FgISW1-GFP-R	CACCACCCCGGTGAACAGCTCCTCGCCCTTGCTCACCTT CTTTCTACCCTTCTTGC
FgDDT-AD-F	GTACCAGATTACGCTCATATGATGAAACGCAAACCCGTC CGC
FgDDT-AD-R	ATGCCCACCCGGGTGGAATTCTCATCGTCGTCGAGTCTT AGCTG
FgDDT-BD-F	TCAGAGGAGGACCTGCATATGATGAAACGCAAACCCGT CCGC
FgDDT-BD-R	TCGACGGATCCCCGGGAATTCTCATCGTCGTCGAGTCTT AGCTG
FgISW1-BD-F	TCAGAGGAGGACCTGCATATGATGGCTCCTCGCTCTCGA G
FgISW1-BD-R	TCGACGGATCCCCGGGAATTCTCACTTCTTTCTACCCTT CTTGCTC
FgISW2-AD-F	GTACCAGATTACGCTCATATGATGGCGTCGCCGAGTACT G
FgISW2-AD-R	ATGCCCACCCGGGTGGAATTCCTAGTCCTTACGCGCCAT CTTG
FgISW2-BD-F	TCAGAGGAGGACCTGCATATGATGGCGTCGCCGAGTAC TGCC
FgISW2-BD-R	TCGACGGATCCCCGGGAATTCCTAGTCCTTACGCGCCAT CT
FgDDT-DDT-AD-F	GTACCAGATTACGCTCATATGATGGAATCCATCGGACCAT TG

FgDDT-DDT-AD-R	ATGCCCACCCGGGTGGAATTCTCAGTCGGCTTCTGAGTC TACCA
FgISW1-SLIDE-BD-F	TCAGAGGAGGACCTGCATATGATGAAGTACATCAAGAC CATTGAAGAT
FgISW1-SLIDE-BD-R	TCGACGGATCCCCGGGAATTCTCAAAACTCTTTAACAAT TGTAGTGATCA
FgACTIN-ChIP-F	CCTTTCTTTTTCTTCCTCGCC
FgACTIN-ChIP-R	GCGACGTACCCTCCATGGCT
FgACTIN-qRT-F	GGAGATCACTGCTCTTGCTC
FgACTIN-qRT-R	CTGCTTGGAGATCCACATTT
FgMgv1-ChIP-F	CCATACATATATCGCAAGAG
FgMgv1-ChIP-R	GGTAAGAGGGATATAGAGATG
FgMgv1-qRT-F	TCCGTACCGTGCCCGGCCAAA
FgMgv1-qRT-R	TACCATGACCAGCATATTCC
FgHog1-ChIP-F	TACTTTATCTCGCACAGCTC
FgHog1-ChIP-R	GGTATAGATGATGGATGGCG
FgHog1-qRT-F	CCTACCATGACCCTACAGAC
FgHog1-qRT-R	GATAGTCGAGAATTTCCGAGTAC
FgAtf1-ChIP-F	CGTGCTGTTGACCTGTCGAA
FgAtf1-ChIP-R	GTCAAATGAGGTCAGAGGCG
FgAtf1-qRT-F	TCCTTCTCGCTCATAAGGAC
FgAtf1-qRT-R	TAGGTGCTGCCATGCCATAA
FgWee1-ChIP-F	CTTTATCCACGAGGATGCAT
FgWee1-ChIP-R	ACATTGCTGTTTGTGGGTGTTG
FgWee1-qRT-F	GACCAACTGTGCACCAAGTA
FgWee1-qRT-R	CGGAGATAGGAAACATATCGG
FgYck1-ChIP-F	CTTGCCTCTTATGCCTACAGT
FgYck1-ChIP-R	GAAACGCTTTAAACAAATCG
FgYck1-qRT-F	CGCCCACTGGCTCAACTCA
FgYck1-qRT-R	TAGCTGGCTGGCCGCTAGCTT
FgIlv3a-ChIP-F	GTCACAGGGAAGATCACAGG
FgIlv3a-ChIP-R	CGATGCCAAGTTTGTGGAA
FgIlv3a-qRT-F	ACGCCGAGTCTCGTGCTAT
FgIlv3a-qRT-R	GTCGAGCGTACTTGCTGAGT
FgPma1-ChIP-F	AAGCGTCCCTAACTTCGTGC
FgPma1-ChIP-R	GCTGTTTTCTGTTGTTGAGAG
FgPma1-qRT-F	CTTTCGGTGTCTTCTGTGTC
FgPma1-qRT-R	CCTCGAGGGATCGCTGCT
FgPld1-ChIP-F	CCGCTTCTGTCAAATACAAC

FgPld1-ChIP-R	TCGATTGTCGCAGCCAATGC
FgPld1-qRT-F	CTTCTCAACATGGTACAGGG
FgPld1-qRT-R	TTACTAGATAGCCAGGGGTG
FgMet14-ChIP-F	GGGAAGACGGTATGAGTTTT
FgMet14-ChIP-R	GGCAAGTAACCTAGTAACTGTC
FgMet14-qRT-F	TTTACAAGAAGGCTCGCGCC
FgMet14-qRT-R	CACATTCCTCAACAGAGTTCTCG
FgNdpk-ChIP-F	CCAATCGACTCTTTTGTAC
FgNdpk-ChIP-R	GACAAATCAAAAGGTCAAGCAA
FgNdpk-qRT-F	CCTCTTCCCCCGGTACCATC
FgNdpk-qRT-R	CGCCCTCCTTGAACCAGAGA
FgDDT-qRT-F	ATACATCGAACTATCGCAGTCTCC
FgDDT-qRT-R	CCTCACGCTTCCGAGTTTGCTT
FgISW1-qRT-F	TGGCACCAGCAAAGAAGAAA
FgISW1-qRT-R	ACGCAACACTGGATGCTCT
HPH-probe-F	CACCGCGACGTCTGTCGAGAAG
HPH-probe-R	GGACGATTGCGTCGCATCGA
FgDDT-probe-F	CGAGATGACGGTATGCCGAGT
FgDDT-probe-R	ACCACCTGCAAGGCCGAAGGT
Neo-probe-F	GGAGGTCAACACATCAATGCT
Neo-probe-R	CCAGATCATCCTGATCGACAAG
FgDDT-BiFC-F	TTTCGTAGGAACCCAATCTTCAAAATGGTACGCGCTCCC ACTGCT
FgDDT-BiFC-R	GTTCGGGATCTTGCAGGCCGGGCGTCGTCGTCGAGTCT TAGCTG
FgISW1-BiFC-F	TTTCGTAGGAACCCAATCTTCAAAATGGCTCCTCGCTCT CGAG
FgISW1-BiFC-R	GCTCACCATCGTGGCGATGGAGCGCTTCTTTCTACCCTT CTTGCTC
FgDDT-His-F	GACGAGCTGTACAAGGGATCCATGAAACGCAAACCCGT CCGC
FgDDT-His-R	TGGTGGTGCTCGAGTGCGGCCGCTCGTCGTCGAGTCTT AGCTGCTGG
FgHog1-EMSA-F	GCCCGCAGTCTCTGGACT
FgHog1-EMSA-R	GGTGAATATGTGGTTGTTGAGC
FgNdpk-EMSA-F	GTATCTCCGTTGGCATTGAT
FgNdpk-EMSA-R	GATGAGCTGGCGTTGAGG
