# Supplementary material for: Transcription Activator FgDDT Interacts With FgISW1 to Regulate Fungal Development and Pathogenicity in the Global Pathogen Fusarium graminearum
Source: Mol Plant Pathol. 2025 Mar 28;26(4):e70076. doi: 10.1111/mpp.70076 (PMC11950633; doi:10.1111/mpp.70076)
Supplement: Supplementary file 7 — Table S3. Accession numbers for proteins used in the study. [file MPP-26-e70076-s005.pdf]

Table S3 Accession numbers for proteins used in the study

Strains	**Accession numbers**
FgDDT	FGRAMPH1_01G06065
FvDDT	FVEG_09204
FoDDT	FOXG_10556
NcDDT	NCU00164
MoDDT	MGG_05687
AnDDT	ANIA_07303
PdDDT	PDIP_37950
BcDDT	BCIN_13g00680
HvDDT	L207DRAFT_458721
ScDDT	NP_011382.1
DmDDT	Dmel_CG1966
HsDDT	NP_038476.2
AtDDT	At5g35210
FgRPB2	FGRAMPH1_01G06377
FvRPB2	FVEG_09286
FoRPB2	FOXG_10639
MoRPB2	MGG_04714
NcRPB2	B0T23DRAFT_134605
AnRPB2	ANIA_09120
PdRPB2	Pdw03_7672
BcRPB2	BCIN_14g01590
HvRPB2	L207DRAFT_461478
ScRPB2	SPAR_O02800
DmRPB2	Dmel_CG3180
HsRPB2	NP_000929.1
AtRPB2	At4g21710
FgISW1	FGRAMPH1_01G07691
FgISW2	FGRAMPH1_01G16881

ScISW1	NP_009804.1
CHR11	At3g06400
SNF2H	NP_003592.3
